# Dietary risk factors and cancer mortality burden from 1990 to 2021: a comparative study of China and global regions with varying sociodemographic development levels based on the Global Burden of Disease database

**DOI:** 10.3389/fnut.2025.1628792

**Published:** 2025-08-05

**Authors:** Chunxiu Zhao, Qian Ruan, Bingbing Xiang, Xuehe Zhang, Pingliang Yang, Shun Wang

**Affiliations:** ^1^Department of Critical Care Medicine, Affiliated Hospital of Southwest Jiaotong University, The Third People's Hospital of Chengdu, Chengdu, Sichuan, China; ^2^Department of Anesthesiology, Clinical Medical College and The First Affiliated Hospital of Chengdu Medical College, Chengdu, Sichuan, China; ^3^Department of Anesthesiology, West China Hospital, Sichuan University, Chengdu, China

**Keywords:** dietary risk factors, cancer mortality, Global Burden of Disease (GBD), socio-demographic index, regional comparison

## Abstract

**Objective:**

To analyze temporal trends of diet-attributable cancer mortality in China (1990–2021), compare patterns between China and regions with varying development levels, and explore gender-specific characteristics to inform targeted prevention strategies.

**Study design:**

Cross-sectional and time-series analyses.

**Methods:**

We conducted cross-sectional and time-series analyses of nine dietary risk factors across China, the global region, and five Socio-demographic Index (SDI) - stratified regions. Joinpoint regression models quantified temporal trends through Annual Percent Change (APC) and Average Annual Percent Change (AAPC).

**Results:**

Diet-attributable cancer deaths in China decreased from 9.9% (95% CI: 2.2–20.5%) to 6.3% (95% CI: 2.1–12.8%) during 1990–2021. China’s 2021 attribution (6.3%) was below the global average (6.8%), exceeding high-SDI regions (5.8%) but below middle-SDI regions (7.4%). Low vegetable intake showed the largest decline (3.0 to 0.3%), while high red meat consumption increased (1.6 to 2.0%). Red meat’s impact was greater in females, while inadequate plant consumption affected males more significantly. Dietary factors most influenced colorectal cancer in China (39.2%). China’s diet-attributable cancer mortality decreased by 53.0% (from 18.4 to 8.7 per 100,000), exceeding global reductions (35.5%).

**Conclusion:**

This study identified distinct regional patterns in diet-attributable cancer mortality. China’s profile reflects its transition between development levels—decreasing vegetable-deficiency risks while increasing red meat consumption. Globally, attribution patterns are shifting from plant food inadequacy toward animal product excess, with persistent gender disparities.

## Highlights

- China achieved remarkable 53.0% reduction in diet-attributable cancer mortality, exceeding global average (35.5%).
- Vegetable intake deficiency dramatically improved 89% in China while red meat attribution proportion increased 27%, reflecting nutrition transition dynamics.
- Regional patterns revealed development- dependent transitions: high-SDI regions showed declining red meat risks while low- middle SDI exhibited increasing trends.
- Gender disparities persisted universally with red meat predominantly affecting females and plant deficiencies impacting males more severely.

## Introduction

Cancer represents a significant global health burden with profound implications for public health systems worldwide. According to the latest global cancer statistics (GLOBOCAN 2022), approximately 20 million new cancer cases and nearly 9.7 million cancer-related deaths occurred globally in 2022, with projections indicating annual new cancer cases could exceed 35 million by 2050—a 77% increase compared to 2022—primarily due to population growth and aging demographics (1). Multiple epidemiological investigations have established that approximately 40–50% of cancer occurrences are associated with modifiable lifestyle and environmental factors, with dietary behaviors emerging as critical intervention targets (2, 3). Sung and colleagues have demonstrated that global food system transformations significantly elevate risk for 13 cancer types through increased body mass index (BMI) (4).

The mechanisms through which dietary risk factors influence cancer development are multifaceted. Excessive consumption of unhealthy dietary components—including red meat, processed meats, and high sodium—may enhance cancer risk through various pathways. For instance, heme iron in red meat can promote oxidative DNA damage and lipid peroxidation in intestinal cells, inducing inflammatory responses that increase colorectal cancer risk (5, 6). Concurrently, nitrites in processed meats can transform into carcinogenic nitrosamines under gastric acidic conditions, while high-sodium diets may elevate risks of chronic gastric mucosal damage and atrophy (7). Conversely, insufficient intake of protective components—such as vegetables, fruits, whole grains, and dietary fiber—is also associated with increased cancer risk (7, 27, 28).

Dietary patterns exhibit marked variations across global regions, reflecting differences in cultural traditions, economic development levels, and food accessibility. The SDI—a composite indicator reflecting regional socioeconomic development levels—stratifies countries and regions into five developmental categories (8). High-SDI regions demonstrate colorectal cancer burdens significantly associated with elevated red meat consumption, while low-SDI regions experience increased esophageal cancer risk primarily due to inadequate fruit and vegetable consumption (9).

As the world’s most populous developing nation, China has experienced rapid economic growth and urbanization over the past three decades, accompanied by substantial dietary structural transformations (29, 30). Huang and colleagues reported that between 1982 and 2012, Chinese residents’ grain consumption decreased by 34%, while animal food and fat intake increased by 162 and 131%, respectively. Dairy consumption surged by 205%, indicating a dietary transition toward higher fat and animal protein content (10, 29, 30). This westernization of dietary patterns may have altered China’s cancer spectrum and mortality burden (11). However, comparative research on dietary risk factors’ contribution to cancer mortality in China vs. global regions with varying development levels remains limited. Wu and colleagues’ recent GBD 2021-based investigation revealed that dietary risks account for 5.89–7.21% of global cancer-related disability-adjusted life years (DALYs), but their global analytical framework did not independently stratify Chinese data—highlighting the critical need for China-specific research (12).

The Global Burden of Disease research provides a standardized framework for comparing disease burdens across different countries and regions (13). Through the GBD database, systematic assessments of various risk factors’ contributions to disease burden can be conducted, providing scientific evidence for developing targeted public health intervention strategies.

Based on the latest GBD 2021 database, this study aims to: (1) analyze temporal evolution trends of dietary risk factors’ contribution to cancer mortality burden in China from 1990 to 2021 and compare differences in dietary risk factor attribution patterns between China, global regions, and regions with different SDI levels; (2) explore gender-specific characteristics in diet-related cancer mortality and provide scientific evidence for formulating targeted cancer prevention strategies.

## Methods

### Data sources and study design

This study employed a cross-sectional and time-series epidemiological approach utilizing the GBD 2021 database. The GBD study, coordinated by the Institute for Health Metrics and Evaluation (IHME), systematically evaluates the prevalence and health impacts of diseases, injuries, and risk factors worldwide (13, 31). We extracted cancer mortality data attributable to dietary risk factors from 1990 to 2021 for China and five regions stratified by SDI.

### Dietary risk factors

This analysis focuses on the nine dietary risk factors included in the GBD 2021 framework. White meat (poultry) and fish consumption are not classified as dietary risk factors in the GBD database, as current evidence suggests neutral or protective associations with cancer risk. Therefore, our analysis is limited to established dietary risk factors with sufficient evidence for cancer causation.

Harmful Components: High red meat intake [theoretical minimum risk exposure level (TMREL): 200 g/day]: beef, pork, and lamb consumption above optimal levels; High processed meat intake (TMREL: 0 g/day): preserved meats with nitrite/nitrate additives; High sodium intake (TMREL: 5 g/day): above optimal intake levels.

Protective Components: Low fruit intake (below TMREL: 340 g/day); Low vegetable intake (below TMREL: 306 g/day); Low whole grain intake (below TMREL: 160 g/day); Low dietary fiber (below TMREL: 22 g/day); Low calcium intake (below TMREL: 0.72 g/day males, 1.1 g/day females); Low milk intake (below TMREL: 280 g/day males, 500 g/day females). Attribution analyses used the TMREL as a counterfactual reference value (3). For both harmful dietary factors (e.g., high red meat intake) and protective dietary factors (e.g., low fruit intake), we followed the methodological standards established in the GBD 2019 study (3). Dietary exposure data were derived from nationally representative dietary surveys, food balance sheets from the Food and Agriculture Organization (FAO), and nutrition monitoring systems (14, 15).

### Cancer mortality outcomes

The primary outcome indicators were cancer mortality rates attributable to dietary risk factors and their proportion of total cancer mortality. Cancer types analyzed included: overall cancer, esophageal cancer, gastric cancer, colorectal cancer, tracheal-bronchial-lung cancer, breast cancer, and prostate cancer.

### Socioeconomic development stratification and regional framework

The Socio-demographic Index is a composite development indicator developed by GBD researchers, calculated as the geometric mean of three normalized indices (0–1 scale): total fertility rate under age 25 (TFU25), mean education for ages 15 and older (EDU15+), and lag distributed income (LDI) per capita. This metric ranges from 0 (minimal development) to 1 (maximum development), providing a standardized measure for health outcome comparisons across regions.

Regional Classifications and Dietary Transition Characteristics: High SDI regions (>0.81) include post-industrial economies (USA, Western Europe, Japan) characterized by completed demographic transition, service-based economies, and advanced nutrition transition. These regions exhibit processed food-dominant dietary patterns with minimal traditional deficiency risks but elevated modern dietary risks. High-middle SDI regions (0.71–0.81) encompass transitional industrial economies (China, Eastern Europe, Russia) experiencing moderate population aging and mixed traditional-modern dietary patterns. These regions demonstrate intermediate dietary risk profiles reflecting ongoing nutrition transition. Middle SDI regions (0.61–0.71) include rapidly developing economies (Brazil, Mexico) undergoing active nutrition transition with compressed development timelines. These regions exhibit the most dynamic dietary risk evolution, transitioning from deficiency-dominant to excess-dominant patterns within decades rather than centuries. Low-middle (0.46–0.61) and Low SDI regions (<0.46) predominantly feature traditional dietary patterns with persistent plant food deficiencies and minimal modern dietary risks, reflecting limited economic development and food system modernization.

China’s Developmental Context: China’s SDI increased from 0.45 (1990) to 0.72 (2021), representing the most rapid socioeconomic transition globally. This unique trajectory—spanning low-middle to high-middle SDI categories within three decades—provides exceptional opportunity to examine dietary risk evolution during compressed development. China’s current positioning between high-middle and middle SDI regions reflects successful traditional risk mitigation while managing emerging modern dietary challenges, distinguishing it from both developed and developing country patterns (12, 32).

### Statistical analysis

Data processing and statistical analyses were performed using R software (version 4.4.2). We employed joinpoint regression models to analyze temporal trends in the burden of dietary risk factors on cancer mortality from 1990 to 2021. This method determined the optimal number of joinpoints (0–5) based on the Akaike Information Criterion (AIC) minimization principle, calculating the APC for each segment and the AAPC overall. When the 95% confidence interval for APC or AAPC did not include 0, the trend was considered statistically significant.

For period-specific analysis, we divided the study period into four segments (1990–1998, 1999–2007, 2008–2015, and 2016–2021), calculating the mean attribution proportion (Mean ± SE) of dietary risk factors for each period. Regional comparative analyses assessed differences in dietary risk patterns between China, global regions, and different SDI regions, visualized using heatmaps. Gender disparity analysis calculated the ratio of male-to-female attribution proportions for various dietary risk factors.

### Ethical statement

This study utilized publicly accessible aggregate data from the GBD 2021 database, which does not involve individual-level patient information. Therefore, in accordance with the Declaration of Helsinki, ethical review was not required. The research process adhered to scientific research ethical norms and good epidemiological research practices.

## Results

Between 1990 and 2021, China’s cancer mortality attribution from dietary risk factors declined from 9.9% (95% CI: 2.2–20.5%) to 6.3% (95% CI: 2.1–12.8%), demonstrating pronounced epidemiological metamorphosis (Table 1). Vegetable inadequacy exhibited the most substantial amelioration, decreasing from 3.0 to 0.3%, while red meat consumption attribution escalated from 1.6 to 2.0%, emerging as the predominant contemporary risk determinant. Low fruit consumption declined from 1.2 to 0.7%, high sodium intake decreased from 2.1 to 1.3%, while low whole grain intake exhibited slight increase from 1.5 to 1.8%.

**Table 1:** Percentage of total cancer mortality attributable to dietary risk factors—China (Age-standardized, 1990–2021).

Year	All dietary risks			Low in fruits			Low in vegetables			Low in whole grains			Low in milk			High in red meat			High in processed meat			Low in fiber			High in sodium			Low in calcium		
	Male	Female	Both	Male	Female	Both	Male	Female	Both	Male	Female	Both	Male	Female	Both	Male	Female	Both	Male	Female	Both	Male	Female	Both	Male	Female	Both	Male	Female	Both
1990	9.8% [2.1–20.4%]	10.1% [2.5–20.1%]	9.9% [2.2–20.5%]	1.3% [0.7–1.9%]	1.0% [0.5–1.5%]	1.2% [0.6–1.8%]	3.3% [−0.8–6.5%]	2.3% [−0.5–4.7%]	3.0% [−0.7–5.9%]	1.4% [0.6–2.1%]	1.7% [0.7–2.6%]	1.5% [0.6–2.3%]	1.0% [0.5–1.5%]	2.2% [0.7–3.6%]	1.5% [0.5–2.4%]	1.1% [−0.0–2.3%]	2.3% [−0.0–4.7%]	1.6% [−0.0–3.3%]	0.1% [−0.0–0.2%]	0.1% [−0.0–0.3%]	0.1% [−0.0–0.3%]	0.1% [0.1–0.2%]	0.2% [0.1–0.3%]	0.1% [0.1–0.2%]	2.2% [−0.0–11.0%]	1.8% [−0.0–8.9%]	2.1% [−0.0–10.2%]	1.1% [0.8–1.4%]	1.7% [1.3–2.2%]	1.4% [1.0–1.7%]
1991	9.7% [2.1–20.3%]	10.0% [2.5–19.9%]	9.8% [2.2–20.4%]	1.3% [0.7–1.9%]	1.0% [0.5–1.5%]	1.2% [0.6–1.7%]	3.3% [−0.8–6.5%]	2.3% [−0.4–4.6%]	2.9% [−0.7–5.8%]	1.4% [0.6–2.1%]	1.7% [0.7–2.6%]	1.5% [0.6–2.3%]	1.0% [0.5–1.5%]	2.2% [0.7–3.6%]	1.5% [0.5–2.4%]	1.1% [−0.0–2.3%]	2.3% [−0.0–4.7%]	1.6% [−0.0–3.3%]	0.1% [−0.0–0.2%]	0.1% [−0.0–0.3%]	0.1% [−0.0–0.3%]	0.1% [0.1–0.2%]	0.2% [0.1–0.3%]	0.1% [0.1–0.2%]	2.2% [−0.0–11.0%]	1.8% [−0.0–8.7%]	2.0% [−0.0–10.1%]	1.1% [0.8–1.4%]	1.7% [1.3–2.1%]	1.3% [1.0–1.6%]
1992	9.5% [2.1–20.0%]	9.9% [2.5–19.7%]	9.7% [2.2–20.1%]	1.3% [0.7–1.9%]	1.0% [0.5–1.5%]	1.2% [0.6–1.7%]	3.2% [−0.8–6.3%]	2.2% [−0.4–4.5%]	2.8% [−0.6–5.7%]	1.4% [0.6–2.1%]	1.7% [0.7–2.6%]	1.5% [0.6–2.3%]	1.0% [0.5–1.5%]	2.2% [0.7–3.6%]	1.5% [0.5–2.5%]	1.2% [−0.0–2.3%]	2.3% [−0.0–4.8%]	1.6% [−0.0–3.3%]	0.1% [−0.0–0.2%]	0.1% [−0.0–0.3%]	0.1% [−0.0–0.3%]	0.1% [0.1–0.2%]	0.2% [0.1–0.3%]	0.1% [0.1–0.2%]	2.2% [−0.0–10.8%]	1.7% [−0.0–8.5%]	2.0% [−0.0–9.9%]	1.1% [0.8–1.4%]	1.7% [1.2–2.1%]	1.3% [1.0–1.6%]
1993	9.4% [2.1–19.6%]	9.8% [2.5–19.4%]	9.6% [2.2–19.8%]	1.3% [0.6–1.8%]	1.0% [0.5–1.5%]	1.1% [0.6–1.7%]	3.1% [−0.7–6.2%]	2.1% [−0.4–4.4%]	2.7% [−0.6–5.4%]	1.4% [0.6–2.1%]	1.7% [0.7–2.6%]	1.5% [0.6–2.3%]	1.0% [0.5–1.5%]	2.2% [0.6–3.6%]	1.5% [0.5–2.4%]	1.2% [−0.0–2.3%]	2.4% [−0.0–4.8%]	1.6% [−0.0–3.3%]	0.1% [−0.0–0.2%]	0.1% [−0.0–0.3%]	0.1% [−0.0–0.3%]	0.1% [0.1–0.2%]	0.2% [0.1–0.3%]	0.1% [0.1–0.2%]	2.1% [−0.0–10.7%]	1.7% [−0.0–8.4%]	2.0% [−0.0–9.8%]	1.1% [0.8–1.3%]	1.6% [1.2–2.0%]	1.3% [1.0–1.6%]
1994	9.2% [2.1–19.1%]	9.7% [2.4–19.1%]	9.4% [2.2–19.4%]	1.2% [0.6–1.8%]	1.0% [0.5–1.5%]	1.1% [0.6–1.7%]	3.0% [−0.7–5.9%]	2.1% [−0.4–4.2%]	2.6% [−0.6–5.3%]	1.4% [0.6–2.1%]	1.7% [0.7–2.6%]	1.5% [0.6–2.3%]	1.0% [0.5–1.5%]	2.2% [0.6–3.6%]	1.5% [0.5–2.4%]	1.2% [−0.0–2.3%]	2.4% [−0.0–4.9%]	1.6% [−0.0–3.3%]	0.1% [−0.0–0.2%]	0.1% [−0.0–0.3%]	0.1% [−0.0–0.3%]	0.1% [0.1–0.2%]	0.2% [0.1–0.3%]	0.1% [0.1–0.2%]	2.1% [−0.0–10.5%]	1.7% [0.0–8.3%]	2.0% [−0.0–9.6%]	1.0% [0.8–1.3%]	1.6% [1.2–2.0%]	1.2% [1.0–1.5%]
1995	9.0% [2.1–18.8%]	9.5% [2.4–18.9%]	9.2% [2.1–19.1%]	1.2% [0.6–1.8%]	1.0% [0.5–1.4%]	1.1% [0.6–1.7%]	2.8% [−0.6–5.7%]	1.9% [−0.4–4.0%]	2.5% [−0.5–5.1%]	1.4% [0.6–2.1%]	1.7% [0.7–2.6%]	1.5% [0.6–2.3%]	1.0% [0.5–1.5%]	2.2% [0.6–3.6%]	1.5% [0.5–2.3%]	1.2% [−0.0–2.3%]	2.4% [−0.0–4.9%]	1.6% [−0.0–3.3%]	0.1% [−0.0–0.2%]	0.1% [−0.0–0.3%]	0.1% [−0.0–0.3%]	0.1% [0.0–0.2%]	0.2% [0.1–0.3%]	0.1% [0.1–0.2%]	2.1% [−0.0–10.3%]	1.7% [0.0–8.2%]	1.9% [−0.0–9.5%]	1.0% [0.7–1.2%]	1.6% [1.2–1.9%]	1.2% [0.9–1.5%]
1996	8.7% [2.1–18.2%]	9.3% [2.4–18.5%]	9.0% [2.1–18.5%]	1.2% [0.6–1.8%]	1.0% [0.5–1.4%]	1.1% [0.6–1.6%]	2.6% [−0.6–5.4%]	1.8% [−0.3–3.8%]	2.3% [−0.5–4.8%]	1.4% [0.6–2.1%]	1.7% [0.7–2.6%]	1.5% [0.6–2.3%]	1.0% [0.5–1.5%]	2.2% [0.6–3.6%]	1.5% [0.5–2.3%]	1.2% [−0.0–2.3%]	2.4% [−0.0–4.9%]	1.6% [−0.0–3.3%]	0.1% [−0.0–0.2%]	0.1% [−0.0–0.3%]	0.1% [−0.0–0.3%]	0.1% [0.0–0.2%]	0.1% [0.1–0.3%]	0.1% [0.1–0.2%]	2.0% [−0.0–10.1%]	1.6% [0.0–8.0%]	1.9% [−0.0–9.3%]	0.9% [0.7–1.2%]	1.5% [1.1–1.9%]	1.2% [0.9–1.4%]
1997	8.5% [2.1–17.7%]	9.2% [2.4–18.2%]	8.8% [2.1–18.1%]	1.2% [0.6–1.7%]	1.0% [0.5–1.4%]	1.1% [0.6–1.6%]	2.5% [−0.5–5.1%]	1.7% [−0.3–3.5%]	2.2% [−0.4–4.5%]	1.4% [0.6–2.1%]	1.7% [0.7–2.5%]	1.5% [0.6–2.3%]	1.0% [0.5–1.5%]	2.2% [0.6–3.6%]	1.5% [0.5–2.3%]	1.2% [−0.0–2.3%]	2.4% [−0.0–4.9%]	1.6% [−0.0–3.3%]	0.1% [−0.0–0.2%]	0.2% [−0.0–0.3%]	0.1% [−0.0–0.3%]	0.1% [0.0–0.2%]	0.1% [0.1–0.3%]	0.1% [0.1–0.2%]	2.0% [−0.0–9.9%]	1.6% [0.0–7.9%]	1.9% [−0.0–9.2%]	0.9% [0.7–1.1%]	1.5% [1.1–1.8%]	1.1% [0.9–1.4%]
1998	8.2% [2.1–17.3%]	9.0% [2.4–17.8%]	8.5% [2.1–17.7%]	1.2% [0.6–1.7%]	0.9% [0.5–1.4%]	1.1% [0.6–1.6%]	2.3% [−0.5–4.8%]	1.5% [−0.3–3.2%]	2.0% [−0.4–4.3%]	1.4% [0.6–2.1%]	1.7% [0.7–2.6%]	1.5% [0.6–2.3%]	1.0% [0.5–1.5%]	2.2% [0.6–3.5%]	1.5% [0.5–2.3%]	1.2% [−0.0–2.4%]	2.4% [−0.0–5.0%]	1.6% [−0.0–3.3%]	0.1% [−0.0–0.2%]	0.2% [−0.0–0.3%]	0.1% [−0.0–0.3%]	0.1% [0.0–0.2%]	0.1% [0.1–0.2%]	0.1% [0.1–0.2%]	2.0% [−0.0–9.8%]	1.6% [0.0–7.8%]	1.8% [−0.0–9.1%]	0.9% [0.7–1.1%]	1.4% [1.1–1.8%]	1.1% [0.8–1.3%]
1999	8.0% [2.1–16.8%]	8.8% [2.4–17.6%]	8.3% [2.1–17.2%]	1.1% [0.6–1.7%]	0.9% [0.5–1.4%]	1.1% [0.6–1.6%]	2.1% [−0.4–4.5%]	1.4% [−0.2–3.0%]	1.8% [−0.4–4.0%]	1.4% [0.6–2.1%]	1.7% [0.7–2.5%]	1.5% [0.6–2.3%]	1.0% [0.5–1.5%]	2.2% [0.6–3.5%]	1.5% [0.5–2.2%]	1.2% [−0.0–2.4%]	2.4% [−0.0–5.0%]	1.6% [−0.0–3.4%]	0.1% [−0.0–0.2%]	0.2% [−0.0–0.3%]	0.1% [−0.0–0.3%]	0.1% [0.0–0.2%]	0.1% [0.1–0.2%]	0.1% [0.0–0.2%]	2.0% [−0.0–9.7%]	1.6% [0.0–7.8%]	1.8% [−0.0–9.1%]	0.9% [0.7–1.1%]	1.4% [1.1–1.7%]	1.1% [0.8–1.3%]
2000	7.8% [2.1–16.5%]	8.6% [2.4–17.3%]	8.1% [2.1–16.9%]	1.1% [0.6–1.6%]	0.9% [0.5–1.4%]	1.0% [0.6–1.5%]	1.9% [−0.4–4.2%]	1.3% [−0.2–2.8%]	1.7% [−0.3–3.7%]	1.4% [0.6–2.1%]	1.7% [0.7–2.5%]	1.5% [0.6–2.3%]	1.0% [0.5–1.5%]	2.2% [0.6–3.5%]	1.5% [0.5–2.3%]	1.2% [−0.0–2.4%]	2.4% [−0.0–4.9%]	1.6% [−0.0–3.3%]	0.1% [−0.0–0.2%]	0.2% [−0.0–0.3%]	0.1% [−0.0–0.3%]	0.1% [0.0–0.2%]	0.1% [0.1–0.2%]	0.1% [0.0–0.2%]	2.0% [−0.0–9.7%]	1.6% [0.0–7.7%]	1.8% [−0.0–9.0%]	0.8% [0.6–1.0%]	1.4% [1.0–1.7%]	1.0% [0.8–1.3%]
2001	7.6% [2.1–16.2%]	8.5% [2.4–17.1%]	7.9% [2.1–16.6%]	1.1% [0.6–1.6%]	0.9% [0.5–1.4%]	1.0% [0.5–1.5%]	1.8% [−0.3–3.9%]	1.2% [−0.2–2.6%]	1.5% [−0.3–3.5%]	1.4% [0.6–2.1%]	1.7% [0.7–2.5%]	1.5% [0.6–2.2%]	1.0% [0.5–1.5%]	2.2% [0.6–3.4%]	1.5% [0.5–2.3%]	1.2% [−0.0–2.4%]	2.4% [−0.0–5.0%]	1.6% [−0.0–3.3%]	0.1% [−0.0–0.3%]	0.2% [−0.0–0.3%]	0.1% [−0.0–0.3%]	0.1% [0.0–0.1%]	0.1% [0.1–0.2%]	0.1% [0.0–0.2%]	2.0% [−0.0–9.7%]	1.6% [0.0–7.7%]	1.8% [−0.0–9.0%]	0.8% [0.6–1.0%]	1.4% [1.0–1.7%]	1.0% [0.8–1.2%]
2002	7.5% [2.1–16.0%]	8.3% [2.4–16.8%]	7.8% [2.2–16.4%]	1.1% [0.6–1.6%]	0.9% [0.5–1.3%]	1.0% [0.5–1.5%]	1.6% [−0.3–3.7%]	1.1% [−0.2–2.4%]	1.4% [−0.2–3.2%]	1.4% [0.6–2.1%]	1.7% [0.7–2.5%]	1.5% [0.6–2.2%]	1.0% [0.5–1.5%]	2.2% [0.6–3.4%]	1.5% [0.5–2.2%]	1.2% [−0.0–2.4%]	2.4% [−0.0–4.9%]	1.6% [−0.0–3.3%]	0.1% [−0.0–0.3%]	0.2% [−0.0–0.3%]	0.1% [−0.0–0.3%]	0.1% [0.0–0.1%]	0.1% [0.1–0.2%]	0.1% [0.0–0.2%]	2.0% [−0.0–9.8%]	1.6% [0.0–7.7%]	1.8% [−0.0–9.1%]	0.8% [0.6–1.0%]	1.3% [1.0–1.7%]	1.0% [0.8–1.2%]
2003	7.4% [2.1–15.9%]	8.2% [2.4–16.6%]	7.6% [2.2–16.2%]	1.1% [0.6–1.5%]	0.9% [0.5–1.3%]	1.0% [0.5–1.4%]	1.5% [−0.3–3.4%]	1.0% [−0.2–2.3%]	1.3% [−0.2–3.0%]	1.4% [0.6–2.2%]	1.7% [0.7–2.5%]	1.5% [0.6–2.2%]	1.1% [0.5–1.5%]	2.2% [0.6–3.4%]	1.5% [0.5–2.2%]	1.2% [−0.0–2.5%]	2.4% [−0.0–5.0%]	1.6% [−0.0–3.3%]	0.1% [−0.0–0.3%]	0.2% [−0.0–0.3%]	0.1% [−0.0–0.3%]	0.1% [0.0–0.1%]	0.1% [0.1–0.2%]	0.1% [0.0–0.2%]	2.0% [−0.0–9.8%]	1.6% [0.0–7.7%]	1.8% [−0.0–9.1%]	0.8% [0.6–1.0%]	1.3% [1.0–1.7%]	1.0% [0.8–1.2%]
2004	7.2% [2.1–15.7%]	8.1% [2.4–16.4%]	7.5% [2.2–16.0%]	1.0% [0.5–1.5%]	0.8% [0.4–1.2%]	0.9% [0.5–1.4%]	1.4% [−0.3–3.2%]	0.9% [−0.1–2.1%]	1.2% [−0.2–2.8%]	1.5% [0.6–2.2%]	1.7% [0.7–2.5%]	1.5% [0.6–2.3%]	1.1% [0.5–1.6%]	2.2% [0.6–3.4%]	1.5% [0.5–2.3%]	1.2% [−0.0–2.5%]	2.4% [−0.0–4.9%]	1.6% [−0.0–3.4%]	0.1% [−0.0–0.3%]	0.2% [−0.0–0.3%]	0.1% [−0.0–0.3%]	0.1% [0.0–0.1%]	0.1% [0.1–0.2%]	0.1% [0.0–0.2%]	2.0% [−0.0–9.8%]	1.6% [0.0–7.7%]	1.8% [−0.0–9.1%]	0.8% [0.6–1.0%]	1.3% [1.0–1.7%]	1.0% [0.8–1.2%]
2005	7.1% [2.1–15.3%]	8.0% [2.4–16.1%]	7.4% [2.2–15.7%]	1.0% [0.5–1.5%]	0.8% [0.4–1.2%]	0.9% [0.5–1.4%]	1.3% [−0.2–3.0%]	0.8% [−0.1–1.9%]	1.1% [−0.2–2.5%]	1.5% [0.6–2.2%]	1.7% [0.7–2.5%]	1.5% [0.6–2.3%]	1.1% [0.5–1.6%]	2.2% [0.6–3.5%]	1.5% [0.5–2.3%]	1.2% [−0.0–2.5%]	2.4% [−0.0–5.0%]	1.7% [−0.0–3.4%]	0.1% [−0.0–0.3%]	0.2% [−0.0–0.4%]	0.1% [−0.0–0.3%]	0.1% [0.0–0.1%]	0.1% [0.1–0.2%]	0.1% [0.0–0.2%]	1.9% [−0.0–9.7%]	1.5% [0.0–7.5%]	1.8% [−0.0–9.0%]	0.8% [0.6–1.0%]	1.3% [1.0–1.6%]	1.0% [0.7–1.2%]
2006	6.9% [2.1–14.9%]	7.8% [2.4–15.7%]	7.2% [2.2–15.2%]	1.0% [0.5–1.4%]	0.8% [0.4–1.2%]	0.9% [0.5–1.3%]	1.1% [−0.2–2.7%]	0.7% [−0.1–1.7%]	1.0% [−0.2–2.3%]	1.5% [0.6–2.3%]	1.7% [0.7–2.5%]	1.6% [0.7–2.3%]	1.1% [0.5–1.6%]	2.2% [0.6–3.5%]	1.5% [0.5–2.3%]	1.3% [−0.0–2.6%]	2.5% [−0.0–5.1%]	1.7% [−0.0–3.5%]	0.1% [−0.0–0.3%]	0.2% [−0.0–0.4%]	0.2% [−0.0–0.3%]	0.1% [0.0–0.1%]	0.1% [0.1–0.2%]	0.1% [0.0–0.2%]	1.9% [−0.0–9.4%]	1.5% [0.0–7.2%]	1.7% [−0.0–8.6%]	0.7% [0.6–1.0%]	1.3% [1.0–1.6%]	0.9% [0.7–1.2%]
2007	6.7% [2.1–14.6%]	7.7% [2.4–15.4%]	7.0% [2.2–14.9%]	0.9% [0.5–1.4%]	0.8% [0.4–1.1%]	0.9% [0.5–1.3%]	1.0% [−0.2–2.4%]	0.6% [−0.1–1.4%]	0.8% [−0.1–2.0%]	1.5% [0.6–2.3%]	1.7% [0.7–2.6%]	1.6% [0.7–2.3%]	1.1% [0.5–1.6%]	2.2% [0.6–3.5%]	1.5% [0.5–2.4%]	1.3% [−0.0–2.6%]	2.5% [−0.0–5.2%]	1.7% [−0.0–3.5%]	0.1% [−0.0–0.3%]	0.2% [−0.0–0.4%]	0.2% [−0.0–0.3%]	0.1% [0.0–0.1%]	0.1% [0.0–0.2%]	0.1% [0.0–0.1%]	1.8% [−0.0–9.1%]	1.4% [0.0–6.9%]	1.7% [−0.0–8.3%]	0.7% [0.6–0.9%]	1.3% [0.9–1.6%]	0.9% [0.7–1.1%]
2008	6.6% [2.1–14.3%]	7.6% [2.4–15.2%]	6.9% [2.2–14.7%]	0.9% [0.5–1.3%]	0.7% [0.4–1.1%]	0.8% [0.4–1.2%]	0.9% [−0.2–2.2%]	0.5% [−0.1–1.3%]	0.8% [−0.1–1.8%]	1.5% [0.7–2.3%]	1.7% [0.7–2.6%]	1.6% [0.7–2.4%]	1.1% [0.5–1.7%]	2.2% [0.6–3.6%]	1.5% [0.5–2.4%]	1.3% [−0.0–2.6%]	2.5% [−0.0–5.2%]	1.7% [−0.0–3.5%]	0.2% [−0.0–0.3%]	0.2% [−0.0–0.4%]	0.2% [−0.0–0.4%]	0.1% [0.0–0.1%]	0.1% [0.0–0.2%]	0.1% [0.0–0.1%]	1.8% [−0.0–8.9%]	1.4% [0.0–6.8%]	1.6% [−0.0–8.2%]	0.7% [0.6–0.9%]	1.3% [0.9–1.6%]	0.9% [0.7–1.1%]
2009	6.5% [2.1–14.1%]	7.5% [2.4–15.0%]	6.8% [2.2–14.5%]	0.9% [0.5–1.3%]	0.7% [0.4–1.1%]	0.8% [0.4–1.2%]	0.8% [−0.1–2.0%]	0.5% [−0.1–1.2%]	0.7% [−0.1–1.7%]	1.6% [0.7–2.4%]	1.7% [0.7–2.6%]	1.6% [0.7–2.4%]	1.2% [0.5–1.7%]	2.2% [0.6–3.6%]	1.6% [0.5–2.4%]	1.3% [−0.0–2.7%]	2.6% [−0.0–5.2%]	1.8% [−0.0–3.6%]	0.2% [−0.0–0.3%]	0.2% [−0.0–0.4%]	0.2% [−0.0–0.4%]	0.1% [0.0–0.1%]	0.1% [0.0–0.2%]	0.1% [0.0–0.1%]	1.7% [−0.0–8.8%]	1.3% [0.0–6.7%]	1.6% [−0.0–8.1%]	0.7% [0.5–0.9%]	1.3% [0.9–1.6%]	0.9% [0.7–1.1%]
2010	6.4% [2.1–13.9%]	7.4% [2.4–14.7%]	6.7% [2.2–14.3%]	0.9% [0.5–1.3%]	0.7% [0.4–1.0%]	0.8% [0.4–1.2%]	0.8% [−0.1–1.9%]	0.4% [−0.1–1.0%]	0.6% [−0.1–1.5%]	1.6% [0.7–2.4%]	1.7% [0.7–2.6%]	1.6% [0.7–2.4%]	1.2% [0.5–1.8%]	2.2% [0.6–3.6%]	1.6% [0.5–2.4%]	1.4% [−0.0–2.8%]	2.6% [−0.0–5.3%]	1.8% [−0.0–3.6%]	0.2% [−0.0–0.4%]	0.2% [−0.0–0.4%]	0.2% [−0.0–0.4%]	0.1% [0.0–0.1%]	0.1% [0.0–0.2%]	0.1% [0.0–0.1%]	1.7% [−0.0–8.6%]	1.3% [0.0–6.5%]	1.6% [−0.0–7.9%]	0.7% [0.5–0.9%]	1.2% [0.9–1.5%]	0.9% [0.7–1.1%]
2011	6.3% [2.1–13.7%]	7.3% [2.4–14.6%]	6.6% [2.2–14.0%]	0.9% [0.4–1.3%]	0.7% [0.4–1.0%]	0.8% [0.4–1.2%]	0.7% [−0.1–1.7%]	0.4% [−0.1–0.9%]	0.5% [−0.1–1.4%]	1.6% [0.7–2.4%]	1.7% [0.7–2.6%]	1.6% [0.7–2.5%]	1.2% [0.5–1.8%]	2.2% [0.6–3.6%]	1.6% [0.5–2.5%]	1.4% [−0.0–2.8%]	2.6% [−0.0–5.3%]	1.8% [−0.0–3.7%]	0.2% [−0.0–0.4%]	0.2% [−0.0–0.5%]	0.2% [−0.0–0.4%]	0.1% [0.0–0.1%]	0.1% [0.0–0.2%]	0.1% [0.0–0.1%]	1.7% [−0.0–8.4%]	1.3% [0.0–6.3%]	1.5% [−0.0–7.6%]	0.7% [0.5–0.8%]	1.2% [0.9–1.5%]	0.8% [0.7–1.1%]
2012	6.2% [2.1–13.5%]	7.2% [2.4–14.4%]	6.5% [2.2–13.7%]	0.8% [0.4–1.3%]	0.7% [0.3–1.0%]	0.8% [0.4–1.1%]	0.6% [−0.1–1.6%]	0.3% [−0.0–0.8%]	0.5% [−0.1–1.3%]	1.6% [0.7–2.5%]	1.7% [0.7–2.6%]	1.7% [0.7–2.5%]	1.2% [0.5–1.8%]	2.3% [0.6–3.6%]	1.6% [0.5–2.5%]	1.4% [−0.0–2.8%]	2.6% [−0.0–5.3%]	1.8% [−0.0–3.7%]	0.2% [−0.0–0.4%]	0.2% [−0.0–0.5%]	0.2% [−0.0–0.4%]	0.1% [0.0–0.1%]	0.1% [0.0–0.2%]	0.1% [0.0–0.1%]	1.6% [−0.0–8.2%]	1.2% [0.0–6.1%]	1.5% [−0.0–7.5%]	0.6% [0.5–0.8%]	1.2% [0.9–1.5%]	0.8% [0.6–1.0%]
2013	6.1% [2.1–13.4%]	7.1% [2.3–14.3%]	6.4% [2.1–13.4%]	0.8% [0.4–1.2%]	0.6% [0.3–1.0%]	0.8% [0.4–1.1%]	0.6% [−0.1–1.4%]	0.3% [−0.0–0.8%]	0.4% [−0.1–1.2%]	1.6% [0.7–2.5%]	1.7% [0.7–2.6%]	1.7% [0.7–2.5%]	1.2% [0.5–1.9%]	2.3% [0.6–3.6%]	1.6% [0.5–2.5%]	1.4% [−0.0–2.9%]	2.6% [−0.0–5.3%]	1.8% [−0.0–3.7%]	0.2% [−0.0–0.4%]	0.2% [−0.1–0.5%]	0.2% [−0.0–0.4%]	0.1% [0.0–0.1%]	0.1% [0.0–0.2%]	0.1% [0.0–0.1%]	1.6% [−0.0–8.0%]	1.2% [0.0–6.0%]	1.5% [−0.0–7.3%]	0.6% [0.5–0.8%]	1.2% [0.9–1.5%]	0.8% [0.6–1.0%]
2014	6.0% [2.1–13.2%]	7.1% [2.3–14.2%]	6.4% [2.1–13.3%]	0.8% [0.4–1.2%]	0.6% [0.3–0.9%]	0.7% [0.4–1.1%]	0.5% [−0.1–1.3%]	0.3% [−0.0–0.7%]	0.4% [−0.1–1.1%]	1.7% [0.7–2.5%]	1.7% [0.7–2.6%]	1.7% [0.7–2.5%]	1.3% [0.5–1.9%]	2.3% [0.6–3.6%]	1.6% [0.5–2.5%]	1.4% [−0.0–2.9%]	2.6% [−0.0–5.3%]	1.8% [−0.0–3.7%]	0.2% [−0.0–0.4%]	0.2% [−0.1–0.5%]	0.2% [−0.0–0.4%]	0.1% [0.0–0.1%]	0.1% [0.0–0.1%]	0.1% [0.0–0.1%]	1.6% [−0.0–7.9%]	1.2% [0.0–5.9%]	1.4% [−0.0–7.2%]	0.6% [0.4–0.8%]	1.2% [0.9–1.5%]	0.8% [0.6–1.0%]
2015	6.0% [2.1–12.9%]	7.0% [2.3–14.0%]	6.3% [2.1–13.2%]	0.8% [0.4–1.2%]	0.6% [0.3–0.9%]	0.7% [0.4–1.1%]	0.5% [−0.1–1.2%]	0.2% [−0.0–0.7%]	0.4% [−0.1–1.0%]	1.7% [0.7–2.5%]	1.8% [0.7–2.6%]	1.7% [0.7–2.5%]	1.3% [0.5–1.9%]	2.3% [0.6–3.7%]	1.6% [0.6–2.5%]	1.5% [−0.0–2.9%]	2.7% [−0.0–5.3%]	1.9% [−0.0–3.7%]	0.2% [−0.0–0.4%]	0.2% [−0.1–0.5%]	0.2% [−0.0–0.4%]	0.1% [0.0–0.1%]	0.1% [0.0–0.1%]	0.1% [0.0–0.1%]	1.5% [−0.0–7.7%]	1.2% [0.0–5.7%]	1.4% [−0.0–6.9%]	0.6% [0.4–0.8%]	1.2% [0.8–1.5%]	0.8% [0.6–1.0%]
2016	5.9% [2.1–12.7%]	7.0% [2.3–13.9%]	6.3% [2.1–13.1%]	0.8% [0.4–1.2%]	0.6% [0.3–0.9%]	0.7% [0.4–1.1%]	0.4% [−0.1–1.1%]	0.2% [−0.0–0.6%]	0.4% [−0.1–0.9%]	1.7% [0.7–2.6%]	1.8% [0.7–2.7%]	1.7% [0.7–2.6%]	1.3% [0.5–1.9%]	2.3% [0.6–3.7%]	1.6% [0.6–2.5%]	1.5% [−0.0–3.0%]	2.7% [−0.0–5.4%]	1.9% [−0.0–3.8%]	0.2% [−0.0–0.4%]	0.2% [−0.1–0.5%]	0.2% [−0.0–0.5%]	0.1% [0.0–0.1%]	0.1% [0.0–0.1%]	0.1% [0.0–0.1%]	1.5% [−0.0–7.5%]	1.1% [0.0–5.7%]	1.4% [−0.0–6.8%]	0.6% [0.4–0.7%]	1.2% [0.8–1.5%]	0.8% [0.6–1.0%]
2017	5.9% [2.1–12.7%]	7.0% [2.3–13.8%]	6.3% [2.1–13.0%]	0.8% [0.4–1.1%]	0.6% [0.3–0.9%]	0.7% [0.4–1.0%]	0.4% [−0.1–1.1%]	0.2% [−0.0–0.6%]	0.3% [−0.1–0.9%]	1.7% [0.7–2.6%]	1.8% [0.7–2.7%]	1.7% [0.7–2.6%]	1.3% [0.6–2.0%]	2.3% [0.7–3.8%]	1.7% [0.6–2.6%]	1.5% [−0.0–3.0%]	2.7% [−0.0–5.5%]	1.9% [−0.0–3.8%]	0.2% [−0.0–0.4%]	0.2% [−0.1–0.5%]	0.2% [−0.0–0.5%]	0.1% [0.0–0.1%]	0.1% [0.0–0.1%]	0.1% [0.0–0.1%]	1.5% [−0.0–7.4%]	1.1% [0.0–5.5%]	1.4% [−0.0–6.7%]	0.5% [0.4–0.7%]	1.1% [0.8–1.4%]	0.8% [0.6–1.0%]
2018	5.9% [2.1–12.8%]	7.0% [2.3–13.8%]	6.3% [2.1–12.9%]	0.8% [0.4–1.1%]	0.6% [0.3–0.9%]	0.7% [0.4–1.0%]	0.4% [−0.1–1.1%]	0.2% [−0.0–0.6%]	0.3% [−0.1–0.9%]	1.8% [0.7–2.6%]	1.8% [0.7–2.7%]	1.8% [0.7–2.6%]	1.3% [0.6–2.0%]	2.3% [0.6–3.8%]	1.7% [0.6–2.6%]	1.5% [−0.0–3.1%]	2.7% [−0.0–5.5%]	1.9% [−0.0–3.9%]	0.2% [−0.0–0.4%]	0.3% [−0.1–0.5%]	0.2% [−0.0–0.5%]	0.1% [0.0–0.1%]	0.1% [0.0–0.1%]	0.1% [0.0–0.1%]	1.5% [−0.0–7.2%]	1.1% [0.0–5.4%]	1.3% [−0.0–6.6%]	0.5% [0.4–0.7%]	1.1% [0.8–1.5%]	0.7% [0.6–0.9%]
2019	5.9% [2.1–12.6%]	7.0% [2.3–13.7%]	6.3% [2.1–12.8%]	0.7% [0.4–1.1%]	0.6% [0.3–0.9%]	0.7% [0.4–1.0%]	0.4% [−0.1–1.0%]	0.2% [−0.0–0.5%]	0.3% [−0.1–0.8%]	1.8% [0.7–2.6%]	1.8% [0.7–2.7%]	1.8% [0.7–2.6%]	1.4% [0.6–2.0%]	2.3% [0.6–3.8%]	1.7% [0.6–2.6%]	1.6% [−0.0–3.1%]	2.7% [−0.0–5.6%]	2.0% [−0.0–4.0%]	0.2% [−0.0–0.4%]	0.3% [−0.1–0.6%]	0.2% [−0.0–0.5%]	0.1% [0.0–0.1%]	0.1% [0.0–0.1%]	0.1% [0.0–0.1%]	1.4% [−0.0–7.1%]	1.1% [0.0–5.3%]	1.3% [−0.0–6.5%]	0.5% [0.4–0.7%]	1.1% [0.8–1.5%]	0.7% [0.6–0.9%]
2020	5.9% [2.1–12.6%]	7.0% [2.3–13.6%]	6.3% [2.1–12.9%]	0.7% [0.4–1.1%]	0.6% [0.3–0.9%]	0.7% [0.4–1.0%]	0.4% [−0.1–1.0%]	0.2% [−0.0–0.5%]	0.3% [−0.1–0.8%]	1.8% [0.8–2.7%]	1.8% [0.7–2.8%]	1.8% [0.7–2.7%]	1.4% [0.6–2.1%]	2.3% [0.6–3.9%]	1.7% [0.6–2.7%]	1.6% [−0.0–3.1%]	2.8% [−0.0–5.7%]	2.0% [−0.0–4.0%]	0.2% [−0.0–0.5%]	0.3% [−0.1–0.6%]	0.2% [−0.0–0.5%]	0.1% [0.0–0.1%]	0.1% [0.0–0.1%]	0.1% [0.0–0.1%]	1.4% [−0.0–7.0%]	1.1% [0.0–5.3%]	1.3% [−0.0–6.4%]	0.5% [0.4–0.7%]	1.1% [0.8–1.5%]	0.7% [0.5–0.9%]
2021	5.9% [2.1–12.6%]	7.0% [2.3–13.5%]	6.3% [2.1–12.8%]	0.7% [0.4–1.1%]	0.6% [0.3–0.9%]	0.7% [0.4–1.0%]	0.4% [−0.1–1.1%]	0.2% [−0.0–0.5%]	0.3% [−0.1–0.8%]	1.8% [0.8–2.7%]	1.8% [0.7–2.8%]	1.8% [0.7–2.7%]	1.4% [0.6–2.1%]	2.3% [0.6–3.9%]	1.7% [0.6–2.7%]	1.6% [−0.0–3.1%]	2.8% [−0.0–5.7%]	2.0% [−0.0–4.0%]	0.2% [−0.0–0.5%]	0.3% [−0.1–0.6%]	0.2% [−0.0–0.5%]	0.1% [0.0–0.1%]	0.1% [0.0–0.1%]	0.1% [0.0–0.1%]	1.4% [−0.0–7.0%]	1.1% [0.0–5.2%]	1.3% [−0.0–6.3%]	0.5% [0.4–0.7%]	1.1% [0.8–1.5%]	0.7% [0.5–1.0%]

Data are presented as percentages (%) with 95% confidence intervals [lower-upper]. Values of “−0.0%” and “0.0%” result from rounding to one decimal place. “−0.0%” indicates that the original value was negative but rounded to zero (absolute value <0.05%), while “0.0%” indicates a positive value that rounded to zero (<0.05%). Negative values suggest an inverse relationship between the dietary factor and cancer mortality, while positive values indicate a positive association. All rates are age-standardized.

By 2021, China’s dietary attribution (6.3%) positioned intermediately between high-SDI territories (5.8%) and middle-SDI populations (7.4%), while remaining below global averages (6.8%) (Appendix 1).

### Spatiotemporal characteristics and temporal trends of dietary risk factors

**Figure 1:**
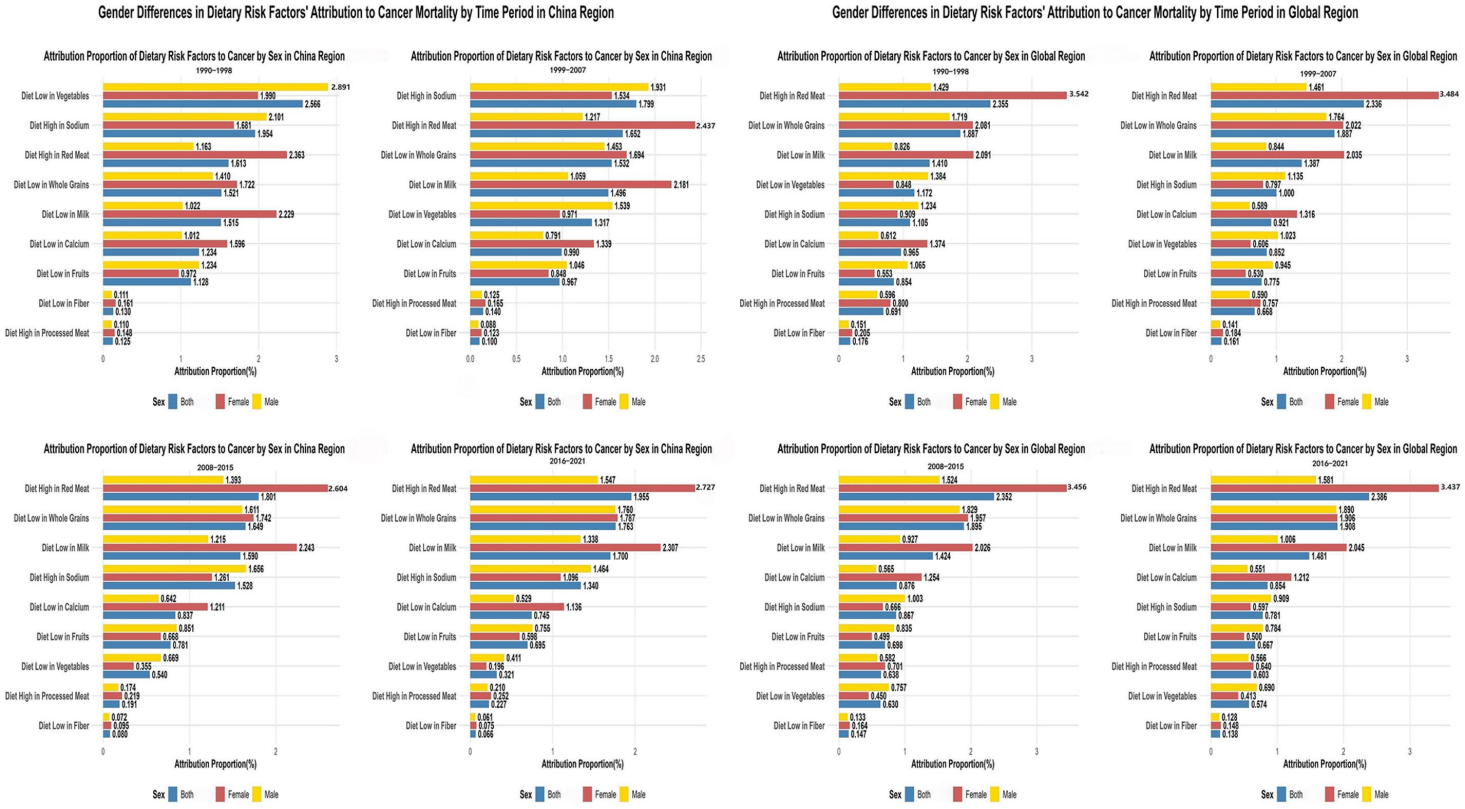
Comparative analysis of dietary risk factors’ attribution to cancer types in china and global regions with temporal patterns (1990–2021). The top panels show attribution proportions of nine dietary risk factors to different cancer types in China (left) and globally (right). Colors represent cancer types. Bottom panels display heatmaps illustrating temporal changes in attribution proportions across four time periods, revealing China’s transition from low vegetable intake dominance toward high red meat consumption, while globally, high red meat intake maintained the highest attribution proportion throughout the study period.

Primary dietary risk determinants comprised high red meat consumption (1.735%), elevated sodium intake (1.689%), low whole grain consumption (1.601%), and insufficient milk intake (1.563%) (Figure 1; Appendix 2). These factors exhibited distinct cancer-specific impacts: red meat consumption predominantly affected colorectal cancer (15.245%) and breast cancer (13.580%); whole grain inadequacy primarily influenced colorectal cancer (18.112%); milk consumption deficiency impacted colorectal cancer (18.827%) while demonstrating protective effects against prostate cancer (−7.980%); elevated sodium intake principally affected gastric cancer (8.305%); and vegetable deficiency showed marked influence on esophageal cancer (9.810%).

Temporal ranking analyses demonstrated China’s dietary risk hierarchy transformation from vegetable inadequacy dominance (1st position during 1990–1998, 2.566%) toward red meat consumption predominance (1st position during 2016–2021, 1.955%). Globally, high red meat intake consistently maintained the 1st position (from 2.355 to 2.386%), while low vegetable intake dropped from 4th position (1.172%) to 8th position (0.574%) (Appendix 2). SDI-stratified analysis (Supplementary Figure S1; Appendix 2) demonstrated that dietary risk factors globally are transitioning from traditionally predominant low vegetable intake toward modern patterns dominated by high red meat consumption.

**Figure 2:**
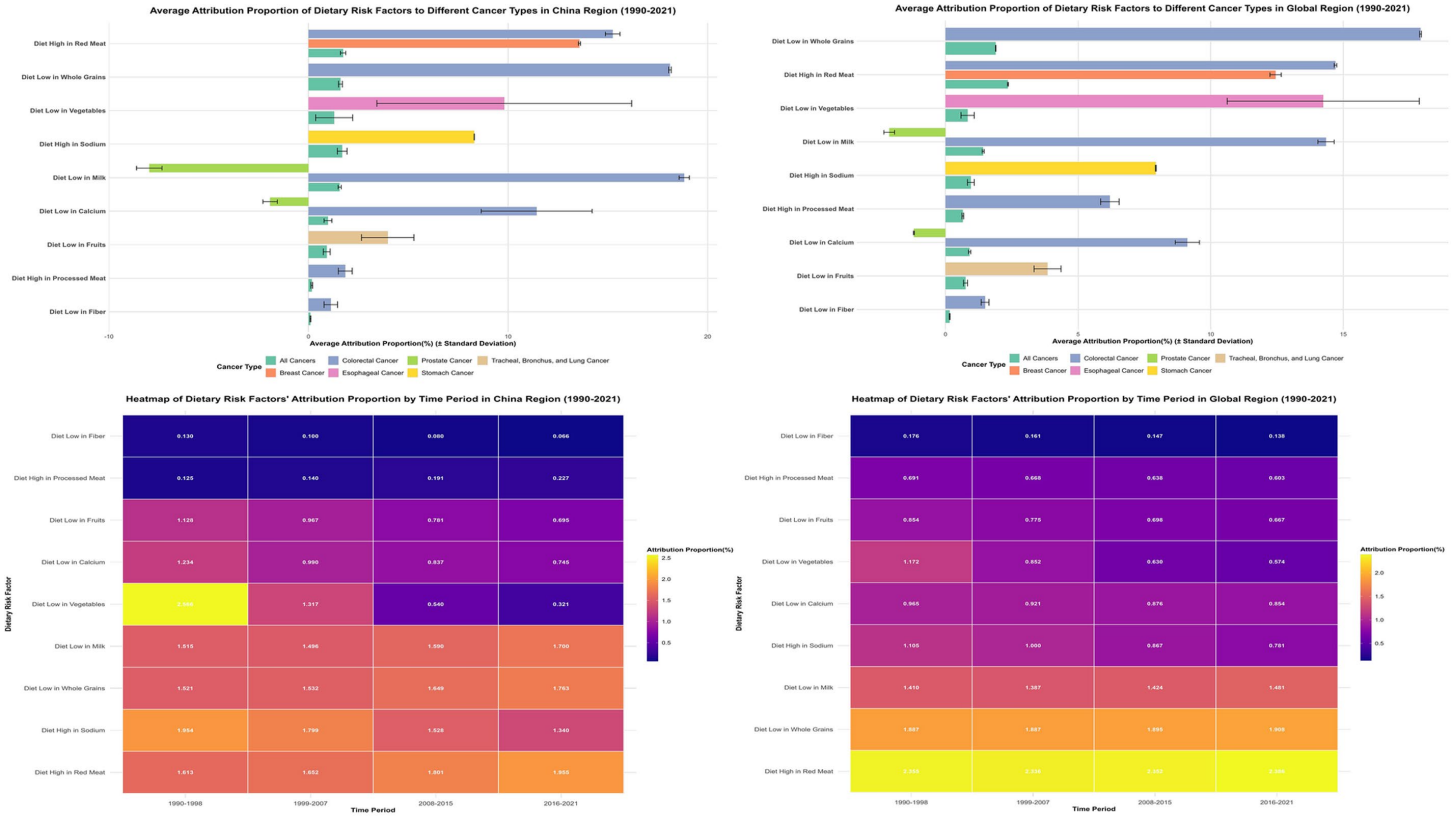
Joinpoint Regression Analysis of Dietary Risk Factors’ Impact on Cancer Mortality in China and Global Regions (1990–2021). Left panels show joinpoint regression analyses of age-standardized cancer mortality rates attributable to nine dietary risk factors in China, with significant joinpoints marked by vertical lines. Right panels display corresponding global trends. Tables below each graph present Annual Percent Change (APC) values for identified segments. APC represents the percentage change per year within each segment, while AAPC (Average Annual Percent Change) indicates the overall average annual percentage change for the entire period. Side panels compare overall temporal trends between China and global regions.

Joinpoint regression analyses revealed China’s overall dietary-attributable cancer mortality rates declined from 18.441/100,000 to 8.662/100,000, representing a 53.03% reduction substantially exceeding global patterns (35.45%) (Figure 2; Appendix 3). Vegetable inadequacy demonstrated the most pronounced improvement with AAPC of −14.86% (95% CI: −15.60% to −14.12%), declining from 5.496/100,000 to 0.434/100,000. However, red meat consumption remained relatively static [AAPC: −0.16% (95% CI: −0.70 to 0.39%)], while processed meat consumption constituted the sole factor exhibiting increasing trajectories [AAPC: 0.30% (95% CI: 0.17 to 0.44%)].

SDI-stratified joinpoint analysis (Supplementary Figure S2; Appendix 3) revealed differentiated patterns: high-SDI regions demonstrated AAPC of −14.63% (95% CI: −17.12% to −12.07%); middle-SDI regions exhibited AAPC of −13.01% (95% CI: −15.34% to −10.62%); while low-middle SDI regions showed minimal change with AAPC of −0.15% (95% CI: −0.98 to 0.69%).

### Gender disparities in dietary risk attribution

**Figure 3:**
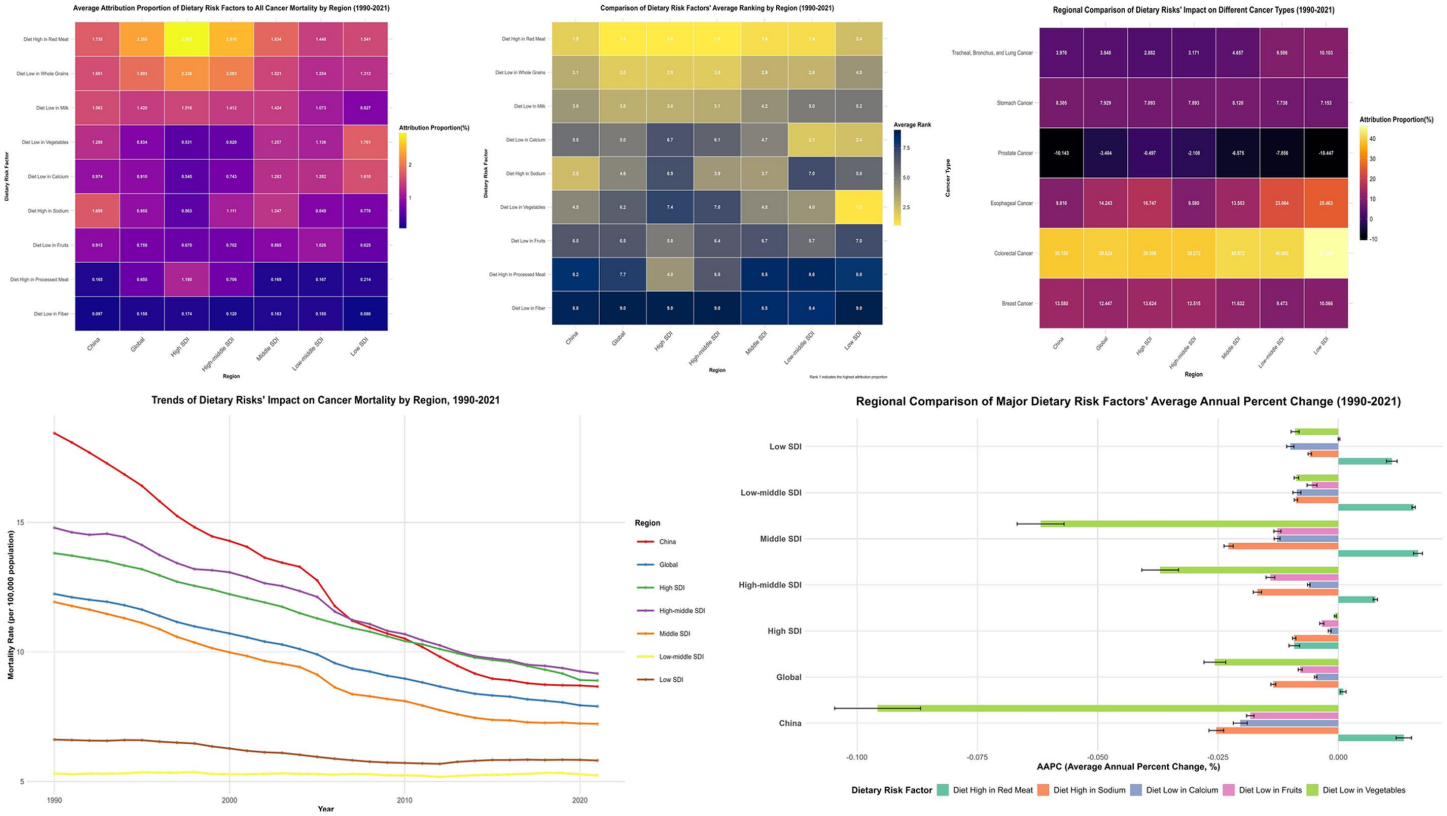
Gender Differences in Dietary Risk Factors’ Attribution to Cancer Mortality Across Different Time Periods in China vs. Global Regions (1990–2021). The left column shows data for China across four time periods (1990–1998, 1999–2007, 2008–2015, 2016–2021), while the right column displays corresponding global data for the same time periods. In each chart, dietary risk factors are listed on the y-axis, with attribution proportion (%) on the x-axis.

Significant gender-specific patterns emerged across the observation period (Figure 3). In China during 1990–1998, low vegetable intake’s attribution among males (2.891% ± 0.121%) significantly exceeded females (1.990% ± 0.092%), with male-to-female ratio of 1.45. Conversely, high red meat intake demonstrated opposite pattern, with females (2.363% ± 0.014%) substantially exceeding males (1.163% ± 0.005%), yielding male-to-female ratio of 0.49 (Appendix 4; Figure 3).

By 2016–2021, high red meat intake’s attribution among Chinese females (2.727% ± 0.017%) continued exceeding males (1.547% ± 0.016%), with male-to-female ratio of 0.57. Low vegetable intake’s impact remained higher among males (0.411% ± 0.009%) vs. females (0.196% ± 0.006%), with male-to-female ratio increasing to 2.10. Global demographic patterns paralleled Chinese observations, with female populations exhibiting higher red meat attribution (3.437% ± 0.001% vs. 1.581% ± 0.004% for males, ratio: 0.46) (Figure 3; Supplementary Figure S3).

### Mineral and dairy-related dietary risk factors

**Figure 4:**
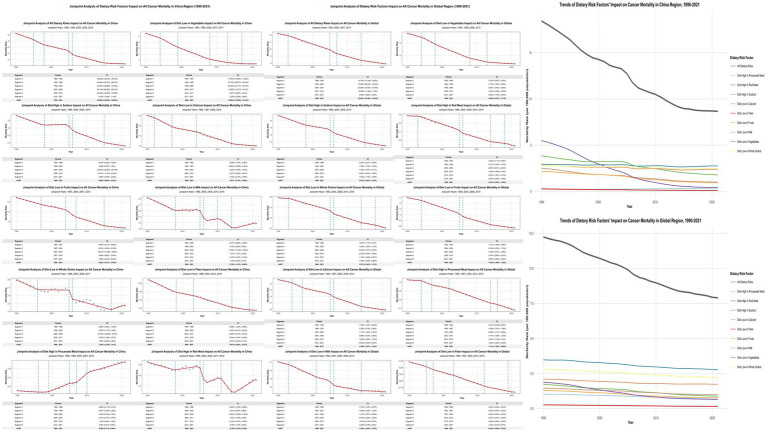
Multidimensional Comparative Analysis of Dietary Risk Factors’ Impact on Cancer Mortality Across Regions with Different Socioeconomic Development Levels (1990–2021). This figure presents a comprehensive six-panel visualization of dietary risk factors’ impact on cancer mortality across different regions and cancer types. Top Row (Three Heatmaps): Left panel: Average attribution proportion (%) of nine dietary risk factors to all cancer mortality by region. Middle panel: Comparative ranking of dietary risk factors across regions, with darker blue indicating higher ranking positions. Right panel: Regional comparison of dietary risks’ impact on different cancer types. Bottom Row (Two Graphs): Left panel: Temporal trends of age-standardized cancer mortality rates (per 100,000) attributable to all dietary risk factors from 1990 to 2021. Right panel: Regional comparison of major dietary risk factors’ average annual percent change (AAPC).

Mineral-associated and dairy-derived nutritional inadequacies demonstrated pronounced cancer-type specificity alongside distinctive regional distribution architectures. Elevated sodium consumption constituted China’s second-ranking dietary risk determinant (1.689%), manifesting predominant gastric malignancy associations with attribution proportions reaching 8.305% (Figure 1, 4; Appendix 2). Inadequate calcium consumption contributed 0.974% to aggregate attribution, whereas insufficient dairy product intake represented the fourth-ranking risk determinant (1.563%), manifesting maximal colorectal cancer impact (18.827%) while demonstrating protective associations against prostate malignancy (−7.980%) (Figure 1, 4; Appendix 2).

Cross-regional comparative analyses unveiled complex non-linear distribution patterns. Elevated sodium consumption exhibited distinctive inverted-U relationships, peaking in middle-SDI territories (1.347%), followed by high-middle SDI regions (1.111%), with diminished impacts in high-SDI (0.563%) and low-SDI areas (0.778%). Calcium deficiency demonstrated ascending patterns with developmental disadvantage, reaching maximum attribution in low-SDI regions (1.610%) and minimum impact in high-SDI areas (0.545%) (Supplementary Figure S1; Appendix 2).

### Cancer-specific impact and regional attribution changes

Dietary risk factors’ impact on colorectal cancer was most pronounced across all regions, with China at 39.158% ± 0.248%, representing the lowest attribution among compared regions—below the global average (39.824% ± 0.099%), middle-SDI regions (40.972% ± 0.249%), and high-middle SDI regions (39.272% ± 0.126%). For breast cancer impact, China (13.580% ± 0.009%) exceeded global average (12.447% ± 0.038%) (Figure 4; Appendix 5).

Notably, dietary risk factors’ impact on esophageal cancer in China (9.810% ± 1.129%) was significantly below global average (14.243% ± 0.640%). The impact on prostate cancer was negative across all regions, with China’s negative effect (−10.143% ± 0.188%) stronger than global level (−3.404% ± 0.038%) (Figure 4; Appendix 5).

China’s attribution proportion for low vegetable intake demonstrated the most substantial decline, decreasing from 2.956% in 1990 to 0.315% in 2021—an 89.36% reduction significantly exceeding global average (55.11%) (Appendix 6). In contrast, China demonstrated increasing trends in red meat consumption, with high red meat intake increasing from 1.575 to 2.008% (27.46% increase), while processed meat intake surged by 91.38% (from 0.122 to 0.234%) (Appendix 6).

## Discussion

Using GBD 2021 data, we examined dietary risk factor burden on cancer mortality in China vs. global regions stratified by socioeconomic development from 1990 to 2021. China showed a decrease in dietary-attributable cancer deaths from 9.9 to 6.3%, placing it between high-SDI regions (5.8%) and middle-SDI regions (7.4%). This position reflects China’s ongoing nutrition transition during rapid economic development. Previous GBD studies identified dietary factors as major modifiable contributors to global disease burden, with impacts varying significantly across development levels (3, 16).

Low and low-middle SDI regions exhibit dietary profiles characterized by inadequate plant food intake. Vegetable insufficiency ranks as the primary dietary risk across these areas, with attribution rates far exceeding those in economically developed regions. This reflects limited food access and economic constraints affecting protective food availability rather than overconsumption of harmful foods.

China demonstrated remarkable improvement, with a 53.0% reduction in dietary risk-attributable cancer mortality vs. 35.5% globally. Vegetable intake adequacy showed the most dramatic improvement, dropping from 3.0 to 0.3% attribution. However, this occurred alongside emerging modern dietary risks. Red meat consumption became China’s leading dietary risk factor, increasing from 1.6 to 2.0%—opposite to declining trends in high-SDI countries. Wang et al. documented similar increases in Chinese meat consumption with urbanization and rising incomes (3, 16, 33). This trend raises concern since IARC classified red meat as a Group 2A carcinogen (7, 17).

High-SDI regions show dietary profiles where animal-based and processed food overconsumption drives primary health risks. Red meat intake remains the top risk factor (2.955% mean attribution), followed by processed meat consumption (1.180% mean attribution). Unlike transitional countries, these populations resolved basic plant food deficiencies but face challenges from dietary excess.

Regional differences in plant food adequacy follow a clear economic development pattern. Developed countries essentially eliminated traditional plant food deficiencies, while China shows substantial progress. This demonstrates how economic growth improves food access and dietary variety. China’s vegetable intake improvement illustrates development-driven nutrition gains, contrasting with persistent deficiencies in lower-income regions.

We observed distinct animal food consumption patterns across development stages. High-SDI regions show stable or declining red meat intake, while China exhibits rapid increases. This represents compressed nutrition transition where dietary westernization occurs faster than historically seen in developed nations. Such rapid change presents intervention opportunities before harmful patterns become established. Regional processed food consumption differences reflect food system modernization stages. Developed regions show established but stable processed meat consumption, while China has lower current levels but concerning growth trajectories. This gap suggests intervention windows exist in transitional countries like China to prevent high-risk dietary behaviors seen in fully developed regions.

We found notable sex differences across dietary categories. Red meat consumption showed greater impact on females (2.7% vs. 1.5% in males), while vegetable inadequacy more severely affected males (0.4% vs. 0.2% in females). Baker and Wardle attributed males’ lower fruit and vegetable consumption to inadequate knowledge about healthy eating practices (18). These patterns suggest dietary transitions affect sexes differently, requiring sex-specific interventions addressing traditional deficiency risks (affecting males more) and modern excess risks (affecting females more).

Dietary factors substantially impact colorectal cancer mortality (39.2% in China), consistent with established links between red meat consumption and fiber deficiency in colorectal carcinogenesis. Meta-analyses show each 100 g/day red meat increase correlates with ~12% higher colorectal cancer risk (19). Red meat’s heme iron catalyzes lipid peroxidation and carcinogen formation, causing intestinal DNA damage (20), while low whole grain and fiber intake reduces intestinal protection (21). This exemplifies how shifting from traditional high-fiber, low-meat to modern low-fiber, high-meat diets directly increases disease burden.

Vegetable insufficiency affects esophageal cancer (9.8% in China), demonstrating continued importance of traditional protective dietary factors. De Stefani et al. found inadequate vegetable and fruit intake correlated with higher esophageal squamous carcinoma risk, attributing protection to comprehensive dietary antioxidants (22). Liu et al.’s meta-analysis confirmed high vegetable and fruit intake significantly reduced esophageal squamous carcinoma risk through antioxidants (vitamin C, lycopene) and folate mechanisms including reduced DNA oxidative damage, enhanced repair, and methylation regulation (23).

High sodium intake affects gastric cancer (8.3% in China), reflecting both traditional food preservation and modern processed food consumption. High-sodium diets may increase chronic gastric mucosal damage and atrophy, raising gastric cancer incidence (24). This shows how certain dietary risks persist across transition stages, requiring sustained attention regardless of development level.

Low calcium and milk intake demonstrated protective effects against prostate cancer, with China showing stronger protective associations than observed globally, suggesting potential benefits of this dietary pattern. This aligns with Aune et al.’s findings linking higher calcium intake to increased prostate cancer risk (24, 34). Such relationships indicate optimal dietary patterns may vary by cancer type, with transitional diets potentially offering unexpected protection for specific malignancies.

Our temporal and regional analysis reveals global shift from traditional vegetable deficiency to red meat excess, with China exemplifying this change through dramatically improved vegetable adequacy alongside increased red meat consumption. This supports Popkin’s nutrition transition theory describing dietary changes with economic development (25). China experiences accelerated transition from traditional to westernized diets, marked by decreased grain consumption and increased animal and processed food intake (26). Our findings suggest this creates both opportunities (traditional risk reduction) and challenges (modern risk emergence) requiring balanced policies.

### Clinical and public health implications

China’s epidemiological transformation—dietary attribution declining from 9.9% (95% CI: 2.2–20.5%) to 6.3% (95% CI: 2.1–12.8%)—necessitates intervention architectures reinforcing protective accomplishments while forestalling emergent risks. Such transitional dynamics demand region-specific methodologies: consolidating phytochemical improvements while circumventing escalating animal protein consumption trajectories.

Colorectal malignancies demonstrate substantial dietary attribution (39.2%), mediated through red meat consumption (15.245%) and whole grain deficiency (18.112%). However, optimal red meat parameters remain epidemiologically contested—while dose–response modeling suggests theoretical risk minimization at negligible intake (0 g/daily), considerable uncertainty intervals (0–200 g/daily) obviate categorical safety thresholds, mandating individualized assessment protocols rather than population-wide prohibitions (15).

Gastric carcinogenesis mitigation necessitates sodium restriction given our quantified 8.305% attributable burden, whereas esophageal malignancy prevention requires sustained vegetable vigilance (9.810% attribution when inadequate), despite China’s transformation from 3.0 to 0.3% attribution.

China’s intermediate positioning (6.3%)—between high-SDI territories (5.8%) and middle-SDI populations (7.4%)—suggests dual-pronged approaches: fortifying established protective transformations while preempting Western consumption paradigm assimilation. Gender-stratified protocols emerge from demographic heterogeneity, whereby red meat manifests disproportionate female vulnerability while plant-based inadequacy predominantly afflicts males.

Implementation architectures encompass transitional nutrition monitoring, graduated dietary guidelines reflecting developmental contexts, plus anticipatory surveillance systems. The documented 87% vegetable inadequacy reduction validates population-scale modification feasibility, furnishing empirical substantiation for comprehensive strategies addressing contemporary challenges while preserving traditional protective mechanisms.

### Study strengths and limitations

This study provides methodological advantages through long-term analysis (1990–2021) and precise trend identification using joinpoint regression, enabling clear dietary pattern transition documentation across development levels. Limitations include ecological design preventing individual-level causal inference, lack of sub-national Chinese data revealing internal dietary variations, and insufficient examination of dietary-lifestyle interactions potentially modifying observed relationships.

Future studies should examine provincial variations within China to understand local dietary transition differences, investigate molecular mechanisms through biomarker studies clarifying biological pathways linking dietary patterns to cancer risks, conduct prospective studies examining dietary modification impacts on cancer outcomes in transitional populations, and assess synergistic effects between dietary and lifestyle factors particularly relevant during nutrition transitions.

## Conclusion

China’s dietary-attributable cancer mortality decreased from 9.9 to 6.3% (1990–2021), showing reduced vegetable deficiency but increased red meat consumption, positioning between high-SDI and middle-SDI regions. This exemplifies global evolution from traditional deficiency-based to modern excess-based dietary patterns. Sex-specific patterns emerged, with red meat consumption affecting females more while inadequate plant consumption impacted males more. Findings suggest targeted prevention strategies accounting for dietary pattern stages, preserving traditional protective factors while preventing modern dietary risks based on regional development contexts and sex-specific behaviors.

## Data Availability

The original contributions presented in the study are included in the article/Supplementary material, further inquiries can be directed to the corresponding author.
