# Supplementary material for: Dietary risk factors and cancer mortality burden from 1990 to 2021: a comparative study of China and global regions with varying sociodemographic development levels based on the Global Burden of Disease database
Source: Front Nutr. 2025 Aug 5;12:1628792. doi: 10.3389/fnut.2025.1628792 (PMC12360945; doi:10.3389/fnut.2025.1628792)
Supplement: Supplementary file 1 [file Data_Sheet_1.docx]

**Appendixes**

1. **Appendixes 1: Table S1a - Table S1f**

**2. Appendixes 2: Table S2a - Table S2n**

**3. Appendixes 3: Table S3a - Table S3g**

**4. Appendixes 4: Table S4a - Table S4ab**

**5. Appendixes 5: Table S5a - Table S5e**

**6. Appendixes 6: Table S6a - Table S6f**

**7. Appendixes 7: Figure S1 - Figure S3**

**Appendixes 1**

| Table S1a. Percentage of Total Cancer Mortality Attributable to Dietary Risk Factors - Global (Age-standardized, 1990-2021). | | | | | | | | | | | | | | | | | | | | | | | | | | | | | | |
| --- | --- | --- | --- | --- | --- | --- | --- | --- | --- | --- | --- | --- | --- | --- | --- | --- | --- | --- | --- | --- | --- | --- | --- | --- | --- | --- | --- | --- | --- | --- |
| Year | All dietary risks | | | Low in fruits | | | Low in vegetables | | | Low in whole grains | | | Low in milk | | | High in red meat | | | High in processed meat | | | Low in fiber | | | High in sodium | | | Low in calcium | | |
|  | Male | Female | Both | Male | Female | Both | Male | Female | Both | Male | Female | Both | Male | Female | Both | Male | Female | Both | Male | Female | Both | Male | Female | Both | Male | Female | Both | Male | Female | Both |
| 1990 | 7.4% [2.2%-14.5%] | 9.2% [2.5%-17.0%] | 8.3% [2.3%-15.7%] | 1.1% [0.6%-1.6%] | 0.6% [0.3%-0.8%] | 0.9% [0.5%-1.3%] | 1.5% [-0.3%-3.0%] | 0.9% [-0.2%-1.9%] | 1.3% [-0.3%-2.5%] | 1.7% [0.7%-2.6%] | 2.1% [0.9%-3.1%Z] | 1.9% [0.8%-2.8%] | 0.8% [0.4%-1.3%] | 2.1% [0.6%-3.5%] | 1.4% [0.5%-2.3%] | 1.4% [-0.0%-2.9%] | 3.5% [-0.0%-7.3%] | 2.4% [-0.0%-4.8%] | 0.6% [-0.1%-1.2%] | 0.8% [-0.2%-1.6%] | 0.7% [-0.2%-1.4%] | 0.2% [0.1%-0.2%] | 0.2% [0.1%-0.3%] | 0.2% [0.1%-0.3%] | 1.3% [-0.0%-6.6%] | 1.0% [0.0%-5.0%] | 1.2% [-0.0%-5.9%] | 0.6% [0.5%-0.8%] | 1.4% [1.0%-1.8%] | 1.0% [0.8%-1.2%] |
| 1991 | 7.3% [2.2%-14.4%] | 9.2% [2.5%-16.8%] | 8.2% [2.3%-15.6%] | 1.1% [0.6%-1.6%] | 0.6% [0.3%-0.8%] | 0.9% [0.5%-1.3%] | 1.5% [-0.3%-2.9%] | 0.9% [-0.2%-1.8%] | 1.3% [-0.3%-2.5%] | 1.7% [0.7%-2.6%] | 2.1% [0.9%-3.1%] | 1.9% [0.8%-2.8%] | 0.8% [0.4%-1.3%] | 2.1% [0.6%-3.4%] | 1.4% [0.5%-2.3%] | 1.4% [-0.0%-2.9%] | 3.5% [-0.0%-7.3%] | 2.4% [-0.0%-4.8%] | 0.6% [-0.1%-1.2%] | 0.8% [-0.2%-1.6%] | 0.7% [-0.2%-1.4%] | 0.2% [0.1%-0.2%] | 0.2% [0.1%-0.3%] | 0.2% [0.1%-0.3%] | 1.3% [-0.0%-6.5%] | 1.0% [0.0%-4.9%] | 1.2% [-0.0%-5.9%] | 0.6% [0.5%-0.8%] | 1.4% [1.0%-1.7%] | 1.0% [0.8%-1.2%] |
| 1992 | 7.3% [2.1%-14.2%] | 9.1% [2.5%-16.6%] | 8.2% [2.3%-15.4%] | 1.1% [0.6%-1.6%] | 0.6% [0.3%-0.8%] | 0.9% [0.5%-1.3%] | 1.5% [-0.3%-2.9%] | 0.9% [-0.2%-1.8%] | 1.2% [-0.3%-2.4%] | 1.7% [0.7%-2.6%] | 2.1% [0.9%-3.1%] | 1.9% [0.8%-2.8%] | 0.8% [0.4%-1.3%] | 2.1% [0.6%-3.4%] | 1.4% [0.5%-2.3%] | 1.4% [-0.0%-2.9%] | 3.6% [-0.0%-7.3%] | 2.4% [-0.0%-4.8%] | 0.6% [-0.1%-1.2%] | 0.8% [-0.2%-1.6%] | 0.7% [-0.2%-1.4%] | 0.2% [0.1%-0.2%] | 0.2% [0.1%-0.3%] | 0.2% [0.1%-0.3%] | 1.3% [-0.0%-6.4%] | 0.9% [0.0%-4.8%] | 1.1% [-0.0%-5.8%] | 0.6% [0.5%-0.8%] | 1.4% [1.0%-1.7%] | 1.0% [0.8%-1.2%] |
| 1993 | 7.2% [2.1%-14.1%] | 9.1% [2.5%-16.7%] | 8.1% [2.3%-15.3%] | 1.1% [0.6%-1.6%] | 0.6% [0.3%-0.8%] | 0.9% [0.5%-1.3%] | 1.4% [-0.3%-2.9%] | 0.9% [-0.2%-1.7%] | 1.2% [-0.3%-2.4%] | 1.7% [0.7%-2.6%] | 2.1% [0.9%-3.1%] | 1.9% [0.8%-2.8%] | 0.8% [0.4%-1.2%] | 2.1% [0.6%-3.4%] | 1.4% [0.5%-2.2%] | 1.4% [-0.0%-2.9%] | 3.6% [-0.0%-7.3%] | 2.4% [-0.0%-4.8%] | 0.6% [-0.1%-1.2%] | 0.8% [-0.2%-1.7%] | 0.7% [-0.2%-1.4%] | 0.2% [0.1%-0.2%] | 0.2% [0.1%-0.3%] | 0.2% [0.1%-0.3%] | 1.2% [-0.0%-6.2%] | 0.9% [0.0%-4.7%] | 1.1% [-0.0%-5.6%] | 0.6% [0.5%-0.7%] | 1.4% [1.0%-1.7%] | 1.0% [0.8%-1.2%] |
| 1994 | 7.2% [2.1%-13.8%] | 9.1% [2.5%-16.5%] | 8.1% [2.3%-15.1%] | 1.1% [0.6%-1.6%] | 0.6% [0.3%-0.8%] | 0.9% [0.4%-1.2%] | 1.4% [-0.3%-2.8%] | 0.9% [-0.2%-1.7%] | 1.2% [-0.3%-2.3%] | 1.7% [0.7%-2.6%] | 2.1% [0.9%-3.1%] | 1.9% [0.8%-2.8%] | 0.8% [0.4%-1.2%] | 2.1% [0.6%-3.4%] | 1.4% [0.5%-2.2%] | 1.4% [-0.0%-2.9%] | 3.6% [-0.0%-7.3%] | 2.4% [-0.0%-4.8%] | 0.6% [-0.2%-1.2%] | 0.8% [-0.2%-1.6%] | 0.7% [-0.2%-1.4%] | 0.2% [0.1%-0.2%] | 0.2% [0.1%-0.3%] | 0.2% [0.1%-0.3%] | 1.2% [-0.0%-6.2%] | 0.9% [0.0%-4.6%] | 1.1% [-0.0%-5.6%] | 0.6% [0.5%-0.7%] | 1.4% [1.0%-1.7%] | 1.0% [0.8%-1.2%] |
| 1995 | 7.1% [2.1%-13.7%] | 9.0% [2.5%-16.3%] | 8.0% [2.3%-14.9%] | 1.1% [0.5%-1.5%] | 0.6% [0.3%-0.8%] | 0.9% [0.4%-1.2%] | 1.4% [-0.3%-2.8%] | 0.8% [-0.2%-1.7%] | 1.2% [-0.2%-2.3%] | 1.7% [0.7%-2.6%] | 2.1% [0.9%-3.1%] | 1.9% [0.8%-2.8%] | 0.8% [0.4%-1.2%] | 2.1% [0.6%-3.4%] | 1.4% [0.5%-2.2%] | 1.4% [-0.0%-2.9%] | 3.5% [-0.0%-7.3%] | 2.4% [-0.0%-4.8%] | 0.6% [-0.1%-1.2%] | 0.8% [-0.2%-1.6%] | 0.7% [-0.2%-1.4%] | 0.1% [0.1%-0.2%] | 0.2% [0.1%-0.3%] | 0.2% [0.1%-0.3%] | 1.2% [-0.0%-6.1%] | 0.9% [0.0%-4.5%] | 1.1% [-0.0%-5.5%] | 0.6% [0.5%-0.7%] | 1.4% [1.0%-1.7%] | 1.0% [0.8%-1.1%] |
| 1996 | 7.0% [2.1%-13.5%] | 8.9% [2.5%-16.2%] | 7.9% [2.3%-14.7%] | 1.0% [0.5%-1.5%] | 0.6% [0.3%-0.8%] | 0.8% [0.4%-1.2%] | 1.3% [-0.3%-2.6%] | 0.8% [-0.2%-1.6%] | 1.1% [-0.2%-2.2%] | 1.7% [0.7%-2.6%] | 2.1% [0.9%-3.1%] | 1.9% [0.8%-2.8%] | 0.8% [0.4%-1.2%] | 2.1% [0.6%-3.4%] | 1.4% [0.5%-2.2%] | 1.4% [-0.0%-2.9%] | 3.5% [-0.0%-7.3%] | 2.4% [-0.0%-4.8%] | 0.6% [-0.1%-1.2%] | 0.8% [-0.2%-1.6%] | 0.7% [-0.2%-1.4%] | 0.1% [0.1%-0.2%] | 0.2% [0.1%-0.3%] | 0.2% [0.1%-0.3%] | 1.2% [-0.0%-6.0%] | 0.9% [0.0%-4.4%] | 1.1% [-0.0%-5.3%] | 0.6% [0.5%-0.7%] | 1.4% [1.0%-1.7%] | 1.0% [0.8%-1.1%] |
| 1997 | 6.9% [2.1%-13.3%] | 8.8% [2.5%-15.9%] | 7.8% [2.3%-14.5%] | 1.0% [0.5%-1.5%] | 0.5% [0.3%-0.8%] | 0.8% [0.4%-1.2%] | 1.3% [-0.3%-2.6%] | 0.8% [-0.2%-1.5%] | 1.1% [-0.2%-2.2%] | 1.7% [0.7%-2.6%] | 2.1% [0.9%-3.1%] | 1.9% [0.8%-2.8%] | 0.8% [0.4%-1.2%] | 2.1% [0.6%-3.4%] | 1.4% [0.5%-2.2%] | 1.4% [-0.0%-2.9%] | 3.5% [-0.0%-7.3%] | 2.3% [-0.0%-4.8%] | 0.6% [-0.1%-1.2%] | 0.8% [-0.2%-1.6%] | 0.7% [-0.2%-1.4%] | 0.1% [0.1%-0.2%] | 0.2% [0.1%-0.3%] | 0.2% [0.1%-0.3%] | 1.2% [-0.0%-5.9%] | 0.9% [0.0%-4.3%] | 1.1% [-0.0%-5.3%] | 0.6% [0.5%-0.7%] | 1.3% [1.0%-1.7%] | 0.9% [0.8%-1.1%] |
| 1998 | 6.9% [2.1%-13.1%] | 8.8% [2.5%-15.8%] | 7.8% [2.3%-14.4%] | 1.0% [0.5%-1.5%] | 0.5% [0.3%-0.8%] | 0.8% [0.4%-1.2%] | 1.2% [-0.3%-2.5%] | 0.7% [-0.1%-1.5%] | 1.0% [-0.2%-2.1%] | 1.7% [0.7%-2.6%] | 2.1% [0.9%-3.0%] | 1.9% [0.8%-2.8%] | 0.8% [0.4%-1.2%] | 2.1% [0.6%-3.4%] | 1.4% [0.5%-2.2%] | 1.4% [-0.0%-2.9%] | 3.5% [-0.0%-7.3%] | 2.3% [-0.0%-4.8%] | 0.6% [-0.1%-1.2%] | 0.8% [-0.2%-1.6%] | 0.7% [-0.2%-1.4%] | 0.1% [0.1%-0.2%] | 0.2% [0.1%-0.3%] | 0.2% [0.1%-0.3%] | 1.2% [-0.0%-5.9%] | 0.8% [0.0%-4.3%] | 1.0% [0.0%-5.2%] | 0.6% [0.5%-0.7%] | 1.3% [1.0%-1.7%] | 0.9% [0.8%-1.1%] |
| 1999 | 6.8% [2.1%-13.1%] | 8.7% [2.5%-15.7%] | 7.7% [2.3%-14.3%] | 1.0% [0.5%-1.4%] | 0.5% [0.3%-0.8%] | 0.8% [0.4%-1.2%] | 1.2% [-0.2%-2.4%] | 0.7% [-0.1%-1.4%] | 1.0% [-0.2%-2.0%] | 1.7% [0.7%-2.6%] | 2.1% [0.9%-3.0%] | 1.9% [0.8%-2.8%] | 0.8% [0.4%-1.2%] | 2.1% [0.6%-3.4%] | 1.4% [0.5%-2.2%] | 1.4% [-0.0%-2.9%] | 3.5% [-0.0%-7.2%] | 2.3% [-0.0%-4.8%] | 0.6% [-0.1%-1.2%] | 0.8% [-0.2%-1.6%] | 0.7% [-0.2%-1.4%] | 0.1% [0.1%-0.2%] | 0.2% [0.1%-0.3%] | 0.2% [0.1%-0.3%] | 1.2% [-0.0%-5.9%] | 0.8% [0.0%-4.2%] | 1.0% [-0.0%-5.2%] | 0.6% [0.5%-0.7%] | 1.3% [1.0%-1.7%] | 0.9% [0.8%-1.1%] |
| 2000 | 6.7% [2.1%-12.9%] | 8.6% [2.5%-15.6%] | 7.6% [2.3%-14.1%] | 1.0% [0.5%-1.4%] | 0.5% [0.3%-0.8%] | 0.8% [0.4%-1.2%] | 1.1% [-0.2%-2.3%] | 0.7% [-0.1%-1.4%] | 1.0% [-0.2%-1.9%] | 1.7% [0.7%-2.6%] | 2.0% [0.9%-3.0%] | 1.9% [0.8%-2.8%] | 0.8% [0.4%-1.2%] | 2.0% [0.6%-3.3%] | 1.4% [0.5%-2.1%] | 1.4% [-0.0%-2.9%] | 3.5% [-0.0%-7.2%] | 2.3% [-0.0%-4.7%] | 0.6% [-0.1%-1.2%] | 0.8% [-0.2%-1.6%] | 0.7% [-0.2%-1.4%] | 0.1% [0.1%-0.2%] | 0.2% [0.1%-0.3%] | 0.2% [0.1%-0.3%] | 1.2% [-0.0%-5.8%] | 0.8% [0.0%-4.2%] | 1.0% [-0.0%-5.2%] | 0.6% [0.5%-0.7%] | 1.3% [1.0%-1.6%] | 0.9% [0.7%-1.1%] |
| 2001 | 6.7% [2.1%-12.8%] | 8.6% [2.4%-15.5%] | 7.6% [2.3%-14.0%] | 1.0% [0.5%-1.4%] | 0.5% [0.3%-0.8%] | 0.8% [0.4%-1.2%] | 1.1% [-0.2%-2.3%] | 0.7% [-0.1%-1.3%] | 0.9% [-0.2%-1.9%] | 1.7% [0.7%-2.6%] | 2.0% [0.9%-3.0%] | 1.9% [0.8%-2.8%] | 0.8% [0.4%-1.2%] | 2.0% [0.6%-3.3%] | 1.4% [0.5%-2.1%] | 1.4% [-0.0%-2.9%] | 3.5% [-0.0%-7.2%] | 2.3% [-0.0%-4.8%] | 0.6% [-0.1%-1.2%] | 0.8% [-0.2%-1.6%] | 0.7% [-0.2%-1.4%] | 0.1% [0.1%-0.2%] | 0.2% [0.1%-0.3%] | 0.2% [0.1%-0.2%] | 1.2% [-0.0%-5.7%] | 0.8% [0.0%-4.1%] | 1.0% [-0.0%-5.1%] | 0.6% [0.5%-0.7%] | 1.3% [1.0%-1.6%] | 0.9% [0.7%-1.1%] |
| 2002 | 6.7% [2.1%-12.7%] | 8.5% [2.4%-15.3%] | 7.5% [2.3%-13.9%] | 1.0% [0.5%-1.4%] | 0.5% [0.3%-0.8%] | 0.8% [0.4%-1.1%] | 1.1% [-0.2%-2.2%] | 0.6% [-0.1%-1.3%] | 0.9% [-0.2%-1.8%] | 1.8% [0.7%-2.6%] | 2.0% [0.9%-3.0%] | 1.9% [0.8%-2.8%] | 0.8% [0.4%-1.2%] | 2.0% [0.6%-3.3%] | 1.4% [0.5%-2.1%] | 1.5% [-0.0%-2.9%] | 3.5% [-0.0%-7.2%] | 2.3% [-0.0%-4.8%] | 0.6% [-0.1%-1.2%] | 0.8% [-0.2%-1.6%] | 0.7% [-0.2%-1.4%] | 0.1% [0.1%-0.2%] | 0.2% [0.1%-0.3%] | 0.2% [0.1%-0.2%] | 1.1% [-0.0%-5.7%] | 0.8% [0.0%-4.1%] | 1.0% [-0.0%-5.1%] | 0.6% [0.5%-0.7%] | 1.3% [1.0%-1.6%] | 0.9% [0.7%-1.1%] |
| 2003 | 6.6% [2.1%-12.7%] | 8.5% [2.4%-15.2%] | 7.5% [2.3%-13.8%] | 0.9% [0.5%-1.4%] | 0.5% [0.3%-0.8%] | 0.8% [0.4%-1.1%] | 1.0% [-0.2%-2.1%] | 0.6% [-0.1%-1.2%] | 0.9% [-0.2%-1.8%] | 1.8% [0.7%-2.6%] | 2.0% [0.9%-3.0%] | 1.9% [0.8%-2.8%] | 0.8% [0.4%-1.2%] | 2.0% [0.6%-3.3%] | 1.4% [0.5%-2.1%] | 1.5% [-0.0%-2.9%] | 3.5% [-0.0%-7.2%] | 2.3% [-0.0%-4.7%] | 0.6% [-0.1%-1.2%] | 0.8% [-0.2%-1.6%] | 0.7% [-0.2%-1.4%] | 0.1% [0.1%-0.2%] | 0.2% [0.1%-0.3%] | 0.2% [0.1%-0.2%] | 1.1% [-0.0%-5.7%] | 0.8% [0.0%-4.1%] | 1.0% [-0.0%-5.0%] | 0.6% [0.5%-0.7%] | 1.3% [1.0%-1.6%] | 0.9% [0.7%-1.1%] |
| 2004 | 6.6% [2.1%-12.6%] | 8.4% [2.4%-15.1%] | 7.4% [2.3%-13.8%] | 0.9% [0.5%-1.3%] | 0.5% [0.3%-0.8%] | 0.8% [0.4%-1.1%] | 1.0% [-0.2%-2.1%] | 0.6% [-0.1%-1.2%] | 0.8% [-0.2%-1.7%] | 1.8% [0.7%-2.6%] | 2.0% [0.9%-3.0%] | 1.9% [0.8%-2.8%] | 0.9% [0.4%-1.3%] | 2.0% [0.6%-3.3%] | 1.4% [0.5%-2.1%] | 1.5% [-0.0%-2.9%] | 3.5% [-0.0%-7.1%] | 2.3% [-0.0%-4.7%] | 0.6% [-0.1%-1.2%] | 0.7% [-0.2%-1.5%] | 0.7% [-0.2%-1.3%] | 0.1% [0.1%-0.2%] | 0.2% [0.1%-0.3%] | 0.2% [0.1%-0.2%] | 1.1% [-0.0%-5.8%] | 0.8% [0.0%-4.1%] | 1.0% [-0.0%-5.1%] | 0.6% [0.5%-0.7%] | 1.3% [1.0%-1.6%] | 0.9% [0.8%-1.1%] |
| 2005 | 6.5% [2.1%-12.4%] | 8.4% [2.4%-15.0%] | 7.4% [2.3%-13.6%] | 0.9% [0.5%-1.3%] | 0.5% [0.3%-0.8%] | 0.8% [0.4%-1.1%] | 1.0% [-0.2%-2.0%] | 0.6% [-0.1%-1.2%] | 0.8% [-0.2%-1.6%] | 1.8% [0.8%-2.7%] | 2.0% [0.8%-3.0%] | 1.9% [0.8%-2.8%] | 0.9% [0.4%-1.3%] | 2.0% [0.6%-3.3%] | 1.4% [0.5%-2.1%] | 1.5% [-0.0%-3.0%] | 3.5% [-0.0%-7.1%] | 2.3% [-0.0%-4.7%] | 0.6% [-0.1%-1.2%] | 0.7% [-0.2%-1.5%] | 0.7% [-0.2%-1.4%] | 0.1% [0.1%-0.2%] | 0.2% [0.1%-0.3%] | 0.2% [0.1%-0.2%] | 1.1% [-0.0%-5.7%] | 0.8% [0.0%-4.0%] | 1.0% [-0.0%-5.0%] | 0.6% [0.5%-0.7%] | 1.3% [1.0%-1.6%] | 0.9% [0.7%-1.1%] |
| 2006 | 6.4% [2.1%-12.2%] | 8.3% [2.4%-14.8%] | 7.3% [2.2%-13.4%] | 0.9% [0.5%-1.3%] | 0.5% [0.3%-0.8%] | 0.7% [0.4%-1.1%] | 0.9% [-0.2%-1.9%] | 0.5% [-0.1%-1.1%] | 0.8% [-0.2%-1.5%] | 1.8% [0.8%-2.7%] | 2.0% [0.8%-3.0%] | 1.9% [0.8%-2.8%] | 0.9% [0.4%-1.3%] | 2.0% [0.6%-3.3%] | 1.4% [0.5%-2.2%] | 1.5% [-0.0%-3.0%] | 3.5% [-0.0%-7.1%] | 2.3% [-0.0%-4.7%] | 0.6% [-0.1%-1.2%] | 0.7% [-0.2%-1.5%] | 0.7% [-0.2%-1.3%] | 0.1% [0.1%-0.2%] | 0.2% [0.1%-0.3%] | 0.2% [0.1%-0.2%] | 1.1% [-0.0%-5.5%] | 0.8% [0.0%-3.8%] | 1.0% [0.0%-4.8%] | 0.6% [0.5%-0.7%] | 1.3% [1.0%-1.6%] | 0.9% [0.7%-1.1%] |
| 2007 | 6.4% [2.1%-12.1%] | 8.2% [2.4%-14.7%] | 7.2% [2.3%-13.3%] | 0.9% [0.5%-1.3%] | 0.5% [0.3%-0.7%] | 0.7% [0.4%-1.1%] | 0.9% [-0.2%-1.8%] | 0.5% [-0.1%-1.0%] | 0.7% [-0.2%-1.5%] | 1.8% [0.8%-2.7%] | 2.0% [0.8%-3.0%] | 1.9% [0.8%-2.8%] | 0.9% [0.4%-1.3%] | 2.0% [0.6%-3.3%] | 1.4% [0.5%-2.2%] | 1.5% [-0.0%-3.0%] | 3.5% [-0.0%-7.1%] | 2.3% [-0.0%-4.8%] | 0.6% [-0.1%-1.2%] | 0.7% [-0.2%-1.5%] | 0.7% [-0.2%-1.3%] | 0.1% [0.1%-0.2%] | 0.2% [0.1%-0.3%] | 0.2% [0.1%-0.2%] | 1.1% [-0.0%-5.4%] | 0.7% [0.0%-3.7%] | 0.9% [0.0%-4.7%] | 0.6% [0.5%-0.7%] | 1.3% [1.0%-1.6%] | 0.9% [0.7%-1.1%] |
| 2008 | 6.3% [2.1%-12.0%] | 8.2% [2.4%-14.6%] | 7.2% [2.2%-13.1%] | 0.9% [0.5%-1.3%] | 0.5% [0.3%-0.7%] | 0.7% [0.4%-1.0%] | 0.8% [-0.2%-1.7%] | 0.5% [-0.1%-1.0%] | 0.7% [-0.1%-1.4%] | 1.8% [0.8%-2.7%] | 2.0% [0.8%-3.0%] | 1.9% [0.8%-2.8%] | 0.9% [0.4%-1.3%] | 2.0% [0.6%-3.3%] | 1.4% [0.5%-2.2%] | 1.5% [-0.0%-3.0%] | 3.5% [-0.0%-7.1%] | 2.3% [-0.0%-4.8%] | 0.6% [-0.1%-1.2%] | 0.7% [-0.2%-1.5%] | 0.7% [-0.2%-1.3%] | 0.1% [0.1%-0.2%] | 0.2% [0.1%-0.3%] | 0.2% [0.1%-0.2%] | 1.1% [-0.0%-5.4%] | 0.7% [0.0%-3.6%] | 0.9% [0.0%-4.6%] | 0.6% [0.5%-0.7%] | 1.3% [1.0%-1.6%] | 0.9% [0.7%-1.1%] |
| 2009 | 6.3% [2.1%-11.8%] | 8.1% [2.4%-14.4%] | 7.1% [2.2%-13.0%] | 0.9% [0.5%-1.2%] | 0.5% [0.3%-0.7%] | 0.7% [0.4%-1.0%] | 0.8% [-0.2%-1.7%] | 0.5% [-0.1%-1.0%] | 0.7% [-0.1%-1.4%] | 1.8% [0.8%-2.7%] | 2.0% [0.8%-2.9%] | 1.9% [0.8%-2.8%] | 0.9% [0.4%-1.3%] | 2.0% [0.6%-3.3%] | 1.4% [0.5%-2.2%] | 1.5% [-0.0%-3.0%] | 3.5% [-0.0%-7.1%] | 2.3% [-0.0%-4.8%] | 0.6% [-0.1%-1.2%] | 0.7% [-0.2%-1.4%] | 0.6% [-0.2%-1.3%] | 0.1% [0.1%-0.2%] | 0.2% [0.1%-0.3%] | 0.2% [0.1%-0.2%] | 1.0% [-0.0%-5.2%] | 0.7% [0.0%-3.6%] | 0.9% [-0.0%-4.6%] | 0.6% [0.5%-0.7%] | 1.3% [1.0%-1.6%] | 0.9% [0.7%-1.1%] |
| 2010 | 6.2% [2.1%-11.8%] | 8.1% [2.3%-14.3%] | 7.1% [2.2%-12.9%] | 0.8% [0.4%-1.2%] | 0.5% [0.3%-0.7%] | 0.7% [0.4%-1.0%] | 0.8% [-0.2%-1.6%] | 0.5% [-0.1%-0.9%] | 0.7% [-0.1%-1.3%] | 1.8% [0.8%-2.7%] | 2.0% [0.8%-2.9%] | 1.9% [0.8%-2.8%] | 0.9% [0.4%-1.3%] | 2.0% [0.6%-3.3%] | 1.4% [0.5%-2.2%] | 1.5% [-0.0%-3.0%] | 3.5% [-0.0%-7.1%] | 2.3% [-0.0%-4.8%] | 0.6% [-0.1%-1.2%] | 0.7% [-0.2%-1.4%] | 0.6% [-0.2%-1.3%] | 0.1% [0.1%-0.2%] | 0.2% [0.1%-0.3%] | 0.1% [0.1%-0.2%] | 1.0% [-0.0%-5.2%] | 0.7% [0.0%-3.5%] | 0.9% [-0.0%-4.5%] | 0.6% [0.5%-0.7%] | 1.3% [1.0%-1.6%] | 0.9% [0.7%-1.1%] |
| 2011 | 6.2% [2.1%-11.6%] | 8.0% [2.3%-14.2%] | 7.0% [2.2%-12.8%] | 0.8% [0.4%-1.2%] | 0.5% [0.3%-0.7%] | 0.7% [0.4%-1.0%] | 0.8% [-0.2%-1.6%] | 0.5% [-0.1%-0.9%] | 0.6% [-0.1%-1.3%] | 1.8% [0.8%-2.7%] | 2.0% [0.8%-2.9%] | 1.9% [0.8%-2.8%] | 0.9% [0.4%-1.4%] | 2.0% [0.6%-3.3%] | 1.4% [0.5%-2.2%] | 1.5% [-0.0%-3.1%] | 3.5% [-0.0%-7.1%] | 2.4% [-0.0%-4.8%] | 0.6% [-0.1%-1.2%] | 0.7% [-0.2%-1.4%] | 0.6% [-0.2%-1.3%] | 0.1% [0.1%-0.2%] | 0.2% [0.1%-0.3%] | 0.1% [0.1%-0.2%] | 1.0% [-0.0%-5.0%] | 0.7% [0.0%-3.4%] | 0.9% [-0.0%-4.4%] | 0.6% [0.5%-0.7%] | 1.3% [0.9%-1.5%] | 0.9% [0.7%-1.1%] |
| 2012 | 6.2% [2.1%-11.6%] | 8.0% [2.3%-14.1%] | 7.0% [2.2%-12.7%] | 0.8% [0.4%-1.2%] | 0.5% [0.3%-0.7%] | 0.7% [0.4%-1.0%] | 0.7% [-0.2%-1.5%] | 0.4% [-0.1%-0.9%] | 0.6% [-0.1%-1.2%] | 1.8% [0.8%-2.7%] | 2.0% [0.8%-2.9%] | 1.9% [0.8%-2.8%] | 0.9% [0.5%-1.4%] | 2.0% [0.6%-3.3%] | 1.4% [0.5%-2.2%] | 1.5% [-0.0%-3.1%] | 3.5% [-0.0%-7.1%] | 2.4% [-0.0%-4.8%] | 0.6% [-0.1%-1.2%] | 0.7% [-0.2%-1.4%] | 0.6% [-0.2%-1.3%] | 0.1% [0.1%-0.2%] | 0.2% [0.1%-0.3%] | 0.1% [0.1%-0.2%] | 1.0% [-0.0%-5.0%] | 0.7% [0.0%-3.3%] | 0.9% [-0.0%-4.3%] | 0.6% [0.5%-0.7%] | 1.2% [0.9%-1.5%] | 0.9% [0.7%-1.0%] |
| 2013 | 6.1% [2.1%-11.4%] | 7.9% [2.3%-14.0%] | 6.9% [2.2%-12.6%] | 0.8% [0.4%-1.2%] | 0.5% [0.3%-0.7%] | 0.7% [0.4%-1.0%] | 0.7% [-0.1%-1.5%] | 0.4% [-0.1%-0.9%] | 0.6% [-0.1%-1.2%] | 1.8% [0.8%-2.7%] | 1.9% [0.8%-2.9%] | 1.9% [0.8%-2.8%] | 0.9% [0.5%-1.4%] | 2.0% [0.6%-3.3%] | 1.4% [0.5%-2.2%] | 1.5% [-0.0%-3.1%] | 3.5% [-0.0%-7.1%] | 2.4% [-0.0%-4.8%] | 0.6% [-0.1%-1.2%] | 0.7% [-0.2%-1.4%] | 0.6% [-0.2%-1.3%] | 0.1% [0.1%-0.2%] | 0.2% [0.1%-0.2%] | 0.1% [0.1%-0.2%] | 1.0% [-0.0%-4.9%] | 0.6% [0.0%-3.3%] | 0.8% [-0.0%-4.3%] | 0.6% [0.4%-0.7%] | 1.2% [0.9%-1.5%] | 0.9% [0.7%-1.0%] |
| 2014 | 6.1% [2.1%-11.3%] | 7.9% [2.3%-13.9%] | 6.9% [2.2%-12.5%] | 0.8% [0.4%-1.2%] | 0.5% [0.3%-0.7%] | 0.7% [0.4%-1.0%] | 0.7% [-0.1%-1.4%] | 0.4% [-0.1%-0.9%] | 0.6% [-0.1%-1.2%] | 1.8% [0.8%-2.7%] | 1.9% [0.8%-2.9%] | 1.9% [0.8%-2.8%] | 1.0% [0.5%-1.4%] | 2.0% [0.6%-3.3%] | 1.4% [0.5%-2.2%] | 1.5% [-0.0%-3.1%] | 3.4% [-0.0%-7.1%] | 2.4% [-0.0%-4.8%] | 0.6% [-0.1%-1.2%] | 0.7% [-0.2%-1.4%] | 0.6% [-0.2%-1.3%] | 0.1% [0.1%-0.2%] | 0.2% [0.1%-0.2%] | 0.1% [0.1%-0.2%] | 1.0% [-0.0%-4.8%] | 0.6% [0.0%-3.2%] | 0.8% [-0.0%-4.1%] | 0.6% [0.4%-0.7%] | 1.2% [0.9%-1.5%] | 0.9% [0.7%-1.0%] |
| 2015 | 6.1% [2.1%-11.2%] | 7.9% [2.3%-13.9%] | 6.9% [2.2%-12.4%] | 0.8% [0.4%-1.1%] | 0.5% [0.3%-0.7%] | 0.7% [0.4%-1.0%] | 0.7% [-0.2%-1.4%] | 0.4% [-0.1%-0.9%] | 0.6% [-0.1%-1.2%] | 1.9% [0.8%-2.8%] | 1.9% [0.8%-2.9%] | 1.9% [0.8%-2.8%] | 1.0% [0.5%-1.4%] | 2.0% [0.6%-3.3%] | 1.4% [0.5%-2.2%] | 1.6% [-0.0%-3.1%] | 3.4% [-0.0%-7.1%] | 2.4% [-0.0%-4.8%] | 0.6% [-0.1%-1.2%] | 0.7% [-0.2%-1.4%] | 0.6% [-0.2%-1.3%] | 0.1% [0.1%-0.2%] | 0.2% [0.1%-0.2%] | 0.1% [0.1%-0.2%] | 0.9% [-0.0%-4.7%] | 0.6% [0.0%-3.1%] | 0.8% [-0.0%-4.0%] | 0.6% [0.4%-0.7%] | 1.2% [0.9%-1.5%] | 0.9% [0.7%-1.0%] |
| 2016 | 6.1% [2.1%-11.2%] | 7.8% [2.3%-13.8%] | 6.9% [2.2%-12.4%] | 0.8% [0.4%-1.1%] | 0.5% [0.3%-0.7%] | 0.7% [0.4%-1.0%] | 0.7% [-0.2%-1.4%] | 0.4% [-0.1%-0.9%] | 0.6% [-0.1%-1.2%] | 1.9% [0.8%-2.8%] | 1.9% [0.8%-2.9%] | 1.9% [0.8%-2.8%] | 1.0% [0.5%-1.4%] | 2.0% [0.6%-3.3%] | 1.5% [0.5%-2.3%] | 1.6% [-0.0%-3.1%] | 3.4% [-0.0%-7.1%] | 2.4% [-0.0%-4.9%] | 0.6% [-0.1%-1.2%] | 0.7% [-0.2%-1.4%] | 0.6% [-0.2%-1.3%] | 0.1% [0.1%-0.2%] | 0.2% [0.1%-0.2%] | 0.1% [0.1%-0.2%] | 0.9% [-0.0%-4.6%] | 0.6% [0.0%-3.1%] | 0.8% [-0.0%-4.0%] | 0.5% [0.4%-0.7%] | 1.2% [0.9%-1.5%] | 0.9% [0.7%-1.0%] |
| 2017 | 6.0% [2.1%-11.1%] | 7.8% [2.3%-13.8%] | 6.9% [2.2%-12.3%] | 0.8% [0.4%-1.1%] | 0.5% [0.3%-0.7%] | 0.7% [0.4%-1.0%] | 0.7% [-0.2%-1.4%] | 0.4% [-0.1%-0.8%] | 0.6% [-0.1%-1.2%] | 1.9% [0.8%-2.8%] | 1.9% [0.8%-2.8%] | 1.9% [0.8%-2.8%] | 1.0% [0.5%-1.5%] | 2.0% [0.6%-3.3%] | 1.5% [0.5%-2.3%] | 1.6% [-0.0%-3.1%] | 3.4% [-0.0%-7.1%] | 2.4% [-0.0%-4.8%] | 0.6% [-0.1%-1.2%] | 0.7% [-0.2%-1.3%] | 0.6% [-0.2%-1.3%] | 0.1% [0.1%-0.2%] | 0.2% [0.1%-0.2%] | 0.1% [0.1%-0.2%] | 0.9% [-0.0%-4.6%] | 0.6% [0.0%-3.0%] | 0.8% [-0.0%-3.9%] | 0.5% [0.4%-0.7%] | 1.2% [0.9%-1.5%] | 0.9% [0.7%-1.0%] |
| 2018 | 6.0% [2.1%-11.0%] | 7.8% [2.3%-13.7%] | 6.8% [2.2%-12.2%] | 0.8% [0.4%-1.1%] | 0.5% [0.3%-0.7%] | 0.7% [0.4%-0.9%] | 0.7% [-0.1%-1.4%] | 0.4% [-0.1%-0.8%] | 0.6% [-0.1%-1.1%] | 1.9% [0.8%-2.8%] | 1.9% [0.8%-2.8%] | 1.9% [0.8%-2.8%] | 1.0% [0.5%-1.5%] | 2.0% [0.6%-3.3%] | 1.5% [0.5%-2.3%] | 1.6% [-0.0%-3.1%] | 3.4% [-0.0%-7.1%] | 2.4% [-0.0%-4.9%] | 0.6% [-0.1%-1.2%] | 0.6% [-0.2%-1.3%] | 0.6% [-0.1%-1.3%] | 0.1% [0.1%-0.2%] | 0.1% [0.1%-0.2%] | 0.1% [0.1%-0.2%] | 0.9% [-0.0%-4.5%] | 0.6% [0.0%-3.0%] | 0.8% [-0.0%-3.9%] | 0.5% [0.4%-0.7%] | 1.2% [0.9%-1.5%] | 0.9% [0.7%-1.0%] |
| 2019 | 6.0% [2.1%-11.0%] | 7.8% [2.3%-13.7%] | 6.8% [2.2%-12.2%] | 0.8% [0.4%-1.1%] | 0.5% [0.3%-0.7%] | 0.7% [0.4%-0.9%] | 0.7% [-0.2%-1.4%] | 0.4% [-0.1%-0.8%] | 0.6% [-0.1%-1.2%] | 1.9% [0.8%-2.8%] | 1.9% [0.8%-2.8%] | 1.9% [0.8%-2.8%] | 1.0% [0.5%-1.5%] | 2.0% [0.6%-3.3%] | 1.5% [0.5%-2.3%] | 1.6% [-0.0%-3.1%] | 3.4% [-0.0%-7.1%] | 2.4% [-0.0%-4.9%] | 0.6% [-0.1%-1.2%] | 0.6% [-0.2%-1.3%] | 0.6% [-0.1%-1.2%] | 0.1% [0.1%-0.2%] | 0.1% [0.1%-0.2%] | 0.1% [0.1%-0.2%] | 0.9% [-0.0%-4.5%] | 0.6% [0.0%-3.0%] | 0.8% [-0.0%-3.9%] | 0.6% [0.4%-0.7%] | 1.2% [0.9%-1.5%] | 0.9% [0.7%-1.0%] |
| 2020 | 6.0% [2.1%-10.9%] | 7.7% [2.3%-13.7%] | 6.8% [2.2%-12.1%] | 0.8% [0.4%-1.1%] | 0.5% [0.3%-0.7%] | 0.7% [0.4%-0.9%] | 0.7% [-0.2%-1.4%] | 0.4% [-0.1%-0.8%] | 0.6% [-0.1%-1.1%] | 1.9% [0.8%-2.8%] | 1.9% [0.8%-2.8%] | 1.9% [0.8%-2.8%] | 1.0% [0.5%-1.5%] | 2.0% [0.6%-3.3%] | 1.5% [0.5%-2.3%] | 1.6% [-0.0%-3.1%] | 3.4% [-0.0%-7.1%] | 2.4% [-0.0%-4.9%] | 0.6% [-0.1%-1.2%] | 0.6% [-0.2%-1.3%] | 0.6% [-0.1%-1.2%] | 0.1% [0.1%-0.2%] | 0.1% [0.1%-0.2%] | 0.1% [0.1%-0.2%] | 0.9% [-0.0%-4.4%] | 0.6% [0.0%-2.9%] | 0.8% [-0.0%-3.8%] | 0.6% [0.4%-0.7%] | 1.2% [0.9%-1.5%] | 0.9% [0.7%-1.0%] |
| 2021 | 6.0% [2.1%-10.9%] | 7.7% [2.3%-13.6%] | 6.8% [2.2%-12.1%] | 0.8% [0.4%-1.1%] | 0.5% [0.3%-0.7%] | 0.7% [0.4%-0.9%] | 0.7% [-0.2%-1.4%] | 0.4% [-0.1%-0.8%] | 0.6% [-0.1%-1.2%] | 1.9% [0.8%-2.8%] | 1.9% [0.8%-2.8%] | 1.9% [0.8%-2.8%] | 1.0% [0.5%-1.5%] | 2.1% [0.6%-3.4%] | 1.5% [0.5%-2.3%] | 1.6% [-0.0%-3.2%] | 3.4% [-0.0%-7.1%] | 2.4% [-0.0%-4.9%] | 0.6% [-0.1%-1.1%] | 0.6% [-0.1%-1.3%] | 0.6% [-0.1%-1.2%] | 0.1% [0.1%-0.2%] | 0.1% [0.1%-0.2%] | 0.1% [0.1%-0.2%] | 0.9% [-0.0%-4.4%] | 0.6% [0.0%-3.0%] | 0.8% [-0.0%-3.8%] | 0.6% [0.4%-0.7%] | 1.2% [0.9%-1.5%] | 0.9% [0.7%-1.0%] |
| **Note:** Data are presented as percentages (%) with 95% confidence intervals [lower-upper]. Values of "-0.0%" and "0.0%" result from rounding to one decimal place. "-0.0%" indicates that the original value was negative but rounded to zero (absolute value <0.05%), while "0.0%" indicates a positive value that rounded to zero (<0.05%). Negative values suggest an inverse relationship between the dietary factor and cancer mortality, while positive values indicate a positive association. All rates are age-standardized. | | | | | | | | | | | | | | | | | | | | | | | | | | | | | | |

| Table S1b. Percentage of Total Cancer Mortality Attributable to Dietary Risk Factors - High SDI Regions (Age-standardized, 1990-2021). | | | | | | | | | | | | | | | | | | | | | | | | | | | | | | |
| --- | --- | --- | --- | --- | --- | --- | --- | --- | --- | --- | --- | --- | --- | --- | --- | --- | --- | --- | --- | --- | --- | --- | --- | --- | --- | --- | --- | --- | --- | --- |
|  | All dietary risks | | | Low in fruits | | | Low in vegetables | | | Low in whole grains | | | Low in milk | | | High in red meat | | | High in processed meat | | | Low in fiber | | | High in sodium | | | Low in calcium | | |
| Year | Male | Female | Both | Male | Female | Both | Male | Female | Both | Male | Female | Both | Male | Female | Both | Male | Female | Both | Male | Female | Both | Male | Female | Both | Male | Female | Both | Male | Female | Both |
| 1990 | 6.8% [1.8%-12.4%] | 9.6% [2.3%-17.1%] | 8.1% [2.1%-14.4%] | 0.9% [0.5%-1.3%] | 0.5% [0.3%-0.7%] | 0.7% [0.4%-1.1%] | 0.7% [-0.2%-1.4%] | 0.3% [-0.1%-0.6%] | 0.6% [-0.1%-1.1%] | 2.1% [0.9%-3.1%] | 2.5% [1.0%-3.7%] | 2.3% [1.0%-3.4%] | 1.0% [0.3%-1.6%] | 2.3% [0.6%-3.8%] | 1.6% [0.4%-2.6%] | 1.9% [-0.0%-3.7%] | 4.7% [-0.0%-9.6%] | 3.1% [-0.0%-6.3%] | 1.1% [-0.3%-2.2%] | 1.3% [-0.3%-2.7%] | 1.2% [-0.3%-2.4%] | 0.2% [0.1%-0.3%] | 0.2% [0.1%-0.4%] | 0.2% [0.1%-0.3%] | 0.8% [0.0%-4.1%] | 0.6% [0.0%-3.2%] | 0.7% [0.0%-3.7%] | 0.2% [0.1%-0.3%] | 1.0% [0.7%-1.3%] | 0.6% [0.4%-0.8%] |
| 1991 | 6.8% [1.8%-12.3%] | 9.6% [2.3%-17.0%] | 8.1% [2.1%-14.2%] | 0.9% [0.5%-1.3%] | 0.5% [0.3%-0.7%] | 0.7% [0.4%-1.1%] | 0.7% [-0.2%-1.4%] | 0.3% [-0.1%-0.6%] | 0.5% [-0.1%-1.1%] | 2.1% [0.9%-3.1%] | 2.5% [1.0%-3.7%] | 2.3% [1.0%-3.4%] | 1.0% [0.3%-1.7%] | 2.3% [0.6%-3.8%] | 1.6% [0.4%-2.6%] | 1.9% [-0.0%-3.7%] | 4.7% [-0.0%-9.6%] | 3.1% [-0.0%-6.3%] | 1.1% [-0.3%-2.2%] | 1.3% [-0.3%-2.7%] | 1.2% [-0.3%-2.4%] | 0.2% [0.1%-0.3%] | 0.2% [0.1%-0.4%] | 0.2% [0.1%-0.3%] | 0.8% [0.0%-4.0%] | 0.6% [0.0%-3.1%] | 0.7% [0.0%-3.7%] | 0.2% [0.1%-0.3%] | 1.0% [0.7%-1.3%] | 0.6% [0.4%-0.8%] |
| 1992 | 6.8% [1.8%-12.2%] | 9.5% [2.3%-17.0%] | 8.1% [2.1%-14.2%] | 0.9% [0.5%-1.3%] | 0.5% [0.3%-0.7%] | 0.7% [0.4%-1.0%] | 0.7% [-0.2%-1.4%] | 0.3% [-0.1%-0.6%] | 0.5% [-0.1%-1.1%] | 2.1% [0.9%-3.1%] | 2.5% [1.0%-3.7%] | 2.3% [1.0%-3.4%] | 1.0% [0.3%-1.7%] | 2.3% [0.6%-3.8%] | 1.6% [0.4%-2.6%] | 1.9% [-0.0%-3.7%] | 4.7% [-0.0%-9.6%] | 3.1% [-0.0%-6.3%] | 1.1% [-0.3%-2.2%] | 1.3% [-0.3%-2.7%] | 1.2% [-0.3%-2.4%] | 0.2% [0.1%-0.3%] | 0.2% [0.1%-0.4%] | 0.2% [0.1%-0.3%] | 0.8% [0.0%-3.9%] | 0.6% [0.0%-3.0%] | 0.7% [0.0%-3.6%] | 0.2% [0.1%-0.3%] | 1.0% [0.7%-1.3%] | 0.6% [0.4%-0.8%] |
| 1993 | 6.7% [1.7%-12.1%] | 9.5% [2.3%-16.9%] | 8.0% [2.1%-14.1%] | 0.9% [0.4%-1.3%] | 0.5% [0.3%-0.7%] | 0.7% [0.4%-1.0%] | 0.7% [-0.2%-1.4%] | 0.3% [-0.1%-0.6%] | 0.5% [-0.1%-1.1%] | 2.1% [0.9%-3.1%] | 2.5% [1.0%-3.7%] | 2.3% [1.0%-3.4%] | 1.0% [0.3%-1.7%] | 2.3% [0.6%-3.8%] | 1.6% [0.4%-2.6%] | 1.9% [-0.0%-3.7%] | 4.6% [-0.0%-9.6%] | 3.1% [-0.0%-6.3%] | 1.1% [-0.3%-2.2%] | 1.3% [-0.3%-2.7%] | 1.2% [-0.3%-2.4%] | 0.2% [0.1%-0.3%] | 0.2% [0.1%-0.3%] | 0.2% [0.1%-0.3%] | 0.8% [0.0%-3.9%] | 0.6% [0.0%-2.9%] | 0.7% [0.0%-3.5%] | 0.2% [0.1%-0.3%] | 1.0% [0.7%-1.3%] | 0.6% [0.4%-0.7%] |
| 1994 | 6.7% [1.7%-12.0%] | 9.4% [2.2%-16.8%] | 8.0% [2.0%-14.0%] | 0.9% [0.4%-1.2%] | 0.5% [0.3%-0.7%] | 0.7% [0.4%-1.0%] | 0.7% [-0.2%-1.4%] | 0.3% [-0.1%-0.6%] | 0.5% [-0.1%-1.1%] | 2.1% [0.9%-3.1%] | 2.4% [1.0%-3.6%] | 2.3% [1.0%-3.4%] | 1.0% [0.3%-1.7%] | 2.3% [0.6%-3.8%] | 1.6% [0.4%-2.6%] | 1.9% [-0.0%-3.7%] | 4.6% [-0.0%-9.5%] | 3.1% [-0.0%-6.3%] | 1.1% [-0.3%-2.2%] | 1.3% [-0.3%-2.7%] | 1.2% [-0.3%-2.4%] | 0.2% [0.1%-0.3%] | 0.2% [0.1%-0.3%] | 0.2% [0.1%-0.3%] | 0.8% [0.0%-3.8%] | 0.6% [0.0%-2.9%] | 0.7% [0.0%-3.4%] | 0.2% [0.1%-0.3%] | 1.0% [0.7%-1.3%] | 0.6% [0.4%-0.7%] |
| 1995 | 6.7% [1.7%-11.9%] | 9.4% [2.2%-16.6%] | 7.9% [2.0%-13.8%] | 0.8% [0.4%-1.2%] | 0.5% [0.3%-0.7%] | 0.7% [0.4%-1.0%] | 0.7% [-0.2%-1.4%] | 0.3% [-0.1%-0.6%] | 0.5% [-0.1%-1.1%] | 2.1% [0.9%-3.1%] | 2.4% [1.0%-3.6%] | 2.3% [1.0%-3.4%] | 1.0% [0.3%-1.7%] | 2.2% [0.6%-3.7%] | 1.6% [0.4%-2.6%] | 1.9% [-0.0%-3.7%] | 4.6% [-0.0%-9.4%] | 3.1% [-0.0%-6.2%] | 1.1% [-0.3%-2.2%] | 1.3% [-0.3%-2.7%] | 1.2% [-0.3%-2.4%] | 0.2% [0.1%-0.3%] | 0.2% [0.1%-0.3%] | 0.2% [0.1%-0.3%] | 0.7% [0.0%-3.7%] | 0.5% [0.0%-2.8%] | 0.7% [0.0%-3.4%] | 0.2% [0.1%-0.3%] | 1.0% [0.7%-1.3%] | 0.6% [0.4%-0.7%] |
| 1996 | 6.6% [1.7%-11.8%] | 9.3% [2.2%-16.5%] | 7.9% [2.0%-13.7%] | 0.8% [0.4%-1.2%] | 0.5% [0.3%-0.7%] | 0.7% [0.4%-1.0%] | 0.7% [-0.2%-1.4%] | 0.3% [-0.1%-0.6%] | 0.5% [-0.1%-1.1%] | 2.1% [0.9%-3.1%] | 2.4% [1.0%-3.6%] | 2.3% [1.0%-3.4%] | 1.0% [0.3%-1.7%] | 2.2% [0.6%-3.7%] | 1.6% [0.4%-2.6%] | 1.9% [-0.0%-3.7%] | 4.5% [-0.0%-9.3%] | 3.0% [-0.0%-6.2%] | 1.1% [-0.3%-2.2%] | 1.3% [-0.3%-2.7%] | 1.2% [-0.3%-2.4%] | 0.2% [0.1%-0.2%] | 0.2% [0.1%-0.3%] | 0.2% [0.1%-0.3%] | 0.7% [0.0%-3.7%] | 0.5% [0.0%-2.8%] | 0.6% [0.0%-3.3%] | 0.2% [0.1%-0.3%] | 1.0% [0.7%-1.3%] | 0.6% [0.4%-0.7%] |
| 1997 | 6.6% [1.7%-11.8%] | 9.2% [2.2%-16.3%] | 7.9% [2.0%-13.7%] | 0.8% [0.4%-1.2%] | 0.5% [0.3%-0.7%] | 0.7% [0.4%-1.0%] | 0.7% [-0.2%-1.4%] | 0.3% [-0.1%-0.6%] | 0.5% [-0.1%-1.1%] | 2.1% [0.9%-3.1%] | 2.4% [1.0%-3.6%] | 2.3% [1.0%-3.4%] | 1.0% [0.3%-1.7%] | 2.2% [0.6%-3.7%] | 1.6% [0.4%-2.6%] | 1.9% [-0.0%-3.7%] | 4.5% [-0.0%-9.2%] | 3.0% [-0.0%-6.2%] | 1.1% [-0.3%-2.2%] | 1.3% [-0.3%-2.7%] | 1.2% [-0.3%-2.4%] | 0.2% [0.1%-0.2%] | 0.2% [0.1%-0.3%] | 0.2% [0.1%-0.3%] | 0.7% [0.0%-3.6%] | 0.5% [0.0%-2.7%] | 0.6% [0.0%-3.3%] | 0.2% [0.1%-0.3%] | 1.0% [0.7%-1.2%] | 0.6% [0.4%-0.7%] |
| 1998 | 6.6% [1.7%-11.8%] | 9.2% [2.1%-16.2%] | 7.8% [2.0%-13.6%] | 0.8% [0.4%-1.2%] | 0.5% [0.3%-0.7%] | 0.7% [0.4%-1.0%] | 0.7% [-0.2%-1.4%] | 0.3% [-0.1%-0.6%] | 0.5% [-0.1%-1.1%] | 2.1% [0.9%-3.1%] | 2.4% [1.0%-3.5%] | 2.3% [0.9%-3.4%] | 1.0% [0.3%-1.6%] | 2.2% [0.6%-3.6%] | 1.5% [0.4%-2.6%] | 1.9% [-0.0%-3.7%] | 4.5% [-0.0%-9.2%] | 3.0% [-0.0%-6.1%] | 1.1% [-0.3%-2.2%] | 1.3% [-0.3%-2.6%] | 1.2% [-0.3%-2.4%] | 0.2% [0.1%-0.2%] | 0.2% [0.1%-0.3%] | 0.2% [0.1%-0.3%] | 0.7% [0.0%-3.6%] | 0.5% [0.0%-2.6%] | 0.6% [0.0%-3.2%] | 0.2% [0.1%-0.3%] | 0.9% [0.7%-1.2%] | 0.6% [0.4%-0.7%] |
| 1999 | 6.6% [1.7%-11.7%] | 9.1% [2.1%-16.1%] | 7.8% [2.0%-13.5%] | 0.8% [0.4%-1.2%] | 0.5% [0.3%-0.7%] | 0.7% [0.3%-1.0%] | 0.7% [-0.2%-1.4%] | 0.3% [-0.1%-0.6%] | 0.5% [-0.1%-1.1%] | 2.1% [0.9%-3.2%] | 2.4% [1.0%-3.5%] | 2.3% [0.9%-3.4%] | 1.0% [0.3%-1.6%] | 2.2% [0.6%-3.6%] | 1.5% [0.4%-2.5%] | 1.9% [-0.0%-3.7%] | 4.4% [-0.0%-9.1%] | 3.0% [-0.0%-6.1%] | 1.1% [-0.3%-2.2%] | 1.3% [-0.3%-2.6%] | 1.2% [-0.3%-2.4%] | 0.2% [0.1%-0.2%] | 0.2% [0.1%-0.3%] | 0.2% [0.1%-0.3%] | 0.7% [0.0%-3.6%] | 0.5% [0.0%-2.6%] | 0.6% [0.0%-3.2%] | 0.2% [0.1%-0.3%] | 0.9% [0.7%-1.2%] | 0.5% [0.4%-0.7%] |
| 2000 | 6.6% [1.7%-11.7%] | 9.0% [2.1%-15.9%] | 7.8% [2.0%-13.5%] | 0.8% [0.4%-1.2%] | 0.5% [0.3%-0.7%] | 0.7% [0.3%-1.0%] | 0.7% [-0.2%-1.4%] | 0.3% [-0.1%-0.6%] | 0.5% [-0.1%-1.1%] | 2.1% [0.9%-3.2%] | 2.4% [1.0%-3.5%] | 2.3% [0.9%-3.4%] | 1.0% [0.3%-1.6%] | 2.1% [0.6%-3.6%] | 1.5% [0.4%-2.5%] | 1.9% [-0.0%-3.7%] | 4.4% [-0.0%-9.0%] | 3.0% [-0.0%-6.1%] | 1.1% [-0.3%-2.2%] | 1.3% [-0.3%-2.6%] | 1.2% [-0.3%-2.4%] | 0.2% [0.1%-0.2%] | 0.2% [0.1%-0.3%] | 0.2% [0.1%-0.3%] | 0.7% [0.0%-3.5%] | 0.5% [0.0%-2.5%] | 0.6% [0.0%-3.1%] | 0.2% [0.1%-0.3%] | 0.9% [0.7%-1.2%] | 0.5% [0.4%-0.7%] |
| 2001 | 6.6% [1.7%-11.7%] | 9.0% [2.1%-15.8%] | 7.7% [2.0%-13.4%] | 0.8% [0.4%-1.1%] | 0.5% [0.3%-0.7%] | 0.7% [0.3%-1.0%] | 0.7% [-0.2%-1.4%] | 0.3% [-0.1%-0.6%] | 0.5% [-0.1%-1.1%] | 2.1% [0.9%-3.2%] | 2.4% [1.0%-3.5%] | 2.3% [1.0%-3.4%] | 1.0% [0.3%-1.6%] | 2.1% [0.6%-3.5%] | 1.5% [0.4%-2.5%] | 1.9% [-0.0%-3.8%] | 4.4% [-0.0%-9.0%] | 3.0% [-0.0%-6.1%] | 1.1% [-0.3%-2.2%] | 1.3% [-0.3%-2.6%] | 1.2% [-0.3%-2.4%] | 0.2% [0.1%-0.2%] | 0.2% [0.1%-0.3%] | 0.2% [0.1%-0.3%] | 0.7% [0.0%-3.4%] | 0.5% [0.0%-2.5%] | 0.6% [0.0%-3.0%] | 0.2% [0.1%-0.3%] | 0.9% [0.7%-1.2%] | 0.5% [0.4%-0.7%] |
| 2002 | 6.6% [1.7%-11.6%] | 9.0% [2.1%-15.7%] | 7.7% [2.0%-13.4%] | 0.8% [0.4%-1.1%] | 0.5% [0.3%-0.7%] | 0.7% [0.3%-1.0%] | 0.7% [-0.2%-1.4%] | 0.3% [-0.1%-0.6%] | 0.5% [-0.1%-1.1%] | 2.1% [0.9%-3.2%] | 2.3% [1.0%-3.5%] | 2.3% [1.0%-3.4%] | 1.0% [0.3%-1.6%] | 2.1% [0.6%-3.5%] | 1.5% [0.4%-2.5%] | 1.9% [-0.0%-3.8%] | 4.4% [-0.0%-9.0%] | 3.0% [-0.0%-6.1%] | 1.1% [-0.3%-2.3%] | 1.3% [-0.3%-2.6%] | 1.2% [-0.3%-2.5%] | 0.2% [0.1%-0.2%] | 0.2% [0.1%-0.3%] | 0.2% [0.1%-0.3%] | 0.7% [0.0%-3.4%] | 0.5% [0.0%-2.4%] | 0.6% [0.0%-3.0%] | 0.2% [0.1%-0.3%] | 0.9% [0.7%-1.2%] | 0.5% [0.4%-0.7%] |
| 2003 | 6.6% [1.7%-11.6%] | 8.9% [2.1%-15.7%] | 7.7% [2.0%-13.4%] | 0.8% [0.4%-1.1%] | 0.5% [0.3%-0.8%] | 0.7% [0.3%-1.0%] | 0.7% [-0.2%-1.4%] | 0.3% [-0.1%-0.5%] | 0.5% [-0.1%-1.1%] | 2.1% [0.9%-3.2%] | 2.3% [1.0%-3.5%] | 2.3% [1.0%-3.4%] | 1.0% [0.3%-1.6%] | 2.1% [0.6%-3.5%] | 1.5% [0.4%-2.5%] | 1.9% [-0.0%-3.8%] | 4.3% [-0.0%-9.0%] | 3.0% [-0.0%-6.0%] | 1.1% [-0.3%-2.3%] | 1.3% [-0.3%-2.6%] | 1.2% [-0.3%-2.5%] | 0.2% [0.1%-0.2%] | 0.2% [0.1%-0.3%] | 0.2% [0.1%-0.3%] | 0.7% [0.0%-3.3%] | 0.5% [0.0%-2.4%] | 0.6% [0.0%-2.9%] | 0.2% [0.1%-0.3%] | 0.9% [0.7%-1.2%] | 0.5% [0.4%-0.7%] |
| 2004 | 6.6% [1.7%-11.5%] | 8.9% [2.1%-15.5%] | 7.7% [2.0%-13.3%] | 0.8% [0.4%-1.1%] | 0.5% [0.3%-0.8%] | 0.7% [0.3%-1.0%] | 0.7% [-0.2%-1.4%] | 0.3% [-0.1%-0.5%] | 0.5% [-0.1%-1.1%] | 2.1% [0.9%-3.2%] | 2.3% [1.0%-3.5%] | 2.2% [0.9%-3.3%] | 1.0% [0.3%-1.6%] | 2.1% [0.6%-3.5%] | 1.5% [0.4%-2.5%] | 1.9% [-0.0%-3.7%] | 4.3% [-0.0%-8.8%] | 2.9% [-0.0%-6.0%] | 1.1% [-0.3%-2.3%] | 1.3% [-0.3%-2.6%] | 1.2% [-0.3%-2.4%] | 0.2% [0.1%-0.2%] | 0.2% [0.1%-0.3%] | 0.2% [0.1%-0.3%] | 0.7% [0.0%-3.3%] | 0.5% [0.0%-2.3%] | 0.6% [0.0%-2.9%] | 0.2% [0.1%-0.3%] | 0.9% [0.7%-1.2%] | 0.5% [0.4%-0.7%] |
| 2005 | 6.6% [1.7%-11.5%] | 8.8% [2.0%-15.4%] | 7.6% [2.0%-13.2%] | 0.8% [0.4%-1.1%] | 0.5% [0.3%-0.8%] | 0.7% [0.3%-1.0%] | 0.7% [-0.2%-1.4%] | 0.3% [-0.1%-0.5%] | 0.5% [-0.1%-1.1%] | 2.1% [0.9%-3.2%] | 2.3% [1.0%-3.4%] | 2.2% [0.9%-3.3%] | 1.0% [0.3%-1.6%] | 2.1% [0.6%-3.4%] | 1.5% [0.4%-2.4%] | 1.9% [-0.0%-3.7%] | 4.3% [-0.0%-8.8%] | 2.9% [-0.0%-6.0%] | 1.1% [-0.3%-2.3%] | 1.3% [-0.3%-2.6%] | 1.2% [-0.3%-2.4%] | 0.1% [0.1%-0.2%] | 0.2% [0.1%-0.3%] | 0.2% [0.1%-0.3%] | 0.6% [0.0%-3.3%] | 0.4% [0.0%-2.3%] | 0.6% [0.0%-2.9%] | 0.2% [0.1%-0.3%] | 0.9% [0.7%-1.2%] | 0.5% [0.4%-0.7%] |
| 2006 | 6.6% [1.7%-11.5%] | 8.7% [2.0%-15.2%] | 7.6% [2.0%-13.2%] | 0.8% [0.4%-1.1%] | 0.5% [0.3%-0.8%] | 0.7% [0.3%-1.0%] | 0.7% [-0.2%-1.5%] | 0.3% [-0.1%-0.5%] | 0.5% [-0.1%-1.1%] | 2.1% [0.9%-3.2%] | 2.3% [1.0%-3.4%] | 2.2% [0.9%-3.3%] | 1.0% [0.3%-1.6%] | 2.1% [0.6%-3.4%] | 1.5% [0.4%-2.4%] | 1.9% [-0.0%-3.7%] | 4.2% [-0.0%-8.7%] | 2.9% [-0.0%-5.9%] | 1.1% [-0.3%-2.3%] | 1.3% [-0.3%-2.6%] | 1.2% [-0.3%-2.4%] | 0.1% [0.1%-0.2%] | 0.2% [0.1%-0.3%] | 0.2% [0.1%-0.3%] | 0.6% [0.0%-3.2%] | 0.4% [0.0%-2.2%] | 0.6% [0.0%-2.8%] | 0.2% [0.1%-0.3%] | 0.9% [0.7%-1.2%] | 0.5% [0.4%-0.7%] |
| 2007 | 6.6% [1.7%-11.4%] | 8.7% [2.0%-15.1%] | 7.6% [1.9%-13.1%] | 0.8% [0.4%-1.1%] | 0.5% [0.3%-0.8%] | 0.7% [0.3%-1.0%] | 0.7% [-0.2%-1.4%] | 0.3% [-0.1%-0.5%] | 0.5% [-0.1%-1.1%] | 2.1% [0.9%-3.2%] | 2.3% [1.0%-3.4%] | 2.2% [0.9%-3.3%] | 1.0% [0.3%-1.6%] | 2.0% [0.6%-3.4%] | 1.5% [0.4%-2.4%] | 1.9% [-0.0%-3.7%] | 4.2% [-0.0%-8.7%] | 2.9% [-0.0%-5.9%] | 1.1% [-0.3%-2.3%] | 1.3% [-0.3%-2.5%] | 1.2% [-0.3%-2.4%] | 0.1% [0.1%-0.2%] | 0.2% [0.1%-0.3%] | 0.2% [0.1%-0.3%] | 0.6% [0.0%-3.2%] | 0.4% [0.0%-2.2%] | 0.5% [0.0%-2.7%] | 0.2% [0.1%-0.3%] | 0.9% [0.7%-1.2%] | 0.5% [0.4%-0.7%] |
| 2008 | 6.6% [1.7%-11.4%] | 8.6% [2.0%-15.1%] | 7.6% [1.9%-13.1%] | 0.8% [0.4%-1.1%] | 0.5% [0.3%-0.8%] | 0.7% [0.3%-1.0%] | 0.7% [-0.2%-1.4%] | 0.3% [-0.1%-0.5%] | 0.5% [-0.1%-1.1%] | 2.1% [0.9%-3.2%] | 2.3% [0.9%-3.4%] | 2.2% [0.9%-3.3%] | 1.0% [0.3%-1.6%] | 2.0% [0.6%-3.4%] | 1.5% [0.4%-2.4%] | 1.9% [-0.0%-3.8%] | 4.2% [-0.0%-8.6%] | 2.9% [-0.0%-5.9%] | 1.1% [-0.3%-2.3%] | 1.3% [-0.3%-2.5%] | 1.2% [-0.3%-2.4%] | 0.1% [0.1%-0.2%] | 0.2% [0.1%-0.3%] | 0.2% [0.1%-0.3%] | 0.6% [0.0%-3.1%] | 0.4% [0.0%-2.1%] | 0.5% [0.0%-2.7%] | 0.2% [0.1%-0.3%] | 0.9% [0.7%-1.2%] | 0.5% [0.4%-0.7%] |
| 2009 | 6.6% [1.7%-11.3%] | 8.6% [2.0%-14.9%] | 7.5% [1.9%-13.0%] | 0.8% [0.4%-1.1%] | 0.5% [0.3%-0.8%] | 0.7% [0.3%-1.0%] | 0.7% [-0.2%-1.4%] | 0.3% [-0.1%-0.5%] | 0.5% [-0.1%-1.1%] | 2.1% [0.9%-3.2%] | 2.2% [0.9%-3.3%] | 2.2% [0.9%-3.3%] | 1.0% [0.3%-1.6%] | 2.0% [0.5%-3.4%] | 1.5% [0.4%-2.4%] | 1.9% [-0.0%-3.7%] | 4.2% [-0.0%-8.5%] | 2.9% [-0.0%-5.9%] | 1.1% [-0.3%-2.3%] | 1.2% [-0.3%-2.5%] | 1.2% [-0.3%-2.4%] | 0.1% [0.1%-0.2%] | 0.2% [0.1%-0.3%] | 0.2% [0.1%-0.3%] | 0.6% [0.0%-3.1%] | 0.4% [0.0%-2.1%] | 0.5% [0.0%-2.7%] | 0.2% [0.1%-0.3%] | 0.9% [0.7%-1.2%] | 0.5% [0.4%-0.7%] |
| 2010 | 6.6% [1.7%-11.3%] | 8.5% [2.0%-14.9%] | 7.5% [1.9%-12.9%] | 0.8% [0.4%-1.1%] | 0.5% [0.3%-0.8%] | 0.7% [0.3%-1.0%] | 0.7% [-0.2%-1.5%] | 0.3% [-0.1%-0.5%] | 0.5% [-0.1%-1.1%] | 2.2% [0.9%-3.2%] | 2.2% [0.9%-3.3%] | 2.2% [0.9%-3.3%] | 1.0% [0.3%-1.7%] | 2.0% [0.5%-3.4%] | 1.5% [0.4%-2.4%] | 1.9% [-0.0%-3.8%] | 4.1% [-0.0%-8.5%] | 2.9% [-0.0%-5.9%] | 1.1% [-0.3%-2.3%] | 1.2% [-0.3%-2.5%] | 1.2% [-0.3%-2.4%] | 0.1% [0.1%-0.2%] | 0.2% [0.1%-0.3%] | 0.2% [0.1%-0.3%] | 0.6% [0.0%-3.1%] | 0.4% [0.0%-2.1%] | 0.5% [0.0%-2.6%] | 0.2% [0.1%-0.3%] | 0.9% [0.7%-1.2%] | 0.5% [0.4%-0.7%] |
| 2011 | 6.5% [1.7%-11.2%] | 8.5% [2.0%-14.8%] | 7.5% [1.9%-12.9%] | 0.8% [0.4%-1.1%] | 0.5% [0.3%-0.8%] | 0.7% [0.3%-1.0%] | 0.7% [-0.2%-1.4%] | 0.3% [-0.1%-0.5%] | 0.5% [-0.1%-1.1%] | 2.2% [0.9%-3.2%] | 2.2% [0.9%-3.3%] | 2.2% [0.9%-3.3%] | 1.0% [0.3%-1.7%] | 2.0% [0.5%-3.4%] | 1.5% [0.4%-2.4%] | 1.9% [-0.0%-3.8%] | 4.1% [-0.0%-8.5%] | 2.9% [-0.0%-5.9%] | 1.1% [-0.3%-2.3%] | 1.2% [-0.3%-2.5%] | 1.2% [-0.3%-2.4%] | 0.2% [0.1%-0.2%] | 0.2% [0.1%-0.3%] | 0.2% [0.1%-0.3%] | 0.6% [0.0%-3.0%] | 0.4% [0.0%-2.0%] | 0.5% [0.0%-2.6%] | 0.2% [0.1%-0.3%] | 0.9% [0.7%-1.2%] | 0.5% [0.4%-0.7%] |
| 2012 | 6.5% [1.7%-11.2%] | 8.5% [2.0%-14.7%] | 7.4% [1.9%-12.8%] | 0.7% [0.4%-1.1%] | 0.6% [0.3%-0.8%] | 0.7% [0.3%-1.0%] | 0.7% [-0.2%-1.4%] | 0.3% [-0.1%-0.5%] | 0.5% [-0.1%-1.0%] | 2.2% [0.9%-3.2%] | 2.2% [0.9%-3.3%] | 2.2% [0.9%-3.3%] | 1.0% [0.3%-1.7%] | 2.0% [0.5%-3.3%] | 1.5% [0.4%-2.4%] | 1.9% [-0.0%-3.8%] | 4.1% [-0.0%-8.4%] | 2.9% [-0.0%-5.9%] | 1.1% [-0.3%-2.3%] | 1.2% [-0.3%-2.4%] | 1.2% [-0.3%-2.4%] | 0.2% [0.1%-0.2%] | 0.2% [0.1%-0.3%] | 0.2% [0.1%-0.3%] | 0.6% [0.0%-3.0%] | 0.4% [0.0%-2.0%] | 0.5% [0.0%-2.5%] | 0.2% [0.1%-0.3%] | 0.9% [0.7%-1.2%] | 0.5% [0.4%-0.7%] |
| 2013 | 6.5% [1.7%-11.1%] | 8.4% [2.0%-14.6%] | 7.4% [1.9%-12.8%] | 0.7% [0.4%-1.1%] | 0.6% [0.3%-0.8%] | 0.7% [0.3%-1.0%] | 0.7% [-0.2%-1.4%] | 0.3% [-0.1%-0.5%] | 0.5% [-0.1%-1.0%] | 2.2% [0.9%-3.2%] | 2.2% [0.9%-3.3%] | 2.2% [0.9%-3.3%] | 1.0% [0.3%-1.7%] | 2.0% [0.5%-3.4%] | 1.5% [0.4%-2.4%] | 1.9% [-0.0%-3.8%] | 4.1% [-0.0%-8.4%] | 2.9% [-0.0%-5.9%] | 1.1% [-0.3%-2.3%] | 1.2% [-0.3%-2.4%] | 1.2% [-0.3%-2.4%] | 0.2% [0.1%-0.2%] | 0.2% [0.1%-0.3%] | 0.2% [0.1%-0.3%] | 0.6% [0.0%-2.9%] | 0.4% [0.0%-1.9%] | 0.5% [0.0%-2.5%] | 0.2% [0.1%-0.3%] | 0.9% [0.6%-1.2%] | 0.5% [0.4%-0.7%] |
| 2014 | 6.5% [1.7%-11.0%] | 8.4% [2.0%-14.5%] | 7.4% [1.9%-12.7%] | 0.7% [0.4%-1.1%] | 0.6% [0.3%-0.8%] | 0.7% [0.3%-1.0%] | 0.7% [-0.2%-1.4%] | 0.3% [-0.1%-0.5%] | 0.5% [-0.1%-1.0%] | 2.2% [0.9%-3.2%] | 2.2% [0.9%-3.3%] | 2.2% [0.9%-3.3%] | 1.0% [0.3%-1.7%] | 2.0% [0.5%-3.3%] | 1.5% [0.4%-2.4%] | 1.9% [-0.0%-3.8%] | 4.1% [-0.0%-8.4%] | 2.8% [-0.0%-5.8%] | 1.1% [-0.3%-2.2%] | 1.2% [-0.3%-2.4%] | 1.1% [-0.3%-2.3%] | 0.1% [0.1%-0.2%] | 0.2% [0.1%-0.3%] | 0.2% [0.1%-0.3%] | 0.6% [0.0%-2.9%] | 0.4% [0.0%-1.9%] | 0.5% [0.0%-2.4%] | 0.2% [0.1%-0.3%] | 0.9% [0.6%-1.2%] | 0.5% [0.4%-0.7%] |
| 2015 | 6.5% [1.7%-11.0%] | 8.4% [2.0%-14.6%] | 7.4% [1.9%-12.8%] | 0.7% [0.4%-1.0%] | 0.5% [0.3%-0.8%] | 0.6% [0.3%-0.9%] | 0.7% [-0.2%-1.4%] | 0.3% [-0.1%-0.5%] | 0.5% [-0.1%-1.0%] | 2.2% [0.9%-3.2%] | 2.2% [0.9%-3.3%] | 2.2% [0.9%-3.3%] | 1.0% [0.3%-1.7%] | 2.0% [0.5%-3.3%] | 1.5% [0.4%-2.5%] | 1.9% [-0.0%-3.8%] | 4.1% [-0.0%-8.4%] | 2.9% [-0.0%-5.9%] | 1.1% [-0.3%-2.3%] | 1.2% [-0.3%-2.4%] | 1.2% [-0.3%-2.3%] | 0.1% [0.1%-0.2%] | 0.2% [0.1%-0.3%] | 0.2% [0.1%-0.3%] | 0.6% [0.0%-2.8%] | 0.4% [0.0%-1.9%] | 0.5% [0.0%-2.4%] | 0.2% [0.1%-0.3%] | 0.9% [0.6%-1.2%] | 0.5% [0.4%-0.7%] |
| 2016 | 6.5% [1.7%-11.0%] | 8.4% [2.0%-14.5%] | 7.4% [1.9%-12.7%] | 0.7% [0.4%-1.0%] | 0.5% [0.3%-0.8%] | 0.6% [0.3%-0.9%] | 0.7% [-0.2%-1.4%] | 0.3% [-0.1%-0.5%] | 0.5% [-0.1%-1.0%] | 2.2% [0.9%-3.2%] | 2.2% [0.9%-3.3%] | 2.2% [0.9%-3.3%] | 1.0% [0.3%-1.7%] | 2.0% [0.5%-3.3%] | 1.5% [0.4%-2.5%] | 1.9% [-0.0%-3.8%] | 4.1% [-0.0%-8.4%] | 2.9% [-0.0%-5.9%] | 1.1% [-0.3%-2.3%] | 1.2% [-0.3%-2.4%] | 1.1% [-0.3%-2.3%] | 0.1% [0.1%-0.2%] | 0.2% [0.1%-0.3%] | 0.2% [0.1%-0.2%] | 0.5% [0.0%-2.7%] | 0.4% [0.0%-1.8%] | 0.5% [0.0%-2.3%] | 0.2% [0.1%-0.3%] | 0.9% [0.6%-1.2%] | 0.5% [0.4%-0.7%] |
| 2017 | 6.5% [1.7%-11.0%] | 8.3% [2.0%-14.5%] | 7.4% [1.9%-12.7%] | 0.7% [0.3%-1.0%] | 0.5% [0.3%-0.8%] | 0.6% [0.3%-0.9%] | 0.7% [-0.2%-1.4%] | 0.3% [-0.1%-0.5%] | 0.5% [-0.1%-1.0%] | 2.2% [0.9%-3.2%] | 2.2% [0.9%-3.3%] | 2.2% [0.9%-3.3%] | 1.0% [0.3%-1.7%] | 2.0% [0.5%-3.3%] | 1.5% [0.4%-2.5%] | 1.9% [-0.0%-3.8%] | 4.1% [-0.0%-8.4%] | 2.9% [-0.0%-5.9%] | 1.1% [-0.3%-2.3%] | 1.2% [-0.3%-2.4%] | 1.1% [-0.3%-2.3%] | 0.1% [0.1%-0.2%] | 0.2% [0.1%-0.3%] | 0.2% [0.1%-0.2%] | 0.5% [0.0%-2.7%] | 0.4% [0.0%-1.8%] | 0.5% [0.0%-2.3%] | 0.2% [0.1%-0.3%] | 0.9% [0.6%-1.2%] | 0.5% [0.4%-0.7%] |
| 2018 | 6.5% [1.7%-11.0%] | 8.3% [2.0%-14.5%] | 7.3% [1.9%-12.6%] | 0.7% [0.3%-1.0%] | 0.5% [0.3%-0.8%] | 0.6% [0.3%-0.9%] | 0.7% [-0.2%-1.4%] | 0.3% [-0.1%-0.5%] | 0.5% [-0.1%-1.0%] | 2.2% [0.9%-3.3%] | 2.2% [0.9%-3.2%] | 2.2% [0.9%-3.3%] | 1.0% [0.3%-1.7%] | 2.0% [0.5%-3.3%] | 1.5% [0.4%-2.5%] | 1.9% [-0.0%-3.8%] | 4.1% [-0.0%-8.4%] | 2.9% [-0.0%-5.9%] | 1.1% [-0.3%-2.3%] | 1.2% [-0.3%-2.3%] | 1.1% [-0.3%-2.3%] | 0.1% [0.1%-0.2%] | 0.2% [0.1%-0.3%] | 0.2% [0.1%-0.2%] | 0.5% [0.0%-2.7%] | 0.3% [0.0%-1.8%] | 0.5% [0.0%-2.3%] | 0.2% [0.1%-0.3%] | 0.9% [0.6%-1.1%] | 0.5% [0.4%-0.7%] |
| 2019 | 6.5% [1.6%-11.0%] | 8.3% [2.0%-14.4%] | 7.3% [1.8%-12.6%] | 0.7% [0.3%-1.0%] | 0.5% [0.3%-0.8%] | 0.6% [0.3%-0.9%] | 0.7% [-0.2%-1.4%] | 0.3% [-0.1%-0.5%] | 0.5% [-0.1%-1.0%] | 2.2% [0.9%-3.3%] | 2.2% [0.9%-3.2%] | 2.2% [0.9%-3.3%] | 1.0% [0.3%-1.7%] | 2.0% [0.5%-3.4%] | 1.5% [0.4%-2.5%] | 1.9% [-0.0%-3.8%] | 4.1% [-0.0%-8.4%] | 2.9% [-0.0%-5.9%] | 1.1% [-0.3%-2.3%] | 1.2% [-0.3%-2.3%] | 1.1% [-0.3%-2.3%] | 0.1% [0.1%-0.2%] | 0.2% [0.1%-0.3%] | 0.2% [0.1%-0.2%] | 0.5% [0.0%-2.7%] | 0.3% [0.0%-1.8%] | 0.4% [0.0%-2.3%] | 0.2% [0.1%-0.3%] | 0.9% [0.6%-1.1%] | 0.5% [0.4%-0.7%] |
| 2020 | 6.4% [1.6%-11.0%] | 8.3% [1.9%-14.4%] | 7.3% [1.8%-12.6%] | 0.7% [0.3%-1.0%] | 0.5% [0.3%-0.8%] | 0.6% [0.3%-0.9%] | 0.7% [-0.2%-1.4%] | 0.3% [-0.1%-0.5%] | 0.5% [-0.1%-1.0%] | 2.2% [0.9%-3.3%] | 2.2% [0.9%-3.2%] | 2.2% [0.9%-3.3%] | 1.0% [0.3%-1.7%] | 2.0% [0.5%-3.3%] | 1.5% [0.4%-2.5%] | 1.9% [-0.0%-3.9%] | 4.1% [-0.0%-8.3%] | 2.9% [-0.0%-5.8%] | 1.1% [-0.3%-2.3%] | 1.2% [-0.3%-2.3%] | 1.1% [-0.3%-2.3%] | 0.1% [0.1%-0.2%] | 0.2% [0.1%-0.3%] | 0.2% [0.1%-0.2%] | 0.5% [0.0%-2.6%] | 0.3% [0.0%-1.7%] | 0.4% [0.0%-2.3%] | 0.2% [0.1%-0.3%] | 0.9% [0.6%-1.1%] | 0.5% [0.4%-0.7%] |
| 2021 | 6.4% [1.6%-11.0%] | 8.3% [1.9%-14.3%] | 7.3% [1.8%-12.5%] | 0.7% [0.3%-1.0%] | 0.5% [0.3%-0.8%] | 0.6% [0.3%-0.9%] | 0.7% [-0.2%-1.4%] | 0.3% [-0.1%-0.5%] | 0.5% [-0.1%-1.1%] | 2.2% [0.9%-3.3%] | 2.2% [0.9%-3.2%] | 2.2% [0.9%-3.3%] | 1.0% [0.3%-1.8%] | 2.0% [0.5%-3.4%] | 1.5% [0.4%-2.5%] | 1.9% [-0.0%-3.9%] | 4.1% [-0.0%-8.3%] | 2.9% [-0.0%-5.8%] | 1.1% [-0.3%-2.2%] | 1.1% [-0.3%-2.3%] | 1.1% [-0.3%-2.3%] | 0.1% [0.1%-0.2%] | 0.2% [0.1%-0.3%] | 0.2% [0.1%-0.2%] | 0.5% [0.0%-2.6%] | 0.3% [0.0%-1.8%] | 0.4% [0.0%-2.2%] | 0.2% [0.1%-0.3%] | 0.9% [0.6%-1.1%] | 0.5% [0.4%-0.7%] |
| **Note:** Data are presented as percentages (%) with 95% confidence intervals [lower-upper]. Values of "-0.0%" and "0.0%" result from rounding to one decimal place. "-0.0%" indicates that the original value was negative but rounded to zero (absolute value <0.05%), while "0.0%" indicates a positive value that rounded to zero (<0.05%). Negative values suggest an inverse relationship between the dietary factor and cancer mortality, while positive values indicate a positive association. All rates are age-standardized. | | | | | | | | | | | | | | | | | | | | | | | | | | | | | | |

| Table S1c. Percentage of Total Cancer Mortality Attributable to Dietary Risk Factors - High-middle SDI Regions (Age-standardized, 1990-2021). | | | | | | | | | | | | | | | | | | | | | | | | | | | | | | |
| --- | --- | --- | --- | --- | --- | --- | --- | --- | --- | --- | --- | --- | --- | --- | --- | --- | --- | --- | --- | --- | --- | --- | --- | --- | --- | --- | --- | --- | --- | --- |
|  | All dietary risks | | | Low in fruits | | | Low in vegetables | | | Low in whole grains | | | Low in milk | | | High in red meat | | | High in processed meat | | | Low in fiber | | | High in sodium | | | Low in calcium | | |
| Year | Male | Female | Both | Male | Female | Both | Male | Female | Both | Male | Female | Both | Male | Female | Both | Male | Female | Both | Male | Female | Both | Male | Female | Both | Male | Female | Both | Male | Female | Both |
| 1990 | 7.6% [2.0%-15.8%] | 9.7% [2.6%-18.5%] | 8.5% [2.2%-17.0%] | 1.1% [0.6%-1.6%] | 0.5% [0.3%-0.8%] | 0.9% [0.5%-1.3%] | 1.6% [-0.3%-3.1%] | 0.9% [-0.2%-1.8%] | 1.3% [-0.3%-2.6%] | 1.7% [0.7%-2.6%] | 2.3% [1.0%-3.4%] | 1.9% [0.8%-2.9%] | 0.8% [0.3%-1.3%] | 2.1% [0.6%-3.4%] | 1.3% [0.4%-2.2%] | 1.4% [-0.0%-2.9%] | 3.8% [-0.0%-7.7%] | 2.4% [-0.0%-4.9%] | 0.6% [-0.1%-1.2%] | 0.9% [-0.2%-1.8%] | 0.7% [-0.2%-1.4%] | 0.1% [0.1%-0.2%] | 0.2% [0.1%-0.2%] | 0.1% [0.1%-0.2%] | 1.5% [-0.0%-7.7%] | 1.2% [-0.0%-6.2%] | 1.4% [-0.0%-7.1%] | 0.5% [0.4%-0.7%] | 1.3% [0.9%-1.6%] | 0.8% [0.6%-1.0%] |
| 1991 | 7.6% [2.0%-15.6%] | 9.7% [2.6%-18.3%] | 8.5% [2.2%-16.8%] | 1.1% [0.6%-1.6%] | 0.5% [0.3%-0.8%] | 0.9% [0.5%-1.3%] | 1.5% [-0.3%-3.1%] | 0.9% [-0.2%-1.8%] | 1.3% [-0.3%-2.5%] | 1.7% [0.7%-2.6%] | 2.3% [1.0%-3.4%] | 1.9% [0.8%-2.9%] | 0.8% [0.3%-1.3%] | 2.0% [0.6%-3.4%] | 1.3% [0.4%-2.2%] | 1.4% [-0.0%-2.9%] | 3.8% [-0.0%-7.8%] | 2.4% [-0.0%-4.8%] | 0.6% [-0.1%-1.1%] | 0.9% [-0.2%-1.8%] | 0.7% [-0.2%-1.4%] | 0.1% [0.0%-0.2%] | 0.2% [0.1%-0.2%] | 0.1% [0.1%-0.2%] | 1.5% [-0.0%-7.5%] | 1.2% [-0.0%-6.0%] | 1.4% [-0.0%-7.0%] | 0.5% [0.4%-0.6%] | 1.3% [0.9%-1.6%] | 0.8% [0.6%-1.0%] |
| 1992 | 7.5% [2.0%-15.4%] | 9.6% [2.6%-18.2%] | 8.4% [2.2%-16.7%] | 1.1% [0.6%-1.6%] | 0.5% [0.3%-0.8%] | 0.9% [0.5%-1.3%] | 1.5% [-0.3%-2.9%] | 0.8% [-0.2%-1.7%] | 1.2% [-0.3%-2.4%] | 1.7% [0.7%-2.6%] | 2.3% [1.0%-3.4%] | 2.0% [0.8%-2.9%] | 0.8% [0.3%-1.3%] | 2.0% [0.6%-3.4%] | 1.3% [0.4%-2.2%] | 1.5% [-0.0%-2.9%] | 3.8% [-0.0%-7.9%] | 2.4% [-0.0%-4.9%] | 0.6% [-0.1%-1.2%] | 0.9% [-0.2%-1.8%] | 0.7% [-0.2%-1.4%] | 0.1% [0.0%-0.2%] | 0.2% [0.1%-0.2%] | 0.1% [0.1%-0.2%] | 1.5% [-0.0%-7.4%] | 1.1% [-0.0%-5.8%] | 1.3% [-0.0%-6.9%] | 0.5% [0.4%-0.6%] | 1.3% [0.9%-1.6%] | 0.8% [0.6%-1.0%] |
| 1993 | 7.5% [2.0%-15.3%] | 9.6% [2.6%-18.1%] | 8.4% [2.2%-16.5%] | 1.1% [0.6%-1.6%] | 0.5% [0.3%-0.8%] | 0.9% [0.5%-1.3%] | 1.4% [-0.3%-2.9%] | 0.8% [-0.2%-1.6%] | 1.2% [-0.3%-2.4%] | 1.7% [0.7%-2.6%] | 2.3% [1.0%-3.4%] | 2.0% [0.8%-2.9%] | 0.8% [0.3%-1.3%] | 2.0% [0.6%-3.4%] | 1.3% [0.4%-2.2%] | 1.5% [-0.0%-3.0%] | 3.9% [-0.0%-7.9%] | 2.4% [-0.0%-4.9%] | 0.6% [-0.1%-1.2%] | 0.9% [-0.2%-1.9%] | 0.7% [-0.2%-1.5%] | 0.1% [0.0%-0.2%] | 0.2% [0.1%-0.2%] | 0.1% [0.1%-0.2%] | 1.4% [-0.0%-7.2%] | 1.1% [-0.0%-5.7%] | 1.3% [-0.0%-6.7%] | 0.5% [0.4%-0.6%] | 1.3% [0.9%-1.6%] | 0.8% [0.6%-1.0%] |
| 1994 | 7.4% [2.0%-14.9%] | 9.6% [2.6%-17.9%] | 8.3% [2.2%-16.3%] | 1.1% [0.6%-1.6%] | 0.5% [0.3%-0.8%] | 0.9% [0.4%-1.3%] | 1.4% [-0.3%-2.8%] | 0.8% [-0.1%-1.6%] | 1.1% [-0.2%-2.3%] | 1.8% [0.7%-2.6%] | 2.3% [1.0%-3.4%] | 2.0% [0.8%-2.9%] | 0.8% [0.3%-1.3%] | 2.0% [0.6%-3.3%] | 1.3% [0.4%-2.1%] | 1.5% [-0.0%-3.0%] | 3.9% [-0.0%-7.9%] | 2.4% [-0.0%-4.9%] | 0.6% [-0.1%-1.2%] | 0.9% [-0.2%-1.9%] | 0.7% [-0.2%-1.5%] | 0.1% [0.0%-0.2%] | 0.2% [0.1%-0.2%] | 0.1% [0.1%-0.2%] | 1.4% [-0.0%-7.0%] | 1.1% [-0.0%-5.6%] | 1.3% [-0.0%-6.5%] | 0.5% [0.4%-0.6%] | 1.3% [0.9%-1.6%] | 0.8% [0.6%-1.0%] |
| 1995 | 7.3% [2.0%-14.7%] | 9.5% [2.6%-17.8%] | 8.2% [2.2%-16.1%] | 1.1% [0.6%-1.6%] | 0.5% [0.3%-0.8%] | 0.9% [0.4%-1.3%] | 1.3% [-0.3%-2.7%] | 0.7% [-0.1%-1.5%] | 1.1% [-0.2%-2.2%] | 1.8% [0.8%-2.6%] | 2.3% [1.0%-3.4%] | 2.0% [0.8%-2.9%] | 0.8% [0.3%-1.3%] | 2.0% [0.6%-3.3%] | 1.3% [0.4%-2.1%] | 1.5% [-0.0%-3.0%] | 3.9% [-0.0%-8.0%] | 2.4% [-0.0%-5.0%] | 0.6% [-0.1%-1.2%] | 0.9% [-0.2%-1.8%] | 0.7% [-0.2%-1.4%] | 0.1% [0.0%-0.2%] | 0.2% [0.1%-0.2%] | 0.1% [0.1%-0.2%] | 1.4% [-0.0%-6.9%] | 1.1% [0.0%-5.4%] | 1.3% [-0.0%-6.4%] | 0.5% [0.4%-0.6%] | 1.3% [0.9%-1.6%] | 0.8% [0.6%-1.0%] |
| 1996 | 7.2% [2.0%-14.4%] | 9.4% [2.6%-17.6%] | 8.1% [2.2%-15.7%] | 1.0% [0.5%-1.5%] | 0.5% [0.3%-0.8%] | 0.9% [0.4%-1.2%] | 1.2% [-0.3%-2.5%] | 0.7% [-0.1%-1.4%] | 1.0% [-0.2%-2.1%] | 1.8% [0.8%-2.7%] | 2.3% [1.0%-3.4%] | 2.0% [0.8%-3.0%] | 0.8% [0.3%-1.3%] | 2.0% [0.6%-3.4%] | 1.3% [0.4%-2.1%] | 1.5% [-0.0%-3.0%] | 3.9% [-0.0%-8.0%] | 2.5% [-0.0%-5.0%] | 0.6% [-0.1%-1.2%] | 0.9% [-0.2%-1.8%] | 0.7% [-0.2%-1.4%] | 0.1% [0.0%-0.2%] | 0.2% [0.1%-0.2%] | 0.1% [0.1%-0.2%] | 1.4% [-0.0%-6.8%] | 1.0% [0.0%-5.3%] | 1.2% [-0.0%-6.3%] | 0.5% [0.3%-0.6%] | 1.3% [0.9%-1.6%] | 0.8% [0.6%-1.0%] |
| 1997 | 7.1% [2.0%-14.2%] | 9.4% [2.6%-17.4%] | 8.0% [2.2%-15.5%] | 1.0% [0.5%-1.5%] | 0.5% [0.3%-0.8%] | 0.8% [0.4%-1.2%] | 1.2% [-0.2%-2.4%] | 0.6% [-0.1%-1.3%] | 1.0% [-0.2%-2.0%] | 1.8% [0.8%-2.7%] | 2.3% [1.0%-3.4%] | 2.0% [0.8%-3.0%] | 0.8% [0.3%-1.3%] | 2.0% [0.6%-3.4%] | 1.3% [0.4%-2.2%] | 1.5% [-0.0%-3.0%] | 3.9% [-0.0%-8.0%] | 2.5% [-0.0%-5.0%] | 0.6% [-0.1%-1.2%] | 0.9% [-0.2%-1.8%] | 0.7% [-0.2%-1.4%] | 0.1% [0.0%-0.2%] | 0.2% [0.1%-0.2%] | 0.1% [0.1%-0.2%] | 1.3% [-0.0%-6.7%] | 1.0% [0.0%-5.2%] | 1.2% [-0.0%-6.2%] | 0.5% [0.3%-0.6%] | 1.3% [0.9%-1.6%] | 0.8% [0.6%-1.0%] |
| 1998 | 7.0% [2.0%-14.0%] | 9.3% [2.6%-17.3%] | 8.0% [2.2%-15.4%] | 1.0% [0.5%-1.5%] | 0.5% [0.3%-0.8%] | 0.8% [0.4%-1.2%] | 1.1% [-0.2%-2.3%] | 0.6% [-0.1%-1.2%] | 0.9% [-0.2%-1.9%] | 1.8% [0.8%-2.7%] | 2.3% [1.0%-3.4%] | 2.0% [0.8%-3.0%] | 0.8% [0.3%-1.3%] | 2.0% [0.6%-3.4%] | 1.3% [0.4%-2.2%] | 1.5% [-0.0%-3.0%] | 3.9% [-0.0%-8.1%] | 2.5% [-0.0%-5.0%] | 0.6% [-0.1%-1.2%] | 0.9% [-0.2%-1.8%] | 0.7% [-0.2%-1.4%] | 0.1% [0.0%-0.2%] | 0.2% [0.1%-0.2%] | 0.1% [0.1%-0.2%] | 1.3% [-0.0%-6.6%] | 1.0% [0.0%-5.2%] | 1.2% [-0.0%-6.1%] | 0.4% [0.3%-0.6%] | 1.3% [0.9%-1.6%] | 0.8% [0.6%-1.0%] |
| 1999 | 6.9% [2.0%-13.8%] | 9.3% [2.6%-17.2%] | 7.9% [2.2%-15.2%] | 1.0% [0.5%-1.4%] | 0.5% [0.3%-0.8%] | 0.8% [0.4%-1.2%] | 1.0% [-0.2%-2.2%] | 0.5% [-0.1%-1.2%] | 0.8% [-0.2%-1.8%] | 1.8% [0.8%-2.7%] | 2.3% [1.0%-3.4%] | 2.0% [0.8%-3.0%] | 0.8% [0.3%-1.3%] | 2.0% [0.6%-3.4%] | 1.3% [0.4%-2.2%] | 1.5% [-0.0%-3.1%] | 3.9% [-0.0%-8.0%] | 2.5% [-0.0%-5.0%] | 0.6% [-0.1%-1.2%] | 0.9% [-0.2%-1.8%] | 0.7% [-0.2%-1.5%] | 0.1% [0.1%-0.2%] | 0.2% [0.1%-0.2%] | 0.1% [0.1%-0.2%] | 1.3% [-0.0%-6.6%] | 1.0% [0.0%-5.1%] | 1.2% [-0.0%-6.0%] | 0.4% [0.3%-0.6%] | 1.3% [0.9%-1.6%] | 0.8% [0.6%-1.0%] |
| 2000 | 6.8% [2.0%-13.7%] | 9.2% [2.6%-17.0%] | 7.8% [2.2%-15.1%] | 1.0% [0.5%-1.4%] | 0.5% [0.3%-0.8%] | 0.8% [0.4%-1.2%] | 0.9% [-0.2%-2.1%] | 0.5% [-0.1%-1.1%] | 0.8% [-0.1%-1.7%] | 1.8% [0.8%-2.7%] | 2.3% [1.0%-3.4%] | 2.0% [0.8%-3.0%] | 0.8% [0.3%-1.3%] | 2.0% [0.6%-3.4%] | 1.3% [0.4%-2.2%] | 1.5% [-0.0%-3.1%] | 3.9% [-0.0%-8.0%] | 2.5% [-0.0%-5.0%] | 0.6% [-0.1%-1.2%] | 0.9% [-0.2%-1.8%] | 0.7% [-0.2%-1.4%] | 0.1% [0.1%-0.2%] | 0.2% [0.1%-0.2%] | 0.1% [0.1%-0.2%] | 1.3% [-0.0%-6.6%] | 1.0% [0.0%-5.1%] | 1.2% [-0.0%-6.0%] | 0.4% [0.3%-0.6%] | 1.2% [0.9%-1.6%] | 0.8% [0.6%-1.0%] |
| 2001 | 6.8% [2.0%-13.5%] | 9.1% [2.6%-16.9%] | 7.7% [2.2%-15.0%] | 0.9% [0.5%-1.4%] | 0.5% [0.3%-0.8%] | 0.8% [0.4%-1.1%] | 0.9% [-0.2%-1.9%] | 0.5% [-0.1%-1.0%] | 0.7% [-0.1%-1.6%] | 1.8% [0.8%-2.7%] | 2.3% [1.0%-3.4%] | 2.0% [0.9%-3.0%] | 0.8% [0.3%-1.3%] | 2.0% [0.6%-3.4%] | 1.3% [0.4%-2.2%] | 1.5% [-0.0%-3.1%] | 3.9% [-0.0%-8.0%] | 2.5% [-0.0%-5.0%] | 0.6% [-0.1%-1.2%] | 0.9% [-0.2%-1.8%] | 0.7% [-0.2%-1.4%] | 0.1% [0.1%-0.2%] | 0.2% [0.1%-0.2%] | 0.1% [0.1%-0.2%] | 1.3% [-0.0%-6.6%] | 1.0% [0.0%-5.0%] | 1.2% [-0.0%-6.0%] | 0.4% [0.3%-0.6%] | 1.2% [0.9%-1.5%] | 0.8% [0.6%-1.0%] |
| 2002 | 6.7% [2.0%-13.4%] | 9.0% [2.6%-16.7%] | 7.7% [2.2%-14.8%] | 0.9% [0.5%-1.3%] | 0.5% [0.3%-0.8%] | 0.8% [0.4%-1.1%] | 0.8% [-0.2%-1.8%] | 0.4% [-0.1%-0.9%] | 0.7% [-0.1%-1.4%] | 1.8% [0.8%-2.7%] | 2.3% [1.0%-3.4%] | 2.0% [0.9%-3.0%] | 0.8% [0.3%-1.3%] | 2.0% [0.6%-3.4%] | 1.3% [0.4%-2.2%] | 1.6% [-0.0%-3.1%] | 3.9% [-0.0%-7.9%] | 2.5% [-0.0%-5.0%] | 0.6% [-0.1%-1.2%] | 0.9% [-0.2%-1.8%] | 0.7% [-0.2%-1.5%] | 0.1% [0.1%-0.2%] | 0.2% [0.1%-0.2%] | 0.1% [0.1%-0.2%] | 1.3% [-0.0%-6.5%] | 1.0% [0.0%-5.0%] | 1.2% [-0.0%-5.9%] | 0.4% [0.3%-0.6%] | 1.2% [0.9%-1.5%] | 0.8% [0.6%-1.0%] |
| 2003 | 6.7% [2.0%-13.3%] | 9.0% [2.6%-16.6%] | 7.6% [2.2%-14.7%] | 0.9% [0.5%-1.3%] | 0.5% [0.3%-0.7%] | 0.8% [0.4%-1.1%] | 0.8% [-0.1%-1.7%] | 0.4% [-0.1%-0.9%] | 0.6% [-0.1%-1.4%] | 1.9% [0.8%-2.8%] | 2.3% [1.0%-3.4%] | 2.0% [0.9%-3.0%] | 0.9% [0.3%-1.3%] | 2.0% [0.6%-3.4%] | 1.3% [0.4%-2.2%] | 1.6% [-0.0%-3.1%] | 3.9% [-0.0%-8.0%] | 2.5% [-0.0%-5.0%] | 0.6% [-0.1%-1.2%] | 0.9% [-0.2%-1.8%] | 0.7% [-0.2%-1.4%] | 0.1% [0.1%-0.2%] | 0.2% [0.1%-0.2%] | 0.1% [0.1%-0.2%] | 1.3% [-0.0%-6.5%] | 1.0% [0.0%-4.9%] | 1.2% [-0.0%-5.9%] | 0.4% [0.3%-0.6%] | 1.2% [0.9%-1.5%] | 0.8% [0.6%-0.9%] |
| 2004 | 6.6% [2.0%-13.2%] | 8.9% [2.5%-16.5%] | 7.5% [2.2%-14.6%] | 0.9% [0.5%-1.3%] | 0.5% [0.3%-0.7%] | 0.7% [0.4%-1.1%] | 0.7% [-0.1%-1.6%] | 0.4% [-0.1%-0.8%] | 0.6% [-0.1%-1.3%] | 1.9% [0.8%-2.8%] | 2.3% [1.0%-3.4%] | 2.0% [0.9%-3.0%] | 0.9% [0.3%-1.4%] | 2.0% [0.6%-3.3%] | 1.4% [0.4%-2.2%] | 1.6% [-0.0%-3.2%] | 3.9% [-0.0%-7.9%] | 2.5% [-0.0%-5.0%] | 0.6% [-0.1%-1.2%] | 0.9% [-0.2%-1.8%] | 0.7% [-0.2%-1.4%] | 0.1% [0.0%-0.2%] | 0.1% [0.1%-0.2%] | 0.1% [0.1%-0.2%] | 1.3% [-0.0%-6.6%] | 1.0% [0.0%-4.9%] | 1.2% [-0.0%-6.0%] | 0.4% [0.3%-0.6%] | 1.2% [0.9%-1.5%] | 0.8% [0.6%-0.9%] |
| 2005 | 6.5% [2.0%-13.1%] | 8.8% [2.5%-16.4%] | 7.5% [2.2%-14.4%] | 0.8% [0.4%-1.2%] | 0.5% [0.3%-0.7%] | 0.7% [0.4%-1.0%] | 0.7% [-0.1%-1.5%] | 0.3% [-0.1%-0.7%] | 0.5% [-0.1%-1.2%] | 1.9% [0.8%-2.8%] | 2.3% [1.0%-3.4%] | 2.1% [0.9%-3.0%] | 0.9% [0.3%-1.4%] | 2.0% [0.6%-3.3%] | 1.4% [0.4%-2.2%] | 1.6% [-0.0%-3.2%] | 3.9% [-0.0%-7.9%] | 2.5% [-0.0%-5.1%] | 0.6% [-0.2%-1.2%] | 0.9% [-0.2%-1.8%] | 0.7% [-0.2%-1.4%] | 0.1% [0.0%-0.2%] | 0.1% [0.1%-0.2%] | 0.1% [0.1%-0.2%] | 1.3% [-0.0%-6.5%] | 0.9% [0.0%-4.8%] | 1.2% [-0.0%-5.8%] | 0.4% [0.3%-0.6%] | 1.2% [0.9%-1.5%] | 0.8% [0.6%-0.9%] |
| 2006 | 6.4% [2.0%-12.8%] | 8.8% [2.5%-16.2%] | 7.4% [2.2%-14.2%] | 0.8% [0.4%-1.2%] | 0.5% [0.2%-0.7%] | 0.7% [0.4%-1.0%] | 0.6% [-0.1%-1.4%] | 0.3% [-0.1%-0.7%] | 0.5% [-0.1%-1.1%] | 1.9% [0.8%-2.9%] | 2.3% [1.0%-3.4%] | 2.1% [0.9%-3.1%] | 0.9% [0.3%-1.4%] | 2.0% [0.6%-3.3%] | 1.4% [0.4%-2.2%] | 1.6% [-0.0%-3.2%] | 3.9% [-0.0%-8.0%] | 2.5% [-0.0%-5.1%] | 0.6% [-0.1%-1.2%] | 0.9% [-0.2%-1.8%] | 0.7% [-0.2%-1.5%] | 0.1% [0.0%-0.2%] | 0.1% [0.1%-0.2%] | 0.1% [0.1%-0.2%] | 1.2% [-0.0%-6.3%] | 0.9% [0.0%-4.6%] | 1.1% [-0.0%-5.7%] | 0.4% [0.3%-0.6%] | 1.2% [0.9%-1.5%] | 0.7% [0.6%-0.9%] |
| 2007 | 6.3% [2.0%-12.6%] | 8.7% [2.5%-16.0%] | 7.3% [2.2%-14.0%] | 0.8% [0.4%-1.1%] | 0.5% [0.2%-0.7%] | 0.7% [0.3%-1.0%] | 0.6% [-0.1%-1.3%] | 0.3% [-0.0%-0.6%] | 0.4% [-0.1%-1.0%] | 1.9% [0.8%-2.9%] | 2.3% [1.0%-3.4%] | 2.1% [0.9%-3.1%] | 0.9% [0.3%-1.4%] | 2.0% [0.6%-3.4%] | 1.4% [0.4%-2.2%] | 1.6% [-0.0%-3.3%] | 3.9% [-0.0%-7.9%] | 2.5% [-0.0%-5.1%] | 0.6% [-0.1%-1.2%] | 0.9% [-0.2%-1.8%] | 0.7% [-0.2%-1.5%] | 0.1% [0.0%-0.2%] | 0.1% [0.1%-0.2%] | 0.1% [0.1%-0.2%] | 1.2% [-0.0%-6.1%] | 0.9% [0.0%-4.5%] | 1.1% [-0.0%-5.5%] | 0.4% [0.3%-0.6%] | 1.2% [0.9%-1.5%] | 0.7% [0.6%-0.9%] |
| 2008 | 6.3% [2.0%-12.4%] | 8.6% [2.5%-15.9%] | 7.3% [2.2%-13.9%] | 0.8% [0.4%-1.1%] | 0.4% [0.2%-0.7%] | 0.6% [0.3%-0.9%] | 0.5% [-0.1%-1.2%] | 0.2% [-0.0%-0.6%] | 0.4% [-0.1%-0.9%] | 2.0% [0.8%-2.9%] | 2.3% [1.0%-3.4%] | 2.1% [0.9%-3.1%] | 0.9% [0.3%-1.4%] | 2.1% [0.6%-3.4%] | 1.4% [0.4%-2.3%] | 1.7% [-0.0%-3.3%] | 3.9% [-0.0%-8.0%] | 2.6% [-0.0%-5.2%] | 0.6% [-0.1%-1.3%] | 0.9% [-0.2%-1.8%] | 0.7% [-0.2%-1.5%] | 0.1% [0.0%-0.2%] | 0.1% [0.1%-0.2%] | 0.1% [0.1%-0.2%] | 1.2% [-0.0%-6.0%] | 0.8% [0.0%-4.3%] | 1.1% [-0.0%-5.4%] | 0.4% [0.3%-0.5%] | 1.2% [0.8%-1.5%] | 0.7% [0.6%-0.9%] |
| 2009 | 6.2% [1.9%-12.3%] | 8.5% [2.5%-15.7%] | 7.2% [2.1%-13.7%] | 0.7% [0.4%-1.1%] | 0.4% [0.2%-0.6%] | 0.6% [0.3%-0.9%] | 0.5% [-0.1%-1.1%] | 0.2% [-0.0%-0.5%] | 0.4% [-0.1%-0.9%] | 2.0% [0.8%-2.9%] | 2.3% [1.0%-3.4%] | 2.1% [0.9%-3.1%] | 0.9% [0.3%-1.5%] | 2.1% [0.6%-3.4%] | 1.4% [0.4%-2.3%] | 1.7% [-0.0%-3.3%] | 3.9% [-0.0%-7.9%] | 2.5% [-0.0%-5.2%] | 0.6% [-0.1%-1.3%] | 0.9% [-0.2%-1.8%] | 0.7% [-0.2%-1.5%] | 0.1% [0.0%-0.2%] | 0.1% [0.1%-0.2%] | 0.1% [0.1%-0.2%] | 1.2% [-0.0%-5.9%] | 0.8% [0.0%-4.3%] | 1.0% [-0.0%-5.3%] | 0.4% [0.3%-0.5%] | 1.1% [0.8%-1.5%] | 0.7% [0.5%-0.9%] |
| 2010 | 6.2% [1.9%-12.3%] | 8.5% [2.5%-15.5%] | 7.1% [2.1%-13.6%] | 0.7% [0.4%-1.1%] | 0.4% [0.2%-0.6%] | 0.6% [0.3%-0.9%] | 0.5% [-0.1%-1.1%] | 0.2% [-0.0%-0.5%] | 0.4% [-0.1%-0.8%] | 2.0% [0.8%-2.9%] | 2.3% [1.0%-3.4%] | 2.1% [0.9%-3.1%] | 1.0% [0.3%-1.5%] | 2.1% [0.6%-3.4%] | 1.4% [0.4%-2.3%] | 1.7% [-0.0%-3.4%] | 3.9% [-0.0%-8.0%] | 2.6% [-0.0%-5.2%] | 0.6% [-0.1%-1.3%] | 0.9% [-0.2%-1.7%] | 0.7% [-0.2%-1.5%] | 0.1% [0.0%-0.2%] | 0.1% [0.1%-0.2%] | 0.1% [0.1%-0.2%] | 1.1% [-0.0%-5.8%] | 0.8% [0.0%-4.1%] | 1.0% [-0.0%-5.2%] | 0.4% [0.3%-0.5%] | 1.1% [0.8%-1.4%] | 0.7% [0.5%-0.9%] |
| 2011 | 6.1% [1.9%-12.2%] | 8.5% [2.5%-15.4%] | 7.1% [2.1%-13.5%] | 0.7% [0.4%-1.0%] | 0.4% [0.2%-0.6%] | 0.6% [0.3%-0.9%] | 0.4% [-0.1%-1.0%] | 0.2% [-0.0%-0.4%] | 0.3% [-0.1%-0.8%] | 2.0% [0.8%-3.0%] | 2.3% [1.0%-3.4%] | 2.1% [0.9%-3.1%] | 1.0% [0.4%-1.5%] | 2.1% [0.6%-3.4%] | 1.4% [0.4%-2.3%] | 1.7% [-0.0%-3.4%] | 3.9% [-0.0%-8.0%] | 2.6% [-0.0%-5.2%] | 0.6% [-0.1%-1.3%] | 0.8% [-0.2%-1.7%] | 0.7% [-0.2%-1.4%] | 0.1% [0.0%-0.2%] | 0.1% [0.1%-0.2%] | 0.1% [0.1%-0.2%] | 1.1% [-0.0%-5.7%] | 0.8% [0.0%-4.0%] | 1.0% [-0.0%-5.0%] | 0.4% [0.3%-0.5%] | 1.1% [0.8%-1.4%] | 0.7% [0.5%-0.9%] |
| 2012 | 6.1% [1.9%-12.1%] | 8.4% [2.4%-15.3%] | 7.0% [2.1%-13.3%] | 0.7% [0.4%-1.0%] | 0.4% [0.2%-0.6%] | 0.6% [0.3%-0.8%] | 0.4% [-0.1%-0.9%] | 0.2% [-0.0%-0.4%] | 0.3% [-0.1%-0.7%] | 2.0% [0.8%-3.0%] | 2.3% [1.0%-3.4%] | 2.1% [0.9%-3.1%] | 1.0% [0.4%-1.6%] | 2.1% [0.6%-3.4%] | 1.5% [0.4%-2.3%] | 1.7% [-0.0%-3.4%] | 3.9% [-0.0%-8.0%] | 2.6% [-0.0%-5.2%] | 0.6% [-0.1%-1.2%] | 0.8% [-0.2%-1.7%] | 0.7% [-0.2%-1.4%] | 0.1% [0.0%-0.2%] | 0.1% [0.1%-0.2%] | 0.1% [0.1%-0.2%] | 1.1% [-0.0%-5.6%] | 0.8% [0.0%-3.9%] | 1.0% [-0.0%-5.0%] | 0.4% [0.3%-0.5%] | 1.1% [0.8%-1.4%] | 0.7% [0.5%-0.9%] |
| 2013 | 6.1% [1.9%-12.0%] | 8.4% [2.4%-15.2%] | 7.0% [2.1%-13.2%] | 0.7% [0.4%-1.0%] | 0.4% [0.2%-0.6%] | 0.6% [0.3%-0.8%] | 0.4% [-0.1%-0.9%] | 0.2% [-0.0%-0.4%] | 0.3% [-0.1%-0.7%] | 2.0% [0.8%-3.0%] | 2.3% [1.0%-3.4%] | 2.1% [0.9%-3.2%] | 1.0% [0.4%-1.6%] | 2.1% [0.6%-3.5%] | 1.5% [0.5%-2.4%] | 1.7% [-0.0%-3.4%] | 3.9% [-0.0%-7.9%] | 2.6% [-0.0%-5.2%] | 0.6% [-0.1%-1.3%] | 0.8% [-0.2%-1.7%] | 0.7% [-0.2%-1.4%] | 0.1% [0.0%-0.2%] | 0.1% [0.1%-0.2%] | 0.1% [0.0%-0.2%] | 1.1% [-0.0%-5.6%] | 0.7% [0.0%-3.8%] | 1.0% [-0.0%-4.9%] | 0.4% [0.3%-0.5%] | 1.1% [0.8%-1.4%] | 0.7% [0.5%-0.9%] |
| 2014 | 6.1% [1.9%-11.9%] | 8.3% [2.4%-15.0%] | 7.0% [2.1%-13.1%] | 0.7% [0.3%-1.0%] | 0.4% [0.2%-0.6%] | 0.6% [0.3%-0.8%] | 0.4% [-0.1%-0.8%] | 0.2% [-0.0%-0.4%] | 0.3% [-0.1%-0.6%] | 2.0% [0.9%-3.0%] | 2.3% [1.0%-3.4%] | 2.1% [0.9%-3.2%] | 1.0% [0.4%-1.7%] | 2.1% [0.6%-3.5%] | 1.5% [0.5%-2.4%] | 1.7% [-0.0%-3.5%] | 3.9% [-0.0%-8.0%] | 2.6% [-0.0%-5.2%] | 0.6% [-0.1%-1.3%] | 0.8% [-0.2%-1.7%] | 0.7% [-0.2%-1.5%] | 0.1% [0.0%-0.2%] | 0.1% [0.1%-0.2%] | 0.1% [0.0%-0.2%] | 1.1% [-0.0%-5.4%] | 0.7% [-0.0%-3.7%] | 0.9% [-0.0%-4.8%] | 0.4% [0.3%-0.5%] | 1.1% [0.8%-1.4%] | 0.7% [0.5%-0.9%] |
| 2015 | 6.0% [1.9%-11.8%] | 8.3% [2.4%-14.9%] | 7.0% [2.1%-13.1%] | 0.7% [0.3%-0.9%] | 0.4% [0.2%-0.6%] | 0.6% [0.3%-0.8%] | 0.4% [-0.1%-0.8%] | 0.2% [-0.0%-0.4%] | 0.3% [-0.1%-0.6%] | 2.1% [0.9%-3.0%] | 2.3% [1.0%-3.4%] | 2.1% [0.9%-3.2%] | 1.1% [0.4%-1.7%] | 2.2% [0.6%-3.5%] | 1.5% [0.5%-2.4%] | 1.8% [-0.0%-3.5%] | 3.9% [-0.0%-8.0%] | 2.6% [-0.0%-5.2%] | 0.6% [-0.1%-1.3%] | 0.8% [-0.2%-1.7%] | 0.7% [-0.2%-1.4%] | 0.1% [0.0%-0.2%] | 0.1% [0.1%-0.2%] | 0.1% [0.0%-0.2%] | 1.1% [-0.0%-5.3%] | 0.7% [0.0%-3.7%] | 0.9% [-0.0%-4.7%] | 0.4% [0.3%-0.5%] | 1.1% [0.8%-1.4%] | 0.7% [0.5%-0.9%] |
| 2016 | 6.0% [1.9%-11.7%] | 8.3% [2.4%-14.9%] | 6.9% [2.1%-13.0%] | 0.6% [0.3%-0.9%] | 0.4% [0.2%-0.6%] | 0.5% [0.3%-0.8%] | 0.3% [-0.1%-0.8%] | 0.2% [-0.0%-0.4%] | 0.3% [-0.1%-0.6%] | 2.1% [0.9%-3.1%] | 2.3% [1.0%-3.4%] | 2.2% [0.9%-3.2%] | 1.1% [0.4%-1.7%] | 2.2% [0.6%-3.6%] | 1.5% [0.5%-2.4%] | 1.8% [-0.0%-3.5%] | 3.9% [-0.0%-8.0%] | 2.6% [-0.0%-5.3%] | 0.6% [-0.1%-1.3%] | 0.8% [-0.2%-1.7%] | 0.7% [-0.2%-1.4%] | 0.1% [0.0%-0.2%] | 0.1% [0.1%-0.2%] | 0.1% [0.0%-0.2%] | 1.0% [-0.0%-5.2%] | 0.7% [0.0%-3.6%] | 0.9% [-0.0%-4.6%] | 0.4% [0.3%-0.5%] | 1.1% [0.8%-1.4%] | 0.7% [0.5%-0.9%] |
| 2017 | 6.0% [1.9%-11.7%] | 8.2% [2.4%-14.7%] | 6.9% [2.1%-12.9%] | 0.6% [0.3%-0.9%] | 0.4% [0.2%-0.6%] | 0.5% [0.3%-0.8%] | 0.3% [-0.1%-0.7%] | 0.1% [-0.0%-0.3%] | 0.3% [-0.1%-0.6%] | 2.1% [0.9%-3.1%] | 2.3% [0.9%-3.4%] | 2.2% [0.9%-3.2%] | 1.1% [0.4%-1.7%] | 2.2% [0.6%-3.6%] | 1.6% [0.5%-2.5%] | 1.8% [-0.0%-3.5%] | 3.9% [-0.0%-8.0%] | 2.6% [-0.0%-5.3%] | 0.6% [-0.2%-1.3%] | 0.8% [-0.2%-1.7%] | 0.7% [-0.2%-1.4%] | 0.1% [0.0%-0.2%] | 0.1% [0.1%-0.2%] | 0.1% [0.0%-0.2%] | 1.0% [-0.0%-5.1%] | 0.7% [0.0%-3.6%] | 0.9% [-0.0%-4.5%] | 0.4% [0.3%-0.5%] | 1.1% [0.8%-1.4%] | 0.7% [0.5%-0.9%] |
| 2018 | 6.0% [1.9%-11.6%] | 8.2% [2.4%-14.7%] | 6.9% [2.1%-12.8%] | 0.6% [0.3%-0.9%] | 0.4% [0.2%-0.6%] | 0.5% [0.3%-0.8%] | 0.3% [-0.1%-0.7%] | 0.1% [-0.0%-0.3%] | 0.3% [-0.1%-0.6%] | 2.1% [0.9%-3.1%] | 2.3% [0.9%-3.3%] | 2.2% [0.9%-3.2%] | 1.1% [0.4%-1.8%] | 2.2% [0.6%-3.6%] | 1.6% [0.5%-2.5%] | 1.8% [-0.0%-3.5%] | 3.8% [-0.0%-7.9%] | 2.6% [-0.0%-5.2%] | 0.6% [-0.2%-1.3%] | 0.8% [-0.2%-1.6%] | 0.7% [-0.2%-1.4%] | 0.1% [0.0%-0.2%] | 0.1% [0.1%-0.2%] | 0.1% [0.0%-0.2%] | 1.0% [-0.0%-5.1%] | 0.7% [0.0%-3.5%] | 0.9% [-0.0%-4.5%] | 0.4% [0.3%-0.5%] | 1.1% [0.8%-1.4%] | 0.7% [0.5%-0.8%] |
| 2019 | 6.0% [1.9%-11.5%] | 8.1% [2.4%-14.6%] | 6.9% [2.1%-12.8%] | 0.6% [0.3%-0.9%] | 0.4% [0.2%-0.6%] | 0.5% [0.3%-0.8%] | 0.3% [-0.1%-0.7%] | 0.1% [-0.0%-0.3%] | 0.3% [-0.1%-0.6%] | 2.1% [0.9%-3.1%] | 2.3% [0.9%-3.3%] | 2.2% [0.9%-3.2%] | 1.1% [0.4%-1.8%] | 2.2% [0.6%-3.6%] | 1.6% [0.5%-2.5%] | 1.8% [-0.0%-3.6%] | 3.8% [-0.0%-7.8%] | 2.6% [-0.0%-5.3%] | 0.6% [-0.1%-1.2%] | 0.8% [-0.2%-1.6%] | 0.7% [-0.2%-1.4%] | 0.1% [0.0%-0.2%] | 0.1% [0.1%-0.2%] | 0.1% [0.0%-0.2%] | 1.0% [-0.0%-5.1%] | 0.7% [0.0%-3.5%] | 0.9% [-0.0%-4.6%] | 0.4% [0.3%-0.5%] | 1.1% [0.8%-1.4%] | 0.7% [0.5%-0.9%] |
| 2020 | 6.0% [1.9%-11.5%] | 8.1% [2.4%-14.5%] | 6.9% [2.1%-12.8%] | 0.6% [0.3%-0.9%] | 0.4% [0.2%-0.6%] | 0.5% [0.3%-0.8%] | 0.3% [-0.1%-0.7%] | 0.1% [-0.0%-0.3%] | 0.3% [-0.1%-0.6%] | 2.1% [0.9%-3.1%] | 2.2% [0.9%-3.4%] | 2.2% [0.9%-3.2%] | 1.2% [0.4%-1.8%] | 2.2% [0.6%-3.7%] | 1.6% [0.5%-2.6%] | 1.8% [-0.0%-3.6%] | 3.8% [-0.0%-7.9%] | 2.6% [-0.0%-5.3%] | 0.6% [-0.1%-1.3%] | 0.8% [-0.2%-1.6%] | 0.7% [-0.2%-1.4%] | 0.1% [0.0%-0.2%] | 0.1% [0.1%-0.2%] | 0.1% [0.0%-0.2%] | 1.0% [-0.0%-5.0%] | 0.7% [0.0%-3.5%] | 0.9% [-0.0%-4.5%] | 0.4% [0.2%-0.5%] | 1.1% [0.8%-1.4%] | 0.7% [0.5%-0.8%] |
| 2021 | 6.0% [1.9%-11.5%] | 8.1% [2.4%-14.4%] | 6.9% [2.1%-12.7%] | 0.6% [0.3%-0.9%] | 0.4% [0.2%-0.6%] | 0.5% [0.3%-0.8%] | 0.3% [-0.1%-0.8%] | 0.1% [-0.0%-0.3%] | 0.3% [-0.1%-0.6%] | 2.1% [0.9%-3.1%] | 2.2% [0.9%-3.3%] | 2.2% [0.9%-3.2%] | 1.2% [0.4%-1.9%] | 2.2% [0.6%-3.7%] | 1.6% [0.5%-2.6%] | 1.8% [-0.0%-3.5%] | 3.8% [-0.0%-7.7%] | 2.6% [-0.0%-5.2%] | 0.6% [-0.1%-1.2%] | 0.8% [-0.2%-1.6%] | 0.7% [-0.2%-1.4%] | 0.1% [0.0%-0.2%] | 0.1% [0.1%-0.2%] | 0.1% [0.0%-0.2%] | 1.0% [-0.0%-5.0%] | 0.7% [0.0%-3.5%] | 0.9% [-0.0%-4.4%] | 0.4% [0.3%-0.5%] | 1.1% [0.8%-1.4%] | 0.7% [0.5%-0.9%] |
| **Note:** Data are presented as percentages (%) with 95% confidence intervals [lower-upper]. Values of "-0.0%" and "0.0%" result from rounding to one decimal place. "-0.0%" indicates that the original value was negative but rounded to zero (absolute value <0.05%), while "0.0%" indicates a positive value that rounded to zero (<0.05%). Negative values suggest an inverse relationship between the dietary factor and cancer mortality, while positive values indicate a positive association. All rates are age-standardized. | | | | | | | | | | | | | | | | | | | | | | | | | | | | | | |

| Table S1d:Percentage of Total Cancer Mortality Attributable to Dietary Risk Factors - Middle SDI Regions (Age-standardized, 1990-2021). | | | | | | | | | | | | | | | | | | | | | | | | | | | | | | |  |
| --- | --- | --- | --- | --- | --- | --- | --- | --- | --- | --- | --- | --- | --- | --- | --- | --- | --- | --- | --- | --- | --- | --- | --- | --- | --- | --- | --- | --- | --- | --- | --- |
|  | All dietary risks | | | Low in fruits | | | Low in vegetables | | | Low in whole grains | | | Low in milk | | | High in red meat | | | High in processed meat | | | Low in fiber | | | High in sodium | | | Low in calcium | | |  |
| Year | Male | Female | Both | Male | Female | Both | Male | Female | Both | Male | Female | Both | Male | Female | Both | Male | Female | Both | Male | Female | Both | Male | Female | Both | Male | Female | Both | Male | Female | Both |  |
| 1990 | 8.4% [2.3%-17.3%] | 9.1% [2.5%-17.8%] | 8.8% [2.4%-17.5%] | 1.3% [0.6%-1.9%] | 0.7% [0.4%-1.1%] | 1.1% [0.5%-1.5%] | 2.6% [-0.6%-5.2%] | 1.8% [-0.3%-3.6%] | 2.3% [-0.5%-4.5%] | 1.3% [0.5%-1.9%] | 1.6% [0.6%-2.4%] | 1.4% [0.6%-2.1%] | 0.8% [0.5%-1.1%] | 2.0% [0.6%-3.2%] | 1.3% [0.5%-2.0%] | 1.0% [-0.0%-2.0%] | 2.5% [-0.0%-5.1%] | 1.6% [-0.0%-3.4%] | 0.1% [-0.0%-0.2%] | 0.2% [-0.0%-0.3%] | 0.1% [-0.0%-0.3%] | 0.2% [0.1%-0.3%] | 0.2% [0.1%-0.4%] | 0.2% [0.1%-0.3%] | 1.9% [-0.0%-9.4%] | 1.4% [-0.0%-7.2%] | 1.7% [-0.0%-8.4%] | 1.2% [1.0%-1.4%] | 1.9% [1.4%-2.3%] | 1.5% [1.2%-1.8%] |  |
| 1991 | 8.4% [2.3%-17.2%] | 9.1% [2.5%-17.6%] | 8.7% [2.4%-17.5%] | 1.3% [0.6%-1.9%] | 0.8% [0.4%-1.1%] | 1.1% [0.5%-1.5%] | 2.6% [-0.6%-5.1%] | 1.7% [-0.3%-3.5%] | 2.3% [-0.5%-4.5%] | 1.3% [0.5%-1.9%] | 1.6% [0.6%-2.3%] | 1.4% [0.6%-2.1%] | 0.8% [0.5%-1.1%] | 2.0% [0.6%-3.2%] | 1.4% [0.5%-2.0%] | 1.0% [-0.0%-2.0%] | 2.5% [-0.0%-5.2%] | 1.6% [-0.0%-3.3%] | 0.1% [-0.0%-0.2%] | 0.2% [-0.0%-0.3%] | 0.1% [-0.0%-0.3%] | 0.2% [0.1%-0.3%] | 0.2% [0.1%-0.4%] | 0.2% [0.1%-0.3%] | 1.8% [-0.0%-9.3%] | 1.4% [-0.0%-7.0%] | 1.7% [-0.0%-8.4%] | 1.2% [1.0%-1.4%] | 1.9% [1.4%-2.3%] | 1.5% [1.2%-1.8%] |  |
| 1992 | 8.3% [2.3%-16.7%] | 9.0% [2.5%-17.4%] | 8.7% [2.4%-17.2%] | 1.3% [0.6%-1.8%] | 0.7% [0.4%-1.1%] | 1.0% [0.5%-1.5%] | 2.5% [-0.6%-5.0%] | 1.7% [-0.3%-3.4%] | 2.2% [-0.5%-4.3%] | 1.3% [0.6%-1.9%] | 1.6% [0.6%-2.3%] | 1.4% [0.6%-2.1%] | 0.8% [0.5%-1.1%] | 2.0% [0.6%-3.2%] | 1.4% [0.5%-2.0%] | 1.0% [-0.0%-2.0%] | 2.5% [-0.0%-5.2%] | 1.6% [-0.0%-3.4%] | 0.1% [-0.0%-0.2%] | 0.2% [-0.0%-0.3%] | 0.1% [-0.0%-0.3%] | 0.2% [0.1%-0.3%] | 0.2% [0.1%-0.3%] | 0.2% [0.1%-0.3%] | 1.8% [-0.0%-9.2%] | 1.4% [-0.0%-6.8%] | 1.6% [-0.0%-8.2%] | 1.2% [1.0%-1.4%] | 1.9% [1.4%-2.3%] | 1.5% [1.2%-1.7%] |  |
| 1993 | 8.2% [2.3%-16.7%] | 9.0% [2.5%-17.2%] | 8.6% [2.4%-17.1%] | 1.2% [0.6%-1.8%] | 0.7% [0.4%-1.1%] | 1.0% [0.5%-1.5%] | 2.5% [-0.6%-5.0%] | 1.6% [-0.3%-3.3%] | 2.2% [-0.5%-4.3%] | 1.3% [0.6%-2.0%] | 1.6% [0.6%-2.3%] | 1.4% [0.6%-2.1%] | 0.8% [0.5%-1.1%] | 2.0% [0.6%-3.2%] | 1.4% [0.5%-2.0%] | 1.0% [-0.0%-2.1%] | 2.5% [-0.0%-5.3%] | 1.7% [-0.0%-3.4%] | 0.1% [-0.0%-0.2%] | 0.2% [-0.0%-0.3%] | 0.1% [-0.0%-0.3%] | 0.2% [0.1%-0.3%] | 0.2% [0.1%-0.3%] | 0.2% [0.1%-0.3%] | 1.8% [-0.0%-9.0%] | 1.4% [-0.0%-6.7%] | 1.6% [-0.0%-8.0%] | 1.2% [1.0%-1.3%] | 1.9% [1.4%-2.3%] | 1.5% [1.2%-1.7%] |  |
| 1994 | 8.1% [2.3%-16.5%] | 8.9% [2.5%-17.2%] | 8.5% [2.4%-16.8%] | 1.2% [0.6%-1.8%] | 0.7% [0.4%-1.1%] | 1.0% [0.5%-1.5%] | 2.4% [-0.6%-4.9%] | 1.6% [-0.3%-3.2%] | 2.1% [-0.5%-4.2%] | 1.3% [0.6%-1.9%] | 1.6% [0.6%-2.3%] | 1.4% [0.6%-2.1%] | 0.8% [0.5%-1.1%] | 2.0% [0.6%-3.2%] | 1.4% [0.5%-2.0%] | 1.0% [-0.0%-2.1%] | 2.5% [-0.0%-5.3%] | 1.7% [-0.0%-3.4%] | 0.1% [-0.0%-0.2%] | 0.2% [-0.0%-0.3%] | 0.1% [-0.0%-0.3%] | 0.2% [0.1%-0.2%] | 0.2% [0.1%-0.3%] | 0.2% [0.1%-0.3%] | 1.8% [-0.0%-8.9%] | 1.3% [-0.0%-6.6%] | 1.6% [-0.0%-7.9%] | 1.1% [0.9%-1.3%] | 1.8% [1.4%-2.2%] | 1.4% [1.2%-1.7%] |  |
| 1995 | 7.9% [2.3%-16.1%] | 8.8% [2.4%-17.0%] | 8.4% [2.4%-16.6%] | 1.2% [0.6%-1.8%] | 0.7% [0.4%-1.1%] | 1.0% [0.5%-1.5%] | 2.3% [-0.5%-4.8%] | 1.5% [-0.3%-3.1%] | 2.0% [-0.4%-4.1%] | 1.3% [0.6%-2.0%] | 1.6% [0.6%-2.3%] | 1.4% [0.6%-2.1%] | 0.8% [0.5%-1.1%] | 2.0% [0.6%-3.2%] | 1.4% [0.5%-2.0%] | 1.0% [-0.0%-2.1%] | 2.6% [-0.0%-5.3%] | 1.7% [-0.0%-3.4%] | 0.1% [-0.0%-0.2%] | 0.2% [-0.0%-0.3%] | 0.1% [-0.0%-0.3%] | 0.2% [0.1%-0.2%] | 0.2% [0.1%-0.3%] | 0.2% [0.1%-0.3%] | 1.7% [-0.0%-8.7%] | 1.3% [-0.0%-6.5%] | 1.6% [-0.0%-7.8%] | 1.1% [0.9%-1.3%] | 1.8% [1.4%-2.2%] | 1.4% [1.2%-1.7%] |  |
| 1996 | 7.8% [2.4%-15.8%] | 8.7% [2.4%-16.7%] | 8.2% [2.4%-16.3%] | 1.2% [0.6%-1.7%] | 0.7% [0.4%-1.1%] | 1.0% [0.5%-1.4%] | 2.2% [-0.5%-4.5%] | 1.5% [-0.3%-2.9%] | 1.9% [-0.4%-3.9%] | 1.3% [0.6%-2.0%] | 1.6% [0.6%-2.3%] | 1.4% [0.6%-2.1%] | 0.8% [0.5%-1.1%] | 2.0% [0.6%-3.2%] | 1.4% [0.5%-2.0%] | 1.0% [-0.0%-2.1%] | 2.6% [-0.0%-5.4%] | 1.7% [-0.0%-3.5%] | 0.1% [-0.0%-0.3%] | 0.2% [-0.0%-0.3%] | 0.1% [-0.0%-0.3%] | 0.2% [0.1%-0.2%] | 0.2% [0.1%-0.3%] | 0.2% [0.1%-0.3%] | 1.7% [-0.0%-8.5%] | 1.3% [-0.0%-6.4%] | 1.5% [-0.0%-7.6%] | 1.1% [0.9%-1.3%] | 1.8% [1.4%-2.2%] | 1.4% [1.1%-1.6%] |  |
| 1997 | 7.6% [2.4%-15.4%] | 8.6% [2.4%-16.5%] | 8.1% [2.4%-15.9%] | 1.2% [0.6%-1.7%] | 0.7% [0.4%-1.1%] | 1.0% [0.5%-1.4%] | 2.1% [-0.5%-4.4%] | 1.4% [-0.3%-2.8%] | 1.8% [-0.4%-3.8%] | 1.3% [0.6%-2.0%] | 1.6% [0.6%-2.3%] | 1.4% [0.6%-2.1%] | 0.8% [0.5%-1.1%] | 2.0% [0.6%-3.2%] | 1.4% [0.5%-2.0%] | 1.1% [-0.0%-2.1%] | 2.6% [-0.0%-5.4%] | 1.7% [-0.0%-3.5%] | 0.1% [-0.0%-0.3%] | 0.2% [-0.0%-0.3%] | 0.1% [-0.0%-0.3%] | 0.2% [0.1%-0.2%] | 0.2% [0.1%-0.3%] | 0.2% [0.1%-0.3%] | 1.7% [-0.0%-8.4%] | 1.3% [0.0%-6.2%] | 1.5% [-0.0%-7.5%] | 1.1% [0.9%-1.2%] | 1.8% [1.3%-2.1%] | 1.4% [1.1%-1.6%] |  |
| 1998 | 7.5% [2.4%-15.2%] | 8.5% [2.5%-16.4%] | 8.0% [2.4%-15.8%] | 1.2% [0.6%-1.7%] | 0.7% [0.4%-1.0%] | 1.0% [0.5%-1.4%] | 2.0% [-0.5%-4.2%] | 1.3% [-0.2%-2.7%] | 1.7% [-0.4%-3.6%] | 1.3% [0.6%-2.0%] | 1.6% [0.6%-2.3%] | 1.4% [0.6%-2.1%] | 0.8% [0.5%-1.1%] | 2.0% [0.6%-3.2%] | 1.4% [0.5%-2.0%] | 1.1% [-0.0%-2.1%] | 2.6% [-0.0%-5.5%] | 1.7% [-0.0%-3.5%] | 0.1% [-0.0%-0.3%] | 0.2% [-0.0%-0.3%] | 0.1% [-0.0%-0.3%] | 0.2% [0.1%-0.2%] | 0.2% [0.1%-0.3%] | 0.2% [0.1%-0.3%] | 1.7% [-0.0%-8.3%] | 1.2% [0.0%-6.0%] | 1.5% [-0.0%-7.4%] | 1.1% [0.9%-1.2%] | 1.7% [1.3%-2.1%] | 1.4% [1.1%-1.6%] |  |
| 1999 | 7.4% [2.4%-14.9%] | 8.4% [2.5%-16.2%] | 7.9% [2.4%-15.5%] | 1.1% [0.6%-1.6%] | 0.7% [0.4%-1.0%] | 1.0% [0.5%-1.4%] | 1.9% [-0.4%-4.0%] | 1.2% [-0.2%-2.5%] | 1.6% [-0.3%-3.4%] | 1.3% [0.6%-2.0%] | 1.6% [0.6%-2.3%] | 1.4% [0.6%-2.1%] | 0.8% [0.5%-1.1%] | 2.0% [0.6%-3.2%] | 1.4% [0.5%-2.0%] | 1.1% [-0.0%-2.2%] | 2.6% [-0.0%-5.5%] | 1.7% [-0.0%-3.5%] | 0.1% [-0.0%-0.3%] | 0.2% [-0.0%-0.3%] | 0.1% [-0.0%-0.3%] | 0.2% [0.1%-0.2%] | 0.2% [0.1%-0.3%] | 0.2% [0.1%-0.3%] | 1.6% [-0.0%-8.3%] | 1.2% [0.0%-6.1%] | 1.5% [-0.0%-7.4%] | 1.0% [0.9%-1.2%] | 1.7% [1.3%-2.1%] | 1.3% [1.1%-1.6%] |  |
| 2000 | 7.3% [2.3%-14.7%] | 8.3% [2.5%-15.8%] | 7.7% [2.3%-15.2%] | 1.1% [0.6%-1.6%] | 0.7% [0.4%-1.0%] | 0.9% [0.5%-1.4%] | 1.8% [-0.4%-3.8%] | 1.1% [-0.2%-2.4%] | 1.6% [-0.3%-3.3%] | 1.4% [0.6%-2.0%] | 1.6% [0.6%-2.3%] | 1.4% [0.6%-2.2%] | 0.8% [0.5%-1.1%] | 2.0% [0.6%-3.2%] | 1.4% [0.5%-2.0%] | 1.1% [-0.0%-2.2%] | 2.6% [-0.0%-5.5%] | 1.7% [-0.0%-3.5%] | 0.1% [-0.0%-0.3%] | 0.2% [-0.0%-0.3%] | 0.1% [-0.0%-0.3%] | 0.2% [0.1%-0.2%] | 0.2% [0.1%-0.3%] | 0.2% [0.1%-0.3%] | 1.6% [-0.0%-8.2%] | 1.2% [0.0%-6.0%] | 1.5% [-0.0%-7.3%] | 1.0% [0.9%-1.2%] | 1.7% [1.3%-2.1%] | 1.3% [1.1%-1.6%] |  |
| 2001 | 7.2% [2.4%-14.5%] | 8.2% [2.5%-15.7%] | 7.7% [2.4%-15.1%] | 1.1% [0.6%-1.6%] | 0.7% [0.4%-1.0%] | 0.9% [0.5%-1.3%] | 1.7% [-0.4%-3.7%] | 1.1% [-0.2%-2.3%] | 1.5% [-0.3%-3.2%] | 1.4% [0.6%-2.0%] | 1.6% [0.6%-2.3%] | 1.5% [0.6%-2.2%] | 0.9% [0.5%-1.2%] | 2.0% [0.6%-3.2%] | 1.4% [0.5%-2.0%] | 1.1% [-0.0%-2.2%] | 2.7% [-0.0%-5.5%] | 1.7% [-0.0%-3.6%] | 0.1% [-0.0%-0.3%] | 0.2% [-0.0%-0.4%] | 0.1% [-0.0%-0.3%] | 0.2% [0.1%-0.2%] | 0.2% [0.1%-0.3%] | 0.2% [0.1%-0.3%] | 1.6% [-0.0%-8.2%] | 1.2% [0.0%-5.9%] | 1.5% [-0.0%-7.3%] | 1.0% [0.9%-1.2%] | 1.7% [1.3%-2.1%] | 1.3% [1.1%-1.6%] |  |
| 2002 | 7.0% [2.4%-14.2%] | 8.2% [2.5%-15.5%] | 7.6% [2.3%-14.9%] | 1.1% [0.6%-1.6%] | 0.6% [0.3%-1.0%] | 0.9% [0.5%-1.3%] | 1.6% [-0.3%-3.5%] | 1.0% [-0.2%-2.2%] | 1.4% [-0.3%-3.0%] | 1.4% [0.6%-2.0%] | 1.6% [0.6%-2.3%] | 1.5% [0.6%-2.2%] | 0.9% [0.5%-1.2%] | 2.0% [0.6%-3.2%] | 1.4% [0.5%-2.0%] | 1.1% [-0.0%-2.2%] | 2.7% [-0.0%-5.5%] | 1.7% [-0.0%-3.6%] | 0.1% [-0.0%-0.3%] | 0.2% [-0.0%-0.4%] | 0.1% [-0.0%-0.3%] | 0.2% [0.1%-0.2%] | 0.2% [0.1%-0.3%] | 0.2% [0.1%-0.3%] | 1.6% [-0.0%-8.1%] | 1.2% [0.0%-5.9%] | 1.5% [-0.0%-7.2%] | 1.0% [0.9%-1.2%] | 1.7% [1.3%-2.1%] | 1.3% [1.1%-1.5%] |  |
| 2003 | 7.0% [2.4%-14.2%] | 8.1% [2.5%-15.5%] | 7.5% [2.3%-14.8%] | 1.1% [0.6%-1.5%] | 0.6% [0.3%-0.9%] | 0.9% [0.5%-1.3%] | 1.6% [-0.3%-3.4%] | 1.0% [-0.2%-2.1%] | 1.3% [-0.3%-2.9%] | 1.4% [0.6%-2.1%] | 1.6% [0.7%-2.3%] | 1.5% [0.6%-2.2%] | 0.9% [0.5%-1.2%] | 2.0% [0.6%-3.2%] | 1.4% [0.5%-2.0%] | 1.1% [-0.0%-2.2%] | 2.7% [-0.0%-5.6%] | 1.7% [-0.0%-3.6%] | 0.1% [-0.0%-0.3%] | 0.2% [-0.0%-0.4%] | 0.1% [-0.0%-0.3%] | 0.1% [0.1%-0.2%] | 0.2% [0.1%-0.3%] | 0.2% [0.1%-0.3%] | 1.6% [-0.0%-8.1%] | 1.2% [0.0%-5.9%] | 1.5% [-0.0%-7.2%] | 1.0% [0.9%-1.2%] | 1.7% [1.3%-2.1%] | 1.3% [1.1%-1.5%] |  |
| 2004 | 6.9% [2.4%-14.0%] | 8.0% [2.5%-15.3%] | 7.4% [2.4%-14.6%] | 1.0% [0.6%-1.5%] | 0.6% [0.3%-0.9%] | 0.9% [0.5%-1.3%] | 1.5% [-0.3%-3.2%] | 0.9% [-0.2%-1.9%] | 1.3% [-0.3%-2.7%] | 1.4% [0.6%-2.1%] | 1.6% [0.7%-2.3%] | 1.5% [0.6%-2.2%] | 0.9% [0.5%-1.2%] | 2.1% [0.6%-3.3%] | 1.4% [0.5%-2.0%] | 1.1% [-0.0%-2.3%] | 2.7% [-0.0%-5.6%] | 1.8% [-0.0%-3.6%] | 0.1% [-0.0%-0.3%] | 0.2% [-0.0%-0.4%] | 0.2% [-0.0%-0.3%] | 0.1% [0.1%-0.2%] | 0.2% [0.1%-0.3%] | 0.2% [0.1%-0.3%] | 1.6% [-0.0%-8.1%] | 1.2% [0.0%-5.9%] | 1.5% [0.0%-7.2%] | 1.0% [0.9%-1.2%] | 1.7% [1.3%-2.1%] | 1.3% [1.1%-1.5%] |  |
| 2005 | 6.8% [2.4%-13.8%] | 8.0% [2.5%-15.1%] | 7.3% [2.4%-14.3%] | 1.0% [0.5%-1.5%] | 0.6% [0.3%-0.9%] | 0.9% [0.5%-1.2%] | 1.4% [-0.3%-3.0%] | 0.8% [-0.1%-1.8%] | 1.2% [-0.2%-2.5%] | 1.4% [0.6%-2.1%] | 1.6% [0.7%-2.4%] | 1.5% [0.6%-2.2%] | 0.9% [0.5%-1.2%] | 2.1% [0.6%-3.3%] | 1.4% [0.6%-2.0%] | 1.1% [-0.0%-2.3%] | 2.7% [-0.0%-5.7%] | 1.8% [-0.0%-3.7%] | 0.1% [-0.0%-0.3%] | 0.2% [-0.0%-0.4%] | 0.2% [-0.0%-0.3%] | 0.1% [0.1%-0.2%] | 0.2% [0.1%-0.3%] | 0.2% [0.1%-0.2%] | 1.6% [-0.0%-7.9%] | 1.1% [0.0%-5.7%] | 1.4% [0.0%-7.1%] | 1.0% [0.9%-1.2%] | 1.7% [1.3%-2.0%] | 1.3% [1.1%-1.5%] |  |
| 2006 | 6.6% [2.4%-13.4%] | 7.9% [2.5%-14.8%] | 7.2% [2.4%-13.9%] | 1.0% [0.5%-1.4%] | 0.6% [0.3%-0.9%] | 0.8% [0.4%-1.2%] | 1.3% [-0.3%-2.8%] | 0.8% [-0.1%-1.6%] | 1.1% [-0.2%-2.3%] | 1.4% [0.6%-2.1%] | 1.6% [0.7%-2.4%] | 1.5% [0.6%-2.2%] | 0.9% [0.6%-1.2%] | 2.1% [0.6%-3.3%] | 1.4% [0.6%-2.1%] | 1.2% [-0.0%-2.3%] | 2.8% [-0.0%-5.7%] | 1.8% [-0.0%-3.7%] | 0.1% [-0.0%-0.3%] | 0.2% [-0.0%-0.4%] | 0.2% [-0.0%-0.3%] | 0.1% [0.1%-0.2%] | 0.2% [0.1%-0.3%] | 0.2% [0.1%-0.3%] | 1.5% [-0.0%-7.6%] | 1.1% [0.0%-5.5%] | 1.4% [0.0%-6.8%] | 1.0% [0.9%-1.2%] | 1.7% [1.3%-2.0%] | 1.3% [1.1%-1.5%] |  |
| 2007 | 6.5% [2.4%-13.1%] | 7.8% [2.5%-14.6%] | 7.1% [2.4%-13.7%] | 1.0% [0.5%-1.4%] | 0.6% [0.3%-0.9%] | 0.8% [0.4%-1.2%] | 1.2% [-0.3%-2.6%] | 0.7% [-0.1%-1.5%] | 1.0% [-0.2%-2.2%] | 1.5% [0.6%-2.1%] | 1.6% [0.7%-2.4%] | 1.5% [0.6%-2.3%] | 0.9% [0.6%-1.3%] | 2.1% [0.6%-3.3%] | 1.4% [0.6%-2.1%] | 1.2% [-0.0%-2.4%] | 2.8% [-0.0%-5.8%] | 1.8% [-0.0%-3.7%] | 0.1% [-0.0%-0.3%] | 0.2% [-0.0%-0.4%] | 0.2% [-0.0%-0.4%] | 0.1% [0.1%-0.2%] | 0.2% [0.1%-0.3%] | 0.2% [0.1%-0.2%] | 1.5% [-0.0%-7.5%] | 1.0% [0.0%-5.2%] | 1.3% [0.0%-6.6%] | 1.0% [0.8%-1.2%] | 1.7% [1.2%-2.0%] | 1.3% [1.1%-1.5%] |  |
| 2008 | 6.4% [2.4%-13.0%] | 7.7% [2.5%-14.5%] | 7.0% [2.4%-13.5%] | 1.0% [0.5%-1.4%] | 0.6% [0.3%-0.8%] | 0.8% [0.4%-1.2%] | 1.1% [-0.2%-2.4%] | 0.7% [-0.1%-1.4%] | 0.9% [-0.2%-2.0%] | 1.5% [0.6%-2.2%] | 1.6% [0.7%-2.4%] | 1.5% [0.6%-2.3%] | 0.9% [0.6%-1.3%] | 2.1% [0.6%-3.3%] | 1.4% [0.6%-2.1%] | 1.2% [-0.0%-2.4%] | 2.8% [-0.0%-5.9%] | 1.9% [-0.0%-3.8%] | 0.2% [-0.0%-0.3%] | 0.2% [-0.0%-0.4%] | 0.2% [-0.0%-0.4%] | 0.1% [0.1%-0.2%] | 0.2% [0.1%-0.3%] | 0.2% [0.1%-0.2%] | 1.5% [-0.0%-7.4%] | 1.0% [0.0%-5.1%] | 1.3% [0.0%-6.5%] | 1.0% [0.8%-1.2%] | 1.6% [1.2%-2.0%] | 1.3% [1.0%-1.5%] |  |
| 2009 | 6.3% [2.4%-12.8%] | 7.7% [2.5%-14.4%] | 6.9% [2.4%-13.4%] | 0.9% [0.5%-1.4%] | 0.6% [0.3%-0.8%] | 0.8% [0.4%-1.1%] | 1.1% [-0.2%-2.3%] | 0.6% [-0.1%-1.4%] | 0.9% [-0.2%-1.9%] | 1.5% [0.6%-2.2%] | 1.6% [0.7%-2.4%] | 1.5% [0.7%-2.3%] | 1.0% [0.6%-1.3%] | 2.1% [0.6%-3.3%] | 1.4% [0.6%-2.1%] | 1.2% [-0.0%-2.5%] | 2.9% [-0.0%-5.9%] | 1.9% [-0.0%-3.8%] | 0.2% [-0.0%-0.3%] | 0.2% [-0.0%-0.4%] | 0.2% [-0.0%-0.4%] | 0.1% [0.1%-0.2%] | 0.2% [0.1%-0.3%] | 0.2% [0.1%-0.2%] | 1.4% [-0.0%-7.3%] | 1.0% [-0.0%-5.0%] | 1.3% [0.0%-6.4%] | 1.0% [0.8%-1.1%] | 1.6% [1.2%-2.0%] | 1.2% [1.0%-1.5%] |  |
| 2010 | 6.3% [2.4%-12.7%] | 7.6% [2.4%-14.3%] | 6.9% [2.4%-13.2%] | 0.9% [0.5%-1.4%] | 0.6% [0.3%-0.8%] | 0.8% [0.4%-1.1%] | 1.0% [-0.2%-2.2%] | 0.6% [-0.1%-1.3%] | 0.8% [-0.2%-1.8%] | 1.5% [0.6%-2.3%] | 1.6% [0.7%-2.4%] | 1.6% [0.7%-2.3%] | 1.0% [0.6%-1.4%] | 2.1% [0.6%-3.3%] | 1.4% [0.6%-2.2%] | 1.2% [-0.0%-2.5%] | 2.9% [-0.0%-5.9%] | 1.9% [-0.0%-3.9%] | 0.2% [-0.0%-0.4%] | 0.2% [-0.0%-0.4%] | 0.2% [-0.0%-0.4%] | 0.1% [0.1%-0.2%] | 0.2% [0.1%-0.3%] | 0.2% [0.1%-0.2%] | 1.4% [-0.0%-7.0%] | 1.0% [-0.0%-4.9%] | 1.2% [-0.0%-6.2%] | 1.0% [0.8%-1.1%] | 1.6% [1.2%-1.9%] | 1.2% [1.0%-1.4%] | |
| 2011 | 6.2% [2.4%-12.4%] | 7.6% [2.4%-14.1%] | 6.8% [2.4%-13.0%] | 0.9% [0.5%-1.3%] | 0.6% [0.3%-0.8%] | 0.8% [0.4%-1.1%] | 0.9% [-0.2%-2.1%] | 0.5% [-0.1%-1.2%] | 0.8% [-0.2%-1.7%] | 1.5% [0.6%-2.3%] | 1.6% [0.7%-2.4%] | 1.6% [0.7%-2.3%] | 1.0% [0.6%-1.4%] | 2.1% [0.6%-3.3%] | 1.5% [0.6%-2.2%] | 1.3% [-0.0%-2.6%] | 2.9% [-0.0%-6.0%] | 1.9% [-0.0%-3.9%] | 0.2% [-0.0%-0.4%] | 0.2% [-0.0%-0.5%] | 0.2% [-0.0%-0.4%] | 0.1% [0.1%-0.2%] | 0.2% [0.1%-0.3%] | 0.2% [0.1%-0.2%] | 1.4% [-0.0%-6.8%] | 0.9% [0.0%-4.7%] | 1.2% [-0.0%-6.0%] | 0.9% [0.8%-1.1%] | 1.6% [1.2%-1.9%] | 1.2% [1.0%-1.4%] | |
| 2012 | 6.1% [2.3%-12.3%] | 7.5% [2.4%-14.0%] | 6.7% [2.4%-12.8%] | 0.9% [0.5%-1.3%] | 0.5% [0.3%-0.8%] | 0.8% [0.4%-1.1%] | 0.9% [-0.2%-2.0%] | 0.5% [-0.1%-1.1%] | 0.7% [-0.1%-1.6%] | 1.6% [0.6%-2.3%] | 1.6% [0.7%-2.4%] | 1.6% [0.7%-2.3%] | 1.0% [0.6%-1.4%] | 2.1% [0.6%-3.4%] | 1.5% [0.6%-2.1%] | 1.3% [-0.0%-2.6%] | 2.9% [-0.0%-6.0%] | 1.9% [-0.0%-4.0%] | 0.2% [-0.0%-0.4%] | 0.2% [-0.0%-0.5%] | 0.2% [-0.0%-0.4%] | 0.1% [0.1%-0.2%] | 0.2% [0.1%-0.3%] | 0.1% [0.1%-0.2%] | 1.3% [-0.0%-6.7%] | 0.9% [0.0%-4.7%] | 1.2% [-0.0%-5.9%] | 0.9% [0.8%-1.1%] | 1.6% [1.2%-1.9%] | 1.2% [1.0%-1.4%] | |
| 2013 | 6.1% [2.4%-12.1%] | 7.5% [2.4%-13.9%] | 6.7% [2.4%-12.6%] | 0.9% [0.5%-1.3%] | 0.5% [0.3%-0.8%] | 0.8% [0.4%-1.1%] | 0.8% [-0.2%-1.9%] | 0.5% [-0.1%-1.1%] | 0.7% [-0.1%-1.6%] | 1.6% [0.7%-2.3%] | 1.6% [0.7%-2.4%] | 1.6% [0.7%-2.4%] | 1.0% [0.6%-1.4%] | 2.1% [0.6%-3.4%] | 1.5% [0.6%-2.2%] | 1.3% [-0.0%-2.6%] | 3.0% [-0.0%-6.1%] | 2.0% [-0.0%-4.0%] | 0.2% [-0.0%-0.4%] | 0.2% [-0.1%-0.5%] | 0.2% [-0.0%-0.4%] | 0.1% [0.1%-0.2%] | 0.2% [0.1%-0.3%] | 0.1% [0.1%-0.2%] | 1.3% [-0.0%-6.6%] | 0.9% [0.0%-4.5%] | 1.2% [-0.0%-5.8%] | 0.9% [0.7%-1.1%] | 1.5% [1.1%-1.9%] | 1.2% [1.0%-1.4%] | |
| 2014 | 6.0% [2.3%-12.0%] | 7.4% [2.4%-13.8%] | 6.6% [2.4%-12.5%] | 0.9% [0.5%-1.3%] | 0.5% [0.3%-0.8%] | 0.7% [0.4%-1.1%] | 0.8% [-0.2%-1.8%] | 0.5% [-0.1%-1.0%] | 0.7% [-0.1%-1.5%] | 1.6% [0.7%-2.4%] | 1.6% [0.7%-2.4%] | 1.6% [0.7%-2.4%] | 1.0% [0.6%-1.5%] | 2.1% [0.6%-3.4%] | 1.5% [0.6%-2.2%] | 1.3% [-0.0%-2.6%] | 3.0% [-0.0%-6.1%] | 2.0% [-0.0%-4.0%] | 0.2% [-0.0%-0.4%] | 0.2% [-0.1%-0.5%] | 0.2% [-0.0%-0.4%] | 0.1% [0.1%-0.2%] | 0.2% [0.1%-0.2%] | 0.1% [0.1%-0.2%] | 1.3% [-0.0%-6.5%] | 0.9% [0.0%-4.4%] | 1.1% [-0.0%-5.6%] | 0.9% [0.7%-1.1%] | 1.5% [1.1%-1.9%] | 1.2% [0.9%-1.4%] | |
| 2015 | 6.0% [2.4%-11.8%] | 7.4% [2.4%-13.7%] | 6.6% [2.4%-12.4%] | 0.9% [0.5%-1.3%] | 0.5% [0.3%-0.8%] | 0.7% [0.4%-1.1%] | 0.8% [-0.2%-1.7%] | 0.5% [-0.1%-1.0%] | 0.7% [-0.1%-1.4%] | 1.6% [0.7%-2.4%] | 1.6% [0.7%-2.4%] | 1.6% [0.7%-2.4%] | 1.0% [0.6%-1.5%] | 2.1% [0.6%-3.4%] | 1.5% [0.6%-2.2%] | 1.3% [-0.0%-2.7%] | 3.0% [-0.0%-6.2%] | 2.0% [-0.0%-4.0%] | 0.2% [-0.0%-0.4%] | 0.2% [-0.1%-0.5%] | 0.2% [-0.0%-0.4%] | 0.1% [0.1%-0.2%] | 0.2% [0.1%-0.2%] | 0.1% [0.1%-0.2%] | 1.3% [-0.0%-6.3%] | 0.9% [0.0%-4.3%] | 1.1% [-0.0%-5.4%] | 0.9% [0.7%-1.1%] | 1.5% [1.1%-1.9%] | 1.1% [0.9%-1.4%] | |
| 2016 | 6.0% [2.4%-11.6%] | 7.4% [2.4%-13.6%] | 6.6% [2.4%-12.3%] | 0.9% [0.5%-1.3%] | 0.5% [0.3%-0.8%] | 0.7% [0.4%-1.1%] | 0.8% [-0.2%-1.7%] | 0.4% [-0.1%-1.0%] | 0.6% [-0.1%-1.4%] | 1.6% [0.7%-2.4%] | 1.6% [0.7%-2.4%] | 1.6% [0.7%-2.4%] | 1.1% [0.6%-1.5%] | 2.1% [0.6%-3.4%] | 1.5% [0.6%-2.2%] | 1.4% [-0.0%-2.7%] | 3.0% [-0.0%-6.2%] | 2.0% [-0.0%-4.1%] | 0.2% [-0.0%-0.4%] | 0.2% [-0.1%-0.5%] | 0.2% [-0.0%-0.4%] | 0.1% [0.1%-0.2%] | 0.2% [0.1%-0.2%] | 0.1% [0.1%-0.2%] | 1.2% [-0.0%-6.2%] | 0.8% [0.0%-4.2%] | 1.1% [-0.0%-5.4%] | 0.9% [0.7%-1.0%] | 1.5% [1.1%-1.8%] | 1.1% [0.9%-1.3%] | |
| 2017 | 6.0% [2.3%-11.6%] | 7.4% [2.3%-13.6%] | 6.6% [2.4%-12.3%] | 0.9% [0.5%-1.3%] | 0.5% [0.3%-0.8%] | 0.7% [0.4%-1.1%] | 0.7% [-0.2%-1.6%] | 0.4% [-0.1%-0.9%] | 0.6% [-0.1%-1.3%] | 1.6% [0.7%-2.4%] | 1.6% [0.7%-2.5%] | 1.6% [0.7%-2.4%] | 1.1% [0.6%-1.5%] | 2.1% [0.6%-3.4%] | 1.5% [0.6%-2.2%] | 1.4% [-0.0%-2.7%] | 3.0% [-0.0%-6.2%] | 2.0% [-0.0%-4.1%] | 0.2% [-0.0%-0.4%] | 0.2% [-0.1%-0.5%] | 0.2% [-0.0%-0.4%] | 0.1% [0.1%-0.2%] | 0.2% [0.1%-0.2%] | 0.1% [0.1%-0.2%] | 1.2% [-0.0%-6.0%] | 0.8% [0.0%-4.1%] | 1.1% [-0.0%-5.2%] | 0.9% [0.7%-1.0%] | 1.5% [1.1%-1.8%] | 1.1% [0.9%-1.3%] | |
| 2018 | 6.0% [2.4%-11.6%] | 7.4% [2.3%-13.6%] | 6.6% [2.4%-12.3%] | 0.9% [0.5%-1.2%] | 0.5% [0.3%-0.8%] | 0.7% [0.4%-1.0%] | 0.7% [-0.2%-1.6%] | 0.4% [-0.1%-0.9%] | 0.6% [-0.1%-1.3%] | 1.7% [0.7%-2.5%] | 1.6% [0.7%-2.5%] | 1.7% [0.7%-2.5%] | 1.1% [0.6%-1.6%] | 2.1% [0.6%-3.4%] | 1.5% [0.6%-2.3%] | 1.4% [-0.0%-2.8%] | 3.0% [-0.0%-6.2%] | 2.1% [-0.0%-4.2%] | 0.2% [-0.0%-0.4%] | 0.2% [-0.1%-0.5%] | 0.2% [-0.0%-0.5%] | 0.1% [0.1%-0.2%] | 0.1% [0.1%-0.2%] | 0.1% [0.1%-0.2%] | 1.2% [-0.0%-5.9%] | 0.8% [0.0%-4.1%] | 1.0% [-0.0%-5.2%] | 0.9% [0.7%-1.0%] | 1.5% [1.1%-1.8%] | 1.1% [0.9%-1.3%] | |
| 2019 | 6.0% [2.4%-11.6%] | 7.4% [2.3%-13.5%] | 6.6% [2.4%-12.2%] | 0.8% [0.5%-1.2%] | 0.5% [0.3%-0.8%] | 0.7% [0.4%-1.1%] | 0.7% [-0.2%-1.6%] | 0.4% [-0.1%-0.9%] | 0.6% [-0.1%-1.3%] | 1.7% [0.7%-2.5%] | 1.6% [0.7%-2.5%] | 1.7% [0.7%-2.5%] | 1.1% [0.6%-1.6%] | 2.1% [0.6%-3.5%] | 1.6% [0.6%-2.3%] | 1.4% [-0.0%-2.8%] | 3.1% [-0.0%-6.3%] | 2.1% [-0.0%-4.2%] | 0.2% [-0.0%-0.4%] | 0.2% [-0.1%-0.5%] | 0.2% [-0.0%-0.5%] | 0.1% [0.1%-0.2%] | 0.1% [0.1%-0.2%] | 0.1% [0.1%-0.2%] | 1.2% [-0.0%-5.9%] | 0.8% [0.0%-4.1%] | 1.0% [-0.0%-5.2%] | 0.9% [0.7%-1.0%] | 1.5% [1.1%-1.8%] | 1.1% [0.9%-1.3%] | |
| 2020 | 6.0% [2.4%-11.6%] | 7.4% [2.3%-13.5%] | 6.6% [2.4%-12.2%] | 0.8% [0.5%-1.2%] | 0.5% [0.3%-0.8%] | 0.7% [0.4%-1.0%] | 0.7% [-0.2%-1.6%] | 0.4% [-0.1%-0.9%] | 0.6% [-0.1%-1.3%] | 1.7% [0.7%-2.5%] | 1.6% [0.7%-2.5%] | 1.7% [0.7%-2.5%] | 1.1% [0.6%-1.6%] | 2.1% [0.6%-3.5%] | 1.6% [0.6%-2.4%] | 1.4% [-0.0%-2.8%] | 3.1% [-0.0%-6.3%] | 2.1% [-0.0%-4.4%] | 0.2% [-0.0%-0.4%] | 0.2% [-0.1%-0.5%] | 0.2% [-0.0%-0.5%] | 0.1% [0.1%-0.2%] | 0.1% [0.1%-0.2%] | 0.1% [0.1%-0.2%] | 1.2% [-0.0%-5.8%] | 0.8% [0.0%-3.9%] | 1.0% [-0.0%-5.0%] | 0.9% [0.7%-1.0%] | 1.5% [1.1%-1.8%] | 1.1% [0.9%-1.3%] | |
| 2021 | 6.0% [2.4%-11.5%] | 7.4% [2.3%-13.4%] | 6.6% [2.4%-12.2%] | 0.8% [0.4%-1.2%] | 0.5% [0.3%-0.8%] | 0.7% [0.4%-1.0%] | 0.7% [-0.2%-1.6%] | 0.4% [-0.1%-0.9%] | 0.6% [-0.1%-1.3%] | 1.7% [0.7%-2.5%] | 1.6% [0.7%-2.5%] | 1.7% [0.7%-2.5%] | 1.1% [0.6%-1.7%] | 2.1% [0.6%-3.5%] | 1.6% [0.6%-2.3%] | 1.4% [-0.0%-2.9%] | 3.1% [-0.0%-6.3%] | 2.1% [-0.0%-4.3%] | 0.2% [-0.0%-0.4%] | 0.2% [-0.1%-0.5%] | 0.2% [-0.0%-0.5%] | 0.1% [0.1%-0.2%] | 0.1% [0.1%-0.2%] | 0.1% [0.1%-0.2%] | 1.2% [-0.0%-5.7%] | 0.8% [0.0%-4.0%] | 1.0% [-0.0%-5.0%] | 0.9% [0.7%-1.1%] | 1.5% [1.1%-1.8%] | 1.1% [0.9%-1.4%] | |
| **Note:** Data are presented as percentages (%) with 95% confidence intervals [lower-upper]. Values of "-0.0%" and "0.0%" result from rounding to one decimal place. "-0.0%" indicates that the original value was negative but rounded to zero (absolute value <0.05%), while "0.0%" indicates a positive value that rounded to zero (<0.05%). Negative values suggest an inverse relationship between the dietary factor and cancer mortality, while positive values indicate a positive association. All rates are age-standardized. | | | | | | | | | | | | | | | | | | | | | | | | | | | | | | | |

| Table S1e: Percentage of Total Cancer Mortality Attributable to Dietary Risk Factors - Low-middle SDI Regions (Age-standardized, 1990-2021). | | | | | | | | | | | | | | | | | | | | | | | | | | | | | | |
| --- | --- | --- | --- | --- | --- | --- | --- | --- | --- | --- | --- | --- | --- | --- | --- | --- | --- | --- | --- | --- | --- | --- | --- | --- | --- | --- | --- | --- | --- | --- |
|  | All dietary risks | | | Low in fruits | | | Low in vegetables | | | Low in whole grains | | | Low in milk | | | High in red meat | | | High in processed meat | | | Low in fiber | | | High in sodium | | | Low in calcium | | |
| Year | Male | Female | Both | Male | Female | Both | Male | Female | Both | Male | Female | Both | Male | Female | Both | Male | Female | Both | Male | Female | Both | Male | Female | Both | Male | Female | Both | Male | Female | Both |
| 1990 | 5.9% [2.8%-11.3%] | 7.0% [2.2%-12.6%] | 6.4% [2.5%-11.8%] | 1.6% [0.9%-2.4%] | 0.6% [0.3%-0.8%] | 1.1% [0.6%-1.7%] | 1.3% [-0.3%-2.5%] | 1.2% [-0.3%-2.3%] | 1.2% [-0.3%-2.4%] | 1.1% [0.5%-1.7%] | 1.3% [0.5%-2.0%] | 1.2% [0.5%-1.8%] | 0.4% [-0.1%-0.7%] | 1.7% [0.5%-2.8%] | 1.0% [0.6%-1.4%] | 0.6% [-0.0%-1.3%] | 1.9% [-0.0%-4.0%] | 1.2% [-0.0%-2.5%] | 0.1% [-0.0%-0.3%] | 0.2% [-0.0%-0.3%] | 0.2% [-0.0%-0.3%] | 0.2% [0.1%-0.3%] | 0.2% [0.1%-0.4%] | 0.2% [0.1%-0.3%] | 1.2% [0.0%-6.0%] | 0.8% [0.0%-4.0%] | 1.0% [0.0%-5.1%] | 1.0% [0.8%-1.3%] | 1.8% [1.3%-2.3%] | 1.4% [1.1%-1.7%] |
| 1991 | 5.9% [2.7%-11.2%] | 7.0% [2.1%-12.6%] | 6.4% [2.5%-11.8%] | 1.6% [0.9%-2.4%] | 0.6% [0.3%-0.8%] | 1.1% [0.6%-1.7%] | 1.3% [-0.3%-2.5%] | 1.1% [-0.3%-2.3%] | 1.2% [-0.3%-2.4%] | 1.1% [0.5%-1.7%] | 1.3% [0.5%-2.0%] | 1.2% [0.5%-1.8%] | 0.4% [-0.1%-0.7%] | 1.7% [0.5%-2.8%] | 1.0% [0.6%-1.4%] | 0.6% [-0.0%-1.3%] | 1.9% [-0.0%-4.1%] | 1.2% [-0.0%-2.6%] | 0.1% [-0.0%-0.3%] | 0.2% [-0.0%-0.3%] | 0.2% [-0.0%-0.3%] | 0.2% [0.1%-0.3%] | 0.2% [0.1%-0.4%] | 0.2% [0.1%-0.3%] | 1.2% [-0.0%-6.0%] | 0.8% [0.0%-4.0%] | 1.0% [0.0%-5.1%] | 1.0% [0.8%-1.3%] | 1.8% [1.3%-2.3%] | 1.4% [1.1%-1.6%] |
| 1992 | 5.8% [2.7%-11.1%] | 7.0% [2.1%-12.5%] | 6.4% [2.5%-11.7%] | 1.6% [0.8%-2.3%] | 0.6% [0.3%-0.8%] | 1.1% [0.6%-1.6%] | 1.3% [-0.3%-2.5%] | 1.1% [-0.3%-2.3%] | 1.2% [-0.3%-2.4%] | 1.1% [0.5%-1.7%] | 1.3% [0.5%-2.0%] | 1.2% [0.5%-1.8%] | 0.4% [-0.1%-0.7%] | 1.7% [0.5%-2.8%] | 1.0% [0.6%-1.4%] | 0.6% [-0.0%-1.3%] | 1.9% [-0.0%-4.2%] | 1.2% [-0.0%-2.6%] | 0.1% [-0.0%-0.3%] | 0.2% [-0.0%-0.3%] | 0.2% [-0.0%-0.3%] | 0.2% [0.1%-0.3%] | 0.2% [0.1%-0.4%] | 0.2% [0.1%-0.3%] | 1.1% [0.0%-5.9%] | 0.8% [0.0%-3.9%] | 1.0% [-0.0%-5.0%] | 1.0% [0.8%-1.3%] | 1.8% [1.3%-2.3%] | 1.4% [1.1%-1.6%] |
| 1993 | 5.8% [2.7%-11.0%] | 7.0% [2.1%-12.6%] | 6.4% [2.5%-11.7%] | 1.6% [0.8%-2.3%] | 0.6% [0.3%-0.8%] | 1.1% [0.6%-1.6%] | 1.3% [-0.3%-2.5%] | 1.1% [-0.3%-2.3%] | 1.2% [-0.3%-2.4%] | 1.1% [0.5%-1.7%] | 1.3% [0.5%-1.9%] | 1.2% [0.5%-1.8%] | 0.4% [-0.1%-0.7%] | 1.7% [0.5%-2.7%] | 1.0% [0.6%-1.4%] | 0.6% [-0.0%-1.3%] | 2.0% [-0.0%-4.2%] | 1.3% [-0.0%-2.6%] | 0.1% [-0.0%-0.3%] | 0.2% [-0.0%-0.3%] | 0.2% [-0.0%-0.3%] | 0.2% [0.1%-0.3%] | 0.2% [0.1%-0.4%] | 0.2% [0.1%-0.3%] | 1.1% [0.0%-5.8%] | 0.8% [0.0%-3.9%] | 1.0% [0.0%-5.0%] | 1.0% [0.8%-1.3%] | 1.8% [1.3%-2.3%] | 1.4% [1.1%-1.6%] |
| 1994 | 5.7% [2.7%-10.8%] | 7.0% [2.1%-12.5%] | 6.4% [2.5%-11.5%] | 1.6% [0.8%-2.3%] | 0.6% [0.3%-0.8%] | 1.1% [0.6%-1.6%] | 1.3% [-0.3%-2.5%] | 1.1% [-0.3%-2.3%] | 1.2% [-0.3%-2.4%] | 1.1% [0.5%-1.7%] | 1.3% [0.5%-1.9%] | 1.2% [0.5%-1.8%] | 0.4% [-0.1%-0.7%] | 1.7% [0.5%-2.7%] | 1.0% [0.6%-1.4%] | 0.6% [-0.0%-1.3%] | 2.0% [-0.0%-4.2%] | 1.3% [-0.0%-2.6%] | 0.1% [-0.0%-0.3%] | 0.2% [-0.0%-0.3%] | 0.2% [-0.0%-0.3%] | 0.2% [0.1%-0.3%] | 0.2% [0.1%-0.4%] | 0.2% [0.1%-0.3%] | 1.1% [0.0%-5.7%] | 0.7% [0.0%-3.8%] | 0.9% [0.0%-4.9%] | 1.0% [0.7%-1.2%] | 1.8% [1.3%-2.2%] | 1.4% [1.1%-1.6%] |
| 1995 | 5.7% [2.7%-10.7%] | 7.1% [2.1%-12.6%] | 6.4% [2.5%-11.5%] | 1.6% [0.8%-2.3%] | 0.6% [0.3%-0.8%] | 1.1% [0.6%-1.6%] | 1.3% [-0.3%-2.5%] | 1.1% [-0.3%-2.3%] | 1.2% [-0.3%-2.4%] | 1.1% [0.5%-1.7%] | 1.3% [0.5%-1.9%] | 1.2% [0.5%-1.8%] | 0.4% [-0.1%-0.7%] | 1.7% [0.5%-2.7%] | 1.0% [0.6%-1.4%] | 0.6% [-0.0%-1.3%] | 2.0% [-0.0%-4.3%] | 1.3% [-0.0%-2.7%] | 0.1% [-0.0%-0.3%] | 0.2% [-0.0%-0.3%] | 0.2% [-0.0%-0.3%] | 0.2% [0.1%-0.3%] | 0.2% [0.1%-0.4%] | 0.2% [0.1%-0.3%] | 1.1% [0.0%-5.5%] | 0.7% [0.0%-3.8%] | 0.9% [0.0%-4.7%] | 1.0% [0.8%-1.2%] | 1.8% [1.3%-2.2%] | 1.4% [1.1%-1.6%] |
| 1996 | 5.7% [2.7%-10.6%] | 7.1% [2.1%-12.5%] | 6.4% [2.5%-11.4%] | 1.5% [0.8%-2.2%] | 0.6% [0.3%-0.8%] | 1.1% [0.6%-1.6%] | 1.3% [-0.3%-2.5%] | 1.1% [-0.3%-2.3%] | 1.2% [-0.3%-2.4%] | 1.1% [0.5%-1.7%] | 1.3% [0.5%-1.9%] | 1.2% [0.5%-1.8%] | 0.4% [-0.1%-0.7%] | 1.7% [0.5%-2.8%] | 1.0% [0.6%-1.4%] | 0.6% [-0.0%-1.3%] | 2.0% [-0.0%-4.4%] | 1.3% [-0.0%-2.7%] | 0.1% [-0.0%-0.3%] | 0.2% [-0.0%-0.3%] | 0.2% [-0.0%-0.3%] | 0.2% [0.1%-0.3%] | 0.2% [0.1%-0.4%] | 0.2% [0.1%-0.3%] | 1.1% [0.0%-5.5%] | 0.7% [0.0%-3.8%] | 0.9% [0.0%-4.7%] | 1.0% [0.7%-1.2%] | 1.8% [1.3%-2.2%] | 1.4% [1.1%-1.6%] |
| 1997 | 5.7% [2.7%-10.5%] | 7.1% [2.1%-12.5%] | 6.3% [2.5%-11.4%] | 1.5% [0.8%-2.2%] | 0.6% [0.3%-0.8%] | 1.1% [0.6%-1.6%] | 1.3% [-0.3%-2.5%] | 1.1% [-0.3%-2.2%] | 1.2% [-0.3%-2.4%] | 1.1% [0.5%-1.7%] | 1.3% [0.5%-2.0%] | 1.2% [0.5%-1.8%] | 0.4% [-0.1%-0.7%] | 1.7% [0.5%-2.7%] | 1.0% [0.6%-1.4%] | 0.6% [-0.0%-1.3%] | 2.1% [-0.0%-4.3%] | 1.3% [-0.0%-2.7%] | 0.1% [-0.0%-0.3%] | 0.2% [-0.0%-0.3%] | 0.2% [-0.0%-0.3%] | 0.2% [0.1%-0.3%] | 0.2% [0.1%-0.3%] | 0.2% [0.1%-0.3%] | 1.1% [0.0%-5.5%] | 0.7% [0.0%-3.7%] | 0.9% [0.0%-4.7%] | 1.0% [0.7%-1.2%] | 1.8% [1.3%-2.2%] | 1.3% [1.1%-1.6%] |
| 1998 | 5.6% [2.6%-10.5%] | 7.1% [2.1%-12.6%] | 6.3% [2.4%-11.3%] | 1.5% [0.8%-2.2%] | 0.6% [0.3%-0.8%] | 1.1% [0.6%-1.6%] | 1.3% [-0.3%-2.5%] | 1.1% [-0.3%-2.2%] | 1.2% [-0.3%-2.4%] | 1.1% [0.5%-1.7%] | 1.3% [0.5%-2.0%] | 1.2% [0.5%-1.8%] | 0.4% [-0.0%-0.7%] | 1.7% [0.5%-2.8%] | 1.0% [0.6%-1.4%] | 0.7% [-0.0%-1.3%] | 2.1% [-0.0%-4.4%] | 1.3% [-0.0%-2.7%] | 0.1% [-0.0%-0.3%] | 0.2% [-0.0%-0.3%] | 0.2% [-0.0%-0.3%] | 0.2% [0.1%-0.3%] | 0.2% [0.1%-0.3%] | 0.2% [0.1%-0.3%] | 1.1% [0.0%-5.5%] | 0.7% [0.0%-3.7%] | 0.9% [0.0%-4.7%] | 1.0% [0.7%-1.2%] | 1.8% [1.3%-2.2%] | 1.3% [1.1%-1.6%] |
| 1999 | 5.6% [2.6%-10.3%] | 7.1% [2.1%-12.6%] | 6.3% [2.4%-11.3%] | 1.5% [0.8%-2.1%] | 0.6% [0.3%-0.8%] | 1.1% [0.6%-1.5%] | 1.3% [-0.3%-2.5%] | 1.1% [-0.3%-2.2%] | 1.2% [-0.3%-2.3%] | 1.1% [0.5%-1.7%] | 1.3% [0.5%-2.0%] | 1.2% [0.5%-1.8%] | 0.4% [-0.1%-0.7%] | 1.7% [0.5%-2.8%] | 1.0% [0.6%-1.4%] | 0.7% [-0.0%-1.4%] | 2.1% [-0.0%-4.4%] | 1.3% [-0.0%-2.8%] | 0.1% [-0.0%-0.3%] | 0.2% [-0.0%-0.4%] | 0.2% [-0.0%-0.3%] | 0.2% [0.1%-0.3%] | 0.2% [0.1%-0.3%] | 0.2% [0.1%-0.3%] | 1.0% [-0.0%-5.4%] | 0.7% [0.0%-3.7%] | 0.9% [0.0%-4.6%] | 1.0% [0.7%-1.2%] | 1.8% [1.3%-2.2%] | 1.3% [1.1%-1.6%] |
| 2000 | 5.6% [2.6%-10.3%] | 7.1% [2.1%-12.6%] | 6.3% [2.4%-11.2%] | 1.4% [0.7%-2.1%] | 0.6% [0.3%-0.8%] | 1.0% [0.5%-1.5%] | 1.3% [-0.3%-2.5%] | 1.1% [-0.3%-2.2%] | 1.2% [-0.3%-2.3%] | 1.1% [0.5%-1.7%] | 1.3% [0.5%-2.0%] | 1.2% [0.5%-1.8%] | 0.4% [-0.1%-0.7%] | 1.7% [0.5%-2.8%] | 1.0% [0.6%-1.4%] | 0.7% [-0.0%-1.4%] | 2.1% [-0.0%-4.5%] | 1.4% [-0.0%-2.8%] | 0.1% [-0.0%-0.3%] | 0.2% [-0.0%-0.4%] | 0.2% [-0.0%-0.3%] | 0.2% [0.1%-0.3%] | 0.2% [0.1%-0.3%] | 0.2% [0.1%-0.3%] | 1.0% [-0.0%-5.3%] | 0.7% [0.0%-3.6%] | 0.9% [0.0%-4.6%] | 1.0% [0.7%-1.2%] | 1.8% [1.3%-2.2%] | 1.3% [1.1%-1.6%] |
| 2001 | 5.6% [2.6%-10.3%] | 7.1% [2.1%-12.6%] | 6.3% [2.4%-11.2%] | 1.4% [0.7%-2.0%] | 0.6% [0.3%-0.8%] | 1.0% [0.5%-1.5%] | 1.3% [-0.3%-2.5%] | 1.1% [-0.3%-2.1%] | 1.2% [-0.3%-2.3%] | 1.2% [0.5%-1.7%] | 1.3% [0.5%-2.0%] | 1.2% [0.5%-1.8%] | 0.4% [-0.1%-0.7%] | 1.7% [0.5%-2.8%] | 1.0% [0.6%-1.4%] | 0.7% [-0.0%-1.4%] | 2.2% [-0.0%-4.6%] | 1.4% [-0.0%-2.8%] | 0.1% [-0.0%-0.3%] | 0.2% [-0.0%-0.4%] | 0.2% [-0.0%-0.3%] | 0.2% [0.1%-0.3%] | 0.2% [0.1%-0.3%] | 0.2% [0.1%-0.3%] | 1.0% [-0.0%-5.3%] | 0.7% [0.0%-3.6%] | 0.9% [0.0%-4.5%] | 1.0% [0.7%-1.2%] | 1.8% [1.3%-2.2%] | 1.3% [1.1%-1.6%] |
| 2002 | 5.5% [2.6%-10.2%] | 7.1% [2.1%-12.6%] | 6.3% [2.4%-11.2%] | 1.4% [0.7%-2.0%] | 0.6% [0.3%-0.8%] | 1.0% [0.5%-1.5%] | 1.3% [-0.3%-2.5%] | 1.1% [-0.3%-2.1%] | 1.2% [-0.3%-2.3%] | 1.2% [0.5%-1.8%] | 1.3% [0.6%-2.0%] | 1.2% [0.5%-1.9%] | 0.4% [-0.1%-0.7%] | 1.8% [0.5%-2.8%] | 1.1% [0.6%-1.4%] | 0.7% [-0.0%-1.4%] | 2.2% [-0.0%-4.7%] | 1.4% [-0.0%-2.9%] | 0.1% [-0.0%-0.3%] | 0.2% [-0.0%-0.4%] | 0.2% [-0.0%-0.3%] | 0.2% [0.1%-0.3%] | 0.2% [0.1%-0.3%] | 0.2% [0.1%-0.3%] | 1.0% [-0.0%-5.3%] | 0.7% [0.0%-3.6%] | 0.9% [0.0%-4.5%] | 1.0% [0.8%-1.2%] | 1.8% [1.3%-2.2%] | 1.3% [1.1%-1.6%] |
| 2003 | 5.5% [2.6%-10.2%] | 7.2% [2.2%-12.6%] | 6.3% [2.4%-11.2%] | 1.4% [0.7%-2.0%] | 0.6% [0.3%-0.8%] | 1.0% [0.5%-1.5%] | 1.3% [-0.3%-2.4%] | 1.1% [-0.3%-2.1%] | 1.2% [-0.3%-2.3%] | 1.2% [0.5%-1.8%] | 1.3% [0.6%-2.0%] | 1.3% [0.5%-1.9%] | 0.4% [-0.0%-0.8%] | 1.8% [0.5%-2.8%] | 1.1% [0.6%-1.4%] | 0.7% [-0.0%-1.4%] | 2.2% [-0.0%-4.7%] | 1.4% [-0.0%-2.9%] | 0.1% [-0.0%-0.3%] | 0.2% [-0.0%-0.4%] | 0.2% [-0.0%-0.3%] | 0.2% [0.1%-0.3%] | 0.2% [0.1%-0.3%] | 0.2% [0.1%-0.3%] | 1.0% [-0.0%-5.2%] | 0.7% [0.0%-3.5%] | 0.9% [0.0%-4.5%] | 1.0% [0.8%-1.2%] | 1.8% [1.3%-2.2%] | 1.3% [1.1%-1.6%] |
| 2004 | 5.5% [2.6%-10.1%] | 7.2% [2.2%-12.7%] | 6.3% [2.4%-11.2%] | 1.4% [0.7%-2.0%] | 0.6% [0.3%-0.8%] | 1.0% [0.5%-1.4%] | 1.2% [-0.3%-2.5%] | 1.0% [-0.2%-2.1%] | 1.2% [-0.3%-2.3%] | 1.2% [0.5%-1.8%] | 1.3% [0.6%-2.0%] | 1.3% [0.5%-1.9%] | 0.4% [0.0%-0.8%] | 1.8% [0.5%-2.8%] | 1.1% [0.6%-1.4%] | 0.7% [-0.0%-1.4%] | 2.3% [-0.0%-4.8%] | 1.4% [-0.0%-3.0%] | 0.2% [-0.0%-0.3%] | 0.2% [-0.0%-0.4%] | 0.2% [-0.0%-0.4%] | 0.2% [0.1%-0.3%] | 0.2% [0.1%-0.3%] | 0.2% [0.1%-0.3%] | 1.0% [-0.0%-5.2%] | 0.7% [0.0%-3.5%] | 0.9% [0.0%-4.4%] | 1.0% [0.8%-1.2%] | 1.7% [1.3%-2.2%] | 1.3% [1.1%-1.6%] |
| 2005 | 5.5% [2.6%-10.0%] | 7.2% [2.2%-12.6%] | 6.3% [2.4%-11.1%] | 1.3% [0.7%-1.9%] | 0.6% [0.3%-0.8%] | 1.0% [0.5%-1.4%] | 1.2% [-0.3%-2.5%] | 1.0% [-0.2%-2.1%] | 1.2% [-0.3%-2.3%] | 1.2% [0.5%-1.8%] | 1.3% [0.6%-2.0%] | 1.3% [0.5%-1.9%] | 0.4% [-0.0%-0.8%] | 1.8% [0.5%-2.8%] | 1.1% [0.6%-1.5%] | 0.7% [-0.0%-1.5%] | 2.3% [-0.0%-4.9%] | 1.5% [-0.0%-3.0%] | 0.2% [-0.0%-0.3%] | 0.2% [-0.0%-0.4%] | 0.2% [-0.0%-0.4%] | 0.2% [0.1%-0.3%] | 0.2% [0.1%-0.3%] | 0.2% [0.1%-0.3%] | 1.0% [-0.0%-5.1%] | 0.7% [0.0%-3.5%] | 0.9% [0.0%-4.4%] | 1.0% [0.8%-1.2%] | 1.7% [1.3%-2.2%] | 1.3% [1.1%-1.5%] |
| 2006 | 5.5% [2.6%-10.0%] | 7.2% [2.1%-12.6%] | 6.3% [2.4%-11.1%] | 1.3% [0.7%-1.9%] | 0.6% [0.3%-0.8%] | 1.0% [0.5%-1.4%] | 1.2% [-0.3%-2.4%] | 1.0% [-0.2%-2.1%] | 1.1% [-0.3%-2.2%] | 1.2% [0.5%-1.8%] | 1.3% [0.6%-2.0%] | 1.3% [0.5%-1.9%] | 0.4% [-0.0%-0.7%] | 1.8% [0.5%-2.8%] | 1.1% [0.6%-1.4%] | 0.7% [-0.0%-1.5%] | 2.3% [-0.0%-4.9%] | 1.5% [-0.0%-3.0%] | 0.2% [-0.0%-0.3%] | 0.2% [-0.0%-0.4%] | 0.2% [-0.0%-0.4%] | 0.2% [0.1%-0.3%] | 0.2% [0.1%-0.3%] | 0.2% [0.1%-0.3%] | 1.0% [-0.0%-5.1%] | 0.7% [0.0%-3.5%] | 0.8% [0.0%-4.4%] | 0.9% [0.7%-1.2%] | 1.7% [1.3%-2.1%] | 1.3% [1.1%-1.5%] |
| 2007 | 5.5% [2.6%-10.0%] | 7.2% [2.2%-12.6%] | 6.3% [2.4%-11.1%] | 1.3% [0.7%-1.9%] | 0.6% [0.3%-0.8%] | 1.0% [0.5%-1.4%] | 1.2% [-0.3%-2.4%] | 1.0% [-0.2%-2.0%] | 1.1% [-0.3%-2.2%] | 1.2% [0.5%-1.8%] | 1.3% [0.6%-2.0%] | 1.3% [0.5%-1.9%] | 0.5% [-0.0%-0.7%] | 1.8% [0.5%-2.8%] | 1.1% [0.6%-1.5%] | 0.7% [-0.0%-1.5%] | 2.3% [-0.0%-4.9%] | 1.5% [-0.0%-3.0%] | 0.2% [-0.0%-0.3%] | 0.2% [-0.0%-0.4%] | 0.2% [-0.0%-0.4%] | 0.2% [0.1%-0.3%] | 0.2% [0.1%-0.3%] | 0.2% [0.1%-0.3%] | 1.0% [-0.0%-5.1%] | 0.7% [0.0%-3.4%] | 0.9% [0.0%-4.3%] | 0.9% [0.7%-1.1%] | 1.7% [1.3%-2.1%] | 1.3% [1.1%-1.5%] |
| 2008 | 5.4% [2.6%-9.9%] | 7.2% [2.1%-12.6%] | 6.3% [2.4%-11.0%] | 1.3% [0.7%-1.9%] | 0.6% [0.3%-0.8%] | 1.0% [0.5%-1.4%] | 1.2% [-0.3%-2.4%] | 1.0% [-0.2%-2.0%] | 1.1% [-0.3%-2.2%] | 1.2% [0.5%-1.8%] | 1.3% [0.6%-2.0%] | 1.3% [0.5%-1.9%] | 0.5% [-0.0%-0.8%] | 1.8% [0.5%-2.8%] | 1.1% [0.6%-1.4%] | 0.7% [-0.0%-1.5%] | 2.4% [-0.0%-5.0%] | 1.5% [-0.0%-3.1%] | 0.2% [-0.0%-0.3%] | 0.2% [-0.0%-0.4%] | 0.2% [-0.0%-0.4%] | 0.2% [0.1%-0.3%] | 0.2% [0.1%-0.3%] | 0.2% [0.1%-0.3%] | 1.0% [0.0%-5.1%] | 0.7% [0.0%-3.4%] | 0.8% [0.0%-4.3%] | 0.9% [0.7%-1.1%] | 1.7% [1.2%-2.1%] | 1.3% [1.1%-1.5%] |
| 2009 | 5.4% [2.5%-9.9%] | 7.1% [2.1%-12.5%] | 6.2% [2.4%-11.0%] | 1.3% [0.7%-1.9%] | 0.6% [0.3%-0.8%] | 1.0% [0.5%-1.4%] | 1.2% [-0.3%-2.3%] | 1.0% [-0.2%-2.0%] | 1.1% [-0.3%-2.2%] | 1.2% [0.5%-1.8%] | 1.3% [0.6%-2.0%] | 1.3% [0.5%-1.9%] | 0.5% [0.0%-0.7%] | 1.8% [0.5%-2.8%] | 1.1% [0.6%-1.4%] | 0.7% [-0.0%-1.5%] | 2.4% [-0.0%-5.0%] | 1.5% [-0.0%-3.1%] | 0.2% [-0.0%-0.3%] | 0.2% [-0.0%-0.4%] | 0.2% [-0.0%-0.4%] | 0.2% [0.1%-0.3%] | 0.2% [0.1%-0.3%] | 0.2% [0.1%-0.3%] | 1.0% [0.0%-5.0%] | 0.7% [0.0%-3.4%] | 0.8% [0.0%-4.3%] | 0.9% [0.7%-1.1%] | 1.6% [1.2%-2.0%] | 1.3% [1.1%-1.5%] |
| 2010 | 5.4% [2.6%-9.9%] | 7.1% [2.1%-12.5%] | 6.2% [2.4%-11.0%] | 1.3% [0.7%-1.9%] | 0.6% [0.3%-0.8%] | 1.0% [0.5%-1.4%] | 1.2% [-0.3%-2.3%] | 1.0% [-0.2%-2.0%] | 1.1% [-0.2%-2.1%] | 1.2% [0.5%-1.8%] | 1.3% [0.5%-2.0%] | 1.3% [0.5%-1.9%] | 0.5% [0.0%-0.7%] | 1.8% [0.5%-2.8%] | 1.1% [0.6%-1.5%] | 0.7% [-0.0%-1.5%] | 2.4% [-0.0%-5.1%] | 1.5% [-0.0%-3.2%] | 0.2% [-0.0%-0.3%] | 0.2% [-0.0%-0.4%] | 0.2% [-0.0%-0.4%] | 0.2% [0.1%-0.2%] | 0.2% [0.1%-0.3%] | 0.2% [0.1%-0.3%] | 1.0% [0.0%-5.0%] | 0.7% [0.0%-3.4%] | 0.8% [0.0%-4.2%] | 0.9% [0.7%-1.1%] | 1.6% [1.2%-2.0%] | 1.2% [1.0%-1.4%] |
| 2011 | 5.4% [2.5%-9.9%] | 7.1% [2.1%-12.4%] | 6.2% [2.3%-10.9%] | 1.3% [0.7%-1.9%] | 0.6% [0.3%-0.8%] | 1.0% [0.5%-1.4%] | 1.2% [-0.3%-2.3%] | 1.0% [-0.2%-2.0%] | 1.1% [-0.2%-2.1%] | 1.2% [0.5%-1.8%] | 1.3% [0.5%-2.0%] | 1.3% [0.5%-1.9%] | 0.5% [0.0%-0.7%] | 1.8% [0.5%-2.8%] | 1.1% [0.6%-1.5%] | 0.8% [-0.0%-1.5%] | 2.4% [-0.0%-5.1%] | 1.5% [-0.0%-3.2%] | 0.2% [-0.0%-0.3%] | 0.2% [-0.0%-0.4%] | 0.2% [-0.0%-0.4%] | 0.2% [0.1%-0.2%] | 0.2% [0.1%-0.3%] | 0.2% [0.1%-0.2%] | 1.0% [0.0%-4.9%] | 0.6% [0.0%-3.3%] | 0.8% [0.0%-4.1%] | 0.9% [0.7%-1.1%] | 1.6% [1.2%-2.0%] | 1.2% [1.0%-1.4%] |
| 2012 | 5.3% [2.5%-9.7%] | 7.1% [2.1%-12.3%] | 6.2% [2.3%-10.9%] | 1.3% [0.7%-1.9%] | 0.6% [0.3%-0.9%] | 1.0% [0.5%-1.4%] | 1.2% [-0.3%-2.3%] | 0.9% [-0.2%-1.9%] | 1.1% [-0.2%-2.1%] | 1.2% [0.5%-1.8%] | 1.3% [0.5%-2.0%] | 1.3% [0.5%-1.9%] | 0.5% [-0.0%-0.7%] | 1.8% [0.5%-2.8%] | 1.1% [0.6%-1.5%] | 0.8% [-0.0%-1.6%] | 2.4% [-0.0%-5.1%] | 1.6% [-0.0%-3.2%] | 0.2% [-0.0%-0.3%] | 0.2% [-0.0%-0.4%] | 0.2% [-0.0%-0.4%] | 0.2% [0.1%-0.2%] | 0.2% [0.1%-0.3%] | 0.2% [0.1%-0.2%] | 0.9% [0.0%-4.8%] | 0.6% [0.0%-3.2%] | 0.8% [0.0%-4.1%] | 0.9% [0.7%-1.0%] | 1.6% [1.2%-1.9%] | 1.2% [1.0%-1.4%] |
| 2013 | 5.3% [2.5%-9.8%] | 7.1% [2.0%-12.3%] | 6.2% [2.3%-10.9%] | 1.3% [0.7%-1.8%] | 0.6% [0.3%-0.9%] | 1.0% [0.5%-1.4%] | 1.2% [-0.3%-2.3%] | 0.9% [-0.2%-1.9%] | 1.1% [-0.2%-2.1%] | 1.2% [0.5%-1.8%] | 1.3% [0.5%-2.0%] | 1.3% [0.5%-1.9%] | 0.5% [-0.0%-0.8%] | 1.8% [0.5%-2.8%] | 1.1% [0.6%-1.5%] | 0.8% [-0.0%-1.6%] | 2.5% [-0.0%-5.1%] | 1.6% [-0.0%-3.2%] | 0.2% [-0.0%-0.3%] | 0.2% [-0.0%-0.4%] | 0.2% [-0.0%-0.4%] | 0.2% [0.1%-0.2%] | 0.2% [0.1%-0.3%] | 0.2% [0.1%-0.2%] | 0.9% [0.0%-4.8%] | 0.6% [0.0%-3.3%] | 0.8% [0.0%-4.0%] | 0.9% [0.7%-1.0%] | 1.5% [1.2%-1.9%] | 1.2% [1.0%-1.4%] |
| 2014 | 5.3% [2.5%-9.6%] | 7.0% [2.0%-12.3%] | 6.2% [2.3%-10.8%] | 1.3% [0.7%-1.8%] | 0.6% [0.3%-0.9%] | 1.0% [0.5%-1.4%] | 1.2% [-0.3%-2.3%] | 0.9% [-0.2%-1.8%] | 1.0% [-0.2%-2.1%] | 1.2% [0.5%-1.9%] | 1.3% [0.5%-2.0%] | 1.3% [0.5%-1.9%] | 0.5% [-0.0%-0.8%] | 1.8% [0.5%-2.8%] | 1.1% [0.6%-1.5%] | 0.8% [-0.0%-1.6%] | 2.5% [-0.0%-5.2%] | 1.6% [-0.0%-3.3%] | 0.2% [-0.0%-0.4%] | 0.2% [-0.0%-0.4%] | 0.2% [-0.0%-0.4%] | 0.2% [0.1%-0.2%] | 0.2% [0.1%-0.2%] | 0.2% [0.1%-0.2%] | 0.9% [0.0%-4.7%] | 0.6% [0.0%-3.2%] | 0.8% [0.0%-4.0%] | 0.9% [0.7%-1.0%] | 1.5% [1.2%-1.9%] | 1.2% [1.0%-1.4%] |
| 2015 | 5.3% [2.5%-9.5%] | 7.0% [2.0%-12.3%] | 6.2% [2.3%-10.8%] | 1.3% [0.7%-1.8%] | 0.6% [0.3%-0.9%] | 1.0% [0.5%-1.4%] | 1.1% [-0.3%-2.3%] | 0.9% [-0.2%-1.8%] | 1.0% [-0.2%-2.1%] | 1.3% [0.5%-1.9%] | 1.3% [0.5%-2.0%] | 1.3% [0.5%-1.9%] | 0.5% [-0.0%-0.8%] | 1.8% [0.5%-2.8%] | 1.1% [0.6%-1.5%] | 0.8% [-0.0%-1.6%] | 2.5% [-0.0%-5.2%] | 1.6% [-0.0%-3.3%] | 0.2% [-0.0%-0.4%] | 0.2% [-0.0%-0.4%] | 0.2% [-0.0%-0.4%] | 0.1% [0.1%-0.2%] | 0.2% [0.1%-0.2%] | 0.2% [0.1%-0.2%] | 0.9% [0.0%-4.6%] | 0.6% [0.0%-3.1%] | 0.8% [0.0%-3.8%] | 0.9% [0.6%-1.0%] | 1.5% [1.1%-1.9%] | 1.2% [1.0%-1.4%] |
| 2016 | 5.3% [2.5%-9.5%] | 7.0% [2.0%-12.2%] | 6.2% [2.3%-10.8%] | 1.3% [0.7%-1.8%] | 0.6% [0.3%-0.9%] | 1.0% [0.5%-1.4%] | 1.1% [-0.3%-2.3%] | 0.9% [-0.2%-1.8%] | 1.0% [-0.2%-2.0%] | 1.3% [0.5%-1.9%] | 1.3% [0.5%-2.0%] | 1.3% [0.5%-1.9%] | 0.5% [0.0%-0.8%] | 1.8% [0.5%-2.8%] | 1.1% [0.6%-1.5%] | 0.8% [-0.0%-1.6%] | 2.5% [-0.0%-5.3%] | 1.6% [-0.0%-3.4%] | 0.2% [-0.0%-0.4%] | 0.2% [-0.0%-0.4%] | 0.2% [-0.0%-0.4%] | 0.1% [0.1%-0.2%] | 0.2% [0.1%-0.2%] | 0.2% [0.1%-0.2%] | 0.9% [0.0%-4.5%] | 0.6% [0.0%-3.1%] | 0.7% [0.0%-3.8%] | 0.8% [0.6%-1.0%] | 1.5% [1.1%-1.8%] | 1.2% [1.0%-1.4%] |
| 2017 | 5.3% [2.5%-9.5%] | 7.0% [2.0%-12.2%] | 6.2% [2.3%-10.8%] | 1.3% [0.7%-1.8%] | 0.6% [0.3%-0.9%] | 1.0% [0.5%-1.4%] | 1.1% [-0.3%-2.3%] | 0.9% [-0.2%-1.8%] | 1.0% [-0.2%-2.0%] | 1.3% [0.5%-1.9%] | 1.3% [0.6%-2.0%] | 1.3% [0.5%-1.9%] | 0.5% [0.0%-0.8%] | 1.8% [0.5%-2.8%] | 1.1% [0.6%-1.5%] | 0.8% [-0.0%-1.6%] | 2.5% [-0.0%-5.3%] | 1.6% [-0.0%-3.4%] | 0.2% [-0.0%-0.4%] | 0.2% [-0.0%-0.4%] | 0.2% [-0.0%-0.4%] | 0.2% [0.1%-0.2%] | 0.2% [0.1%-0.2%] | 0.2% [0.1%-0.2%] | 0.9% [0.0%-4.4%] | 0.6% [0.0%-3.0%] | 0.7% [0.0%-3.7%] | 0.8% [0.6%-1.0%] | 1.5% [1.1%-1.8%] | 1.2% [1.0%-1.4%] |
| 2018 | 5.3% [2.5%-9.5%] | 7.0% [2.0%-12.2%] | 6.2% [2.3%-10.7%] | 1.3% [0.7%-1.8%] | 0.7% [0.3%-0.9%] | 1.0% [0.5%-1.4%] | 1.1% [-0.3%-2.3%] | 0.9% [-0.2%-1.8%] | 1.0% [-0.2%-2.0%] | 1.3% [0.5%-1.9%] | 1.3% [0.6%-2.0%] | 1.3% [0.5%-1.9%] | 0.5% [0.1%-0.8%] | 1.8% [0.5%-2.8%] | 1.1% [0.6%-1.5%] | 0.8% [-0.0%-1.6%] | 2.5% [-0.0%-5.4%] | 1.6% [-0.0%-3.4%] | 0.2% [-0.0%-0.4%] | 0.2% [-0.0%-0.4%] | 0.2% [-0.0%-0.4%] | 0.2% [0.1%-0.2%] | 0.2% [0.1%-0.2%] | 0.2% [0.1%-0.2%] | 0.9% [0.0%-4.4%] | 0.6% [0.0%-3.0%] | 0.7% [0.0%-3.7%] | 0.8% [0.6%-1.0%] | 1.5% [1.1%-1.8%] | 1.2% [1.0%-1.3%] |
| 2019 | 5.3% [2.5%-9.5%] | 7.0% [2.0%-12.2%] | 6.2% [2.3%-10.7%] | 1.3% [0.7%-1.8%] | 0.7% [0.3%-0.9%] | 1.0% [0.5%-1.4%] | 1.1% [-0.3%-2.3%] | 0.9% [-0.2%-1.8%] | 1.0% [-0.2%-2.0%] | 1.3% [0.5%-1.9%] | 1.3% [0.5%-2.0%] | 1.3% [0.5%-1.9%] | 0.5% [0.1%-0.8%] | 1.8% [0.5%-2.8%] | 1.1% [0.6%-1.5%] | 0.8% [-0.0%-1.6%] | 2.6% [-0.0%-5.4%] | 1.7% [-0.0%-3.4%] | 0.2% [-0.0%-0.4%] | 0.2% [-0.0%-0.4%] | 0.2% [-0.0%-0.4%] | 0.2% [0.1%-0.2%] | 0.2% [0.1%-0.2%] | 0.2% [0.1%-0.2%] | 0.9% [0.0%-4.4%] | 0.6% [0.0%-2.9%] | 0.7% [0.0%-3.6%] | 0.8% [0.6%-1.0%] | 1.5% [1.1%-1.8%] | 1.2% [1.0%-1.3%] |
| 2020 | 5.3% [2.5%-9.5%] | 7.0% [2.0%-12.2%] | 6.2% [2.3%-10.6%] | 1.3% [0.7%-1.8%] | 0.7% [0.3%-0.9%] | 1.0% [0.5%-1.4%] | 1.1% [-0.3%-2.3%] | 0.9% [-0.2%-1.8%] | 1.0% [-0.2%-2.0%] | 1.3% [0.5%-1.9%] | 1.3% [0.5%-2.0%] | 1.3% [0.5%-1.9%] | 0.5% [0.1%-0.8%] | 1.8% [0.5%-2.8%] | 1.1% [0.6%-1.5%] | 0.8% [-0.0%-1.7%] | 2.6% [-0.0%-5.5%] | 1.7% [-0.0%-3.5%] | 0.2% [-0.0%-0.4%] | 0.2% [-0.0%-0.4%] | 0.2% [-0.0%-0.4%] | 0.2% [0.1%-0.2%] | 0.2% [0.1%-0.2%] | 0.2% [0.1%-0.2%] | 0.8% [0.0%-4.3%] | 0.6% [0.0%-2.8%] | 0.7% [0.0%-3.6%] | 0.8% [0.6%-1.0%] | 1.5% [1.1%-1.8%] | 1.1% [1.0%-1.3%] |
| 2021 | 5.3% [2.5%-9.3%] | 7.0% [2.0%-12.1%] | 6.2% [2.3%-10.6%] | 1.3% [0.7%-1.8%] | 0.7% [0.3%-0.9%] | 1.0% [0.5%-1.4%] | 1.1% [-0.3%-2.3%] | 0.9% [-0.2%-1.8%] | 1.0% [-0.2%-2.0%] | 1.3% [0.5%-1.9%] | 1.3% [0.5%-2.0%] | 1.3% [0.5%-1.9%] | 0.5% [0.0%-0.8%] | 1.8% [0.5%-2.8%] | 1.1% [0.6%-1.5%] | 0.8% [-0.0%-1.7%] | 2.6% [-0.0%-5.4%] | 1.7% [-0.0%-3.5%] | 0.2% [-0.0%-0.4%] | 0.2% [-0.0%-0.4%] | 0.2% [-0.0%-0.4%] | 0.2% [0.1%-0.2%] | 0.2% [0.1%-0.2%] | 0.2% [0.1%-0.2%] | 0.8% [0.0%-4.2%] | 0.5% [0.0%-2.8%] | 0.7% [0.0%-3.5%] | 0.9% [0.6%-1.0%] | 1.5% [1.1%-1.8%] | 1.2% [1.0%-1.3%] |
| **Note:** Data are presented as percentages (%) with 95% confidence intervals [lower-upper]. Values of "-0.0%" and "0.0%" result from rounding to one decimal place. "-0.0%" indicates that the original value was negative but rounded to zero (absolute value <0.05%), while "0.0%" indicates a positive value that rounded to zero (<0.05%). Negative values suggest an inverse relationship between the dietary factor and cancer mortality, while positive values indicate a positive association. All rates are age-standardized. | | | | | | | | | | | | | | | | | | | | | | | | | | | | | |  |

| Table S1f. Percentage of Total Cancer Mortality Attributable to Dietary Risk Factors - Low SDI Regions (Age-standardized, 1990-2021). | | | | | | | | | | | | | | | | | | | | | | | | | | | | | | |
| --- | --- | --- | --- | --- | --- | --- | --- | --- | --- | --- | --- | --- | --- | --- | --- | --- | --- | --- | --- | --- | --- | --- | --- | --- | --- | --- | --- | --- | --- | --- |
|  | All dietary risks | | | Low in fruits | | | Low in vegetables | | | Low in whole grains | | | Low in milk | | | High in red meat | | | High in processed meat | | | Low in fiber | | | High in sodium | | | Low in calcium | | |
| Year | Male | Female | Both | Male | Female | Both | Male | Female | Both | Male | Female | Both | Male | Female | Both | Male | Female | Both | Male | Female | Both | Male | Female | Both | Male | Female | Both | Male | Female | Both |
| 1990 | 6.2% [3.2%-11.0%] | 7.1% [1.8%-12.8%] | 6.7% [2.5%-11.7%] | 1.0% [0.5%-1.5%] | 0.3% [0.1%-0.4%] | 0.6% [0.3%-1.0%] | 2.0% [-0.5%-3.8%] | 1.7% [-0.4%-3.2%] | 1.9% [-0.5%-3.5%] | 1.4% [0.6%-2.2%] | 1.2% [0.5%-1.8%] | 1.3% [0.5%-2.0%] | 0.2% [-1.1%-1.2%] | 1.5% [0.4%-2.5%] | 0.9% [0.4%-1.2%] | 0.9% [-0.0%-1.9%] | 2.0% [-0.0%-4.3%] | 1.4% [-0.0%-3.1%] | 0.2% [-0.0%-0.5%] | 0.2% [-0.0%-0.4%] | 0.2% [-0.0%-0.4%] | 0.1% [0.0%-0.1%] | 0.1% [0.0%-0.1%] | 0.1% [0.0%-0.1%] | 1.0% [0.0%-5.6%] | 0.7% [0.0%-3.7%] | 0.9% [0.0%-4.7%] | 1.6% [1.0%-2.2%] | 1.8% [1.3%-2.3%] | 1.7% [1.3%-2.1%] |
| 1991 | 6.2% [3.2%-11.0%] | 7.1% [1.8%-12.8%] | 6.7% [2.5%-11.8%] | 1.0% [0.5%-1.5%] | 0.3% [0.1%-0.4%] | 0.6% [0.3%-1.0%] | 2.0% [-0.5%-3.8%] | 1.7% [-0.4%-3.2%] | 1.9% [-0.5%-3.5%] | 1.4% [0.6%-2.2%] | 1.2% [0.5%-1.8%] | 1.3% [0.5%-2.0%] | 0.2% [-1.1%-1.1%] | 1.5% [0.4%-2.5%] | 0.9% [0.4%-1.2%] | 0.9% [-0.0%-1.9%] | 2.0% [-0.0%-4.2%] | 1.4% [-0.0%-3.1%] | 0.2% [-0.0%-0.5%] | 0.2% [-0.0%-0.4%] | 0.2% [-0.0%-0.4%] | 0.1% [0.0%-0.1%] | 0.1% [0.0%-0.1%] | 0.1% [0.0%-0.1%] | 1.0% [0.0%-5.6%] | 0.7% [0.0%-3.7%] | 0.9% [0.0%-4.6%] | 1.6% [1.0%-2.2%] | 1.8% [1.3%-2.3%] | 1.7% [1.3%-2.1%] |
| 1992 | 6.2% [3.2%-10.8%] | 7.1% [1.8%-12.7%] | 6.7% [2.5%-11.6%] | 1.0% [0.5%-1.5%] | 0.3% [0.1%-0.4%] | 0.6% [0.3%-1.0%] | 2.0% [-0.5%-3.7%] | 1.7% [-0.4%-3.3%] | 1.9% [-0.5%-3.5%] | 1.4% [0.6%-2.2%] | 1.2% [0.5%-1.8%] | 1.3% [0.5%-2.0%] | 0.2% [-1.1%-1.1%] | 1.5% [0.4%-2.5%] | 0.9% [0.4%-1.2%] | 0.9% [-0.0%-1.9%] | 2.0% [-0.0%-4.3%] | 1.4% [-0.0%-3.1%] | 0.2% [-0.0%-0.5%] | 0.2% [-0.0%-0.4%] | 0.2% [-0.0%-0.4%] | 0.1% [0.0%-0.1%] | 0.1% [0.0%-0.1%] | 0.1% [0.0%-0.1%] | 1.0% [0.0%-5.5%] | 0.7% [0.0%-3.7%] | 0.9% [0.0%-4.6%] | 1.6% [1.0%-2.2%] | 1.8% [1.3%-2.3%] | 1.7% [1.3%-2.1%] |
| 1993 | 6.1% [3.2%-10.8%] | 7.1% [1.8%-12.7%] | 6.7% [2.5%-11.6%] | 1.0% [0.5%-1.4%] | 0.3% [0.1%-0.4%] | 0.6% [0.3%-0.9%] | 2.0% [-0.5%-3.7%] | 1.7% [-0.4%-3.3%] | 1.9% [-0.5%-3.5%] | 1.4% [0.6%-2.1%] | 1.2% [0.5%-1.8%] | 1.3% [0.5%-2.0%] | 0.2% [-1.1%-1.1%] | 1.5% [0.4%-2.5%] | 0.8% [0.4%-1.2%] | 0.9% [-0.0%-1.9%] | 2.0% [-0.0%-4.3%] | 1.4% [-0.0%-3.1%] | 0.2% [-0.0%-0.5%] | 0.2% [-0.0%-0.4%] | 0.2% [-0.0%-0.4%] | 0.1% [0.0%-0.1%] | 0.1% [0.0%-0.1%] | 0.1% [0.0%-0.1%] | 1.0% [0.0%-5.5%] | 0.7% [0.0%-3.6%] | 0.8% [0.0%-4.5%] | 1.6% [1.0%-2.2%] | 1.8% [1.3%-2.3%] | 1.7% [1.3%-2.1%] |
| 1994 | 6.1% [3.2%-10.8%] | 7.1% [1.8%-12.7%] | 6.6% [2.5%-11.5%] | 1.0% [0.5%-1.5%] | 0.3% [0.1%-0.4%] | 0.6% [0.3%-1.0%] | 2.0% [-0.5%-3.7%] | 1.7% [-0.4%-3.3%] | 1.8% [-0.5%-3.5%] | 1.4% [0.6%-2.2%] | 1.2% [0.5%-1.8%] | 1.3% [0.5%-2.0%] | 0.1% [-1.1%-1.2%] | 1.5% [0.4%-2.5%] | 0.8% [0.4%-1.2%] | 0.9% [-0.0%-1.9%] | 2.0% [-0.0%-4.2%] | 1.4% [-0.0%-3.0%] | 0.2% [-0.0%-0.5%] | 0.2% [-0.0%-0.4%] | 0.2% [-0.0%-0.4%] | 0.1% [0.0%-0.1%] | 0.1% [0.0%-0.1%] | 0.1% [0.0%-0.1%] | 1.0% [0.0%-5.4%] | 0.7% [0.0%-3.6%] | 0.8% [0.0%-4.5%] | 1.6% [1.0%-2.2%] | 1.9% [1.3%-2.3%] | 1.7% [1.3%-2.1%] |
| 1995 | 6.1% [3.2%-10.7%] | 7.1% [1.8%-12.7%] | 6.6% [2.5%-11.5%] | 1.0% [0.5%-1.5%] | 0.3% [0.1%-0.4%] | 0.6% [0.3%-0.9%] | 2.0% [-0.5%-3.7%] | 1.7% [-0.4%-3.3%] | 1.8% [-0.5%-3.4%] | 1.4% [0.6%-2.2%] | 1.2% [0.5%-1.8%] | 1.3% [0.5%-2.0%] | 0.1% [-1.2%-1.2%] | 1.5% [0.4%-2.5%] | 0.8% [0.4%-1.2%] | 0.9% [-0.0%-1.8%] | 2.0% [-0.0%-4.2%] | 1.4% [-0.0%-3.1%] | 0.2% [-0.0%-0.5%] | 0.2% [-0.0%-0.4%] | 0.2% [-0.0%-0.4%] | 0.1% [0.0%-0.1%] | 0.1% [0.0%-0.1%] | 0.1% [0.0%-0.1%] | 1.0% [0.0%-5.3%] | 0.7% [0.0%-3.6%] | 0.8% [0.0%-4.5%] | 1.6% [1.0%-2.1%] | 1.9% [1.3%-2.4%] | 1.7% [1.3%-2.1%] |
| 1996 | 6.0% [3.2%-10.6%] | 7.1% [1.9%-12.6%] | 6.6% [2.5%-11.4%] | 1.0% [0.5%-1.5%] | 0.3% [0.1%-0.4%] | 0.6% [0.3%-0.9%] | 2.0% [-0.5%-3.7%] | 1.7% [-0.4%-3.3%] | 1.8% [-0.5%-3.4%] | 1.4% [0.6%-2.2%] | 1.2% [0.5%-1.8%] | 1.3% [0.5%-2.0%] | 0.1% [-1.2%-1.2%] | 1.5% [0.4%-2.5%] | 0.8% [0.4%-1.2%] | 0.9% [-0.0%-1.8%] | 2.0% [-0.0%-4.3%] | 1.4% [-0.0%-3.1%] | 0.2% [-0.0%-0.5%] | 0.2% [-0.0%-0.4%] | 0.2% [-0.0%-0.4%] | 0.1% [0.0%-0.1%] | 0.1% [0.0%-0.1%] | 0.1% [0.0%-0.1%] | 1.0% [0.0%-5.3%] | 0.7% [0.0%-3.6%] | 0.8% [0.0%-4.4%] | 1.6% [1.0%-2.1%] | 1.9% [1.3%-2.4%] | 1.7% [1.3%-2.1%] |
| 1997 | 6.0% [3.2%-10.6%] | 7.1% [1.9%-12.6%] | 6.6% [2.5%-11.4%] | 1.0% [0.5%-1.4%] | 0.3% [0.1%-0.4%] | 0.6% [0.3%-0.9%] | 2.0% [-0.5%-3.7%] | 1.7% [-0.4%-3.3%] | 1.8% [-0.5%-3.4%] | 1.4% [0.6%-2.2%] | 1.2% [0.5%-1.8%] | 1.3% [0.5%-2.0%] | 0.1% [-1.1%-1.2%] | 1.5% [0.4%-2.5%] | 0.8% [0.4%-1.2%] | 0.9% [-0.0%-1.8%] | 2.0% [-0.0%-4.3%] | 1.4% [-0.0%-3.0%] | 0.2% [-0.0%-0.5%] | 0.2% [-0.0%-0.4%] | 0.2% [-0.0%-0.4%] | 0.1% [0.0%-0.1%] | 0.1% [0.0%-0.1%] | 0.1% [0.0%-0.1%] | 1.0% [0.0%-5.3%] | 0.7% [0.0%-3.6%] | 0.8% [0.0%-4.4%] | 1.5% [1.0%-2.1%] | 1.9% [1.3%-2.4%] | 1.7% [1.3%-2.1%] |
| 1998 | 6.0% [3.2%-10.5%] | 7.2% [1.9%-12.6%] | 6.6% [2.5%-11.4%] | 1.0% [0.5%-1.4%] | 0.3% [0.1%-0.4%] | 0.6% [0.3%-0.9%] | 2.0% [-0.5%-3.7%] | 1.7% [-0.4%-3.3%] | 1.8% [-0.5%-3.5%] | 1.4% [0.6%-2.2%] | 1.2% [0.5%-1.9%] | 1.3% [0.5%-2.0%] | 0.1% [-1.2%-1.1%] | 1.5% [0.4%-2.5%] | 0.8% [0.4%-1.2%] | 0.9% [-0.0%-1.8%] | 2.0% [-0.0%-4.3%] | 1.4% [-0.0%-3.0%] | 0.2% [-0.0%-0.5%] | 0.2% [-0.0%-0.4%] | 0.2% [-0.0%-0.4%] | 0.1% [0.0%-0.1%] | 0.1% [0.0%-0.1%] | 0.1% [0.0%-0.1%] | 1.0% [0.0%-5.2%] | 0.7% [0.0%-3.6%] | 0.8% [0.0%-4.4%] | 1.5% [1.0%-2.1%] | 1.9% [1.3%-2.4%] | 1.7% [1.3%-2.1%] |
| 1999 | 5.9% [3.2%-10.4%] | 7.2% [1.9%-12.6%] | 6.6% [2.5%-11.3%] | 1.0% [0.5%-1.4%] | 0.3% [0.1%-0.4%] | 0.6% [0.3%-0.9%] | 2.0% [-0.5%-3.7%] | 1.7% [-0.4%-3.3%] | 1.8% [-0.5%-3.5%] | 1.4% [0.6%-2.2%] | 1.2% [0.5%-1.8%] | 1.3% [0.5%-2.0%] | 0.1% [-1.3%-1.2%] | 1.5% [0.4%-2.5%] | 0.8% [0.4%-1.2%] | 0.9% [-0.0%-1.8%] | 2.0% [-0.0%-4.3%] | 1.4% [-0.0%-3.1%] | 0.2% [-0.0%-0.5%] | 0.2% [-0.0%-0.4%] | 0.2% [-0.0%-0.4%] | 0.1% [0.0%-0.1%] | 0.1% [0.0%-0.1%] | 0.1% [0.0%-0.1%] | 1.0% [0.0%-5.1%] | 0.6% [0.0%-3.5%] | 0.8% [0.0%-4.3%] | 1.5% [1.0%-2.1%] | 1.9% [1.3%-2.4%] | 1.7% [1.3%-2.1%] |
| 2000 | 5.9% [3.2%-10.2%] | 7.2% [1.9%-12.6%] | 6.5% [2.5%-11.2%] | 0.9% [0.5%-1.4%] | 0.3% [0.1%-0.4%] | 0.6% [0.3%-0.9%] | 2.0% [-0.5%-3.7%] | 1.7% [-0.4%-3.3%] | 1.8% [-0.5%-3.5%] | 1.4% [0.6%-2.1%] | 1.2% [0.5%-1.8%] | 1.3% [0.5%-2.0%] | 0.1% [-1.3%-1.2%] | 1.5% [0.4%-2.5%] | 0.8% [0.4%-1.2%] | 0.9% [-0.0%-1.8%] | 2.0% [-0.0%-4.4%] | 1.5% [-0.0%-3.1%] | 0.2% [-0.0%-0.5%] | 0.2% [-0.0%-0.4%] | 0.2% [-0.0%-0.4%] | 0.1% [0.0%-0.1%] | 0.1% [0.0%-0.1%] | 0.1% [0.0%-0.1%] | 1.0% [0.0%-5.1%] | 0.6% [0.0%-3.5%] | 0.8% [0.0%-4.3%] | 1.5% [1.0%-2.0%] | 1.9% [1.3%-2.4%] | 1.7% [1.3%-2.1%] |
| 2001 | 5.8% [3.2%-10.2%] | 7.2% [1.9%-12.7%] | 6.5% [2.5%-11.2%] | 0.9% [0.5%-1.4%] | 0.3% [0.2%-0.4%] | 0.6% [0.3%-0.9%] | 2.0% [-0.5%-3.7%] | 1.7% [-0.4%-3.3%] | 1.8% [-0.5%-3.5%] | 1.4% [0.6%-2.1%] | 1.2% [0.5%-1.9%] | 1.3% [0.5%-2.0%] | 0.1% [-1.3%-1.2%] | 1.5% [0.4%-2.5%] | 0.8% [0.4%-1.1%] | 0.9% [-0.0%-1.8%] | 2.1% [-0.0%-4.5%] | 1.5% [-0.0%-3.1%] | 0.2% [-0.0%-0.5%] | 0.2% [-0.0%-0.4%] | 0.2% [-0.0%-0.4%] | 0.1% [0.0%-0.1%] | 0.1% [0.0%-0.1%] | 0.1% [0.0%-0.1%] | 1.0% [0.0%-5.1%] | 0.6% [0.0%-3.5%] | 0.8% [0.0%-4.3%] | 1.5% [1.0%-2.0%] | 1.8% [1.3%-2.4%] | 1.7% [1.3%-2.0%] |
| 2002 | 5.8% [3.2%-10.2%] | 7.2% [1.9%-12.7%] | 6.5% [2.5%-11.2%] | 0.9% [0.5%-1.4%] | 0.3% [0.2%-0.4%] | 0.6% [0.3%-0.9%] | 1.9% [-0.5%-3.7%] | 1.7% [-0.4%-3.2%] | 1.8% [-0.4%-3.4%] | 1.4% [0.6%-2.1%] | 1.2% [0.5%-1.9%] | 1.3% [0.5%-2.0%] | 0.1% [-1.4%-1.2%] | 1.5% [0.4%-2.5%] | 0.8% [0.4%-1.2%] | 0.9% [-0.0%-1.8%] | 2.1% [-0.0%-4.4%] | 1.5% [-0.0%-3.1%] | 0.2% [-0.0%-0.5%] | 0.2% [-0.0%-0.4%] | 0.2% [-0.0%-0.4%] | 0.1% [0.0%-0.1%] | 0.1% [0.0%-0.1%] | 0.1% [0.0%-0.1%] | 0.9% [0.0%-5.0%] | 0.6% [0.0%-3.5%] | 0.8% [0.0%-4.2%] | 1.5% [1.0%-2.0%] | 1.9% [1.3%-2.4%] | 1.7% [1.3%-2.0%] |
| 2003 | 5.8% [3.2%-10.1%] | 7.2% [1.9%-12.8%] | 6.5% [2.5%-11.3%] | 0.9% [0.5%-1.4%] | 0.3% [0.2%-0.4%] | 0.6% [0.3%-0.9%] | 1.9% [-0.5%-3.7%] | 1.7% [-0.4%-3.2%] | 1.8% [-0.4%-3.4%] | 1.4% [0.6%-2.1%] | 1.2% [0.5%-1.9%] | 1.3% [0.5%-2.0%] | 0.1% [-1.4%-1.2%] | 1.6% [0.4%-2.5%] | 0.8% [0.4%-1.2%] | 0.9% [-0.0%-1.9%] | 2.1% [-0.0%-4.5%] | 1.5% [-0.0%-3.1%] | 0.2% [-0.1%-0.5%] | 0.2% [-0.0%-0.4%] | 0.2% [-0.1%-0.4%] | 0.1% [0.0%-0.1%] | 0.1% [0.0%-0.1%] | 0.1% [0.0%-0.1%] | 0.9% [0.0%-5.0%] | 0.6% [0.0%-3.5%] | 0.8% [0.0%-4.2%] | 1.5% [1.0%-2.0%] | 1.8% [1.3%-2.3%] | 1.7% [1.4%-2.0%] |
| 2004 | 5.8% [3.2%-10.1%] | 7.2% [1.9%-12.8%] | 6.5% [2.5%-11.2%] | 0.9% [0.5%-1.4%] | 0.3% [0.2%-0.4%] | 0.6% [0.3%-0.9%] | 1.9% [-0.5%-3.7%] | 1.7% [-0.4%-3.2%] | 1.8% [-0.4%-3.4%] | 1.4% [0.6%-2.1%] | 1.2% [0.5%-1.9%] | 1.3% [0.5%-2.0%] | 0.1% [-1.4%-1.2%] | 1.6% [0.4%-2.5%] | 0.8% [0.4%-1.2%] | 0.9% [-0.0%-1.9%] | 2.1% [-0.0%-4.5%] | 1.5% [-0.0%-3.2%] | 0.2% [-0.1%-0.5%] | 0.2% [-0.1%-0.4%] | 0.2% [-0.1%-0.5%] | 0.1% [0.0%-0.1%] | 0.1% [0.0%-0.1%] | 0.1% [0.0%-0.1%] | 0.9% [0.0%-4.9%] | 0.6% [0.0%-3.5%] | 0.8% [0.0%-4.2%] | 1.5% [1.0%-2.0%] | 1.8% [1.3%-2.3%] | 1.7% [1.3%-2.0%] |
| 2005 | 5.8% [3.2%-10.0%] | 7.2% [1.9%-12.8%] | 6.5% [2.5%-11.1%] | 0.9% [0.5%-1.4%] | 0.3% [0.2%-0.4%] | 0.6% [0.3%-0.9%] | 1.9% [-0.5%-3.7%] | 1.7% [-0.4%-3.2%] | 1.8% [-0.4%-3.4%] | 1.4% [0.6%-2.1%] | 1.2% [0.5%-1.8%] | 1.3% [0.6%-2.0%] | 0.1% [-1.3%-1.2%] | 1.6% [0.4%-2.5%] | 0.8% [0.4%-1.2%] | 0.9% [-0.0%-1.9%] | 2.1% [-0.0%-4.6%] | 1.5% [-0.0%-3.2%] | 0.2% [-0.1%-0.5%] | 0.2% [-0.1%-0.4%] | 0.2% [-0.1%-0.5%] | 0.1% [0.0%-0.1%] | 0.1% [0.0%-0.1%] | 0.1% [0.0%-0.1%] | 0.9% [0.0%-4.9%] | 0.6% [0.0%-3.5%] | 0.8% [0.0%-4.2%] | 1.4% [1.0%-2.0%] | 1.8% [1.4%-2.3%] | 1.6% [1.3%-2.0%] |
| 2006 | 5.7% [3.1%-9.9%] | 7.2% [1.9%-12.8%] | 6.5% [2.6%-11.1%] | 0.9% [0.5%-1.4%] | 0.3% [0.2%-0.4%] | 0.6% [0.3%-0.9%] | 1.9% [-0.5%-3.6%] | 1.6% [-0.4%-3.1%] | 1.8% [-0.4%-3.4%] | 1.4% [0.6%-2.2%] | 1.2% [0.5%-1.8%] | 1.3% [0.6%-2.0%] | 0.1% [-1.5%-1.2%] | 1.6% [0.4%-2.5%] | 0.8% [0.4%-1.1%] | 0.9% [-0.0%-1.9%] | 2.2% [-0.0%-4.6%] | 1.5% [-0.0%-3.2%] | 0.2% [-0.1%-0.5%] | 0.2% [-0.1%-0.4%] | 0.2% [-0.1%-0.5%] | 0.1% [0.0%-0.1%] | 0.1% [0.0%-0.1%] | 0.1% [0.0%-0.1%] | 0.9% [0.0%-4.9%] | 0.6% [0.0%-3.4%] | 0.8% [0.0%-4.1%] | 1.4% [0.9%-1.9%] | 1.8% [1.3%-2.3%] | 1.6% [1.3%-2.0%] |
| 2007 | 5.7% [3.1%-9.8%] | 7.2% [1.9%-12.8%] | 6.5% [2.6%-11.1%] | 0.9% [0.5%-1.4%] | 0.3% [0.2%-0.4%] | 0.6% [0.3%-0.9%] | 1.9% [-0.5%-3.6%] | 1.6% [-0.4%-3.1%] | 1.8% [-0.4%-3.4%] | 1.4% [0.6%-2.1%] | 1.2% [0.5%-1.8%] | 1.3% [0.6%-2.0%] | 0.1% [-1.5%-1.2%] | 1.6% [0.4%-2.5%] | 0.8% [0.4%-1.2%] | 0.9% [-0.0%-1.9%] | 2.2% [-0.0%-4.6%] | 1.5% [-0.0%-3.2%] | 0.2% [-0.1%-0.5%] | 0.2% [-0.1%-0.4%] | 0.2% [-0.1%-0.5%] | 0.1% [0.0%-0.1%] | 0.1% [0.0%-0.1%] | 0.1% [0.0%-0.1%] | 0.9% [0.0%-4.9%] | 0.6% [0.0%-3.4%] | 0.8% [0.0%-4.1%] | 1.4% [0.9%-1.9%] | 1.8% [1.3%-2.2%] | 1.6% [1.3%-1.9%] |
| 2008 | 5.6% [3.0%-9.8%] | 7.2% [1.9%-12.8%] | 6.4% [2.5%-11.0%] | 0.9% [0.5%-1.4%] | 0.3% [0.2%-0.4%] | 0.6% [0.3%-0.9%] | 1.9% [-0.5%-3.6%] | 1.6% [-0.3%-3.1%] | 1.7% [-0.4%-3.3%] | 1.4% [0.6%-2.2%] | 1.2% [0.5%-1.8%] | 1.3% [0.6%-2.0%] | 0.0% [-1.6%-1.2%] | 1.6% [0.4%-2.5%] | 0.8% [0.3%-1.1%] | 0.9% [-0.0%-1.9%] | 2.2% [-0.0%-4.6%] | 1.5% [-0.0%-3.3%] | 0.2% [-0.1%-0.5%] | 0.2% [-0.1%-0.4%] | 0.2% [-0.1%-0.5%] | 0.1% [0.0%-0.1%] | 0.1% [0.0%-0.1%] | 0.1% [0.0%-0.1%] | 0.9% [0.0%-4.9%] | 0.6% [0.0%-3.4%] | 0.8% [0.0%-4.1%] | 1.4% [0.9%-1.9%] | 1.8% [1.3%-2.2%] | 1.6% [1.3%-1.9%] |
| 2009 | 5.6% [3.1%-9.7%] | 7.2% [1.9%-12.7%] | 6.4% [2.5%-11.0%] | 0.9% [0.5%-1.3%] | 0.3% [0.2%-0.5%] | 0.6% [0.3%-0.9%] | 1.9% [-0.5%-3.6%] | 1.6% [-0.3%-3.1%] | 1.7% [-0.4%-3.3%] | 1.4% [0.6%-2.1%] | 1.2% [0.5%-1.8%] | 1.3% [0.6%-2.0%] | 0.1% [-1.5%-1.2%] | 1.6% [0.4%-2.4%] | 0.8% [0.3%-1.1%] | 0.9% [-0.0%-1.9%] | 2.2% [-0.0%-4.7%] | 1.6% [-0.0%-3.3%] | 0.2% [-0.1%-0.5%] | 0.2% [-0.1%-0.4%] | 0.2% [-0.1%-0.5%] | 0.1% [0.0%-0.1%] | 0.1% [0.0%-0.1%] | 0.1% [0.0%-0.1%] | 0.9% [0.0%-4.8%] | 0.6% [0.0%-3.3%] | 0.8% [0.0%-4.1%] | 1.3% [0.9%-1.8%] | 1.8% [1.3%-2.2%] | 1.6% [1.3%-1.9%] |
| 2010 | 5.6% [3.0%-9.7%] | 7.2% [1.8%-12.7%] | 6.4% [2.5%-10.9%] | 0.9% [0.5%-1.3%] | 0.3% [0.2%-0.5%] | 0.6% [0.3%-0.9%] | 1.8% [-0.5%-3.5%] | 1.6% [-0.3%-3.0%] | 1.7% [-0.4%-3.3%] | 1.4% [0.6%-2.1%] | 1.2% [0.5%-1.8%] | 1.3% [0.6%-2.0%] | 0.1% [-1.6%-1.2%] | 1.5% [0.4%-2.4%] | 0.8% [0.3%-1.1%] | 0.9% [-0.0%-1.9%] | 2.3% [-0.0%-4.7%] | 1.6% [-0.0%-3.3%] | 0.2% [-0.1%-0.5%] | 0.2% [-0.1%-0.4%] | 0.2% [-0.1%-0.5%] | 0.1% [0.0%-0.1%] | 0.1% [0.0%-0.1%] | 0.1% [0.0%-0.1%] | 0.9% [0.0%-4.8%] | 0.6% [0.0%-3.3%] | 0.8% [0.0%-4.1%] | 1.3% [0.9%-1.8%] | 1.7% [1.3%-2.2%] | 1.5% [1.3%-1.9%] |
| 2011 | 5.5% [3.1%-9.7%] | 7.2% [1.8%-12.7%] | 6.4% [2.5%-11.0%] | 0.9% [0.5%-1.3%] | 0.3% [0.2%-0.5%] | 0.6% [0.3%-0.9%] | 1.8% [-0.5%-3.5%] | 1.6% [-0.3%-3.0%] | 1.7% [-0.4%-3.3%] | 1.4% [0.6%-2.1%] | 1.2% [0.5%-1.8%] | 1.3% [0.6%-2.0%] | 0.1% [-1.5%-1.2%] | 1.5% [0.4%-2.4%] | 0.8% [0.4%-1.1%] | 0.9% [-0.0%-1.9%] | 2.3% [-0.0%-4.8%] | 1.6% [-0.0%-3.3%] | 0.2% [-0.1%-0.5%] | 0.2% [-0.1%-0.4%] | 0.2% [-0.1%-0.5%] | 0.1% [0.0%-0.1%] | 0.1% [0.0%-0.1%] | 0.1% [0.0%-0.1%] | 0.9% [0.0%-4.8%] | 0.6% [0.0%-3.3%] | 0.8% [0.0%-4.0%] | 1.3% [0.8%-1.8%] | 1.7% [1.3%-2.1%] | 1.5% [1.3%-1.8%] |
| 2012 | 5.5% [2.9%-9.6%] | 7.2% [1.8%-12.7%] | 6.4% [2.5%-10.9%] | 0.9% [0.5%-1.3%] | 0.3% [0.2%-0.5%] | 0.6% [0.3%-0.9%] | 1.8% [-0.5%-3.5%] | 1.5% [-0.3%-2.9%] | 1.7% [-0.4%-3.2%] | 1.4% [0.6%-2.1%] | 1.2% [0.5%-1.8%] | 1.3% [0.6%-2.0%] | 0.0% [-1.5%-1.2%] | 1.5% [0.4%-2.4%] | 0.8% [0.3%-1.1%] | 0.9% [-0.0%-1.9%] | 2.3% [-0.0%-4.8%] | 1.6% [-0.0%-3.3%] | 0.2% [-0.1%-0.5%] | 0.2% [-0.1%-0.4%] | 0.2% [-0.1%-0.5%] | 0.1% [0.0%-0.1%] | 0.1% [0.0%-0.1%] | 0.1% [0.0%-0.1%] | 0.9% [0.0%-4.7%] | 0.6% [0.0%-3.2%] | 0.7% [0.0%-4.0%] | 1.3% [0.8%-1.8%] | 1.7% [1.3%-2.1%] | 1.5% [1.3%-1.8%] |
| 2013 | 5.5% [3.0%-9.6%] | 7.2% [1.8%-12.7%] | 6.4% [2.5%-10.9%] | 0.9% [0.5%-1.3%] | 0.3% [0.2%-0.5%] | 0.6% [0.3%-0.9%] | 1.8% [-0.5%-3.5%] | 1.5% [-0.3%-2.9%] | 1.7% [-0.4%-3.2%] | 1.4% [0.6%-2.1%] | 1.2% [0.5%-1.8%] | 1.3% [0.6%-2.0%] | 0.1% [-1.5%-1.2%] | 1.5% [0.4%-2.4%] | 0.8% [0.4%-1.1%] | 0.9% [-0.0%-1.9%] | 2.3% [-0.0%-4.8%] | 1.6% [-0.0%-3.3%] | 0.2% [-0.1%-0.5%] | 0.2% [-0.1%-0.4%] | 0.2% [-0.1%-0.5%] | 0.1% [0.0%-0.1%] | 0.1% [0.0%-0.1%] | 0.1% [0.0%-0.1%] | 0.9% [0.0%-4.7%] | 0.6% [0.0%-3.3%] | 0.7% [0.0%-4.0%] | 1.3% [0.9%-1.8%] | 1.7% [1.3%-2.1%] | 1.5% [1.3%-1.8%] |
| 2014 | 5.5% [3.1%-9.4%] | 7.2% [1.8%-12.7%] | 6.4% [2.5%-10.9%] | 0.9% [0.5%-1.3%] | 0.3% [0.2%-0.5%] | 0.6% [0.3%-0.9%] | 1.8% [-0.4%-3.5%] | 1.5% [-0.3%-2.9%] | 1.7% [-0.4%-3.2%] | 1.4% [0.6%-2.2%] | 1.2% [0.5%-1.8%] | 1.3% [0.6%-2.0%] | 0.1% [-1.6%-1.2%] | 1.5% [0.4%-2.5%] | 0.8% [0.4%-1.1%] | 0.9% [-0.0%-1.9%] | 2.3% [-0.0%-4.9%] | 1.6% [-0.0%-3.4%] | 0.2% [-0.1%-0.5%] | 0.2% [-0.0%-0.4%] | 0.2% [-0.1%-0.5%] | 0.1% [0.0%-0.1%] | 0.1% [0.0%-0.1%] | 0.1% [0.0%-0.1%] | 0.9% [0.0%-4.6%] | 0.6% [0.0%-3.2%] | 0.7% [0.0%-4.0%] | 1.3% [0.8%-1.8%] | 1.7% [1.3%-2.0%] | 1.5% [1.3%-1.8%] |
| 2015 | 5.5% [3.0%-9.4%] | 7.2% [1.8%-12.7%] | 6.4% [2.5%-10.8%] | 0.9% [0.5%-1.3%] | 0.3% [0.2%-0.5%] | 0.6% [0.3%-0.9%] | 1.8% [-0.4%-3.5%] | 1.5% [-0.3%-2.9%] | 1.6% [-0.4%-3.2%] | 1.5% [0.6%-2.2%] | 1.2% [0.5%-1.8%] | 1.3% [0.6%-2.0%] | 0.1% [-1.6%-1.3%] | 1.6% [0.4%-2.5%] | 0.8% [0.4%-1.2%] | 0.9% [-0.0%-2.0%] | 2.3% [-0.0%-4.9%] | 1.6% [-0.0%-3.4%] | 0.2% [-0.1%-0.5%] | 0.2% [-0.0%-0.4%] | 0.2% [-0.1%-0.5%] | 0.1% [0.0%-0.1%] | 0.1% [0.0%-0.1%] | 0.1% [0.0%-0.1%] | 0.9% [0.0%-4.6%] | 0.6% [0.0%-3.2%] | 0.7% [0.0%-3.9%] | 1.3% [0.8%-1.8%] | 1.7% [1.3%-2.0%] | 1.5% [1.3%-1.8%] |
| 2016 | 5.5% [3.0%-9.4%] | 7.2% [1.9%-12.7%] | 6.4% [2.5%-10.8%] | 0.9% [0.5%-1.3%] | 0.3% [0.2%-0.5%] | 0.6% [0.3%-0.9%] | 1.8% [-0.4%-3.4%] | 1.5% [-0.3%-2.8%] | 1.6% [-0.4%-3.1%] | 1.5% [0.6%-2.2%] | 1.2% [0.5%-1.8%] | 1.3% [0.6%-2.0%] | 0.1% [-1.6%-1.3%] | 1.6% [0.4%-2.5%] | 0.8% [0.4%-1.1%] | 0.9% [-0.0%-2.0%] | 2.4% [-0.0%-5.0%] | 1.7% [-0.0%-3.5%] | 0.2% [-0.1%-0.5%] | 0.2% [-0.0%-0.4%] | 0.2% [-0.1%-0.5%] | 0.1% [0.0%-0.1%] | 0.1% [0.0%-0.1%] | 0.1% [0.0%-0.1%] | 0.8% [0.0%-4.5%] | 0.6% [0.0%-3.1%] | 0.7% [0.0%-3.8%] | 1.3% [0.8%-1.8%] | 1.7% [1.3%-2.0%] | 1.5% [1.3%-1.8%] |
| 2017 | 5.5% [3.1%-9.3%] | 7.2% [1.9%-12.7%] | 6.4% [2.5%-10.7%] | 0.9% [0.5%-1.3%] | 0.3% [0.2%-0.5%] | 0.6% [0.3%-0.9%] | 1.8% [-0.4%-3.4%] | 1.5% [-0.3%-2.8%] | 1.6% [-0.4%-3.1%] | 1.5% [0.6%-2.2%] | 1.2% [0.5%-1.8%] | 1.3% [0.6%-2.0%] | 0.1% [-1.6%-1.2%] | 1.6% [0.5%-2.5%] | 0.8% [0.4%-1.2%] | 1.0% [-0.0%-2.0%] | 2.4% [-0.0%-5.1%] | 1.7% [-0.0%-3.5%] | 0.2% [-0.1%-0.5%] | 0.2% [-0.0%-0.4%] | 0.2% [-0.1%-0.5%] | 0.1% [0.0%-0.1%] | 0.1% [0.0%-0.1%] | 0.1% [0.0%-0.1%] | 0.8% [0.0%-4.4%] | 0.6% [0.0%-3.1%] | 0.7% [0.0%-3.7%] | 1.3% [0.8%-1.8%] | 1.7% [1.3%-2.0%] | 1.5% [1.3%-1.8%] |
| 2018 | 5.5% [3.2%-9.3%] | 7.2% [1.9%-12.8%] | 6.4% [2.5%-10.7%] | 0.9% [0.5%-1.3%] | 0.4% [0.2%-0.5%] | 0.6% [0.3%-0.9%] | 1.8% [-0.4%-3.5%] | 1.5% [-0.3%-2.8%] | 1.6% [-0.4%-3.2%] | 1.5% [0.6%-2.2%] | 1.2% [0.5%-1.8%] | 1.3% [0.6%-2.0%] | 0.1% [-1.6%-1.3%] | 1.6% [0.5%-2.5%] | 0.8% [0.4%-1.2%] | 1.0% [-0.0%-2.0%] | 2.4% [-0.0%-5.1%] | 1.7% [-0.0%-3.5%] | 0.2% [-0.1%-0.5%] | 0.2% [-0.0%-0.4%] | 0.2% [-0.1%-0.5%] | 0.1% [0.0%-0.1%] | 0.1% [0.0%-0.1%] | 0.1% [0.0%-0.1%] | 0.8% [0.0%-4.3%] | 0.6% [0.0%-3.0%] | 0.7% [0.0%-3.7%] | 1.3% [0.8%-1.8%] | 1.7% [1.3%-2.0%] | 1.5% [1.3%-1.8%] |
| 2019 | 5.5% [3.1%-9.1%] | 7.2% [1.9%-12.8%] | 6.4% [2.6%-10.7%] | 0.9% [0.5%-1.3%] | 0.4% [0.2%-0.5%] | 0.6% [0.3%-0.9%] | 1.8% [-0.4%-3.5%] | 1.5% [-0.3%-2.8%] | 1.6% [-0.4%-3.2%] | 1.5% [0.6%-2.2%] | 1.2% [0.5%-1.8%] | 1.4% [0.6%-2.0%] | 0.1% [-1.5%-1.3%] | 1.6% [0.5%-2.5%] | 0.8% [0.4%-1.2%] | 1.0% [-0.0%-2.0%] | 2.5% [-0.0%-5.2%] | 1.7% [-0.0%-3.6%] | 0.2% [-0.1%-0.5%] | 0.2% [-0.0%-0.4%] | 0.2% [-0.1%-0.5%] | 0.1% [0.0%-0.1%] | 0.1% [0.0%-0.1%] | 0.1% [0.0%-0.1%] | 0.8% [0.0%-4.3%] | 0.6% [0.0%-3.0%] | 0.7% [0.0%-3.7%] | 1.3% [0.8%-1.8%] | 1.7% [1.3%-2.0%] | 1.5% [1.3%-1.8%] |
| 2020 | 5.5% [3.1%-9.0%] | 7.2% [1.8%-12.8%] | 6.4% [2.6%-10.8%] | 0.9% [0.5%-1.3%] | 0.4% [0.2%-0.5%] | 0.6% [0.3%-0.9%] | 1.8% [-0.4%-3.5%] | 1.5% [-0.3%-2.8%] | 1.6% [-0.4%-3.1%] | 1.5% [0.6%-2.2%] | 1.2% [0.5%-1.8%] | 1.4% [0.6%-2.0%] | 0.1% [-1.5%-1.3%] | 1.6% [0.5%-2.5%] | 0.8% [0.5%-1.2%] | 1.0% [-0.0%-2.0%] | 2.5% [-0.0%-5.2%] | 1.7% [-0.0%-3.6%] | 0.2% [-0.1%-0.5%] | 0.2% [-0.1%-0.4%] | 0.2% [-0.1%-0.5%] | 0.1% [0.0%-0.1%] | 0.1% [0.0%-0.1%] | 0.1% [0.0%-0.1%] | 0.8% [0.0%-4.3%] | 0.5% [0.0%-3.0%] | 0.7% [0.0%-3.6%] | 1.3% [0.8%-1.7%] | 1.6% [1.3%-2.0%] | 1.5% [1.3%-1.8%] |
| 2021 | 5.5% [3.1%-9.1%] | 7.2% [1.9%-12.8%] | 6.4% [2.5%-10.8%] | 0.9% [0.5%-1.3%] | 0.4% [0.2%-0.5%] | 0.6% [0.3%-0.9%] | 1.8% [-0.4%-3.5%] | 1.4% [-0.3%-2.8%] | 1.6% [-0.4%-3.2%] | 1.5% [0.7%-2.3%] | 1.2% [0.5%-1.8%] | 1.4% [0.6%-2.0%] | 0.1% [-1.5%-1.3%] | 1.6% [0.5%-2.5%] | 0.9% [0.5%-1.2%] | 1.0% [-0.0%-2.0%] | 2.5% [-0.0%-5.2%] | 1.8% [-0.0%-3.7%] | 0.2% [-0.1%-0.5%] | 0.2% [-0.1%-0.4%] | 0.2% [-0.1%-0.5%] | 0.1% [0.0%-0.1%] | 0.1% [0.0%-0.1%] | 0.1% [0.0%-0.1%] | 0.8% [0.0%-4.2%] | 0.5% [0.0%-3.0%] | 0.7% [0.0%-3.6%] | 1.3% [0.8%-1.8%] | 1.6% [1.3%-2.0%] | 1.5% [1.3%-1.8%] |
| **Note:** Data are presented as percentages (%) with 95% confidence intervals [lower-upper]. Values of "-0.0%" and "0.0%" result from rounding to one decimal place. "-0.0%" indicates that the original value was negative but rounded to zero (absolute value <0.05%), while "0.0%" indicates a positive value that rounded to zero (<0.05%). Negative values suggest an inverse relationship between the dietary factor and cancer mortality, while positive values indicate a positive association. All rates are age-standardized. | | | | | | | | | | | | | | | | | | | | | | | | | | | | | | |

**Appendixes 2**

| Table S2a. Changes in Ranking of Dietary Risk Factors' Attribution Proportion to All Cancer Mortality in China Region by Different Time Periods. | | | | | | | | |
| --- | --- | --- | --- | --- | --- | --- | --- | --- |
|  | 1990-1998 | | 1999-2007 | | 2008-2015 | | 2016-2021 | |
| Dietary Risk Factor | Rank1 | Proportion1(%) | Rank2 | Proportion2(%) | Rank3 | Proportion3(%) | Rank4 | Proportion4(%) |
| Diet High in Red Meat | 3 | 1.613 | 2 | 1.652 | 1 | 1.801 | 1 | 1.955 |
| Diet Low in Whole Grains | 4 | 1.521 | 3 | 1.532 | 2 | 1.649 | 2 | 1.763 |
| Diet Low in Milk | 5 | 1.515 | 4 | 1.496 | 3 | 1.590 | 3 | 1.700 |
| Diet High in Sodium | 2 | 1.954 | 1 | 1.799 | 4 | 1.528 | 4 | 1.340 |
| Diet Low in Calcium | 6 | 1.234 | 6 | 0.990 | 5 | 0.837 | 5 | 0.745 |
| Diet Low in Fruits | 7 | 1.128 | 7 | 0.967 | 6 | 0.781 | 6 | 0.695 |
| Diet Low in Vegetables | 1 | 2.566 | 5 | 1.317 | 7 | 0.540 | 7 | 0.321 |
| Diet High in Processed Meat | 9 | 0.125 | 8 | 0.140 | 8 | 0.191 | 8 | 0.227 |
| Diet Low in Fiber | 8 | 0.130 | 9 | 0.100 | 9 | 0.080 | 9 | 0.066 |
| Note: Rankings are based on attribution proportion of dietary risk factors to all cancer mortality. Rank 1 indicates the highest attribution proportion. | | | | | | | | |

| Table S2b. Average Attribution Proportion (%) of Dietary Risk Factors to Different Cancer Types in China Region (1990-2021) (Mean ± Standard Deviation) | | | | | | | |
| --- | --- | --- | --- | --- | --- | --- | --- |
| Dietary Risk Factor | All Cancers | Colorectal Cancer | Breast Cancer | Stomach Cancer | Prostate Cancer | Tracheal, Bronchus, and Lung Cancer | Esophageal Cancer |
| Diet High in Red Meat | 1.735 ± 0.134 | 15.245 ± 0.365 | 13.580 ± 0.051 |  |  |  |  |
| Diet High in Sodium | 1.689 ± 0.241 |  |  | 8.305 ± 0.006 |  |  |  |
| Diet Low in Whole Grains | 1.601 ± 0.098 | 18.112 ± 0.063 |  |  |  |  |  |
| Diet Low in Milk | 1.563 ± 0.079 | 18.827 ± 0.258 |  |  | -7.980 ± 0.637 |  |  |
| Diet Low in Vegetables | 1.288 ± 0.928 |  |  |  |  |  | 9.810 ± 6.384 |
| Diet Low in Calcium | 0.974 ± 0.195 | 11.434 ± 2.779 |  |  | -1.924 ± 0.363 |  |  |
| Diet Low in Fruits | 0.915 ± 0.173 |  |  |  |  | 3.970 ± 1.312 |  |
| Diet High in Processed Meat | 0.165 ± 0.041 | 1.843 ± 0.346 |  |  |  |  |  |
| Diet Low in Fiber | 0.097 ± 0.025 | 1.118 ± 0.338 |  |  |  |  |  |
| Note: Higher values indicate greater impact of the dietary factor on specific cancer type mortality. | | | | | | | |

| Table S2c. Changes in Ranking of Dietary Risk Factors' Attribution Proportion to All Cancer Mortality in Global Region by Different Time Periods | | | | | | | | |
| --- | --- | --- | --- | --- | --- | --- | --- | --- |
|  | 1990-1998 | | 1999-2007 | | 2008-2015 | | 2016-2021 | |
| Dietary Risk Factor | Rank1 | Proportion1(%) | Rank2 | Proportion2(%) | Rank3 | Proportion3(%) | Rank4 | Proportion4(%) |
| Diet High in Red Meat | 1 | 2.355 | 1 | 2.336 | 1 | 2.352 | 1 | 2.386 |
| Diet Low in Whole Grains | 2 | 1.887 | 2 | 1.887 | 2 | 1.895 | 2 | 1.908 |
| Diet Low in Milk | 3 | 1.410 | 3 | 1.387 | 3 | 1.424 | 3 | 1.481 |
| Diet Low in Calcium | 6 | 0.965 | 5 | 0.921 | 4 | 0.876 | 4 | 0.854 |
| Diet High in Sodium | 5 | 1.105 | 4 | 1.000 | 5 | 0.867 | 5 | 0.781 |
| Diet Low in Fruits | 7 | 0.854 | 7 | 0.775 | 6 | 0.698 | 6 | 0.667 |
| Diet High in Processed Meat | 8 | 0.691 | 8 | 0.668 | 7 | 0.638 | 7 | 0.603 |
| Diet Low in Vegetables | 4 | 1.172 | 6 | 0.852 | 8 | 0.630 | 8 | 0.574 |
| Diet Low in Fiber | 9 | 0.176 | 9 | 0.161 | 9 | 0.147 | 9 | 0.138 |
| Note: Rankings are based on attribution proportion of dietary risk factors to all cancer mortality. Rank 1 indicates the highest attribution proportion. | | | | | | | | |

| Table S2d. Average Attribution Proportion (%) of Dietary Risk Factors to Different Cancer Types in Global Region (1990-2021) (Mean ± Standard Deviation) | | | | | | | |
| --- | --- | --- | --- | --- | --- | --- | --- |
| Dietary Risk Factor | All Cancers | Colorectal Cancer | Breast Cancer | Stomach Cancer | Prostate Cancer | Tracheal, Bronchus, and Lung Cancer | Esophageal Cancer |
| Diet High in Red Meat | 2.355 ± 0.018 | 14.703 ± 0.049 | 12.447 ± 0.213 |  |  |  |  |
| Diet Low in Whole Grains | 1.893 ± 0.008 | 17.911 ± 0.035 |  |  |  |  |  |
| Diet Low in Milk | 1.420 ± 0.035 | 14.348 ± 0.306 |  |  | -2.121 ± 0.202 |  |  |
| Diet High in Sodium | 0.955 ± 0.128 |  |  | 7.929 ± 0.015 |  |  |  |
| Diet Low in Calcium | 0.910 ± 0.045 | 9.121 ± 0.456 |  |  | -1.191 ± 0.019 |  |  |
| Diet Low in Vegetables | 0.834 ± 0.248 |  |  |  |  |  | 14.243 ± 3.622 |
| Diet Low in Fruits | 0.758 ± 0.075 |  |  |  |  | 3.848 ± 0.509 |  |
| Diet High in Processed Meat | 0.655 ± 0.034 | 6.197 ± 0.349 |  |  |  |  |  |
| Diet Low in Fiber | 0.158 ± 0.015 | 1.493 ± 0.149 |  |  |  |  |  |
| Note: Higher values indicate greater impact of the dietary factor on specific cancer type mortality. | | | | | | | |

| Table S2e. Changes in Ranking of Dietary Risk Factors' Attribution Proportion to All Cancer Mortality in High SDI Region by Different Time Periods | | | | | | | | |
| --- | --- | --- | --- | --- | --- | --- | --- | --- |
|  | 1990-1998 | | 1999-2007 | | 2008-2015 | | 2016-2021 | |
| Dietary Risk Factor | Rank1 | Proportion1(%) | Rank2 | Proportion2(%) | Rank3 | Proportion3(%) | Rank4 | Proportion4(%) |
| Diet High in Red Meat | 1 | 3.076 | 1 | 2.963 | 1 | 2.869 | 1 | 2.873 |
| Diet Low in Whole Grains | 2 | 2.269 | 2 | 2.245 | 2 | 2.205 | 2 | 2.212 |
| Diet Low in Milk | 3 | 1.580 | 3 | 1.502 | 3 | 1.477 | 3 | 1.494 |
| Diet High in Processed Meat | 4 | 1.197 | 4 | 1.201 | 4 | 1.168 | 4 | 1.137 |
| Diet Low in Fruits | 5 | 0.708 | 5 | 0.677 | 5 | 0.663 | 5 | 0.615 |
| Diet Low in Calcium | 7 | 0.570 | 7 | 0.540 | 6 | 0.537 | 6 | 0.525 |
| Diet Low in Vegetables | 8 | 0.538 | 8 | 0.532 | 7 | 0.528 | 7 | 0.524 |
| Diet High in Sodium | 6 | 0.674 | 6 | 0.579 | 8 | 0.505 | 8 | 0.452 |
| Diet Low in Fiber | 9 | 0.196 | 9 | 0.172 | 9 | 0.165 | 9 | 0.159 |
| Note: Rankings are based on attribution proportion of dietary risk factors to all cancer mortality. Rank 1 indicates the highest attribution proportion. | | | | | | | | |

| Table S2f. Average Attribution Proportion (%) of Dietary Risk Factors to Different Cancer Types in High SDI Region (1990-2021) (Mean ± Standard Deviation) | | | | | | | |
| --- | --- | --- | --- | --- | --- | --- | --- |
| Dietary Risk Factor | All Cancers | Colorectal Cancer | Breast Cancer | Stomach Cancer | Prostate Cancer | Tracheal, Bronchus, and Lung Cancer | Esophageal Cancer |
| Diet High in Red Meat | 2.955 ± 0.091 | 15.683 ± 0.052 | 13.624 ± 0.012 |  |  |  |  |
| Diet Low in Whole Grains | 2.236 ± 0.028 | 17.883 ± 0.106 |  |  |  |  |  |
| Diet Low in Milk | 1.516 ± 0.044 | 12.190 ± 0.160 |  |  | -0.160 ± 0.036 |  |  |
| Diet High in Processed Meat | 1.180 ± 0.026 | 9.434 ± 0.147 |  |  |  |  |  |
| Diet Low in Fruits | 0.670 ± 0.034 |  |  |  |  | 2.882 ± 0.146 |  |
| Diet High in Sodium | 0.563 ± 0.087 |  |  | 7.893 ± 0.049 |  |  |  |
| Diet Low in Calcium | 0.545 ± 0.019 | 4.495 ± 0.091 |  |  | -0.334 ± 0.011 |  |  |
| Diet Low in Vegetables | 0.531 ± 0.008 |  |  |  |  |  | 16.747 ± 0.864 |
| Diet Low in Fiber | 0.174 ± 0.015 | 1.394 ± 0.098 |  |  |  |  |  |
| Note: Higher values indicate greater impact of the dietary factor on specific cancer type mortality. | | | | | | | |

| Table S2g. Changes in Ranking of Dietary Risk Factors' Attribution Proportion to All Cancer Mortality in High-middle SDI Region by Different Time Periods. | | | | | | | | |
| --- | --- | --- | --- | --- | --- | --- | --- | --- |
|  | 1990-1998 | | 1999-2007 | | 2008-2015 | | 2016-2021 | |
| Dietary Risk Factor | Rank1 | Proportion1(%) | Rank2 | Proportion2(%) | Rank3 | Proportion3(%) | Rank4 | Proportion4(%) |
| Diet High in Red Meat | 1 | 2.429 | 1 | 2.493 | 1 | 2.569 | 1 | 2.611 |
| Diet Low in Whole Grains | 2 | 1.973 | 2 | 2.039 | 2 | 2.116 | 2 | 2.163 |
| Diet Low in Milk | 3 | 1.327 | 3 | 1.350 | 3 | 1.452 | 3 | 1.576 |
| Diet High in Sodium | 4 | 1.298 | 4 | 1.168 | 4 | 0.994 | 4 | 0.900 |
| Diet High in Processed Meat | 8 | 0.712 | 7 | 0.713 | 5 | 0.707 | 5 | 0.685 |
| Diet Low in Calcium | 7 | 0.814 | 5 | 0.763 | 6 | 0.698 | 6 | 0.669 |
| Diet Low in Fruits | 6 | 0.867 | 6 | 0.746 | 7 | 0.589 | 7 | 0.539 |
| Diet Low in Vegetables | 5 | 1.122 | 8 | 0.621 | 8 | 0.328 | 8 | 0.258 |
| Diet Low in Fiber | 9 | 0.126 | 9 | 0.127 | 9 | 0.115 | 9 | 0.106 |
| Note: Rankings are based on attribution proportion of dietary risk factors to all cancer mortality. Rank 1 indicates the highest attribution proportion. | | | | | | | | |

| Table S2h. Average Attribution Proportion (%) of Dietary Risk Factors to Different Cancer Types in High-middle SDI Region (1990-2021) (Mean ± Standard Deviation) | | | | | | | |
| --- | --- | --- | --- | --- | --- | --- | --- |
| Dietary Risk Factor | All Cancers | Colorectal Cancer | Breast Cancer | Stomach Cancer | Prostate Cancer | Tracheal, Bronchus, and Lung Cancer | Esophageal Cancer |
| Diet High in Red Meat | 2.516 ± 0.073 | 15.497 ± 0.058 | 13.515 ± 0.038 |  |  |  |  |
| Diet Low in Whole Grains | 2.063 ± 0.075 | 18.481 ± 0.055 |  |  |  |  |  |
| Diet Low in Milk | 1.412 ± 0.097 | 13.028 ± 0.463 |  |  | -1.321 ± 0.108 |  |  |
| Diet High in Sodium | 1.111 ± 0.159 |  |  | 7.893 ± 0.034 |  |  |  |
| Diet Low in Calcium | 0.743 ± 0.058 | 6.912 ± 0.798 |  |  | -0.750 ± 0.061 |  |  |
| Diet High in Processed Meat | 0.706 ± 0.013 | 6.334 ± 0.327 |  |  |  |  |  |
| Diet Low in Fruits | 0.702 ± 0.135 |  |  |  |  | 3.171 ± 0.783 |  |
| Diet Low in Vegetables | 0.620 ± 0.362 |  |  |  |  |  | 9.580 ± 5.368 |
| Diet Low in Fiber | 0.120 ± 0.009 | 1.076 ± 0.115 |  |  |  |  |  |
| Note: Higher values indicate greater impact of the dietary factor on specific cancer type mortality. | | | | | | | |

| Table S2i. Changes in Ranking of Dietary Risk Factors' Attribution Proportion to All Cancer Mortality in Middle SDI Region by Different Time Periods | | | | | | | | |
| --- | --- | --- | --- | --- | --- | --- | --- | --- |
|  | 1990-1998 | | 1999-2007 | | 2008-2015 | | 2016-2021 | |
| Dietary Risk Factor | Rank1 | Proportion1(%) | Rank2 | Proportion2(%) | Rank3 | Proportion3(%) | Rank4 | Proportion4(%) |
| Diet High in Red Meat | 2 | 1.663 | 1 | 1.762 | 1 | 1.929 | 1 | 2.071 |
| Diet Low in Whole Grains | 5 | 1.421 | 2 | 1.476 | 2 | 1.579 | 2 | 1.663 |
| Diet Low in Milk | 6 | 1.358 | 4 | 1.382 | 3 | 1.459 | 3 | 1.541 |
| Diet Low in Calcium | 4 | 1.434 | 6 | 1.310 | 5 | 1.199 | 4 | 1.128 |
| Diet High in Sodium | 3 | 1.595 | 3 | 1.432 | 4 | 1.200 | 5 | 1.046 |
| Diet Low in Fruits | 7 | 1.021 | 7 | 0.890 | 7 | 0.768 | 6 | 0.724 |
| Diet Low in Vegetables | 1 | 2.058 | 5 | 1.321 | 6 | 0.771 | 7 | 0.611 |
| Diet High in Processed Meat | 9 | 0.135 | 9 | 0.152 | 8 | 0.192 | 8 | 0.216 |
| Diet Low in Fiber | 8 | 0.185 | 8 | 0.167 | 9 | 0.150 | 9 | 0.140 |
| Note: Rankings are based on attribution proportion of dietary risk factors to all cancer mortality. Rank 1 indicates the highest attribution proportion. | | | | | | | | |

| Table S2j. Average Attribution Proportion (%) of Dietary Risk Factors to Different Cancer Types in Middle SDI Region (1990-2021) (Mean ± Standard Deviation). | | | | | | | |
| --- | --- | --- | --- | --- | --- | --- | --- |
| Dietary Risk Factor | All Cancers | Colorectal Cancer | Breast Cancer | Stomach Cancer | Prostate Cancer | Tracheal, Bronchus, and Lung Cancer | Esophageal Cancer |
| Diet High in Red Meat | 1.834 ± 0.157 | 13.974 ± 0.232 | 11.632 ± 0.078 |  |  |  |  |
| Diet Low in Whole Grains | 1.521 ± 0.093 | 17.441 ± 0.062 |  |  |  |  |  |
| Diet Low in Milk | 1.424 ± 0.071 | 18.214 ± 0.352 |  |  | -4.250 ± 0.161 |  |  |
| Diet High in Sodium | 1.347 ± 0.216 |  |  | 8.120 ± 0.034 |  |  |  |
| Diet Low in Calcium | 1.283 ± 0.121 | 15.792 ± 2.410 |  |  | -2.136 ± 0.224 |  |  |
| Diet Low in Vegetables | 1.257 ± 0.594 |  |  |  |  |  | 13.553 ± 4.847 |
| Diet Low in Fruits | 0.865 ± 0.120 |  |  |  |  | 4.657 ± 1.054 |  |
| Diet High in Processed Meat | 0.169 ± 0.032 | 1.922 ± 0.243 |  |  |  |  |  |
| Diet Low in Fiber | 0.163 ± 0.018 | 1.883 ± 0.320 |  |  |  |  |  |
| Note: Higher values indicate greater impact of the dietary factor on specific cancer type mortality. | | | | | | | |

| Table S2k. Changes in Ranking of Dietary Risk Factors' Attribution Proportion to All Cancer Mortality in Low-middle SDI Region by Different Time Periods | | | | | | | | |
| --- | --- | --- | --- | --- | --- | --- | --- | --- |
|  | 1990-1998 | | 1999-2007 | | 2008-2015 | | 2016-2021 | |
| Dietary Risk Factor | Rank1 | Proportion1(%) | Rank2 | Proportion2(%) | Rank3 | Proportion3(%) | Rank4 | Proportion4(%) |
| Diet High in Red Meat | 2 | 1.266 | 1 | 1.410 | 1 | 1.548 | 1 | 1.647 |
| Diet Low in Whole Grains | 4 | 1.200 | 3 | 1.250 | 2 | 1.281 | 2 | 1.306 |
| Diet Low in Calcium | 1 | 1.366 | 2 | 1.332 | 3 | 1.222 | 3 | 1.160 |
| Diet Low in Milk | 6 | 1.026 | 5 | 1.061 | 4 | 1.095 | 4 | 1.132 |
| Diet Low in Vegetables | 3 | 1.232 | 4 | 1.169 | 5 | 1.076 | 5 | 1.021 |
| Diet Low in Fruits | 5 | 1.110 | 6 | 1.015 | 6 | 0.974 | 6 | 0.987 |
| Diet High in Sodium | 7 | 0.947 | 7 | 0.870 | 7 | 0.807 | 7 | 0.724 |
| Diet High in Processed Meat | 9 | 0.152 | 9 | 0.162 | 8 | 0.176 | 8 | 0.184 |
| Diet Low in Fiber | 8 | 0.211 | 8 | 0.198 | 9 | 0.165 | 9 | 0.154 |
| Note: Rankings are based on attribution proportion of dietary risk factors to all cancer mortality. Rank 1 indicates the highest attribution proportion. | | | | | | | | |

| Table S2l. Average Attribution Proportion (%) of Dietary Risk Factors to Different Cancer Types in Low-middle SDI Region (1990-2021) (Mean ± Standard Deviation) | | | | | | | |
| --- | --- | --- | --- | --- | --- | --- | --- |
| Dietary Risk Factor | All Cancers | Colorectal Cancer | Breast Cancer | Stomach Cancer | Prostate Cancer | Tracheal, Bronchus, and Lung Cancer | Esophageal Cancer |
| Diet High in Red Meat | 1.448 ± 0.147 | 9.852 ± 0.359 | 9.473 ± 0.165 |  |  |  |  |
| Diet Low in Calcium | 1.282 ± 0.084 | 19.581 ± 1.928 |  |  | -2.657 ± 0.235 |  |  |
| Diet Low in Whole Grains | 1.254 ± 0.041 | 17.035 ± 0.048 |  |  |  |  |  |
| Diet Low in Vegetables | 1.136 ± 0.083 |  |  |  |  |  | 23.064 ± 0.322 |
| Diet Low in Milk | 1.073 ± 0.040 | 18.538 ± 0.185 |  |  | -4.959 ± 0.229 |  |  |
| Diet Low in Fruits | 1.026 ± 0.058 |  |  |  |  | 9.506 ± 0.791 |  |
| Diet High in Sodium | 0.849 ± 0.083 |  |  | 7.738 ± 0.025 |  |  |  |
| Diet Low in Fiber | 0.185 ± 0.024 | 2.530 ± 0.410 |  |  |  |  |  |
| Diet High in Processed Meat | 0.167 ± 0.012 | 2.260 ± 0.090 |  |  |  |  |  |
| Note: Higher values indicate greater impact of the dietary factor on specific cancer type mortality. | | | | | | | |

| Table S2m. Changes in Ranking of Dietary Risk Factors' Attribution Proportion to All Cancer Mortality in Low SDI Region by Different Time Periods. | | | | | | | | |
| --- | --- | --- | --- | --- | --- | --- | --- | --- |
|  | 1990-1998 | | 1999-2007 | | 2008-2015 | | 2016-2021 | |
| Dietary Risk Factor | Rank1 | Proportion1(%) | Rank2 | Proportion2(%) | Rank3 | Proportion3(%) | Rank4 | Proportion4(%) |
| Diet High in Red Meat | 3 | 1.431 | 3 | 1.488 | 2 | 1.593 | 1 | 1.716 |
| Diet Low in Vegetables | 1 | 1.851 | 1 | 1.811 | 1 | 1.695 | 2 | 1.639 |
| Diet Low in Calcium | 2 | 1.721 | 2 | 1.653 | 3 | 1.528 | 3 | 1.489 |
| Diet Low in Whole Grains | 4 | 1.301 | 4 | 1.302 | 4 | 1.313 | 4 | 1.346 |
| Diet Low in Milk | 5 | 0.845 | 5 | 0.815 | 5 | 0.810 | 5 | 0.839 |
| Diet High in Sodium | 6 | 0.844 | 6 | 0.792 | 6 | 0.755 | 6 | 0.689 |
| Diet Low in Fruits | 7 | 0.626 | 7 | 0.622 | 7 | 0.621 | 7 | 0.632 |
| Diet High in Processed Meat | 8 | 0.206 | 8 | 0.210 | 8 | 0.219 | 8 | 0.227 |
| Diet Low in Fiber | 9 | 0.080 | 9 | 0.088 | 9 | 0.079 | 9 | 0.072 |
| Note: Rankings are based on attribution proportion of dietary risk factors to all cancer mortality. Rank 1 indicates the highest attribution proportion. | | | | | | | | |

| Table S2n. Average Attribution Proportion (%) of Dietary Risk Factors to Different Cancer Types in Low SDI Region (1990-2021) (Mean ± Standard Deviation) | | | | | | | |
| --- | --- | --- | --- | --- | --- | --- | --- |
| Dietary Risk Factor | All Cancers | Colorectal Cancer | Breast Cancer | Stomach Cancer | Prostate Cancer | Tracheal, Bronchus, and Lung Cancer | Esophageal Cancer |
| Diet Low in Vegetables | 1.761 ± 0.087 |  |  |  |  |  | 25.463 ± 0.272 |
| Diet Low in Calcium | 1.610 ± 0.096 | 25.652 ± 1.604 |  |  | -3.341 ± 0.134 |  |  |
| Diet High in Red Meat | 1.541 ± 0.108 | 10.261 ± 0.114 | 10.066 ± 0.049 |  |  |  |  |
| Diet Low in Whole Grains | 1.313 ± 0.018 | 17.890 ± 0.042 |  |  |  |  |  |
| Diet Low in Milk | 0.827 ± 0.018 | 18.743 ± 0.068 |  |  | -6.744 ± 0.134 |  |  |
| Diet High in Sodium | 0.778 ± 0.057 |  |  | 7.153 ± 0.024 |  |  |  |
| Diet Low in Fruits | 0.625 ± 0.005 |  |  |  |  | 10.103 ± 0.232 |  |
| Diet High in Processed Meat | 0.214 ± 0.008 | 2.918 ± 0.072 |  |  |  |  |  |
| Diet Low in Fiber | 0.080 ± 0.006 | 1.098 ± 0.097 |  |  |  |  |  |
| Note: Higher values indicate greater impact of the dietary factor on specific cancer type mortality. | | | | | | | |

**Appendixes 3**

| Table S3a. Key Year Values and Annual Percent Change of Dietary Risk Factors' Impact on All Cancer Mortality in China Region | | | | | |
| --- | --- | --- | --- | --- | --- |
| Dietary Risk Factor | 1990 | 2000 | 2010 | 2021 | AAPC |
| Diet Low in Vegetables | 5.496 | 2.969 | 0.958 | 0.434 | -14.86% (-15.60%, -14.12%) |
| Diet High in Sodium | 3.848 | 3.206 | 2.475 | 1.783 | -5.85% (-6.86%, -4.83%) |
| Diet High in Red Meat | 2.928 | 2.874 | 2.779 | 2.751 | -0.16% (-0.70%, 0.39%) |
| Diet Low in Milk | 2.815 | 2.619 | 2.469 | 2.388 | -1.10% (-1.51%, -0.69%) |
| Diet Low in Whole Grains | 2.790 | 2.677 | 2.547 | 2.470 | -1.00% (-3.25%, 1.31%) |
| Diet Low in Calcium | 2.503 | 1.830 | 1.359 | 1.012 | -4.57% (-5.15%, -3.98%) |
| Diet Low in Fruits | 2.181 | 1.829 | 1.260 | 0.935 | -3.93% (-4.37%, -3.49%) |
| All Dietary Risks | 18.441 | 14.282 | 10.511 | 8.662 | -24.89% (-28.69%, -20.89%) |
| Diet Low in Fiber | 0.271 | 0.189 | 0.132 | 0.088 | -0.61% (-0.65%, -0.57%) |
| Diet High in Processed Meat | 0.227 | 0.228 | 0.285 | 0.321 | 0.30% (0.17%, 0.44%) |
| Note: Mortality rate is per 100,000 population; AAPC = Average Annual Percent Change (95% Confidence Interval); Negative values indicate a decrease in mortality rate. | | | | | |

| Table S3b. Key Year Values and Annual Percent Change of Dietary Risk Factors' Impact on All Cancer Mortality in Global Region | | | | | |
| --- | --- | --- | --- | --- | --- |
| Dietary Risk Factor | 1990 | 2000 | 2010 | 2021 | AAPC |
| Diet High in Red Meat | 3.477 | 3.277 | 2.973 | 2.773 | -2.23% (-2.98%, -1.49%) |
| Diet Low in Whole Grains | 2.787 | 2.641 | 2.399 | 2.212 | -1.81% (-2.40%, -1.21%) |
| Diet Low in Milk | 2.106 | 1.942 | 1.791 | 1.740 | -1.09% (-1.49%, -0.70%) |
| All Dietary Risks | 12.237 | 10.711 | 8.968 | 7.899 | -12.94% (-15.30%, -10.52%) |
| Diet Low in Vegetables | 1.888 | 1.333 | 0.828 | 0.665 | -3.89% (-4.07%, -3.71%) |
| Diet High in Sodium | 1.744 | 1.435 | 1.136 | 0.887 | -2.63% (-2.92%, -2.34%) |
| Diet Low in Calcium | 1.464 | 1.300 | 1.122 | 0.993 | -1.46% (-1.67%, -1.24%) |
| Diet Low in Fruits | 1.300 | 1.132 | 0.895 | 0.769 | -1.62% (-1.88%, -1.35%) |
| Diet High in Processed Meat | 1.031 | 0.947 | 0.816 | 0.681 | -1.11% (-1.26%, -0.95%) |
| Diet Low in Fiber | 0.273 | 0.232 | 0.190 | 0.158 | -0.36% (-0.40%, -0.33%) |
| Note: Mortality rate is per 100,000 population; AAPC = Average Annual Percent Change (95% Confidence Interval); Negative values indicate a decrease in mortality rate. | | | | | |

| Table S3c. Key Year Values and Annual Percent Change of Dietary Risk Factors' Impact on All Cancer Mortality in High SDI Region. | | | | | |
| --- | --- | --- | --- | --- | --- |
| Dietary Risk Factor | 1990 | 2000 | 2010 | 2021 | AAPC |
| Diet High in Red Meat | 5.293 | 4.720 | 3.996 | 3.497 | -5.71% (-6.32%, -5.09%) |
| Diet Low in Whole Grains | 3.868 | 3.549 | 3.065 | 2.698 | -3.71% (-4.33%, -3.08%) |
| Diet Low in Milk | 2.711 | 2.398 | 2.047 | 1.829 | -2.85% (-3.13%, -2.57%) |
| Diet High in Processed Meat | 2.029 | 1.890 | 1.633 | 1.369 | -2.12% (-2.47%, -1.77%) |
| All Dietary Risks | 13.809 | 12.226 | 10.417 | 8.892 | -14.63% (-17.12%, -12.07%) |
| Diet Low in Fruits | 1.249 | 1.068 | 0.927 | 0.735 | -1.63% (-1.78%, -1.47%) |
| Diet High in Sodium | 1.238 | 0.956 | 0.725 | 0.543 | -2.20% (-2.36%, -2.04%) |
| Diet Low in Calcium | 1.000 | 0.854 | 0.747 | 0.634 | -1.18% (-1.28%, -1.07%) |
| Diet Low in Vegetables | 0.936 | 0.829 | 0.746 | 0.636 | -0.95% (-1.20%, -0.69%) |
| Diet Low in Fiber | 0.356 | 0.277 | 0.231 | 0.192 | -0.54% (-0.57%, -0.51%) |
| Note: Mortality rate is per 100,000 population; AAPC = Average Annual Percent Change (95% Confidence Interval); Negative values indicate a decrease in mortality rate. | | | | | |

| Table S3d. Key Year Values and Annual Percent Change of Dietary Risk Factors' Impact on All Cancer Mortality in High-middle SDI Region. | | | | | |
| --- | --- | --- | --- | --- | --- |
| Dietary Risk Factor | 1990 | 2000 | 2010 | 2021 | AAPC |
| Diet High in Red Meat | 4.117 | 4.136 | 3.832 | 3.484 | -1.91% (-3.16%, -0.65%) |
| Diet Low in Whole Grains | 3.360 | 3.371 | 3.150 | 2.887 | -1.45% (-2.80%, -0.09%) |
| Diet High in Sodium | 2.442 | 1.998 | 1.533 | 1.184 | -3.66% (-4.23%, -3.08%) |
| Diet Low in Milk | 2.332 | 2.231 | 2.136 | 2.157 | -0.47% (-1.12%, 0.18%) |
| Diet Low in Vegetables | 2.263 | 1.283 | 0.527 | 0.339 | -5.88% (-6.13%, -5.63%) |
| All Dietary Risks | 14.789 | 13.071 | 10.685 | 9.163 | -16.46% (-19.89%, -12.88%) |
| Diet Low in Fruits | 1.547 | 1.357 | 0.907 | 0.712 | -2.57% (-3.05%, -2.10%) |
| Diet Low in Calcium | 1.460 | 1.303 | 1.061 | 0.893 | -1.77% (-2.21%, -1.32%) |
| Diet High in Processed Meat | 1.213 | 1.191 | 1.064 | 0.892 | -1.05% (-1.48%, -0.63%) |
| Diet Low in Fiber | 0.217 | 0.217 | 0.174 | 0.138 | -0.26% (-0.31%, -0.20%) |
| Note: Mortality rate is per 100,000 population; AAPC = Average Annual Percent Change (95% Confidence Interval); Negative values indicate a decrease in mortality rate. | | | | | |

| Table S3e. Key Year Values and Annual Percent Change of Dietary Risk Factors' Impact on All Cancer Mortality in Middle SDI Region. | | | | | |
| --- | --- | --- | --- | --- | --- |
| Dietary Risk Factor | 1990 | 2000 | 2010 | 2021 | AAPC |
| Diet Low in Vegetables | 3.107 | 2.012 | 0.977 | 0.665 | -7.59% (-8.30%, -6.87%) |
| Diet High in Sodium | 2.301 | 1.890 | 1.474 | 1.107 | -3.60% (-4.24%, -2.96%) |
| Diet High in Red Meat | 2.188 | 2.219 | 2.246 | 2.311 | 0.53% (0.19%, 0.87%) |
| Diet Low in Calcium | 2.027 | 1.717 | 1.448 | 1.233 | -2.42% (-2.80%, -2.04%) |
| All Dietary Risks | 11.925 | 9.981 | 8.101 | 7.223 | -13.01% (-15.34%, -10.62%) |
| Diet Low in Whole Grains | 1.894 | 1.870 | 1.848 | 1.848 | 0.04% (-1.07%, 1.16%) |
| Diet Low in Milk | 1.830 | 1.759 | 1.714 | 1.717 | -0.33% (-0.71%, 0.05%) |
| Diet Low in Fruits | 1.438 | 1.215 | 0.926 | 0.788 | -1.99% (-2.35%, -1.63%) |
| Diet Low in Fiber | 0.264 | 0.219 | 0.181 | 0.153 | -0.37% (-0.40%, -0.33%) |
| Diet High in Processed Meat | 0.179 | 0.183 | 0.218 | 0.241 | 0.20% (0.17%, 0.24%) |
| Note: Mortality rate is per 100,000 population; AAPC = Average Annual Percent Change (95% Confidence Interval); Negative values indicate a decrease in mortality rate. | | | | | |

| Table S3f. Key Year Values and Annual Percent Change of Dietary Risk Factors' Impact on All Cancer Mortality in Low-middle SDI Region. | | | | | |
| --- | --- | --- | --- | --- | --- |
| Dietary Risk Factor | 1990 | 2000 | 2010 | 2021 | AAPC |
| All Dietary Risks | 5.304 | 5.276 | 5.238 | 5.232 | -0.15% (-0.98%, 0.69%) |
| Diet Low in Calcium | 1.149 | 1.123 | 1.041 | 0.982 | -0.56% (-0.80%, -0.31%) |
| Diet Low in Vegetables | 1.025 | 1.000 | 0.923 | 0.860 | -0.49% (-0.67%, -0.32%) |
| Diet High in Red Meat | 1.000 | 1.132 | 1.280 | 1.422 | 1.40% (1.11%, 1.69%) |
| Diet Low in Whole Grains | 0.979 | 1.027 | 1.069 | 1.111 | 0.40% (-0.09%, 0.89%) |
| Diet Low in Fruits | 0.943 | 0.873 | 0.819 | 0.841 | -0.31% (-0.42%, -0.20%) |
| Diet Low in Milk | 0.844 | 0.867 | 0.910 | 0.966 | 0.41% (0.27%, 0.55%) |
| Diet High in Sodium | 0.816 | 0.747 | 0.698 | 0.592 | -0.74% (-1.14%, -0.34%) |
| Diet Low in Fiber | 0.178 | 0.172 | 0.143 | 0.131 | -0.14% (-0.15%, -0.12%) |
| Diet High in Processed Meat | 0.124 | 0.132 | 0.146 | 0.158 | 0.12% (0.11%, 0.13%) |
| Note: Mortality rate is per 100,000 population; AAPC = Average Annual Percent Change (95% Confidence Interval); Negative values indicate a decrease in mortality rate. | | | | | |

| Table S3g. Key Year Values and Annual Percent Change of Dietary Risk Factors' Impact on All Cancer Mortality in Low SDI Region. | | | | | |
| --- | --- | --- | --- | --- | --- |
| Dietary Risk Factor | 1990 | 2000 | 2010 | 2021 | AAPC |
| All Dietary Risks | 6.614 | 6.272 | 5.713 | 5.808 | -2.32% (-3.01%, -1.62%) |
| Diet Low in Vegetables | 1.839 | 1.767 | 1.536 | 1.483 | -1.12% (-1.25%, -0.99%) |
| Diet Low in Calcium | 1.706 | 1.606 | 1.373 | 1.343 | -1.10% (-1.41%, -0.79%) |
| Diet High in Red Meat | 1.417 | 1.393 | 1.399 | 1.593 | 0.56% (0.46%, 0.66%) |
| Diet Low in Whole Grains | 1.291 | 1.238 | 1.167 | 1.226 | -0.20% (-0.37%, -0.03%) |
| Diet High in Sodium | 0.858 | 0.770 | 0.686 | 0.599 | -0.80% (-1.01%, -0.58%) |
| Diet Low in Milk | 0.851 | 0.774 | 0.712 | 0.770 | -0.28% (-0.45%, -0.11%) |
| Diet Low in Fruits | 0.623 | 0.599 | 0.555 | 0.573 | -0.15% (-0.36%, 0.06%) |
| Diet High in Processed Meat | 0.205 | 0.197 | 0.194 | 0.208 | 0.01% (-0.01%, 0.04%) |
| Diet Low in Fiber | 0.074 | 0.083 | 0.072 | 0.064 | -0.04% (-0.06%, -0.01%) |
| Note: Mortality rate is per 100,000 population; AAPC = Average Annual Percent Change (95% Confidence Interval); Negative values indicate a decrease in mortality rate. | | | | | |

**Appendixes 4**

| Table S4a. Gender Differences in Dietary Risk Factors' Attribution to Cancer Mortality (%) in China Region, 1990-1998 (Mean ± Standard Error). | | | | |
| --- | --- | --- | --- | --- |
| Dietary Risk Factor | Both Sexes | Male | Female | Male/Female Ratio |
| Diet Low in Vegetables | 2.566 ± 0.112 | 2.891 ± 0.121 | 1.990 ± 0.092 | 1.45 |
| Diet High in Sodium | 1.954 ± 0.026 | 2.101 ± 0.028 | 1.681 ± 0.025 | 1.25 |
| Diet High in Red Meat | 1.613 ± 0.007 | 1.163 ± 0.005 | 2.363 ± 0.014 | 0.49 |
| Diet Low in Whole Grains | 1.521 ± 0.003 | 1.410 ± 0.005 | 1.722 ± 0.002 | 0.82 |
| Diet Low in Milk | 1.515 ± 0.003 | 1.022 ± 0.001 | 2.229 ± 0.003 | 0.46 |
| Diet Low in Calcium | 1.234 ± 0.032 | 1.012 ± 0.030 | 1.596 ± 0.034 | 0.63 |
| Diet Low in Fruits | 1.128 ± 0.012 | 1.234 ± 0.017 | 0.972 ± 0.006 | 1.27 |
| Diet Low in Fiber | 0.130 ± 0.004 | 0.111 ± 0.003 | 0.161 ± 0.005 | 0.69 |
| Diet High in Processed Meat | 0.125 ± 0.001 | 0.110 ± 0.001 | 0.148 ± 0.001 | 0.74 |
| Note: Male/Female Ratio greater than 1 indicates that the dietary risk factor has a greater impact on males than females. | | | | |

| Table S4b. Gender Differences in Dietary Risk Factors' Attribution to Cancer Mortality (%) in China Region, 1999-2007 (Mean ± Standard Error). | | | | |
| --- | --- | --- | --- | --- |
| Dietary Risk Factor | Both Sexes | Male | Female | Male/Female Ratio |
| Diet High in Sodium | 1.799 ± 0.018 | 1.931 ± 0.017 | 1.534 ± 0.020 | 1.26 |
| Diet High in Red Meat | 1.652 ± 0.011 | 1.217 ± 0.012 | 2.437 ± 0.014 | 0.50 |
| Diet Low in Whole Grains | 1.532 ± 0.007 | 1.453 ± 0.011 | 1.694 ± 0.004 | 0.86 |
| Diet Low in Milk | 1.496 ± 0.006 | 1.059 ± 0.012 | 2.181 ± 0.005 | 0.49 |
| Diet Low in Vegetables | 1.317 ± 0.111 | 1.539 ± 0.120 | 0.971 ± 0.091 | 1.58 |
| Diet Low in Calcium | 0.990 ± 0.015 | 0.791 ± 0.013 | 1.339 ± 0.016 | 0.59 |
| Diet Low in Fruits | 0.967 ± 0.022 | 1.046 ± 0.023 | 0.848 ± 0.019 | 1.23 |
| Diet High in Processed Meat | 0.140 ± 0.004 | 0.125 ± 0.004 | 0.165 ± 0.004 | 0.76 |
| Diet Low in Fiber | 0.100 ± 0.002 | 0.088 ± 0.001 | 0.123 ± 0.003 | 0.71 |
| Note: Male/Female Ratio greater than 1 indicates that the dietary risk factor has a greater impact on males than females. | | | | |

| Table S4c. Gender Differences in Dietary Risk Factors' Attribution to Cancer Mortality (%) in China Region, 2008-2015 (Mean ± Standard Error). | | | | |
| --- | --- | --- | --- | --- |
| Dietary Risk Factor | Both Sexes | Male | Female | Male/Female Ratio |
| Diet High in Red Meat | 1.801 ± 0.016 | 1.393 ± 0.020 | 2.604 ± 0.014 | 0.53 |
| Diet Low in Whole Grains | 1.649 ± 0.013 | 1.611 ± 0.018 | 1.742 ± 0.004 | 0.92 |
| Diet Low in Milk | 1.590 ± 0.010 | 1.215 ± 0.016 | 2.243 ± 0.006 | 0.54 |
| Diet High in Sodium | 1.528 ± 0.029 | 1.656 ± 0.031 | 1.261 ± 0.027 | 1.31 |
| Diet Low in Calcium | 0.837 ± 0.018 | 0.642 ± 0.018 | 1.211 ± 0.013 | 0.53 |
| Diet Low in Fruits | 0.781 ± 0.015 | 0.851 ± 0.015 | 0.668 ± 0.016 | 1.27 |
| Diet Low in Vegetables | 0.540 ± 0.047 | 0.669 ± 0.057 | 0.355 ± 0.035 | 1.89 |
| Diet High in Processed Meat | 0.191 ± 0.006 | 0.174 ± 0.006 | 0.219 ± 0.006 | 0.79 |
| Diet Low in Fiber | 0.080 ± 0.002 | 0.072 ± 0.002 | 0.095 ± 0.003 | 0.76 |
| Note: Male/Female Ratio greater than 1 indicates that the dietary risk factor has a greater impact on males than females. | | | | |

| Table S4d. Gender Differences in Dietary Risk Factors' Attribution to Cancer Mortality (%) in China Region, 2016-2021 (Mean ± Standard Error). | | | | |
| --- | --- | --- | --- | --- |
| Dietary Risk Factor | Both Sexes | Male | Female | Male/Female Ratio |
| Diet High in Red Meat | 1.955 ± 0.019 | 1.547 ± 0.016 | 2.727 ± 0.017 | 0.57 |
| Diet Low in Whole Grains | 1.763 ± 0.012 | 1.760 ± 0.016 | 1.787 ± 0.005 | 0.99 |
| Diet Low in Milk | 1.700 ± 0.015 | 1.338 ± 0.016 | 2.307 ± 0.007 | 0.58 |
| Diet High in Sodium | 1.340 ± 0.015 | 1.464 ± 0.015 | 1.096 ± 0.013 | 1.34 |
| Diet Low in Calcium | 0.745 ± 0.004 | 0.529 ± 0.007 | 1.136 ± 0.005 | 0.47 |
| Diet Low in Fruits | 0.695 ± 0.006 | 0.755 ± 0.008 | 0.598 ± 0.001 | 1.26 |
| Diet Low in Vegetables | 0.321 ± 0.008 | 0.411 ± 0.009 | 0.196 ± 0.006 | 2.10 |
| Diet High in Processed Meat | 0.227 ± 0.003 | 0.210 ± 0.003 | 0.252 ± 0.002 | 0.83 |
| Diet Low in Fiber | 0.066 ± 0.001 | 0.061 ± 0.001 | 0.075 ± 0.002 | 0.82 |
| Note: Male/Female Ratio greater than 1 indicates that the dietary risk factor has a greater impact on males than females. | | | | |

| Table S4e. Gender Differences in Dietary Risk Factors' Attribution to Cancer Mortality (%) in Global Region, 1990-1998 (Mean ± Standard Error). | | | | |
| --- | --- | --- | --- | --- |
| Dietary Risk Factor | Both Sexes | Male | Female | Male/Female Ratio |
| Diet High in Red Meat | 2.355 ± 0.002 | 1.429 ± 0.002 | 3.542 ± 0.005 | 0.40 |
| Diet Low in Whole Grains | 1.887 ± 0.001 | 1.719 ± 0.004 | 2.081 ± 0.006 | 0.83 |
| Diet Low in Milk | 1.410 ± 0.003 | 0.826 ± 0.001 | 2.091 ± 0.006 | 0.39 |
| Diet Low in Vegetables | 1.172 ± 0.028 | 1.384 ± 0.032 | 0.848 ± 0.022 | 1.63 |
| Diet High in Sodium | 1.105 ± 0.016 | 1.234 ± 0.016 | 0.909 ± 0.016 | 1.36 |
| Diet Low in Calcium | 0.965 ± 0.007 | 0.612 ± 0.005 | 1.374 ± 0.007 | 0.45 |
| Diet Low in Fruits | 0.854 ± 0.007 | 1.065 ± 0.011 | 0.553 ± 0.001 | 1.93 |
| Diet High in Processed Meat | 0.691 ± 0.002 | 0.596 ± 0.001 | 0.800 ± 0.004 | 0.75 |
| Diet Low in Fiber | 0.176 ± 0.002 | 0.151 ± 0.001 | 0.205 ± 0.002 | 0.74 |
| Note: Male/Female Ratio greater than 1 indicates that the dietary risk factor has a greater impact on males than females. | | | | |

| Table S4f. Gender Differences in Dietary Risk Factors' Attribution to Cancer Mortality (%) in Global Region, 1999-2007 (Mean ± Standard Error). | | | | |
| --- | --- | --- | --- | --- |
| Dietary Risk Factor | Both Sexes | Male | Female | Male/Female Ratio |
| Diet High in Red Meat | 2.336 ± 0.002 | 1.461 ± 0.005 | 3.484 ± 0.005 | 0.42 |
| Diet Low in Whole Grains | 1.887 ± 0.001 | 1.764 ± 0.006 | 2.022 ± 0.007 | 0.87 |
| Diet Low in Milk | 1.387 ± 0.001 | 0.844 ± 0.005 | 2.035 ± 0.004 | 0.41 |
| Diet High in Sodium | 1.000 ± 0.011 | 1.135 ± 0.010 | 0.797 ± 0.011 | 1.43 |
| Diet Low in Calcium | 0.921 ± 0.003 | 0.589 ± 0.001 | 1.316 ± 0.005 | 0.45 |
| Diet Low in Vegetables | 0.852 ± 0.030 | 1.023 ± 0.035 | 0.606 ± 0.022 | 1.69 |
| Diet Low in Fruits | 0.775 ± 0.010 | 0.945 ± 0.014 | 0.530 ± 0.004 | 1.78 |
| Diet High in Processed Meat | 0.668 ± 0.002 | 0.590 ± 0.001 | 0.757 ± 0.005 | 0.78 |
| Diet Low in Fiber | 0.161 ± 0.001 | 0.141 ± 0.001 | 0.184 ± 0.002 | 0.77 |
| Note: Male/Female Ratio greater than 1 indicates that the dietary risk factor has a greater impact on males than females. | | | | |

| Table S4g. Gender Differences in Dietary Risk Factors' Attribution to Cancer Mortality (%) in Global Region, 2008-2015 (Mean ± Standard Error). | | | | |
| --- | --- | --- | --- | --- |
| Dietary Risk Factor | Both Sexes | Male | Female | Male/Female Ratio |
| Diet High in Red Meat | 2.352 ± 0.003 | 1.524 ± 0.008 | 3.456 ± 0.004 | 0.44 |
| Diet Low in Whole Grains | 1.895 ± 0.002 | 1.829 ± 0.008 | 1.957 ± 0.007 | 0.93 |
| Diet Low in Milk | 1.424 ± 0.006 | 0.927 ± 0.011 | 2.026 ± 0.002 | 0.46 |
| Diet Low in Calcium | 0.876 ± 0.005 | 0.565 ± 0.004 | 1.254 ± 0.007 | 0.45 |
| Diet High in Sodium | 0.867 ± 0.014 | 1.003 ± 0.014 | 0.666 ± 0.012 | 1.51 |
| Diet Low in Fruits | 0.698 ± 0.006 | 0.835 ± 0.008 | 0.499 ± 0.002 | 1.67 |
| Diet High in Processed Meat | 0.638 ± 0.004 | 0.582 ± 0.002 | 0.701 ± 0.007 | 0.83 |
| Diet Low in Vegetables | 0.630 ± 0.014 | 0.757 ± 0.017 | 0.450 ± 0.009 | 1.68 |
| Diet Low in Fiber | 0.147 ± 0.001 | 0.133 ± 0.001 | 0.164 ± 0.002 | 0.81 |
| Note: Male/Female Ratio greater than 1 indicates that the dietary risk factor has a greater impact on males than females. | | | | |

| Table S4h. Gender Differences in Dietary Risk Factors' Attribution to Cancer Mortality (%) in Global Region, 2016-2021 (Mean ± Standard Error). | | | | |
| --- | --- | --- | --- | --- |
| Dietary Risk Factor | Both Sexes | Male | Female | Male/Female Ratio |
| Diet High in Red Meat | 2.386 ± 0.003 | 1.581 ± 0.004 | 3.437 ± 0.001 | 0.46 |
| Diet Low in Whole Grains | 1.908 ± 0.001 | 1.890 ± 0.005 | 1.906 ± 0.005 | 0.99 |
| Diet Low in Milk | 1.481 ± 0.006 | 1.006 ± 0.008 | 2.045 ± 0.002 | 0.49 |
| Diet Low in Calcium | 0.854 ± 0.001 | 0.551 ± 0.002 | 1.212 ± 0.002 | 0.45 |
| Diet High in Sodium | 0.781 ± 0.006 | 0.909 ± 0.006 | 0.597 ± 0.004 | 1.52 |
| Diet Low in Fruits | 0.667 ± 0.001 | 0.784 ± 0.003 | 0.500 ± 0.001 | 1.57 |
| Diet High in Processed Meat | 0.603 ± 0.005 | 0.566 ± 0.003 | 0.640 ± 0.007 | 0.88 |
| Diet Low in Vegetables | 0.574 ± 0.002 | 0.690 ± 0.001 | 0.413 ± 0.002 | 1.67 |
| Diet Low in Fiber | 0.138 ± 0.001 | 0.128 ± 0.000 | 0.148 ± 0.001 | 0.86 |
| Note: Male/Female Ratio greater than 1 indicates that the dietary risk factor has a greater impact on males than females. | | | | |

| Table S4i. Gender Differences in Dietary Risk Factors' Attribution to Cancer Mortality (%) in High SDI Region, 1990-1998 (Mean ± Standard Error). | | | | |
| --- | --- | --- | --- | --- |
| Dietary Risk Factor | Both Sexes | Male | Female | Male/Female Ratio |
| Diet High in Red Meat | 3.076 ± 0.012 | 1.872 ± 0.001 | 4.590 ± 0.028 | 0.41 |
| Diet Low in Whole Grains | 2.269 ± 0.003 | 2.105 ± 0.003 | 2.432 ± 0.011 | 0.87 |
| Diet Low in Milk | 1.580 ± 0.006 | 0.973 ± 0.001 | 2.258 ± 0.014 | 0.43 |
| Diet High in Processed Meat | 1.197 ± 0.001 | 1.076 ± 0.002 | 1.320 ± 0.004 | 0.82 |
| Diet Low in Fruits | 0.708 ± 0.006 | 0.858 ± 0.011 | 0.501 ± 0.001 | 1.71 |
| Diet High in Sodium | 0.674 ± 0.011 | 0.754 ± 0.011 | 0.561 ± 0.012 | 1.35 |
| Diet Low in Calcium | 0.570 ± 0.005 | 0.201 ± 0.002 | 0.980 ± 0.008 | 0.21 |
| Diet Low in Vegetables | 0.538 ± 0.003 | 0.704 ± 0.003 | 0.293 ± 0.002 | 2.40 |
| Diet Low in Fiber | 0.196 ± 0.003 | 0.169 ± 0.003 | 0.224 ± 0.004 | 0.76 |
| Note: Male/Female Ratio greater than 1 indicates that the dietary risk factor has a greater impact on males than females. | | | | |

| Table S4j. Gender Differences in Dietary Risk Factors' Attribution to Cancer Mortality (%) in High SDI Region, 1999-2007 (Mean ± Standard Error). | | | | |
| --- | --- | --- | --- | --- |
| Dietary Risk Factor | Both Sexes | Male | Female | Male/Female Ratio |
| Diet High in Red Meat | 2.963 ± 0.013 | 1.883 ± 0.002 | 4.326 ± 0.025 | 0.44 |
| Diet Low in Whole Grains | 2.245 ± 0.005 | 2.141 ± 0.002 | 2.326 ± 0.013 | 0.92 |
| Diet Low in Milk | 1.502 ± 0.007 | 0.961 ± 0.001 | 2.109 ± 0.014 | 0.46 |
| Diet High in Processed Meat | 1.201 ± 0.002 | 1.108 ± 0.003 | 1.285 ± 0.005 | 0.86 |
| Diet Low in Fruits | 0.677 ± 0.001 | 0.790 ± 0.003 | 0.518 ± 0.004 | 1.53 |
| Diet High in Sodium | 0.579 ± 0.009 | 0.664 ± 0.008 | 0.462 ± 0.009 | 1.44 |
| Diet Low in Calcium | 0.540 ± 0.001 | 0.202 ± 0.002 | 0.920 ± 0.003 | 0.22 |
| Diet Low in Vegetables | 0.532 ± 0.001 | 0.712 ± 0.004 | 0.272 ± 0.001 | 2.62 |
| Diet Low in Fiber | 0.172 ± 0.001 | 0.151 ± 0.001 | 0.193 ± 0.002 | 0.79 |
| Note: Male/Female Ratio greater than 1 indicates that the dietary risk factor has a greater impact on males than females. | | | | |

| Table S4k. Gender Differences in Dietary Risk Factors' Attribution to Cancer Mortality (%) in High SDI Region, 2008-2015 (Mean ± Standard Error). | | | | |
| --- | --- | --- | --- | --- |
| Dietary Risk Factor | Both Sexes | Male | Female | Male/Female Ratio |
| Diet High in Red Meat | 2.869 ± 0.005 | 1.887 ± 0.004 | 4.115 ± 0.016 | 0.46 |
| Diet Low in Whole Grains | 2.205 ± 0.002 | 2.159 ± 0.005 | 2.215 ± 0.007 | 0.97 |
| Diet Low in Milk | 1.477 ± 0.003 | 0.995 ± 0.007 | 2.026 ± 0.003 | 0.49 |
| Diet High in Processed Meat | 1.168 ± 0.005 | 1.109 ± 0.002 | 1.213 ± 0.008 | 0.91 |
| Diet Low in Fruits | 0.663 ± 0.004 | 0.746 ± 0.007 | 0.549 ± 0.002 | 1.36 |
| Diet Low in Calcium | 0.537 ± 0.001 | 0.217 ± 0.001 | 0.902 ± 0.001 | 0.24 |
| Diet Low in Vegetables | 0.528 ± 0.002 | 0.718 ± 0.002 | 0.259 ± 0.002 | 2.78 |
| Diet High in Sodium | 0.505 ± 0.008 | 0.591 ± 0.008 | 0.389 ± 0.007 | 1.52 |
| Diet Low in Fiber | 0.165 ± 0.000 | 0.149 ± 0.000 | 0.181 ± 0.001 | 0.83 |
| Note: Male/Female Ratio greater than 1 indicates that the dietary risk factor has a greater impact on males than females. | | | | |

| Table S4l. Gender Differences in Dietary Risk Factors' Attribution to Cancer Mortality (%) in High SDI Region, 2016-2021 (Mean ± Standard Error). | | | | |
| --- | --- | --- | --- | --- |
| Dietary Risk Factor | Both Sexes | Male | Female | Male/Female Ratio |
| Diet High in Red Meat | 2.873 ± 0.002 | 1.926 ± 0.004 | 4.066 ± 0.003 | 0.47 |
| Diet Low in Whole Grains | 2.212 ± 0.001 | 2.196 ± 0.003 | 2.184 ± 0.002 | 1.01 |
| Diet Low in Milk | 1.494 ± 0.002 | 1.032 ± 0.003 | 2.020 ± 0.001 | 0.51 |
| Diet High in Processed Meat | 1.137 ± 0.004 | 1.096 ± 0.003 | 1.160 ± 0.005 | 0.94 |
| Diet Low in Fruits | 0.615 ± 0.005 | 0.672 ± 0.007 | 0.536 ± 0.002 | 1.25 |
| Diet Low in Calcium | 0.525 ± 0.002 | 0.214 ± 0.001 | 0.882 ± 0.003 | 0.24 |
| Diet Low in Vegetables | 0.524 ± 0.001 | 0.716 ± 0.001 | 0.257 ± 0.001 | 2.79 |
| Diet High in Sodium | 0.452 ± 0.003 | 0.533 ± 0.004 | 0.346 ± 0.003 | 1.54 |
| Diet Low in Fiber | 0.159 ± 0.001 | 0.146 ± 0.001 | 0.171 ± 0.001 | 0.85 |
| Note: Male/Female Ratio greater than 1 indicates that the dietary risk factor has a greater impact on males than females. | | | | |

| Table S4m. Gender Differences in Dietary Risk Factors' Attribution to Cancer Mortality (%) in High-middle SDI Region, 1990-1998 (Mean ± Standard Error). | | | | |
| --- | --- | --- | --- | --- |
| Dietary Risk Factor | Both Sexes | Male | Female | Male/Female Ratio |
| Diet High in Red Meat | 2.429 ± 0.012 | 1.476 ± 0.011 | 3.861 ± 0.015 | 0.38 |
| Diet Low in Whole Grains | 1.973 ± 0.008 | 1.756 ± 0.012 | 2.305 ± 0.003 | 0.76 |
| Diet Low in Milk | 1.327 ± 0.004 | 0.808 ± 0.003 | 2.036 ± 0.004 | 0.40 |
| Diet High in Sodium | 1.298 ± 0.023 | 1.414 ± 0.024 | 1.094 ± 0.022 | 1.29 |
| Diet Low in Vegetables | 1.122 ± 0.049 | 1.346 ± 0.055 | 0.748 ± 0.035 | 1.80 |
| Diet Low in Fruits | 0.867 ± 0.008 | 1.062 ± 0.012 | 0.540 ± 0.001 | 1.97 |
| Diet Low in Calcium | 0.814 ± 0.007 | 0.480 ± 0.008 | 1.268 ± 0.005 | 0.38 |
| Diet High in Processed Meat | 0.712 ± 0.003 | 0.577 ± 0.004 | 0.906 ± 0.004 | 0.64 |
| Diet Low in Fiber | 0.126 ± 0.001 | 0.108 ± 0.000 | 0.153 ± 0.001 | 0.71 |
| Note: Male/Female Ratio greater than 1 indicates that the dietary risk factor has a greater impact on males than females. | | | | |

| Table S4n. Gender Differences in Dietary Risk Factors' Attribution to Cancer Mortality (%) in High-middle SDI Region, 1999-2007 (Mean ± Standard Error). | | | | |
| --- | --- | --- | --- | --- |
| Dietary Risk Factor | Both Sexes | Male | Female | Male/Female Ratio |
| Diet High in Red Meat | 2.493 ± 0.007 | 1.574 ± 0.013 | 3.889 ± 0.004 | 0.40 |
| Diet Low in Whole Grains | 2.039 ± 0.008 | 1.870 ± 0.014 | 2.302 ± 0.003 | 0.81 |
| Diet Low in Milk | 1.350 ± 0.006 | 0.856 ± 0.010 | 2.035 ± 0.003 | 0.42 |
| Diet High in Sodium | 1.168 ± 0.013 | 1.289 ± 0.013 | 0.955 ± 0.014 | 1.35 |
| Diet Low in Calcium | 0.763 ± 0.005 | 0.439 ± 0.003 | 1.213 ± 0.009 | 0.36 |
| Diet Low in Fruits | 0.746 ± 0.019 | 0.894 ± 0.025 | 0.501 ± 0.009 | 1.78 |
| Diet High in Processed Meat | 0.713 ± 0.001 | 0.596 ± 0.002 | 0.882 ± 0.003 | 0.68 |
| Diet Low in Vegetables | 0.621 ± 0.043 | 0.775 ± 0.050 | 0.389 ± 0.031 | 1.99 |
| Diet Low in Fiber | 0.127 ± 0.001 | 0.110 ± 0.000 | 0.151 ± 0.002 | 0.73 |
| Note: Male/Female Ratio greater than 1 indicates that the dietary risk factor has a greater impact on males than females. | | | | |

| Table S4o. Gender Differences in Dietary Risk Factors' Attribution to Cancer Mortality (%) in High-middle SDI Region, 2008-2015 (Mean ± Standard Error). | | | | |
| --- | --- | --- | --- | --- |
| Dietary Risk Factor | Both Sexes | Male | Female | Male/Female Ratio |
| Diet High in Red Meat | 2.569 ± 0.006 | 1.704 ± 0.013 | 3.901 ± 0.003 | 0.44 |
| Diet Low in Whole Grains | 2.116 ± 0.007 | 2.002 ± 0.013 | 2.291 ± 0.003 | 0.87 |
| Diet Low in Milk | 1.452 ± 0.014 | 0.990 ± 0.017 | 2.102 ± 0.012 | 0.47 |
| Diet High in Sodium | 0.994 ± 0.016 | 1.121 ± 0.016 | 0.778 ± 0.016 | 1.44 |
| Diet High in Processed Meat | 0.707 ± 0.002 | 0.609 ± 0.001 | 0.847 ± 0.005 | 0.72 |
| Diet Low in Calcium | 0.698 ± 0.006 | 0.394 ± 0.005 | 1.129 ± 0.007 | 0.35 |
| Diet Low in Fruits | 0.589 ± 0.010 | 0.698 ± 0.013 | 0.411 ± 0.007 | 1.70 |
| Diet Low in Vegetables | 0.328 ± 0.016 | 0.423 ± 0.021 | 0.190 ± 0.010 | 2.23 |
| Diet Low in Fiber | 0.115 ± 0.001 | 0.104 ± 0.001 | 0.131 ± 0.002 | 0.79 |
| Note: Male/Female Ratio greater than 1 indicates that the dietary risk factor has a greater impact on males than females. | | | | |

| Table S4p. Gender Differences in Dietary Risk Factors' Attribution to Cancer Mortality (%) in High-middle SDI Region, 2016-2021 (Mean ± Standard Error). | | | | |
| --- | --- | --- | --- | --- |
| Dietary Risk Factor | Both Sexes | Male | Female | Male/Female Ratio |
| Diet High in Red Meat | 2.611 ± 0.002 | 1.799 ± 0.007 | 3.847 ± 0.009 | 0.47 |
| Diet Low in Whole Grains | 2.163 ± 0.002 | 2.098 ± 0.007 | 2.258 ± 0.005 | 0.93 |
| Diet Low in Milk | 1.576 ± 0.013 | 1.126 ± 0.014 | 2.199 ± 0.009 | 0.51 |
| Diet High in Sodium | 0.900 ± 0.006 | 1.024 ± 0.006 | 0.694 ± 0.004 | 1.48 |
| Diet High in Processed Meat | 0.685 ± 0.004 | 0.610 ± 0.003 | 0.792 ± 0.007 | 0.77 |
| Diet Low in Calcium | 0.669 ± 0.002 | 0.368 ± 0.002 | 1.092 ± 0.003 | 0.34 |
| Diet Low in Fruits | 0.539 ± 0.002 | 0.628 ± 0.005 | 0.396 ± 0.002 | 1.59 |
| Diet Low in Vegetables | 0.258 ± 0.003 | 0.334 ± 0.003 | 0.146 ± 0.002 | 2.30 |
| Diet Low in Fiber | 0.106 ± 0.001 | 0.099 ± 0.001 | 0.118 ± 0.002 | 0.84 |
| Note: Male/Female Ratio greater than 1 indicates that the dietary risk factor has a greater impact on males than females. | | | | |

| Table S4q. Gender Differences in Dietary Risk Factors' Attribution to Cancer Mortality (%) in Middle SDI Region, 1990-1998 (Mean ± Standard Error). | | | | |
| --- | --- | --- | --- | --- |
| Dietary Risk Factor | Both Sexes | Male | Female | Male/Female Ratio |
| Diet Low in Vegetables | 2.058 ± 0.063 | 2.384 ± 0.069 | 1.560 ± 0.054 | 1.53 |
| Diet High in Red Meat | 1.663 ± 0.011 | 1.034 ± 0.007 | 2.537 ± 0.020 | 0.41 |
| Diet High in Sodium | 1.595 ± 0.023 | 1.764 ± 0.024 | 1.334 ± 0.022 | 1.32 |
| Diet Low in Calcium | 1.434 ± 0.017 | 1.126 ± 0.015 | 1.823 ± 0.018 | 0.62 |
| Diet Low in Whole Grains | 1.421 ± 0.005 | 1.311 ± 0.007 | 1.562 ± 0.002 | 0.84 |
| Diet Low in Milk | 1.358 ± 0.001 | 0.825 ± 0.002 | 2.030 ± 0.002 | 0.41 |
| Diet Low in Fruits | 1.021 ± 0.010 | 1.225 ± 0.014 | 0.729 ± 0.006 | 1.68 |
| Diet Low in Fiber | 0.185 ± 0.003 | 0.163 ± 0.002 | 0.212 ± 0.003 | 0.77 |
| Diet High in Processed Meat | 0.135 ± 0.001 | 0.116 ± 0.001 | 0.158 ± 0.001 | 0.74 |
| Note: Male/Female Ratio greater than 1 indicates that the dietary risk factor has a greater impact on males than females. | | | | |

| Table S4r. Gender Differences in Dietary Risk Factors' Attribution to Cancer Mortality (%) in Middle SDI Region, 1999-2007 (Mean ± Standard Error). | | | | |
| --- | --- | --- | --- | --- |
| Dietary Risk Factor | Both Sexes | Male | Female | Male/Female Ratio |
| Diet High in Red Meat | 1.762 ± 0.014 | 1.117 ± 0.012 | 2.705 ± 0.020 | 0.41 |
| Diet Low in Whole Grains | 1.476 ± 0.009 | 1.392 ± 0.012 | 1.584 ± 0.004 | 0.88 |
| Diet High in Sodium | 1.432 ± 0.018 | 1.600 ± 0.018 | 1.163 ± 0.020 | 1.38 |
| Diet Low in Milk | 1.382 ± 0.006 | 0.876 ± 0.009 | 2.050 ± 0.004 | 0.43 |
| Diet Low in Vegetables | 1.321 ± 0.073 | 1.563 ± 0.083 | 0.957 ± 0.058 | 1.63 |
| Diet Low in Calcium | 1.310 ± 0.007 | 1.022 ± 0.005 | 1.695 ± 0.008 | 0.60 |
| Diet Low in Fruits | 0.890 ± 0.016 | 1.061 ± 0.019 | 0.633 ± 0.012 | 1.68 |
| Diet Low in Fiber | 0.167 ± 0.001 | 0.150 ± 0.001 | 0.190 ± 0.001 | 0.79 |
| Diet High in Processed Meat | 0.152 ± 0.003 | 0.133 ± 0.003 | 0.177 ± 0.003 | 0.75 |
| Note: Male/Female Ratio greater than 1 indicates that the dietary risk factor has a greater impact on males than females. | | | | |

| Table S4s. Gender Differences in Dietary Risk Factors' Attribution to Cancer Mortality (%) in Middle SDI Region, 2008-2015 (Mean ± Standard Error). | | | | |
| --- | --- | --- | --- | --- |
| Dietary Risk Factor | Both Sexes | Male | Female | Male/Female Ratio |
| Diet High in Red Meat | 1.929 ± 0.017 | 1.274 ± 0.017 | 2.925 ± 0.020 | 0.44 |
| Diet Low in Whole Grains | 1.579 ± 0.010 | 1.539 ± 0.016 | 1.623 ± 0.002 | 0.95 |
| Diet Low in Milk | 1.459 ± 0.007 | 1.000 ± 0.013 | 2.091 ± 0.002 | 0.48 |
| Diet High in Sodium | 1.200 ± 0.025 | 1.362 ± 0.026 | 0.936 ± 0.022 | 1.45 |
| Diet Low in Calcium | 1.199 ± 0.015 | 0.929 ± 0.013 | 1.575 ± 0.016 | 0.59 |
| Diet Low in Vegetables | 0.771 ± 0.036 | 0.925 ± 0.042 | 0.542 ± 0.025 | 1.71 |
| Diet Low in Fruits | 0.768 ± 0.009 | 0.912 ± 0.011 | 0.544 ± 0.006 | 1.68 |
| Diet High in Processed Meat | 0.192 ± 0.004 | 0.173 ± 0.004 | 0.216 ± 0.004 | 0.80 |
| Diet Low in Fiber | 0.150 ± 0.002 | 0.137 ± 0.002 | 0.166 ± 0.003 | 0.83 |
| Note: Male/Female Ratio greater than 1 indicates that the dietary risk factor has a greater impact on males than females. | | | | |

| Table S4t. Gender Differences in Dietary Risk Factors' Attribution to Cancer Mortality (%) in Middle SDI Region, 2016-2021 (Mean ± Standard Error). | | | | |
| --- | --- | --- | --- | --- |
| Dietary Risk Factor | Both Sexes | Male | Female | Male/Female Ratio |
| Diet High in Red Meat | 2.071 ± 0.015 | 1.399 ± 0.012 | 3.060 ± 0.013 | 0.46 |
| Diet Low in Whole Grains | 1.663 ± 0.009 | 1.667 ± 0.014 | 1.642 ± 0.003 | 1.02 |
| Diet Low in Milk | 1.541 ± 0.011 | 1.105 ± 0.012 | 2.119 ± 0.005 | 0.52 |
| Diet Low in Calcium | 1.128 ± 0.002 | 0.866 ± 0.002 | 1.478 ± 0.006 | 0.59 |
| Diet High in Sodium | 1.046 ± 0.012 | 1.196 ± 0.013 | 0.812 ± 0.008 | 1.47 |
| Diet Low in Fruits | 0.724 ± 0.002 | 0.850 ± 0.004 | 0.533 ± 0.003 | 1.59 |
| Diet Low in Vegetables | 0.611 ± 0.006 | 0.741 ± 0.005 | 0.423 ± 0.006 | 1.75 |
| Diet High in Processed Meat | 0.216 ± 0.002 | 0.200 ± 0.002 | 0.235 ± 0.001 | 0.85 |
| Diet Low in Fiber | 0.140 ± 0.000 | 0.132 ± 0.000 | 0.149 ± 0.001 | 0.89 |
| Note: Male/Female Ratio greater than 1 indicates that the dietary risk factor has a greater impact on males than females. | | | | |

| Table S4u. Gender Differences in Dietary Risk Factors' Attribution to Cancer Mortality (%) in Low-middle SDI Region, 1990-1998 (Mean ± Standard Error). | | | | |
| --- | --- | --- | --- | --- |
| Dietary Risk Factor | Both Sexes | Male | Female | Male/Female Ratio |
| Diet Low in Calcium | 1.366 ± 0.006 | 0.986 ± 0.006 | 1.786 ± 0.005 | 0.55 |
| Diet High in Red Meat | 1.266 ± 0.011 | 0.636 ± 0.004 | 1.994 ± 0.021 | 0.32 |
| Diet Low in Vegetables | 1.232 ± 0.003 | 1.308 ± 0.002 | 1.135 ± 0.005 | 1.15 |
| Diet Low in Whole Grains | 1.200 ± 0.003 | 1.119 ± 0.004 | 1.289 ± 0.003 | 0.87 |
| Diet Low in Fruits | 1.110 ± 0.008 | 1.566 ± 0.016 | 0.573 ± 0.002 | 2.73 |
| Diet Low in Milk | 1.026 ± 0.001 | 0.407 ± 0.001 | 1.721 ± 0.004 | 0.24 |
| Diet High in Sodium | 0.947 ± 0.010 | 1.111 ± 0.012 | 0.751 ± 0.007 | 1.48 |
| Diet Low in Fiber | 0.211 ± 0.001 | 0.195 ± 0.001 | 0.228 ± 0.001 | 0.86 |
| Diet High in Processed Meat | 0.152 ± 0.000 | 0.140 ± 0.000 | 0.165 ± 0.000 | 0.85 |
| Note: Male/Female Ratio greater than 1 indicates that the dietary risk factor has a greater impact on males than females. | | | | |

| Table S4v. Gender Differences in Dietary Risk Factors' Attribution to Cancer Mortality (%) in Low-middle SDI Region, 1999-2007 (Mean ± Standard Error). | | | | |
| --- | --- | --- | --- | --- |
| Dietary Risk Factor | Both Sexes | Male | Female | Male/Female Ratio |
| Diet High in Red Meat | 1.410 ± 0.017 | 0.697 ± 0.008 | 2.232 ± 0.026 | 0.31 |
| Diet Low in Calcium | 1.332 ± 0.005 | 0.960 ± 0.004 | 1.740 ± 0.009 | 0.55 |
| Diet Low in Whole Grains | 1.250 ± 0.006 | 1.176 ± 0.007 | 1.328 ± 0.005 | 0.89 |
| Diet Low in Vegetables | 1.169 ± 0.008 | 1.256 ± 0.007 | 1.059 ± 0.009 | 1.19 |
| Diet Low in Milk | 1.061 ± 0.006 | 0.431 ± 0.006 | 1.760 ± 0.005 | 0.24 |
| Diet Low in Fruits | 1.015 ± 0.009 | 1.387 ± 0.016 | 0.582 ± 0.000 | 2.38 |
| Diet High in Sodium | 0.870 ± 0.006 | 1.020 ± 0.007 | 0.693 ± 0.005 | 1.47 |
| Diet Low in Fiber | 0.198 ± 0.003 | 0.186 ± 0.002 | 0.212 ± 0.003 | 0.87 |
| Diet High in Processed Meat | 0.162 ± 0.001 | 0.150 ± 0.001 | 0.176 ± 0.002 | 0.85 |
| Note: Male/Female Ratio greater than 1 indicates that the dietary risk factor has a greater impact on males than females. | | | | |

| Table S4w. Gender Differences in Dietary Risk Factors' Attribution to Cancer Mortality (%) in Low-middle SDI Region, 2008-2015 (Mean ± Standard Error). | | | | |
| --- | --- | --- | --- | --- |
| Dietary Risk Factor | Both Sexes | Male | Female | Male/Female Ratio |
| Diet High in Red Meat | 1.548 ± 0.014 | 0.761 ± 0.007 | 2.432 ± 0.015 | 0.31 |
| Diet Low in Whole Grains | 1.281 ± 0.003 | 1.223 ± 0.007 | 1.337 ± 0.002 | 0.92 |
| Diet Low in Calcium | 1.222 ± 0.012 | 0.878 ± 0.009 | 1.583 ± 0.019 | 0.55 |
| Diet Low in Milk | 1.095 ± 0.005 | 0.469 ± 0.004 | 1.761 ± 0.003 | 0.27 |
| Diet Low in Vegetables | 1.076 ± 0.011 | 1.173 ± 0.008 | 0.955 ± 0.014 | 1.23 |
| Diet Low in Fruits | 0.974 ± 0.001 | 1.304 ± 0.004 | 0.594 ± 0.006 | 2.20 |
| Diet High in Sodium | 0.807 ± 0.010 | 0.953 ± 0.011 | 0.637 ± 0.007 | 1.50 |
| Diet High in Processed Meat | 0.176 ± 0.001 | 0.162 ± 0.001 | 0.189 ± 0.001 | 0.85 |
| Diet Low in Fiber | 0.165 ± 0.003 | 0.157 ± 0.003 | 0.174 ± 0.004 | 0.91 |
| Note: Male/Female Ratio greater than 1 indicates that the dietary risk factor has a greater impact on males than females. | | | | |

| Table S4x. Gender Differences in Dietary Risk Factors' Attribution to Cancer Mortality (%) in Low-middle SDI Region, 2016-2021 (Mean ± Standard Error). | | | | |
| --- | --- | --- | --- | --- |
| Dietary Risk Factor | Both Sexes | Male | Female | Male/Female Ratio |
| Diet High in Red Meat | 1.647 ± 0.009 | 0.805 ± 0.002 | 2.555 ± 0.017 | 0.31 |
| Diet Low in Whole Grains | 1.306 ± 0.002 | 1.276 ± 0.004 | 1.328 ± 0.002 | 0.96 |
| Diet Low in Calcium | 1.160 ± 0.003 | 0.848 ± 0.002 | 1.469 ± 0.006 | 0.58 |
| Diet Low in Milk | 1.132 ± 0.002 | 0.511 ± 0.006 | 1.759 ± 0.002 | 0.29 |
| Diet Low in Vegetables | 1.021 ± 0.003 | 1.147 ± 0.001 | 0.874 ± 0.007 | 1.31 |
| Diet Low in Fruits | 0.987 ± 0.004 | 1.291 ± 0.004 | 0.648 ± 0.004 | 1.99 |
| Diet High in Sodium | 0.724 ± 0.008 | 0.862 ± 0.008 | 0.570 ± 0.007 | 1.51 |
| Diet High in Processed Meat | 0.184 ± 0.001 | 0.172 ± 0.001 | 0.194 ± 0.001 | 0.89 |
| Diet Low in Fiber | 0.154 ± 0.000 | 0.151 ± 0.000 | 0.157 ± 0.001 | 0.96 |
| Note: Male/Female Ratio greater than 1 indicates that the dietary risk factor has a greater impact on males than females. | | | | |

| Table S4y. Gender Differences in Dietary Risk Factors' Attribution to Cancer Mortality (%) in Low SDI Region, 1990-1998 (Mean ± Standard Error). | | | | |
| --- | --- | --- | --- | --- |
| Dietary Risk Factor | Both Sexes | Male | Female | Male/Female Ratio |
| Diet Low in Vegetables | 1.851 ± 0.003 | 1.979 ± 0.004 | 1.705 ± 0.001 | 1.16 |
| Diet Low in Calcium | 1.721 ± 0.004 | 1.575 ± 0.008 | 1.849 ± 0.003 | 0.85 |
| Diet High in Red Meat | 1.431 ± 0.002 | 0.894 ± 0.005 | 1.980 ± 0.005 | 0.45 |
| Diet Low in Whole Grains | 1.301 ± 0.002 | 1.403 ± 0.003 | 1.187 ± 0.001 | 1.18 |
| Diet Low in Milk | 0.845 ± 0.005 | 0.150 ± 0.006 | 1.534 ± 0.001 | 0.10 |
| Diet High in Sodium | 0.844 ± 0.005 | 1.008 ± 0.006 | 0.668 ± 0.004 | 1.51 |
| Diet Low in Fruits | 0.626 ± 0.001 | 0.967 ± 0.003 | 0.264 ± 0.002 | 3.66 |
| Diet High in Processed Meat | 0.206 ± 0.000 | 0.215 ± 0.001 | 0.195 ± 0.000 | 1.11 |
| Diet Low in Fiber | 0.080 ± 0.001 | 0.080 ± 0.001 | 0.079 ± 0.001 | 1.01 |
| Note: Male/Female Ratio greater than 1 indicates that the dietary risk factor has a greater impact on males than females. | | | | |

| Table S4z. Gender Differences in Dietary Risk Factors' Attribution to Cancer Mortality (%) in Low SDI Region, 1999-2007 (Mean ± Standard Error). | | | | |
| --- | --- | --- | --- | --- |
| Dietary Risk Factor | Both Sexes | Male | Female | Male/Female Ratio |
| Diet Low in Vegetables | 1.811 ± 0.009 | 1.931 ± 0.010 | 1.673 ± 0.008 | 1.15 |
| Diet Low in Calcium | 1.653 ± 0.010 | 1.455 ± 0.013 | 1.839 ± 0.006 | 0.79 |
| Diet High in Red Meat | 1.488 ± 0.011 | 0.886 ± 0.004 | 2.102 ± 0.018 | 0.42 |
| Diet Low in Whole Grains | 1.302 ± 0.003 | 1.393 ± 0.003 | 1.203 ± 0.003 | 1.16 |
| Diet Low in Milk | 0.815 ± 0.002 | 0.076 ± 0.006 | 1.547 ± 0.003 | 0.05 |
| Diet High in Sodium | 0.792 ± 0.004 | 0.942 ± 0.005 | 0.633 ± 0.002 | 1.49 |
| Diet Low in Fruits | 0.622 ± 0.001 | 0.941 ± 0.003 | 0.290 ± 0.003 | 3.24 |
| Diet High in Processed Meat | 0.210 ± 0.001 | 0.217 ± 0.001 | 0.201 ± 0.001 | 1.08 |
| Diet Low in Fiber | 0.088 ± 0.001 | 0.085 ± 0.001 | 0.091 ± 0.001 | 0.94 |
| Note: Male/Female Ratio greater than 1 indicates that the dietary risk factor has a greater impact on males than females. | | | | |

| Table S4aa. Gender Differences in Dietary Risk Factors' Attribution to Cancer Mortality (%) in Low SDI Region, 2008-2015 (Mean ± Standard Error). | | | | |
| --- | --- | --- | --- | --- |
| Dietary Risk Factor | Both Sexes | Male | Female | Male/Female Ratio |
| Diet Low in Vegetables | 1.695 ± 0.014 | 1.821 ± 0.010 | 1.551 ± 0.017 | 1.17 |
| Diet High in Red Meat | 1.593 ± 0.012 | 0.918 ± 0.004 | 2.276 ± 0.017 | 0.40 |
| Diet Low in Calcium | 1.528 ± 0.011 | 1.318 ± 0.009 | 1.726 ± 0.013 | 0.76 |
| Diet Low in Whole Grains | 1.313 ± 0.003 | 1.418 ± 0.006 | 1.204 ± 0.001 | 1.18 |
| Diet Low in Milk | 0.810 ± 0.003 | 0.053 ± 0.003 | 1.548 ± 0.002 | 0.03 |
| Diet High in Sodium | 0.755 ± 0.006 | 0.898 ± 0.007 | 0.606 ± 0.004 | 1.48 |
| Diet Low in Fruits | 0.621 ± 0.001 | 0.916 ± 0.001 | 0.319 ± 0.004 | 2.87 |
| Diet High in Processed Meat | 0.219 ± 0.001 | 0.229 ± 0.001 | 0.208 ± 0.000 | 1.10 |
| Diet Low in Fiber | 0.079 ± 0.001 | 0.076 ± 0.001 | 0.082 ± 0.001 | 0.92 |
| Note: Male/Female Ratio greater than 1 indicates that the dietary risk factor has a greater impact on males than females. | | | | |

| Table S4ab. Gender Differences in Dietary Risk Factors' Attribution to Cancer Mortality (%) in Low SDI Region, 2016-2021 (Mean ± Standard Error). | | | | |
| --- | --- | --- | --- | --- |
| Dietary Risk Factor | Both Sexes | Male | Female | Male/Female Ratio |
| Diet High in Red Meat | 1.716 ± 0.014 | 0.967 ± 0.006 | 2.450 ± 0.020 | 0.39 |
| Diet Low in Vegetables | 1.639 ± 0.002 | 1.796 ± 0.004 | 1.465 ± 0.007 | 1.23 |
| Diet Low in Calcium | 1.489 ± 0.003 | 1.301 ± 0.001 | 1.657 ± 0.006 | 0.79 |
| Diet Low in Whole Grains | 1.346 ± 0.003 | 1.484 ± 0.007 | 1.209 ± 0.000 | 1.23 |
| Diet Low in Milk | 0.839 ± 0.004 | 0.067 ± 0.004 | 1.561 ± 0.001 | 0.04 |
| Diet High in Sodium | 0.689 ± 0.008 | 0.817 ± 0.009 | 0.560 ± 0.006 | 1.46 |
| Diet Low in Fruits | 0.632 ± 0.001 | 0.919 ± 0.002 | 0.349 ± 0.002 | 2.63 |
| Diet High in Processed Meat | 0.227 ± 0.001 | 0.241 ± 0.001 | 0.212 ± 0.001 | 1.14 |
| Diet Low in Fiber | 0.072 ± 0.001 | 0.071 ± 0.000 | 0.073 ± 0.001 | 0.97 |
| Note: Male/Female Ratio greater than 1 indicates that the dietary risk factor has a greater impact on males than females. | | | | |

**Appendixes 5**

| Table S5a. Average Attribution Proportion (%) of Dietary Risk Factors to All Cancer Mortality by Region (1990-2021) (Mean ± Standard Error). | | | | | | | |
| --- | --- | --- | --- | --- | --- | --- | --- |
|  | | | | | | | |
| Dietary Risk Factor | China | Global | High SDI | High-middle SDI | Middle SDI | Low-middle SDI | Low SDI |
| Diet High in Red Meat | 1.735 ± 0.024 | 2.355 ± 0.003 | 2.955 ± 0.016 | 2.516 ± 0.013 | 1.834 ± 0.028 | 1.448 ± 0.026 | 1.541 ± 0.019 |
| Diet High in Sodium | 1.689 ± 0.043 | 0.955 ± 0.023 | 0.563 ± 0.015 | 1.111 ± 0.028 | 1.347 ± 0.038 | 0.849 ± 0.015 | 0.778 ± 0.010 |
| Diet Low in Whole Grains | 1.601 ± 0.017 | 1.893 ± 0.001 | 2.236 ± 0.005 | 2.063 ± 0.013 | 1.521 ± 0.017 | 1.254 ± 0.007 | 1.313 ± 0.003 |
| Diet Low in Milk | 1.563 ± 0.014 | 1.420 ± 0.006 | 1.516 ± 0.008 | 1.412 ± 0.017 | 1.424 ± 0.013 | 1.073 ± 0.007 | 0.827 ± 0.003 |
| Diet Low in Vegetables | 1.288 ± 0.164 | 0.834 ± 0.044 | 0.531 ± 0.001 | 0.620 ± 0.064 | 1.257 ± 0.105 | 1.136 ± 0.015 | 1.761 ± 0.015 |
| Diet Low in Calcium | 0.974 ± 0.034 | 0.910 ± 0.008 | 0.545 ± 0.003 | 0.743 ± 0.010 | 1.283 ± 0.021 | 1.282 ± 0.015 | 1.610 ± 0.017 |
| Diet Low in Fruits | 0.915 ± 0.031 | 0.758 ± 0.013 | 0.670 ± 0.006 | 0.702 ± 0.024 | 0.865 ± 0.021 | 1.026 ± 0.010 | 0.625 ± 0.001 |
| Diet High in Processed Meat | 0.165 ± 0.007 | 0.655 ± 0.006 | 1.180 ± 0.005 | 0.706 ± 0.002 | 0.169 ± 0.006 | 0.167 ± 0.002 | 0.214 ± 0.001 |
| Diet Low in Fiber | 0.097 ± 0.004 | 0.158 ± 0.003 | 0.174 ± 0.003 | 0.120 ± 0.002 | 0.163 ± 0.003 | 0.185 ± 0.004 | 0.080 ± 0.001 |
| Note: Higher values indicate greater impact of the dietary factor on cancer mortality. | | | | | | | |

| TableS5b. Comparison of Dietary Risk Factors' Average Ranking and Attribution Proportion (%) by Region (1990-2021). | | | | | | | | | | | | | | |
| --- | --- | --- | --- | --- | --- | --- | --- | --- | --- | --- | --- | --- | --- | --- |
| Dietary Risk Factor | Rank (China) | Percent (China) | Rank (Global) | Percent (Global) | Rank (High SDI) | Percent (High SDI) | Rank (High-middle SDI) | Percent (High-middle SDI) | Rank (Middle SDI) | Percent (Middle SDI) | Rank (Low-middle SDI) | Percent (Low-middle SDI) | Rank (Low SDI) | Percent (Low SDI) |
| Diet High in Processed Meat | 8.2 | 0.165 | 7.7 | 0.655 | 4.0 | 1.180 | 6.5 | 0.706 | 8.5 | 0.169 | 8.6 | 0.167 | 8.0 | 0.214 |
| Diet High in Red Meat | 1.9 | 1.735 | 1.0 | 2.355 | 1.0 | 2.955 | 1.0 | 2.516 | 1.4 | 1.834 | 1.4 | 1.448 | 2.4 | 1.541 |
| Diet High in Sodium | 2.5 | 1.689 | 4.6 | 0.955 | 6.9 | 0.563 | 3.9 | 1.111 | 3.7 | 1.347 | 7.0 | 0.849 | 5.8 | 0.778 |
| Diet Low in Calcium | 5.5 | 0.974 | 5.0 | 0.910 | 6.7 | 0.545 | 6.1 | 0.743 | 4.7 | 1.283 | 2.1 | 1.282 | 2.4 | 1.610 |
| Diet Low in Fiber | 8.8 | 0.097 | 9.0 | 0.158 | 9.0 | 0.174 | 9.0 | 0.120 | 8.5 | 0.163 | 8.4 | 0.185 | 9.0 | 0.080 |
| Diet Low in Fruits | 6.5 | 0.915 | 6.5 | 0.758 | 5.0 | 0.670 | 6.4 | 0.702 | 6.7 | 0.865 | 5.7 | 1.026 | 7.0 | 0.625 |
| Diet Low in Milk | 3.9 | 1.563 | 3.0 | 1.420 | 3.0 | 1.516 | 3.1 | 1.412 | 4.2 | 1.424 | 5.0 | 1.073 | 5.2 | 0.827 |
| Diet Low in Vegetables | 4.5 | 1.288 | 6.2 | 0.834 | 7.4 | 0.531 | 7.0 | 0.620 | 4.5 | 1.257 | 4.0 | 1.136 | 1.2 | 1.761 |
| Diet Low in Whole Grains | 3.1 | 1.601 | 2.0 | 1.893 | 2.0 | 2.236 | 2.0 | 2.063 | 2.9 | 1.521 | 2.8 | 1.254 | 4.0 | 1.313 |
| Note: Rank 1 indicates the highest attribution proportion; lower values of rank indicate greater impact. | | | | | | | | | | | | | | |

| Table S5c. Regional Comparison of Dietary Risks' Impact on Cancer Mortality Rates (per 100,000) Over Time (1990-2021). | | | | | | | | | |
| --- | --- | --- | --- | --- | --- | --- | --- | --- | --- |
| Region | 1990 | 1995 | 2000 | 2005 | 2010 | 2015 | 2020 | 2021 | Change Rate (%) |
| China | 18.441 | 16.408 | 14.282 | 12.759 | 10.511 | 8.965 | 8.702 | 8.662 | -53.03 |
| Global | 12.237 | 11.632 | 10.711 | 9.903 | 8.968 | 8.318 | 7.937 | 7.899 | -35.45 |
| High SDI | 13.809 | 13.190 | 12.226 | 11.292 | 10.417 | 9.689 | 8.913 | 8.892 | -35.61 |
| High-middle SDI | 14.789 | 14.124 | 13.071 | 12.121 | 10.685 | 9.744 | 9.247 | 9.163 | -38.04 |
| Middle SDI | 11.925 | 11.117 | 9.981 | 9.120 | 8.101 | 7.374 | 7.237 | 7.223 | -39.44 |
| Low-middle SDI | 5.304 | 5.351 | 5.276 | 5.284 | 5.238 | 5.256 | 5.278 | 5.232 | -1.36 |
| Low SDI | 6.614 | 6.592 | 6.272 | 5.948 | 5.713 | 5.828 | 5.832 | 5.808 | -12.19 |
| Note: Values represent mortality rates attributable to dietary risks per 100,000 population. Negative change rate indicates a decrease over time. | | | | | | | | | |

| Table S5d. Attribution Proportion (%) of Dietary Risks to Different Cancer Types by Region (1990-2021) (Mean ± Standard Error). | | | | | | | |
| --- | --- | --- | --- | --- | --- | --- | --- |
| Cancer Type | China | Global | High SDI | High-middle SDI | Middle SDI | Low-middle SDI | Low SDI |
| Colorectal Cancer | 39.158 ± 0.248 | 39.824 ± 0.099 | 39.306 ± 0.017 | 39.272 ± 0.126 | 40.972 ± 0.249 | 40.852 ± 0.206 | 46.298 ± 0.187 |
| Breast Cancer | 13.580 ± 0.009 | 12.447 ± 0.038 | 13.624 ± 0.002 | 13.515 ± 0.007 | 11.632 ± 0.014 | 9.473 ± 0.029 | 10.066 ± 0.009 |
| Esophageal Cancer | 9.810 ± 1.129 | 14.243 ± 0.640 | 16.747 ± 0.153 | 9.580 ± 0.949 | 13.553 ± 0.857 | 23.064 ± 0.057 | 25.463 ± 0.048 |
| Stomach Cancer | 8.305 ± 0.001 | 7.929 ± 0.003 | 7.893 ± 0.009 | 7.893 ± 0.006 | 8.120 ± 0.006 | 7.738 ± 0.004 | 7.153 ± 0.004 |
| Tracheal, Bronchus, and Lung Cancer | 3.970 ± 0.232 | 3.848 ± 0.090 | 2.882 ± 0.026 | 3.171 ± 0.138 | 4.657 ± 0.186 | 9.506 ± 0.140 | 10.103 ± 0.041 |
| Prostate Cancer | -10.143 ± 0.188 | -3.404 ± 0.038 | -0.497 ± 0.008 | -2.108 ± 0.011 | -6.575 ± 0.070 | -7.856 ± 0.085 | -10.447 ± 0.051 |
| Note: Higher values indicate greater impact of dietary risks on specific cancer type. | | | | | | | |

| Table S5e. Regional Comparison of Major Dietary Risk Factors' Annual Percent Change (AAPC) (1990-2021). | | | | | |
| --- | --- | --- | --- | --- | --- |
| Region | Diet Low in Vegetables | Diet High in Sodium | Diet Low in Fruits | Diet High in Red Meat | Diet Low in Calcium |
| China | -0.096 (-0.105, -0.087) | -0.025 (-0.027, -0.024) | -0.018 (-0.019, -0.018) | 0.014 (0.012, 0.015) | -0.020 (-0.022, -0.019) |
| Global | -0.026 (-0.028, -0.023) | -0.014 (-0.014, -0.013) | -0.008 (-0.008, -0.008) | 0.001 (0.000, 0.002) | -0.005 (-0.005, -0.004) |
| High SDI | -0.001 (-0.001, -0.000) | -0.009 (-0.010, -0.009) | -0.003 (-0.004, -0.003) | -0.009 (-0.010, -0.008) | -0.002 (-0.002, -0.001) |
| High-middle SDI | -0.037 (-0.041, -0.033) | -0.017 (-0.018, -0.016) | -0.014 (-0.015, -0.013) | 0.008 (0.007, 0.008) | -0.006 (-0.006, -0.006) |
| Middle SDI | -0.062 (-0.067, -0.057) | -0.023 (-0.024, -0.022) | -0.013 (-0.013, -0.012) | 0.017 (0.016, 0.017) | -0.013 (-0.013, -0.012) |
| Low-middle SDI | -0.009 (-0.009, -0.008) | -0.009 (-0.009, -0.008) | -0.005 (-0.007, -0.004) | 0.016 (0.015, 0.016) | -0.009 (-0.009, -0.008) |
| Low SDI | -0.009 (-0.010, -0.008) | -0.006 (-0.006, -0.006) | 0.000 (-0.000, 0.000) | 0.011 (0.010, 0.012) | -0.010 (-0.011, -0.009) |
| Note: AAPC = Average Annual Percent Change (95% Confidence Interval); Negative values indicate a decrease in risk. | | | | | |

**Appendixes 6**

| Table S6a. Regional Comparison of Diet Low in Vegetables Attribution Proportion to Cancer Mortality (1990-2021). | | | | | | | |
| --- | --- | --- | --- | --- | --- | --- | --- |
| Region | Mean (%) | SD (%) | Min (%) | Max (%) | 1990 (%) | 2021 (%) | Change Rate (%) |
| China | 1.288 | 0.928 | 0.304 | 2.956 | 2.956 | 0.315 | -89.36 |
| Global | 0.834 | 0.248 | 0.570 | 1.277 | 1.277 | 0.573 | -55.11 |
| High SDI | 0.531 | 0.008 | 0.519 | 0.551 | 0.551 | 0.521 | -5.35 |
| High-middle SDI | 0.620 | 0.362 | 0.251 | 1.304 | 1.304 | 0.254 | -80.51 |
| Middle SDI | 1.257 | 0.594 | 0.599 | 2.288 | 2.288 | 0.607 | -73.48 |
| Low-middle SDI | 1.136 | 0.083 | 1.011 | 1.244 | 1.244 | 1.011 | -18.74 |
| Low SDI | 1.761 | 0.087 | 1.634 | 1.864 | 1.864 | 1.634 | -12.33 |
| Note: Values represent attribution proportion (%) of dietary risk factor to all cancer mortality. Negative change rate indicates a decrease over time. | | | | | | | |

| Table S6b. Regional Comparison of Diet Low in Calcium Attribution Proportion to Cancer Mortality (1990-2021). | | | | | | | |
| --- | --- | --- | --- | --- | --- | --- | --- |
| Region | Mean (%) | SD (%) | Min (%) | Max (%) | 1990 (%) | 2021 (%) | Change Rate (%) |
| China | 0.974 | 0.195 | 0.735 | 1.350 | 1.350 | 0.738 | -45.37 |
| Global | 0.910 | 0.045 | 0.850 | 0.991 | 0.991 | 0.857 | -13.53 |
| High SDI | 0.545 | 0.019 | 0.519 | 0.589 | 0.589 | 0.520 | -11.73 |
| High-middle SDI | 0.743 | 0.058 | 0.663 | 0.842 | 0.842 | 0.670 | -20.47 |
| Middle SDI | 1.283 | 0.121 | 1.119 | 1.496 | 1.496 | 1.128 | -24.59 |
| Low-middle SDI | 1.282 | 0.084 | 1.149 | 1.394 | 1.394 | 1.157 | -17.02 |
| Low SDI | 1.610 | 0.096 | 1.479 | 1.733 | 1.733 | 1.486 | -14.27 |
| Note: Values represent attribution proportion (%) of dietary risk factor to all cancer mortality. Negative change rate indicates a decrease over time. | | | | | | | |

| Table S6c. Regional Comparison of Diet Low in Whole Grains Attribution Proportion to Cancer Mortality (1990-2021). | | | | | | | |
| --- | --- | --- | --- | --- | --- | --- | --- |
| Region | Mean (%) | SD (%) | Min (%) | Max (%) | 1990 (%) | 2021 (%) | Change Rate (%) |
| China | 1.601 | 0.098 | 1.503 | 1.798 | 1.503 | 1.798 | 19.58 |
| Global | 1.893 | 0.008 | 1.883 | 1.911 | 1.886 | 1.908 | 1.16 |
| High SDI | 2.236 | 0.028 | 2.194 | 2.282 | 2.278 | 2.213 | -2.86 |
| High-middle SDI | 2.063 | 0.075 | 1.938 | 2.168 | 1.938 | 2.165 | 11.71 |
| Middle SDI | 1.521 | 0.093 | 1.397 | 1.688 | 1.397 | 1.688 | 20.83 |
| Low-middle SDI | 1.254 | 0.041 | 1.188 | 1.310 | 1.188 | 1.308 | 10.06 |
| Low SDI | 1.313 | 0.018 | 1.291 | 1.354 | 1.310 | 1.354 | 3.37 |
| Note: Values represent attribution proportion (%) of dietary risk factor to all cancer mortality. Negative change rate indicates a decrease over time. | | | | | | | |

| Table S6d. Regional Comparison of Diet High in Processed Meat Attribution Proportion to Cancer Mortality (1990-2021). | | | | | | | |
| --- | --- | --- | --- | --- | --- | --- | --- |
| Region | Mean (%) | SD (%) | Min (%) | Max (%) | 1990 (%) | 2021 (%) | Change Rate (%) |
| China | 0.165 | 0.041 | 0.122 | 0.234 | 0.122 | 0.234 | 91.38 |
| Global | 0.655 | 0.034 | 0.587 | 0.699 | 0.698 | 0.587 | -15.86 |
| High SDI | 1.180 | 0.026 | 1.122 | 1.209 | 1.195 | 1.122 | -6.03 |
| High-middle SDI | 0.706 | 0.013 | 0.669 | 0.727 | 0.700 | 0.669 | -4.40 |
| Middle SDI | 0.169 | 0.032 | 0.132 | 0.220 | 0.132 | 0.220 | 67.05 |
| Low-middle SDI | 0.167 | 0.012 | 0.150 | 0.185 | 0.151 | 0.185 | 23.15 |
| Low SDI | 0.214 | 0.008 | 0.204 | 0.230 | 0.208 | 0.230 | 10.31 |
| Note: Values represent attribution proportion (%) of dietary risk factor to all cancer mortality. Negative change rate indicates a decrease over time. | | | | | | | |

| Table S6e. Regional Comparison of Diet Low in Fruits Attribution Proportion to Cancer Mortality (1990-2021) | | | | | | | |
| --- | --- | --- | --- | --- | --- | --- | --- |
| Region | Mean (%) | SD (%) | Min (%) | Max (%) | 1990 (%) | 2021 (%) | Change Rate (%) |
| China | 0.915 | 0.173 | 0.680 | 1.175 | 1.175 | 0.680 | -42.13 |
| Global | 0.758 | 0.075 | 0.663 | 0.880 | 0.880 | 0.663 | -24.64 |
| High SDI | 0.670 | 0.034 | 0.603 | 0.736 | 0.736 | 0.603 | -18.07 |
| High-middle SDI | 0.702 | 0.135 | 0.533 | 0.892 | 0.892 | 0.533 | -40.21 |
| Middle SDI | 0.865 | 0.120 | 0.720 | 1.061 | 1.061 | 0.720 | -32.11 |
| Low-middle SDI | 1.026 | 0.058 | 0.969 | 1.144 | 1.144 | 0.990 | -13.46 |
| Low SDI | 0.625 | 0.005 | 0.617 | 0.637 | 0.631 | 0.633 | 0.37 |
| Note: Values represent attribution proportion (%) of dietary risk factor to all cancer mortality. Negative change rate indicates a decrease over time. | | | | | | | |

| Table S6f. Regional Comparison of Diet High in Red Meat Attribution Proportion to Cancer Mortality (1990-2021). | | | | | | | |
| --- | --- | --- | --- | --- | --- | --- | --- |
| Region | Mean (%) | SD (%) | Min (%) | Max (%) | 1990 (%) | 2021 (%) | Change Rate (%) |
| China | 1.735 | 0.134 | 1.575 | 2.008 | 1.575 | 2.008 | 27.46 |
| Global | 2.355 | 0.018 | 2.326 | 2.393 | 2.352 | 2.393 | 1.75 |
| High SDI | 2.955 | 0.091 | 2.848 | 3.116 | 3.116 | 2.868 | -7.97 |
| High-middle SDI | 2.516 | 0.073 | 2.374 | 2.616 | 2.374 | 2.616 | 10.19 |
| Middle SDI | 1.834 | 0.157 | 1.612 | 2.113 | 1.612 | 2.113 | 31.12 |
| Low-middle SDI | 1.448 | 0.147 | 1.215 | 1.674 | 1.215 | 1.674 | 37.75 |
| Low SDI | 1.541 | 0.108 | 1.423 | 1.760 | 1.440 | 1.760 | 22.26 |
| Note: Values represent attribution proportion (%) of dietary risk factor to all cancer mortality. Negative change rate indicates a decrease over time. | | | | | | | |
