# Supplementary material for: Dietary risk factors and cancer mortality burden from 1990 to 2021: a comparative study of China and global regions with varying sociodemographic development levels based on the Global Burden of Disease database
Source: Front Nutr. 2025 Aug 5;12:1628792. doi: 10.3389/fnut.2025.1628792 (PMC12360945; doi:10.3389/fnut.2025.1628792)
Supplement: Supplementary file 2 [file Image_1.pdf]

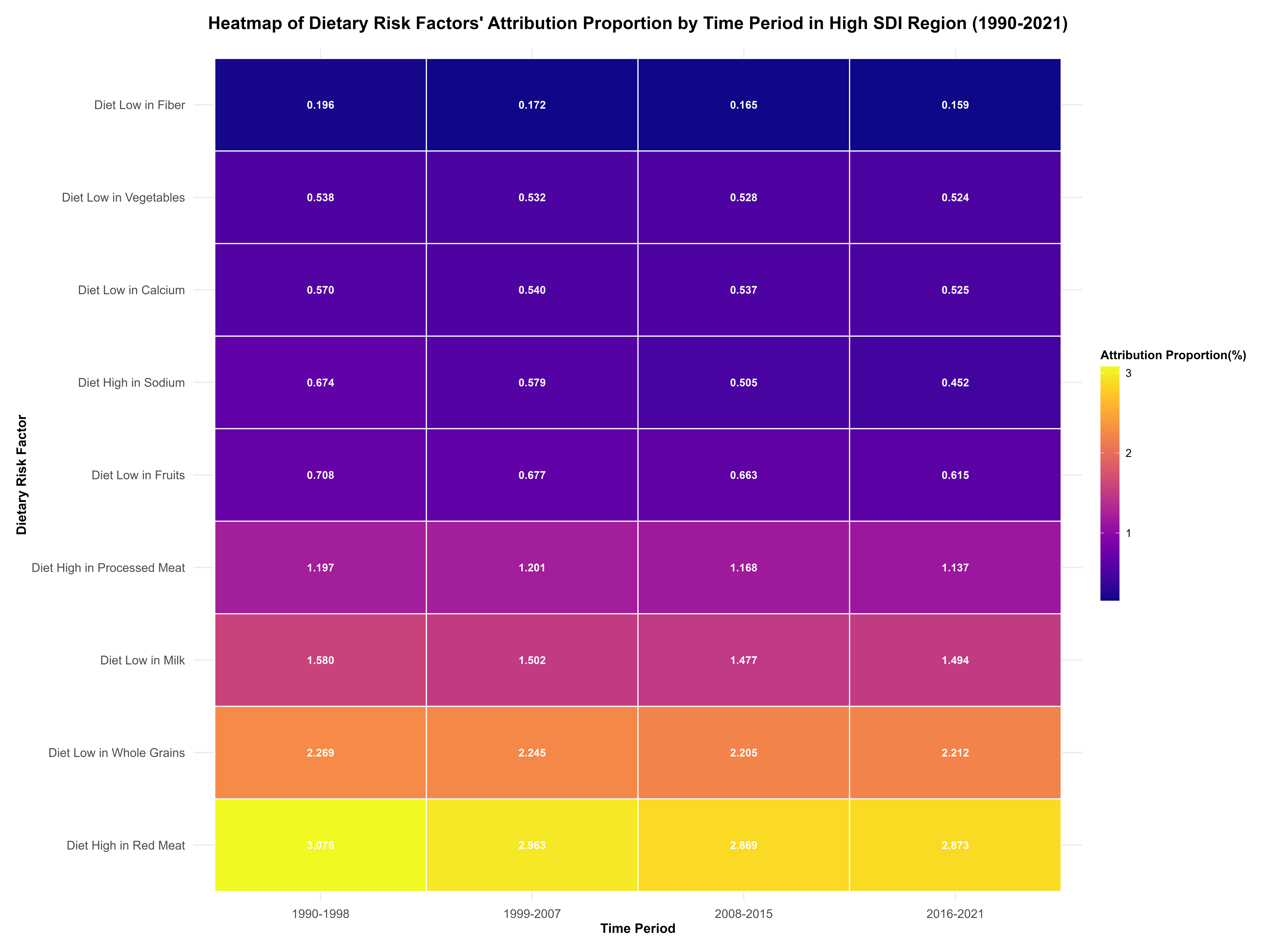
Heatmap of Dietary Risk Factors' Attribution Proportion by Time Period in High SDI Region (1990-2021)
Dietary Risk Factor
Diet Low in Fiber
Diet Low in Vegetables
Diet Low in Calcium
Diet High in Sodium
Diet Low in Fruits
Diet High in Processed Meat
Diet Low in Milk
Diet Low in Whole Grains
Diet High in Red Meat
0.196 0.172 0.165 0.159
0.538 0.532 0.528 0.524
0.570 0.540 0.537 0.525
0.674 0.579 0.505 0.452
0.708 0.677 0.663 0.615
1.197 1.201 1.168 1.137
1.580 1.502 1.477 1.494
2.269 2.245 2.205 2.212
3.076 2.963 2.869 2.873
1990-1998
1999-2007
2008-2015
2016-2021
Time Period
Attribution Proportion(%)
3
2
1

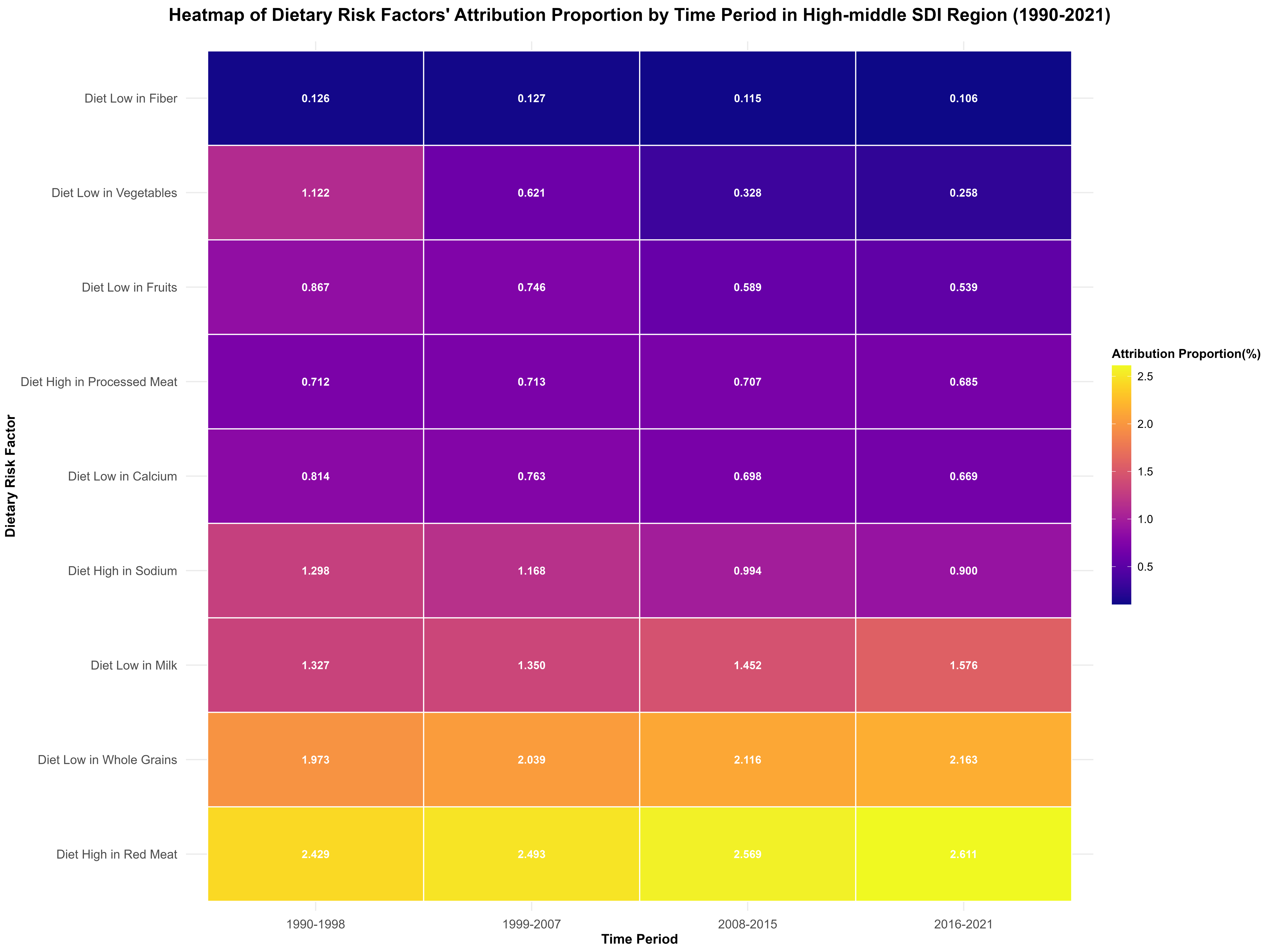
Heatmap of Dietary Risk Factors' Attribution Proportion by Time Period in High-middle SDI Region (1990-2021)
Dietary Risk Factor
Diet Low in Fiber
Diet Low in Vegetables
Diet Low in Fruits
Diet High in Processed Meat
Diet Low in Calcium
Diet High in Sodium
Diet Low in Milk
Diet Low in Whole Grains
Diet High in Red Meat
0.126
0.127
0.115
0.106
1.122
0.621
0.328
0.258
0.867
0.746
0.589
0.539
0.712
0.713
0.707
0.685
0.814
0.763
0.698
0.669
1.298
1.168
0.994
0.900
1.327
1.350
1.452
1.576
1.973
2.039
2.116
2.163
2.429
2.493
2.569
2.611
1990-1998
1999-2007
2008-2015
2016-2021
Time Period
Attribution Proportion(%)
2.5
2.0
1.5
1.0
0.5

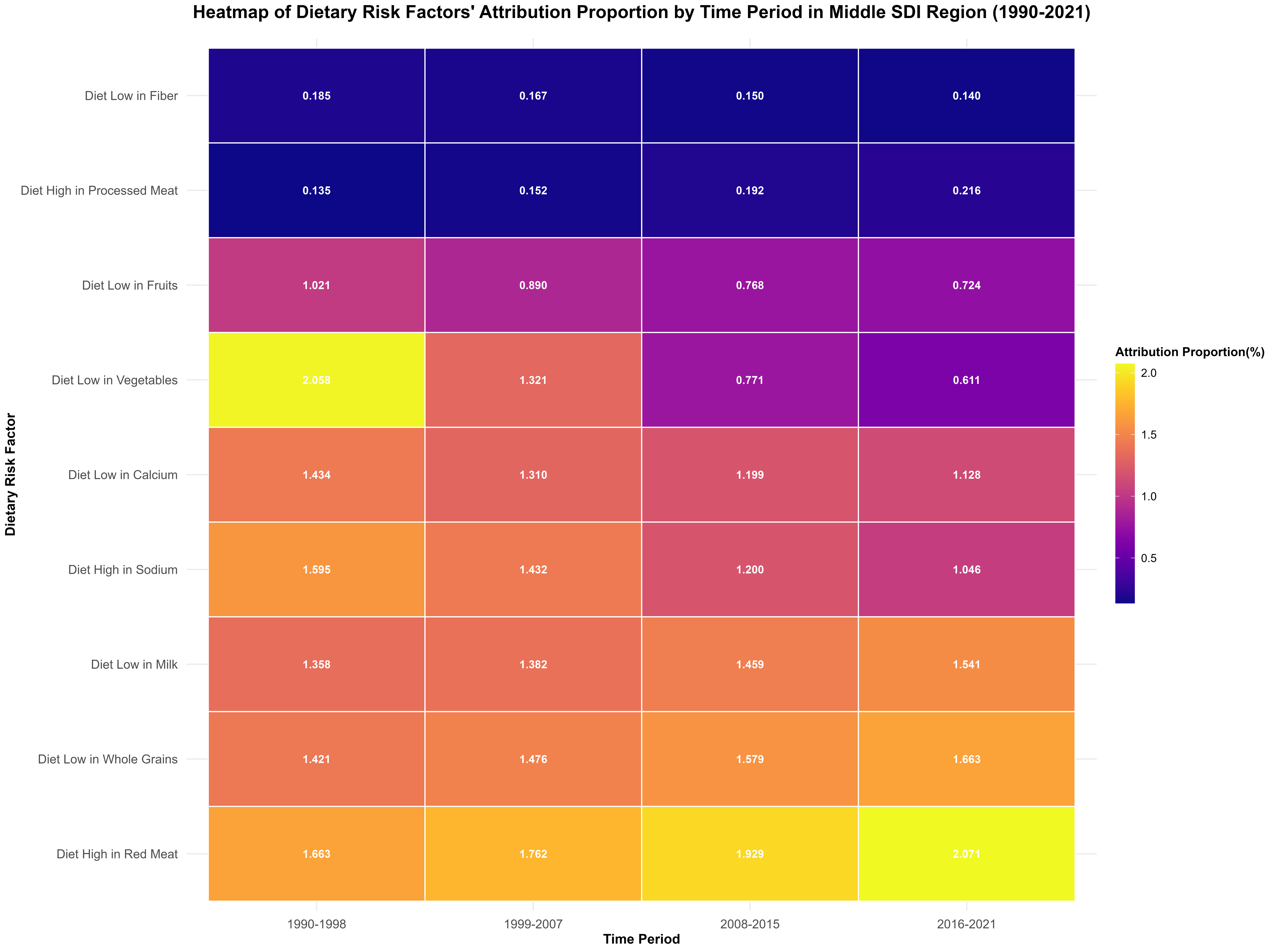

Heatmap of Dietary Risk Factors' Attribution Proportion by Time Period in Middle SDI Region (1990-2021)
Dietary Risk Factor
Time Period
1990-1998
1999-2007
2008-2015
2016-2021
Attribution Proportion(%)
0.5
1.0
1.5
2.0
Diet Low in Fiber 0.185 0.167 0.150 0.140
Diet High in Processed Meat 0.135 0.152 0.192 0.216
Diet Low in Fruits 1.021 0.890 0.768 0.724
Diet Low in Vegetables 2.058 1.321 0.771 0.611
Diet Low in Calcium 1.434 1.310 1.199 1.128
Diet High in Sodium 1.595 1.432 1.200 1.046
Diet Low in Milk 1.358 1.382 1.459 1.541
Diet Low in Whole Grains 1.421 1.476 1.579 1.663
Diet High in Red Meat 1.663 1.762 1.929 2.071

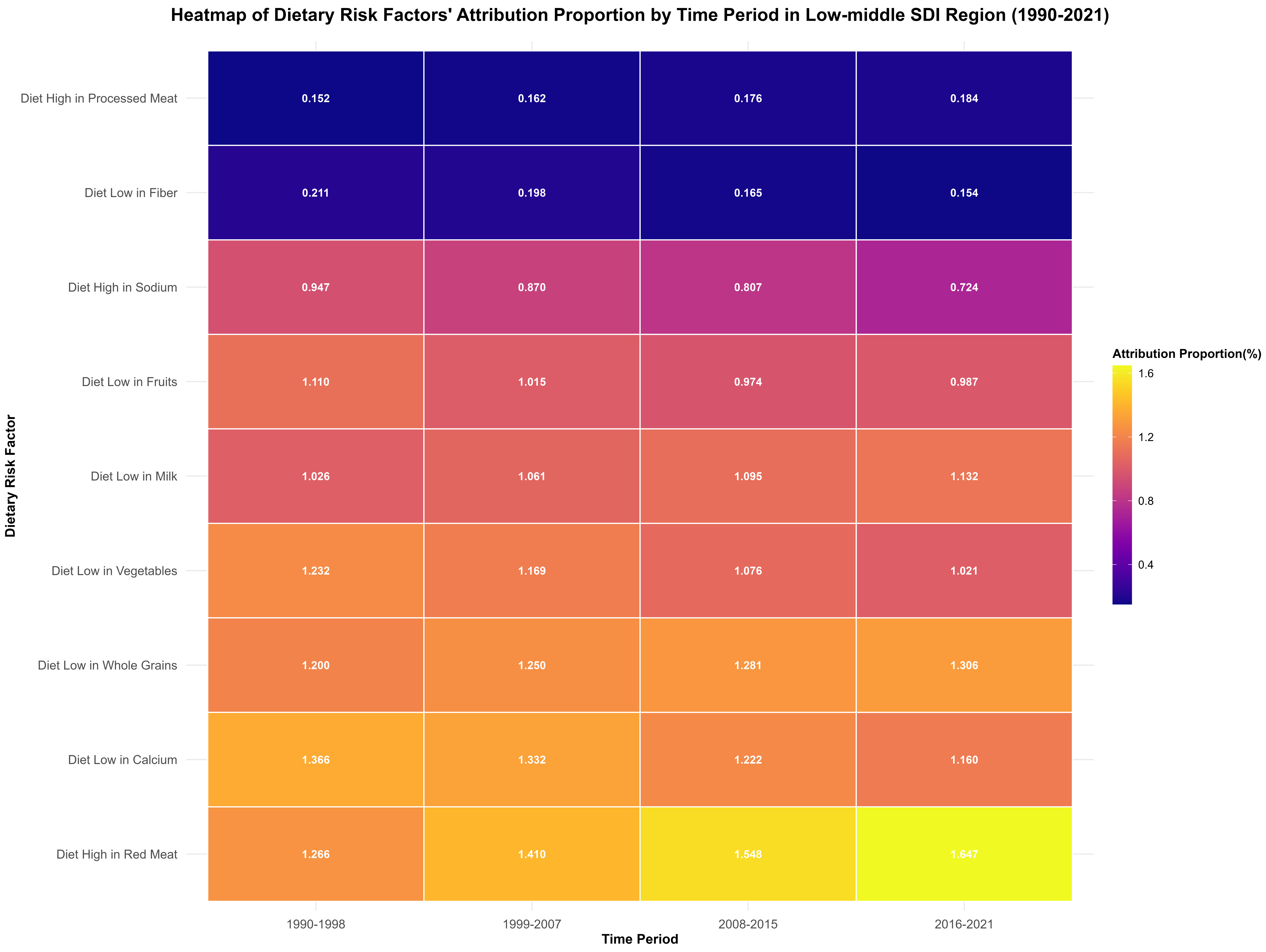

Heatmap of Dietary Risk Factors' Attribution Proportion by Time Period in Low-middle SDI Region (1990-2021)
Dietary Risk Factor
Diet High in Processed Meat
0.152
0.162
0.176
0.184
Diet Low in Fiber
0.211
0.198
0.165
0.154
Diet High in Sodium
0.947
0.870
0.807
0.724
Diet Low in Fruits
1.110
1.015
0.974
0.987
Diet Low in Milk
1.026
1.061
1.095
1.132
Diet Low in Vegetables
1.232
1.169
1.076
1.021
Diet Low in Whole Grains
1.200
1.250
1.281
1.306
Diet Low in Calcium
1.366
1.332
1.222
1.160
Diet High in Red Meat
1.266
1.410
1.548
1.647
1990-1998
1999-2007
2008-2015
2016-2021
Time Period
Attribution Proportion(%)
1.6
1.2
0.8
0.4

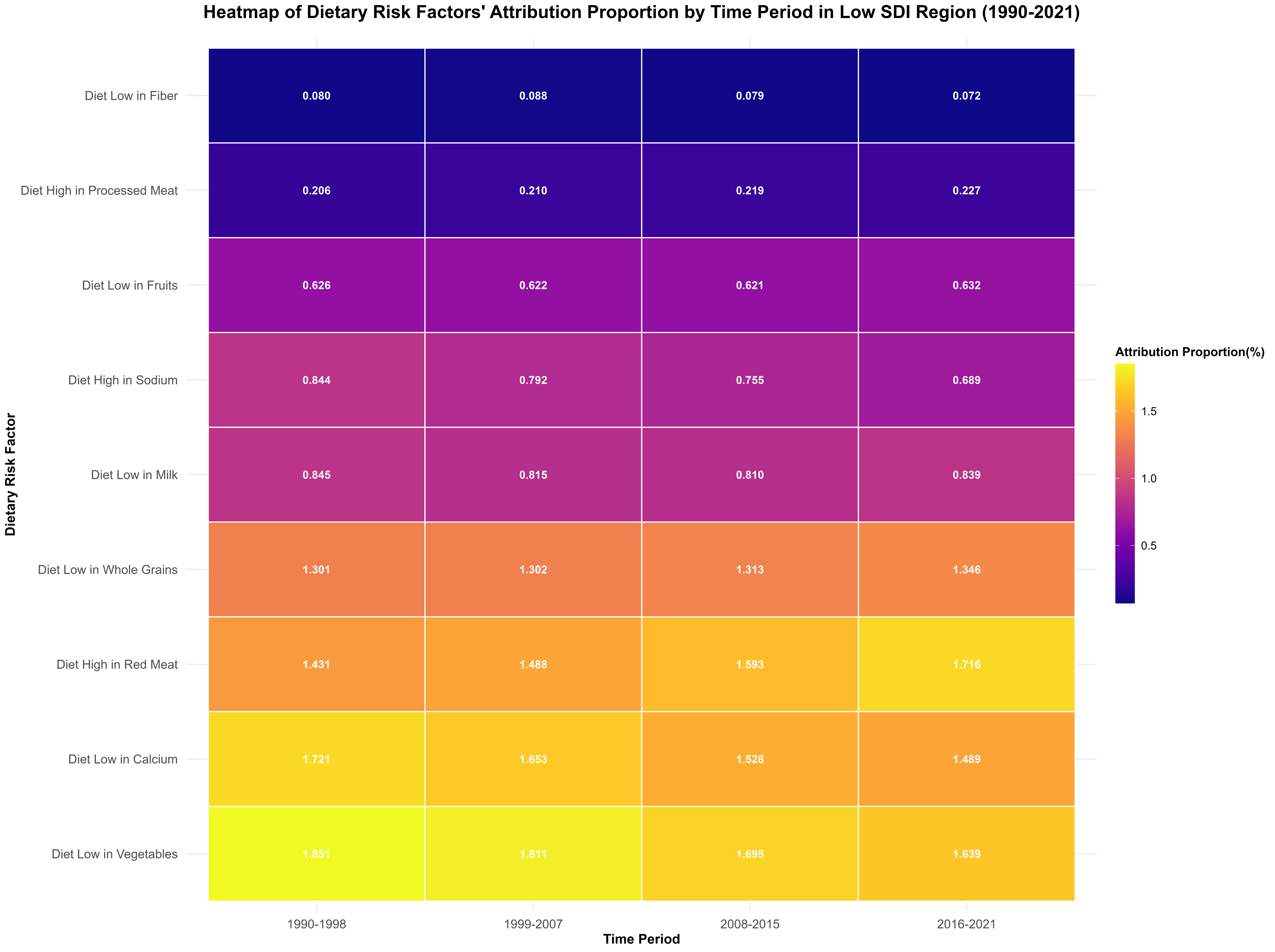

Heatmap of Dietary Risk Factors' Attribution Proportion by Time Period in Low SDI Region (1990-2021)
Dietary Risk Factor
Diet Low in Fiber
Diet High in Processed Meat
Diet Low in Fruits
Diet High in Sodium
Diet Low in Milk
Diet Low in Whole Grains
Diet High in Red Meat
Diet Low in Calcium
Diet Low in Vegetables
0.080 0.088 0.079 0.072
0.206 0.210 0.219 0.227
0.626 0.622 0.621 0.632
0.844 0.792 0.755 0.689
0.845 0.815 0.810 0.839
1.301 1.302 1.313 1.346
1.431 1.488 1.593 1.716
1.721 1.653 1.528 1.489
1.851 1.811 1.695 1.639
1990-1998
1999-2007
2008-2015
2016-2021
Time Period
Attribution Proportion(%)
1.5
1.0
0.5

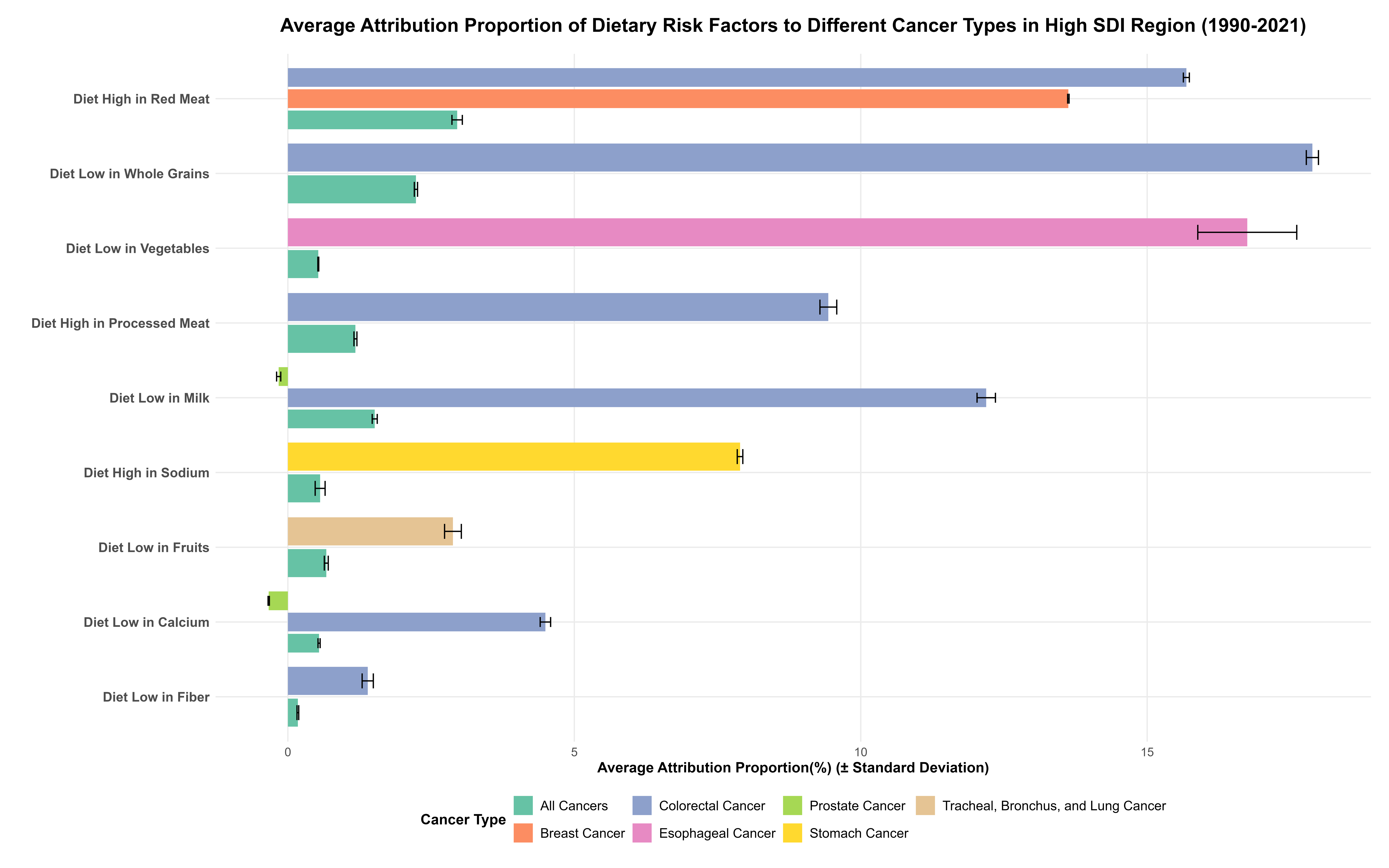

Average Attribution Proportion of Dietary Risk Factors to Different Cancer Types in High SDI Region (1990-2021)
Diet High in Red Meat
Diet Low in Whole Grains
Diet Low in Vegetables
Diet High in Processed Meat
Diet Low in Milk
Diet High in Sodium
Diet Low in Fruits
Diet Low in Calcium
Diet Low in Fiber
0
5
10
15
Average Attribution Proportion(%) (± Standard Deviation)
Cancer Type
All Cancers
Breast Cancer
Colorectal Cancer
Esophageal Cancer
Prostate Cancer
Stomach Cancer
Tracheal, Bronchus, and Lung Cancer

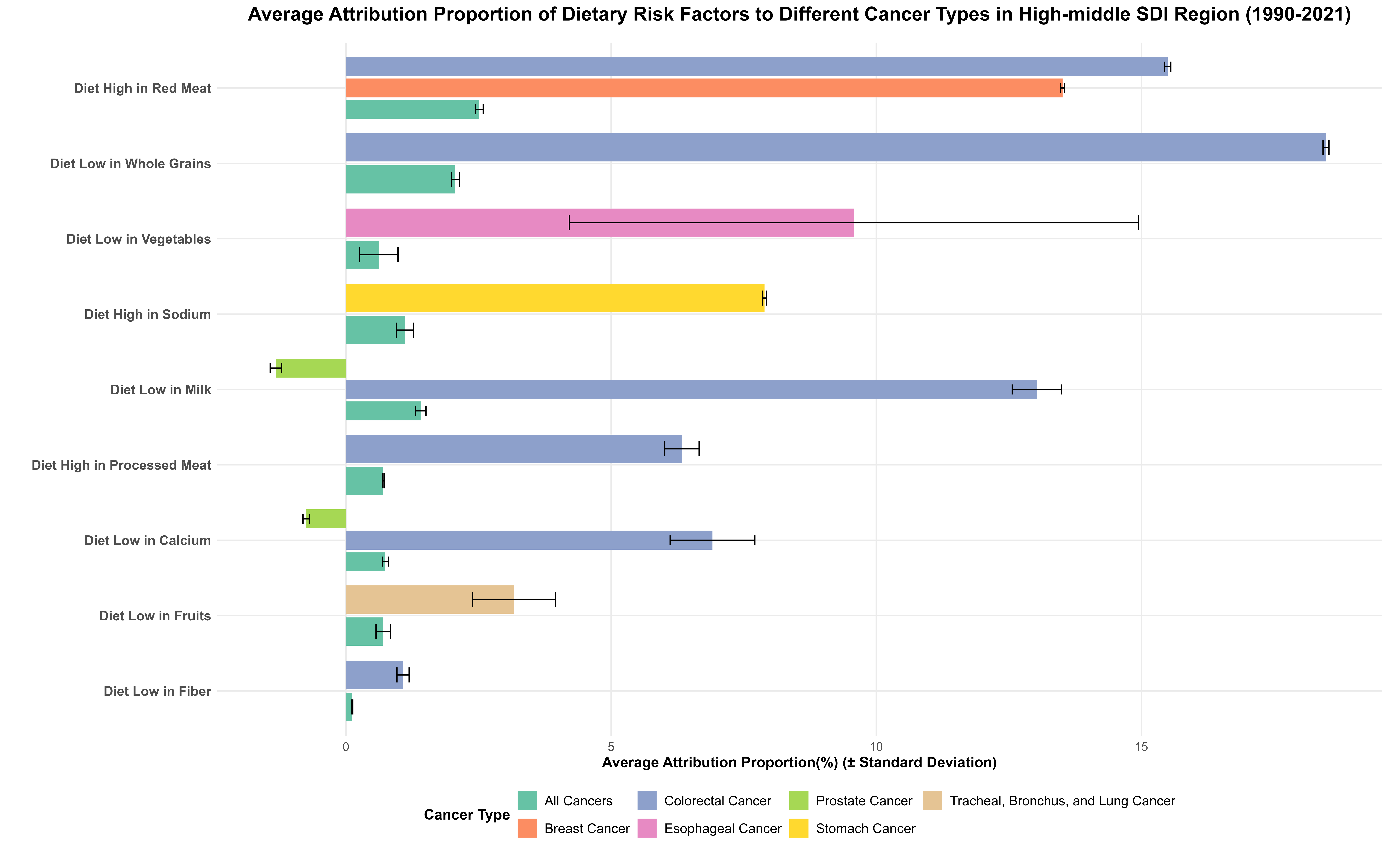

Average Attribution Proportion of Dietary Risk Factors to Different Cancer Types in High-middle SDI Region (1990-2021)
Diet High in Red Meat
Diet Low in Whole Grains
Diet Low in Vegetables
Diet High in Sodium
Diet Low in Milk
Diet High in Processed Meat
Diet Low in Calcium
Diet Low in Fruits
Diet Low in Fiber
0
5
10
15
Average Attribution Proportion(%) (± Standard Deviation)
Cancer Type
All Cancers
Breast Cancer
Colorectal Cancer
Esophageal Cancer
Prostate Cancer
Stomach Cancer
Tracheal, Bronchus, and Lung Cancer

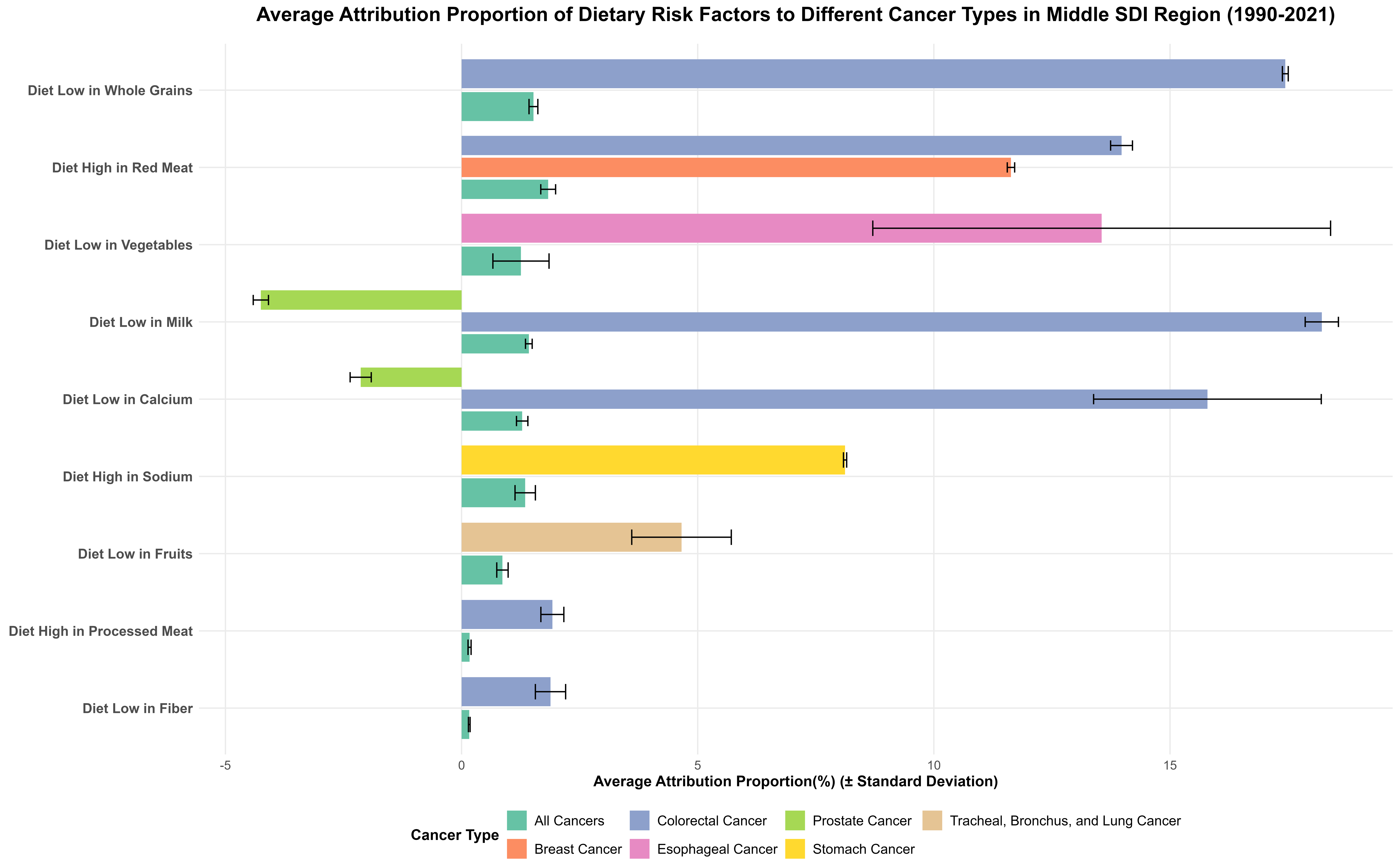
Average Attribution Proportion of Dietary Risk Factors to Different Cancer Types in Middle SDI Region (1990-2021)
Diet Low in Whole Grains
Diet High in Red Meat
Diet Low in Vegetables
Diet Low in Milk
Diet Low in Calcium
Diet High in Sodium
Diet Low in Fruits
Diet High in Processed Meat
Diet Low in Fiber
-5
0
5
10
15
Average Attribution Proportion(%) (± Standard Deviation)
Cancer Type
All Cancers
Breast Cancer
Colorectal Cancer
Esophageal Cancer
Prostate Cancer
Stomach Cancer
Tracheal, Bronchus, and Lung Cancer

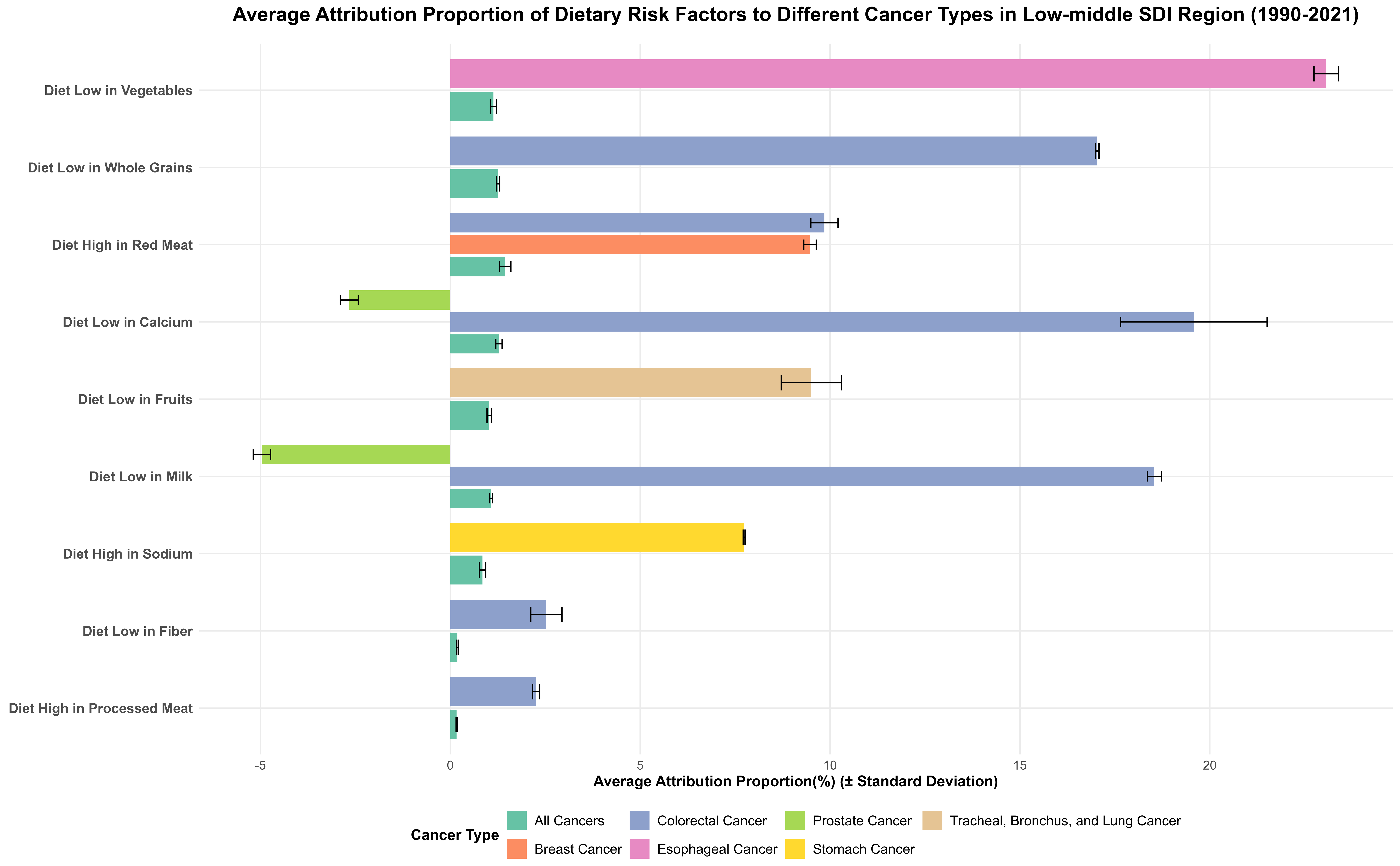
Average Attribution Proportion of Dietary Risk Factors to Different Cancer Types in Low-middle SDI Region (1990-2021)
Diet Low in Vegetables
Diet Low in Whole Grains
Diet High in Red Meat
Diet Low in Calcium
Diet Low in Fruits
Diet Low in Milk
Diet High in Sodium
Diet Low in Fiber
Diet High in Processed Meat
-5
0
5
10
15
20
Average Attribution Proportion(%) (± Standard Deviation)
Cancer Type
All Cancers
Colorectal Cancer
Prostate Cancer
Tracheal, Bronchus, and Lung Cancer
Breast Cancer
Esophageal Cancer
Stomach Cancer

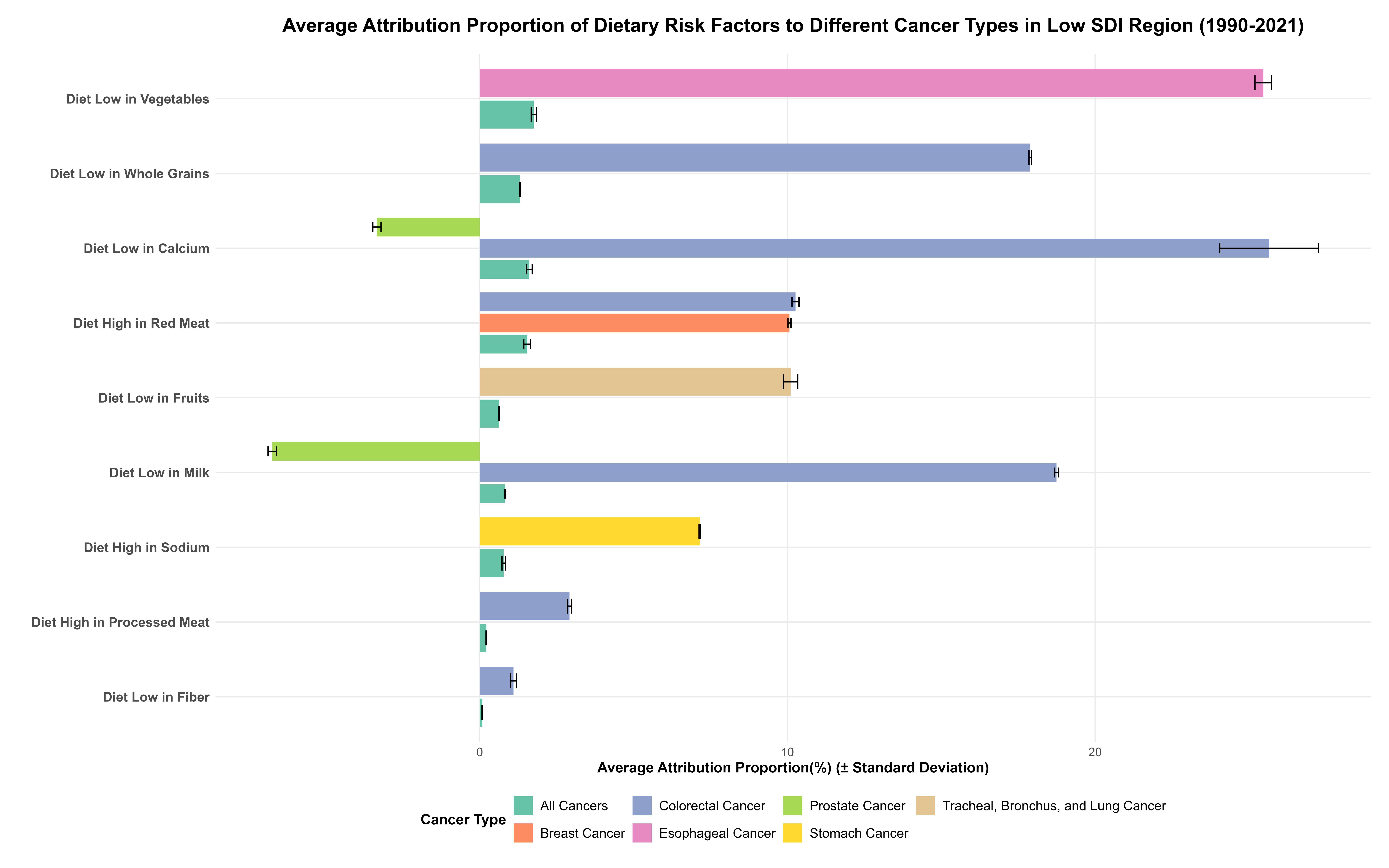

Average Attribution Proportion of Dietary Risk Factors to Different Cancer Types in Low SDI Region (1990-2021)
Diet Low in Vegetables
Diet Low in Whole Grains
Diet Low in Calcium
Diet High in Red Meat
Diet Low in Fruits
Diet Low in Milk
Diet High in Sodium
Diet High in Processed Meat
Diet Low in Fiber
0
10
20
Average Attribution Proportion(%) (± Standard Deviation)
Cancer Type
All Cancers
Breast Cancer
Colorectal Cancer
Esophageal Cancer
Prostate Cancer
Stomach Cancer
Tracheal, Bronchus, and Lung Cancer
