# Supplementary material for: Dietary risk factors and cancer mortality burden from 1990 to 2021: a comparative study of China and global regions with varying sociodemographic development levels based on the Global Burden of Disease database
Source: Front Nutr. 2025 Aug 5;12:1628792. doi: 10.3389/fnut.2025.1628792 (PMC12360945; doi:10.3389/fnut.2025.1628792)
Supplement: Supplementary file 3 [file Image_2.pdf]

Joinpoint Analysis of Dietary Risk Factors' Impact on All Cancer Mortality in High SDI Region (1990-2021)

Joinpoint Analysis of All Dietary Risks Impact on All Cancer Mortality in High SDI

Joinpoint Years: 1995, 1997, 2003, 2005, 2012

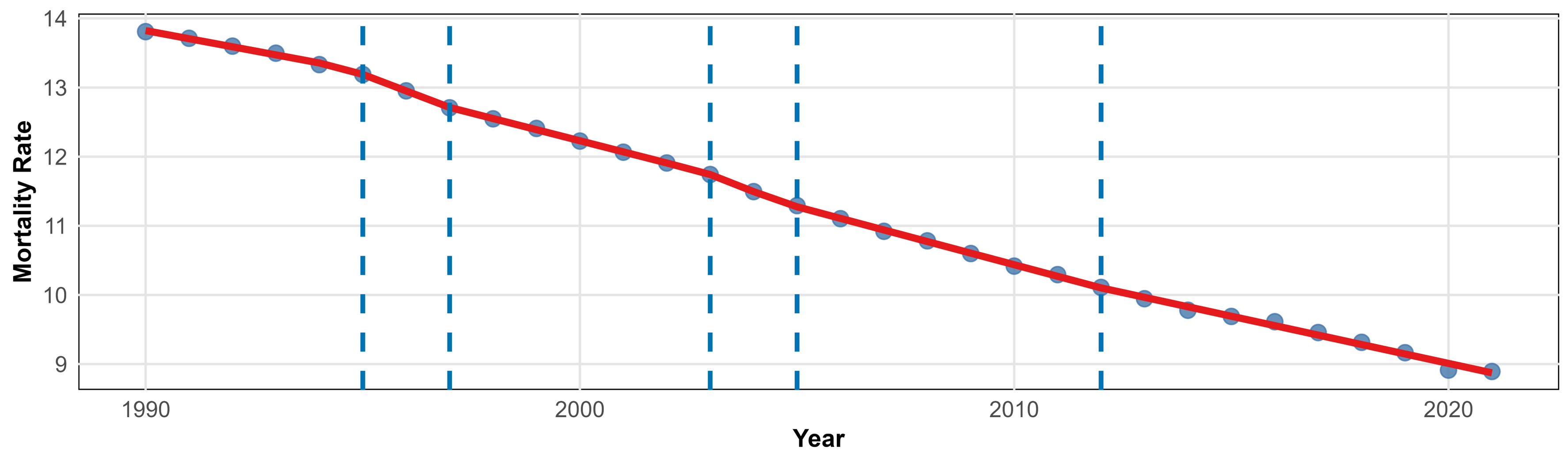

Segment	Period	CI
Segment 1	1990 - 1995	-11.04% (-12.87%, -9.18%)
Segment 2	1995 - 1997	-21.27% (-28.39%, -13.43%)
Segment 3	1997 - 2003	-14.85% (-22.48%, -6.46%)
Segment 4	2003 - 2005	-21.92% (-28.92%, -14.23%)
Segment 5	2005 - 2012	-15.44% (-22.97%, -7.16%)
Segment 6	2012 - 2021	-12.77% (-14.01%, -11.51%)
**AAPC**	**1990 - 2021**	**-14.63% (-17.12%, -12.07%)**

Joinpoint Analysis of Diet High in Red Meat Impact on All Cancer Mortality in High SDI

Joinpoint Years: 1993, 2003, 2006, 2014, 2016

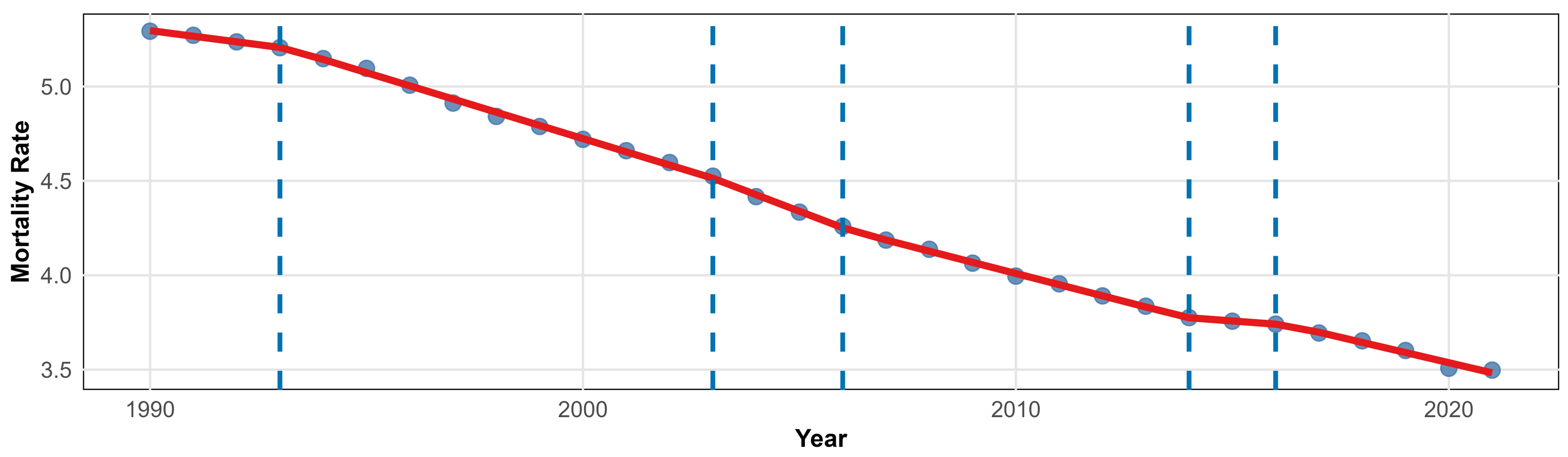

Segment	Period	CI
Segment 1	1990 - 1993	-2.93% (-4.08%, -1.76%)
Segment 2	1993 - 2003	-6.75% (-7.90%, -5.59%)
Segment 3	2003 - 2006	-8.37% (-9.50%, -7.23%)
Segment 4	2006 - 2014	-5.74% (-6.95%, -4.51%)
Segment 5	2014 - 2016	-1.68% (-3.58%, 0.25%)
Segment 6	2016 - 2021	-5.23% (-7.17%, -3.26%)
**AAPC**	**1990 - 2021**	**-5.71% (-6.32%, -5.09%)**

Joinpoint Analysis of Diet Low in Whole Grains Impact on All Cancer Mortality in High SDI

Joinpoint Years: 1993, 2003, 2006, 2014, 2016

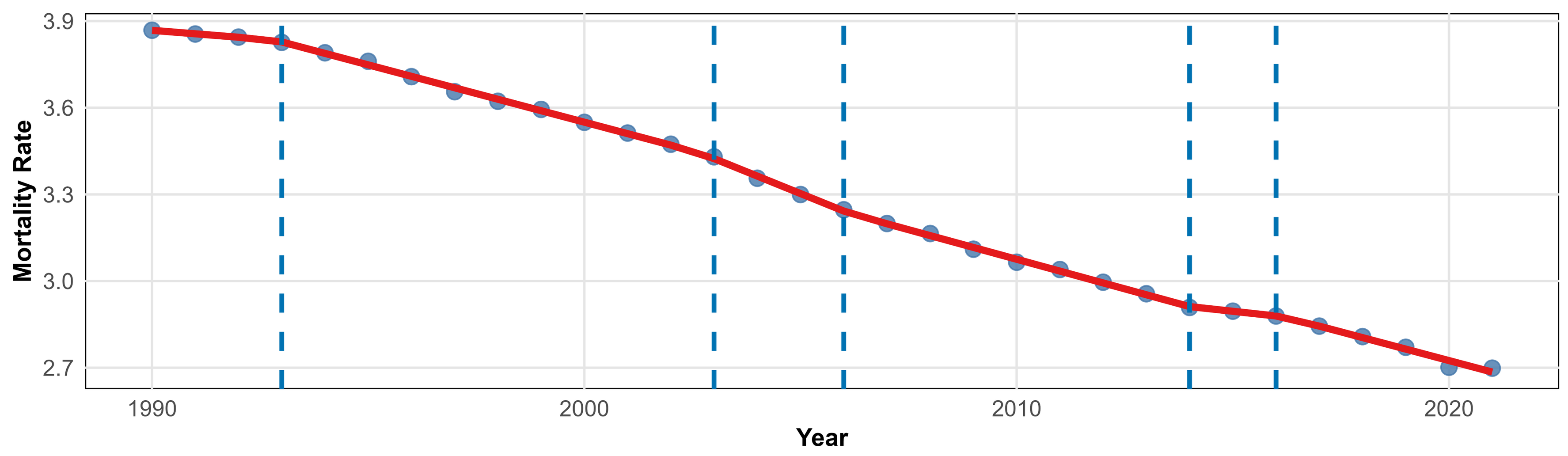

Segment	Period	CI
Segment 1	1990 - 1993	-1.18% (-2.39%, 0.04%)
Segment 2	1993 - 2003	-3.89% (-5.08%, -2.69%)
Segment 3	2003 - 2006	-5.87% (-6.62%, -5.12%)
Segment 4	2006 - 2014	-4.01% (-4.80%, -3.22%)
Segment 5	2014 - 2016	-1.61% (-4.01%, 0.85%)
Segment 6	2016 - 2021	-3.90% (-6.29%, -1.45%)
**AAPC**	**1990 - 2021**	**-3.71% (-4.33%, -3.08%)**

Joinpoint Analysis of Diet Low in Milk Impact on All Cancer Mortality in High SDI

Joinpoint Years: 1993, 2007, 2018

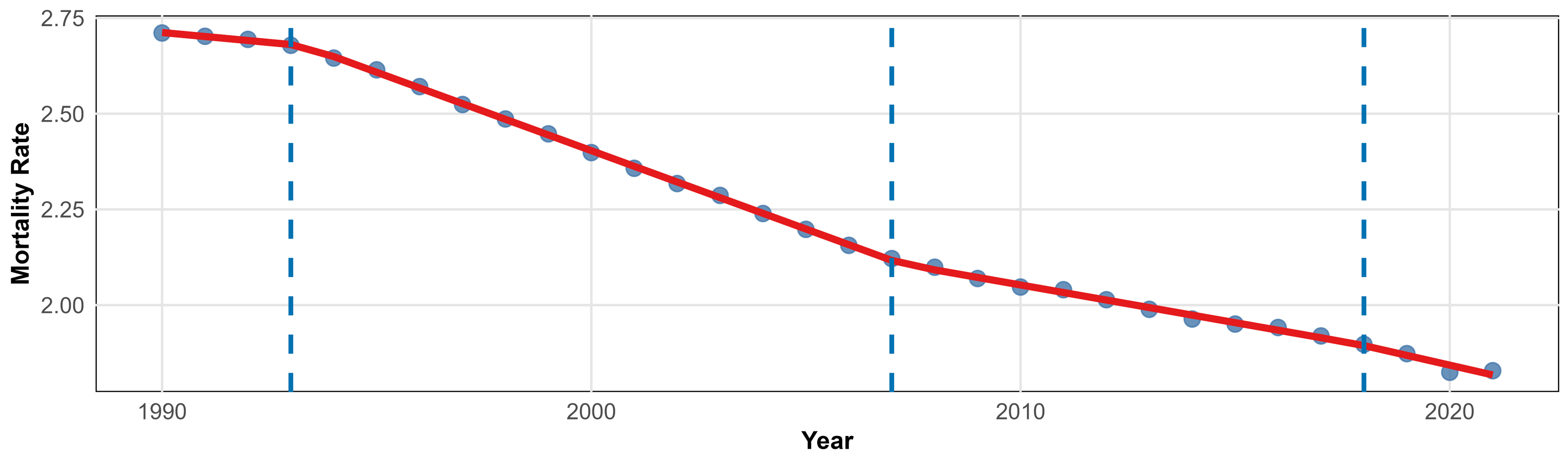

Segment	Period	CI
Segment 1	1990 - 1993	-1.04% (-1.62%, -0.46%)
Segment 2	1993 - 2007	-4.01% (-4.58%, -3.44%)
Segment 3	2007 - 2018	-1.95% (-2.12%, -1.78%)
Segment 4	2018 - 2021	-2.52% (-3.11%, -1.93%)
**AAPC**	**1990 - 2021**	**-2.85% (-3.13%, -2.57%)**

Joinpoint Analysis of Diet High in Sodium Impact on All Cancer Mortality in High SDI

Joinpoint Years: 1992, 2002, 2007, 2015, 2019

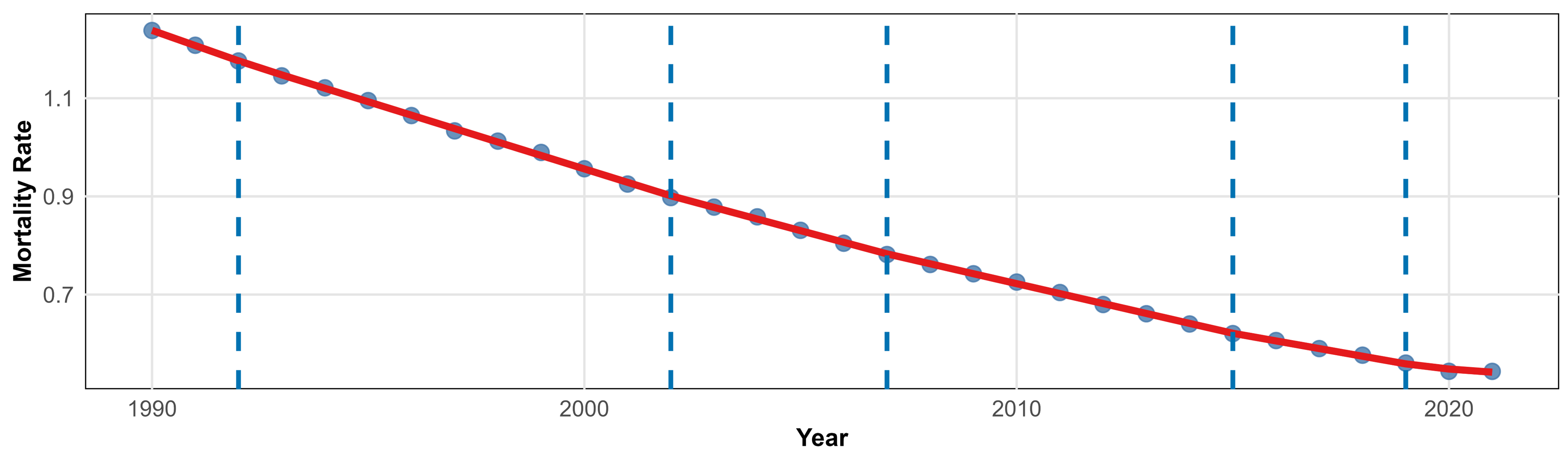

Segment	Period	CI
Segment 1	1990 - 1992	-3.05% (-3.46%, -2.65%)
Segment 2	1992 - 2002	-2.70% (-3.11%, -2.29%)
Segment 3	2002 - 2007	-2.34% (-2.54%, -2.14%)
Segment 4	2007 - 2015	-2.00% (-2.21%, -1.80%)
Segment 5	2015 - 2019	-1.55% (-1.75%, -1.34%)
Segment 6	2019 - 2021	-0.64% (-1.48%, 0.22%)
**AAPC**	**1990 - 2021**	**-2.20% (-2.36%, -2.04%)**

Joinpoint Analysis of Diet High in Processed Meat Impact on All Cancer Mortality in High SDI

Joinpoint Years: 1993, 2002, 2014, 2016

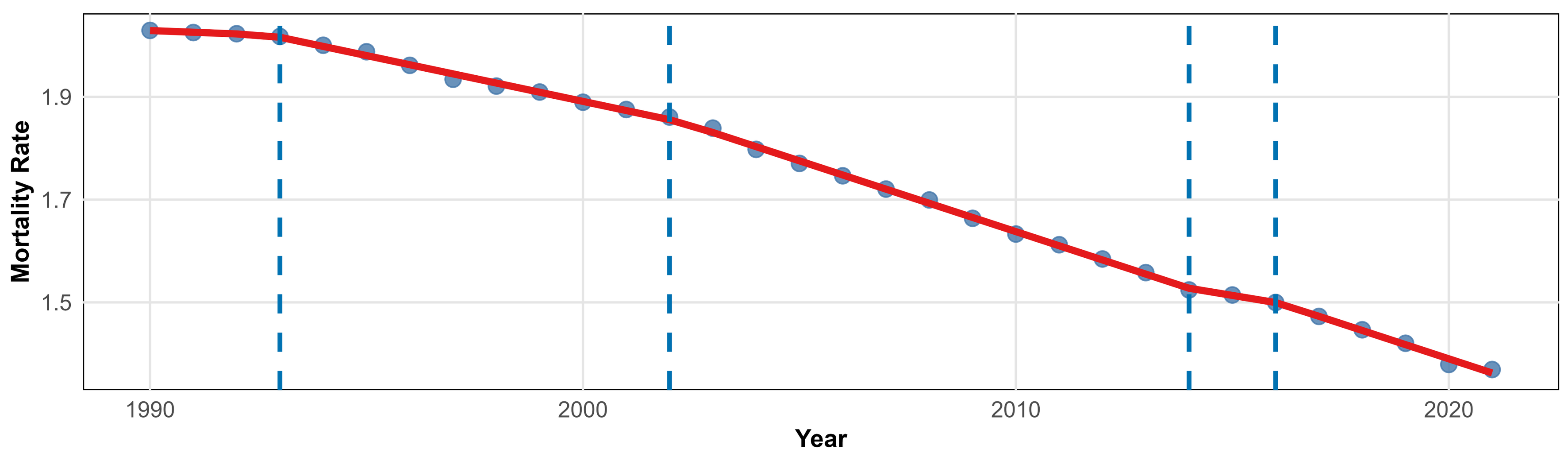

Segment	Period	CI
Segment 1	1990 - 1993	-0.33% (-1.07%, 0.42%)
Segment 2	1993 - 2002	-1.77% (-2.51%, -1.02%)
Segment 3	2002 - 2014	-2.72% (-2.86%, -2.58%)
Segment 4	2014 - 2016	-1.38% (-2.85%, 0.11%)
Segment 5	2016 - 2021	-2.72% (-4.20%, -1.22%)
**AAPC**	**1990 - 2021**	**-2.12% (-2.47%, -1.77%)**

Joinpoint Analysis of Diet Low in Fruits Impact on All Cancer Mortality in High SDI

Joinpoint Years: 1995, 1998, 2009, 2014, 2019

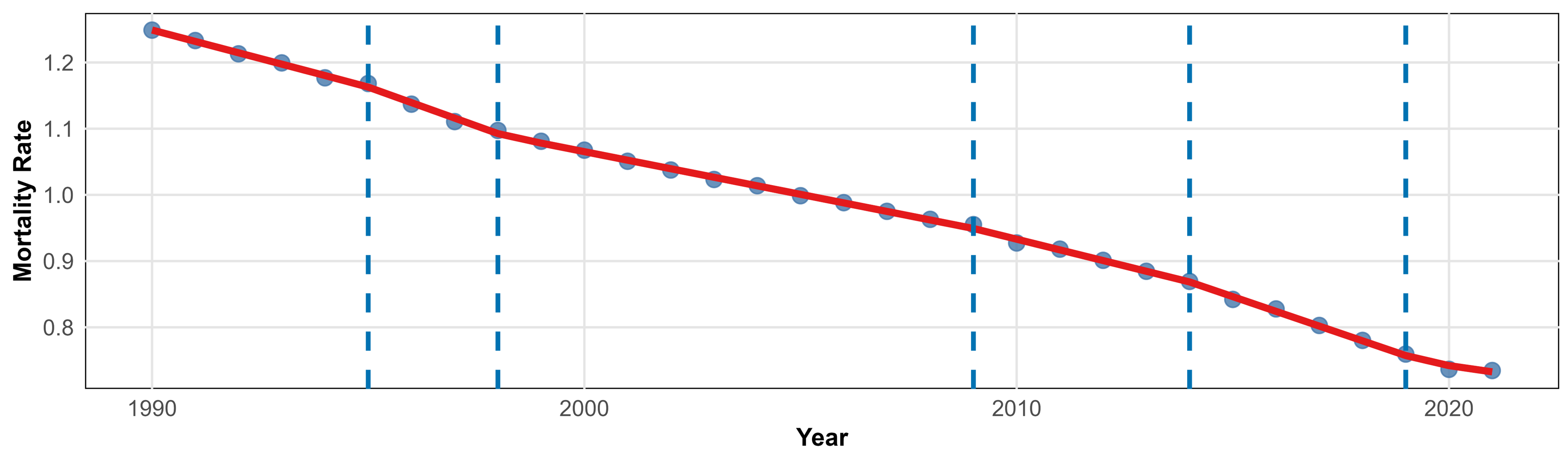

Segment	Period	CI
Segment 1	1990 - 1995	-1.70% (-1.93%, -1.47%)
Segment 2	1995 - 1998	-2.31% (-2.71%, -1.92%)
Segment 3	1998 - 2009	-1.28% (-1.62%, -0.95%)
Segment 4	2009 - 2014	-1.60% (-1.79%, -1.41%)
Segment 5	2014 - 2019	-2.20% (-2.49%, -1.91%)
Segment 6	2019 - 2021	-0.94% (-1.99%, 0.13%)
**AAPC**	**1990 - 2021**	**-1.63% (-1.78%, -1.47%)**

Joinpoint Analysis of Diet Low in Calcium Impact on All Cancer Mortality in High SDI

Joinpoint Years: 1993, 1999, 2007, 2011, 2016

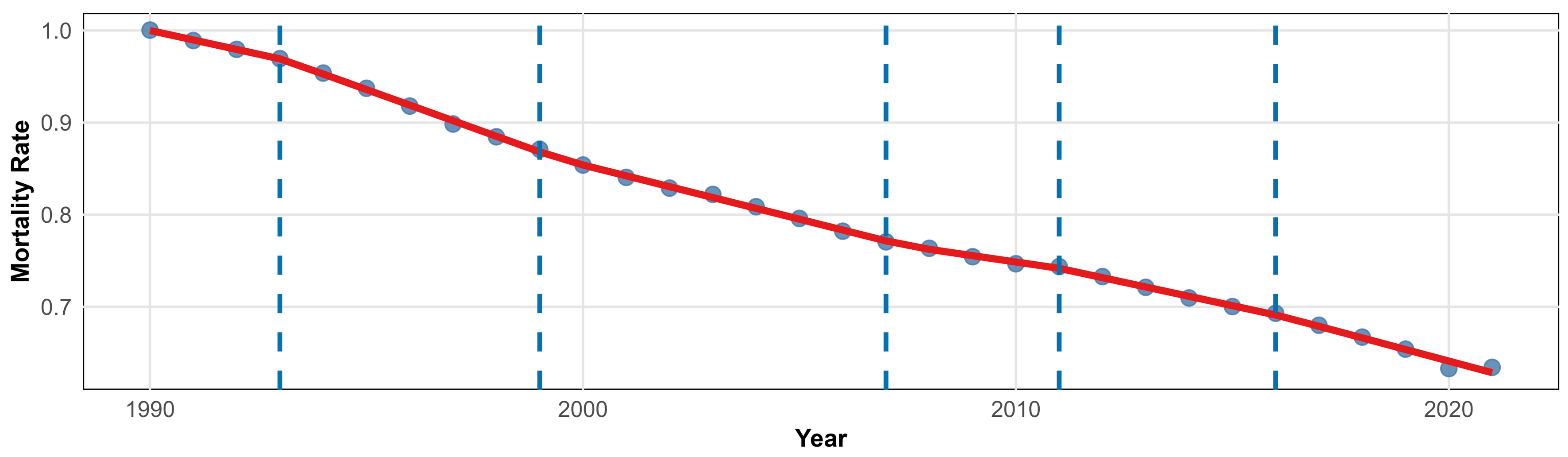

Segment	Period	CI
Segment 1	1990 - 1993	-1.02% (-1.27%, -0.77%)
Segment 2	1993 - 1999	-1.68% (-1.96%, -1.39%)
Segment 3	1999 - 2007	-1.17% (-1.33%, -1.01%)
Segment 4	2007 - 2011	-0.68% (-0.95%, -0.42%)
Segment 5	2011 - 2016	-1.01% (-1.32%, -0.70%)
Segment 6	2016 - 2021	-1.25% (-1.50%, -1.00%)
**AAPC**	**1990 - 2021**	**-1.18% (-1.28%, -1.07%)**

Joinpoint Analysis of Diet Low in Vegetables Impact on All Cancer Mortality in High SDI

Joinpoint Years: 1995, 1997, 2007, 2014, 2016

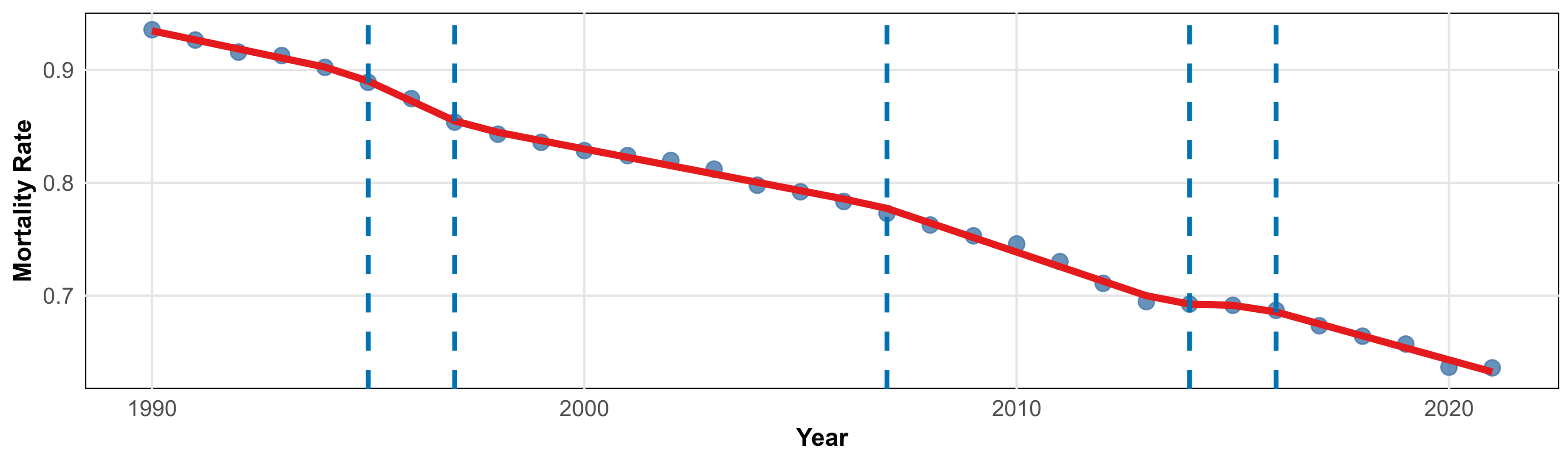

Segment	Period	CI
Segment 1	1990 - 1995	-0.80% (-1.03%, -0.57%)
Segment 2	1995 - 1997	-1.75% (-2.30%, -1.19%)
Segment 3	1997 - 2007	-0.74% (-1.26%, -0.21%)
Segment 4	2007 - 2014	-1.28% (-1.45%, -1.12%)
Segment 5	2014 - 2016	-0.11% (-1.14%, 0.94%)
Segment 6	2016 - 2021	-1.06% (-2.09%, -0.01%)
**AAPC**	**1990 - 2021**	**-0.95% (-1.20%, -0.69%)**

Joinpoint Analysis of Diet Low in Fiber Impact on All Cancer Mortality in High SDI

Joinpoint Years: 1994, 2000, 2007, 2012

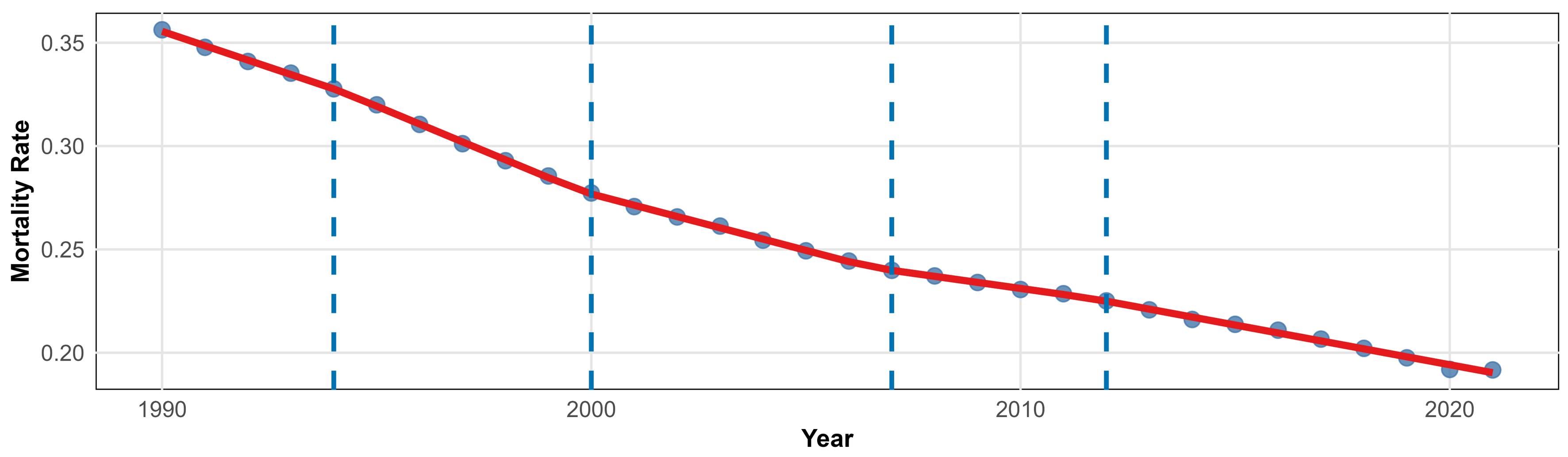

Segment	Period	CI
Segment 1	1990 - 1994	-0.69% (-0.75%, -0.64%)
Segment 2	1994 - 2000	-0.86% (-0.94%, -0.78%)
Segment 3	2000 - 2007	-0.54% (-0.61%, -0.48%)
Segment 4	2007 - 2012	-0.29% (-0.36%, -0.23%)
Segment 5	2012 - 2021	-0.38% (-0.44%, -0.33%)
**AAPC**	**1990 - 2021**	**-0.54% (-0.57%, -0.51%)**

Joinpoint Analysis of Dietary Risk Factors' Impact on All Cancer Mortality in High-middle SDI Region (1990-2021)

Joinpoint Analysis of All Dietary Risks Impact on All Cancer Mortality in High-middle SDI

Joinpoint Years: 1994, 1997, 2004, 2007, 2014

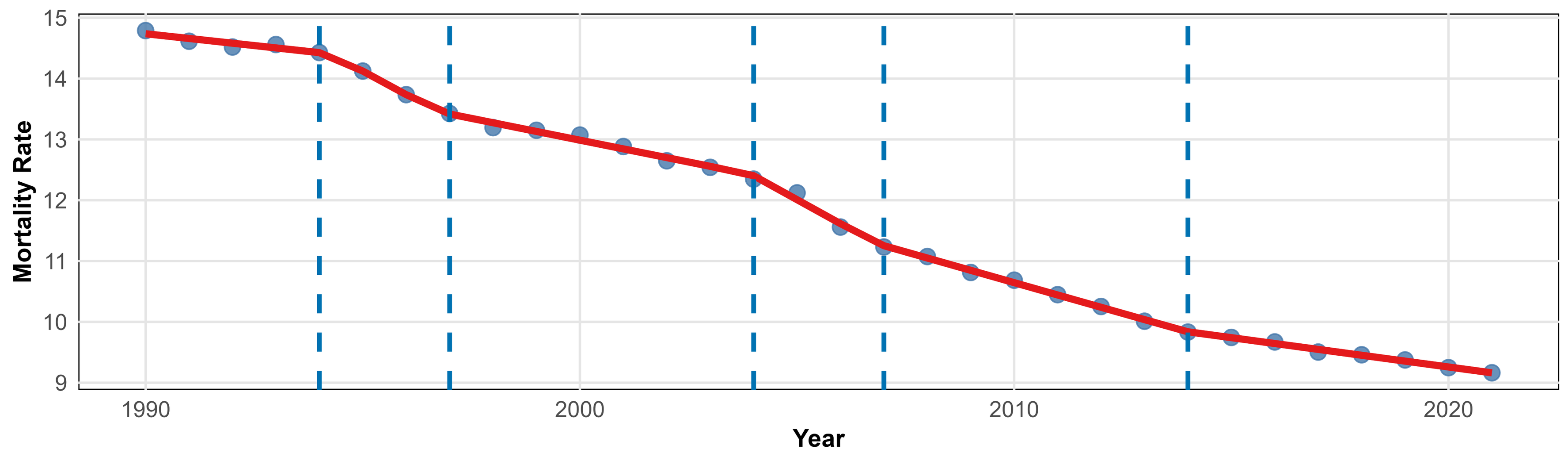

Segment	Period	CI
Segment 1	1990 - 1994	-7.46% (-10.48%, -4.35%)
Segment 2	1994 - 1997	-32.14% (-41.69%, -21.02%)
Segment 3	1997 - 2004	-13.36% (-25.38%, 0.60%)
Segment 4	2004 - 2007	-32.59% (-37.56%, -27.22%)
Segment 5	2007 - 2014	-18.33% (-24.36%, -11.82%)
Segment 6	2014 - 2021	-9.19% (-11.48%, -6.84%)
**AAPC**	**1990 - 2021**	**-16.46% (-19.89%, -12.88%)**

Joinpoint Analysis of Diet Low in Vegetables Impact on All Cancer Mortality in High-middle SDI

Joinpoint Years: 1994, 1998, 2002, 2008, 2014

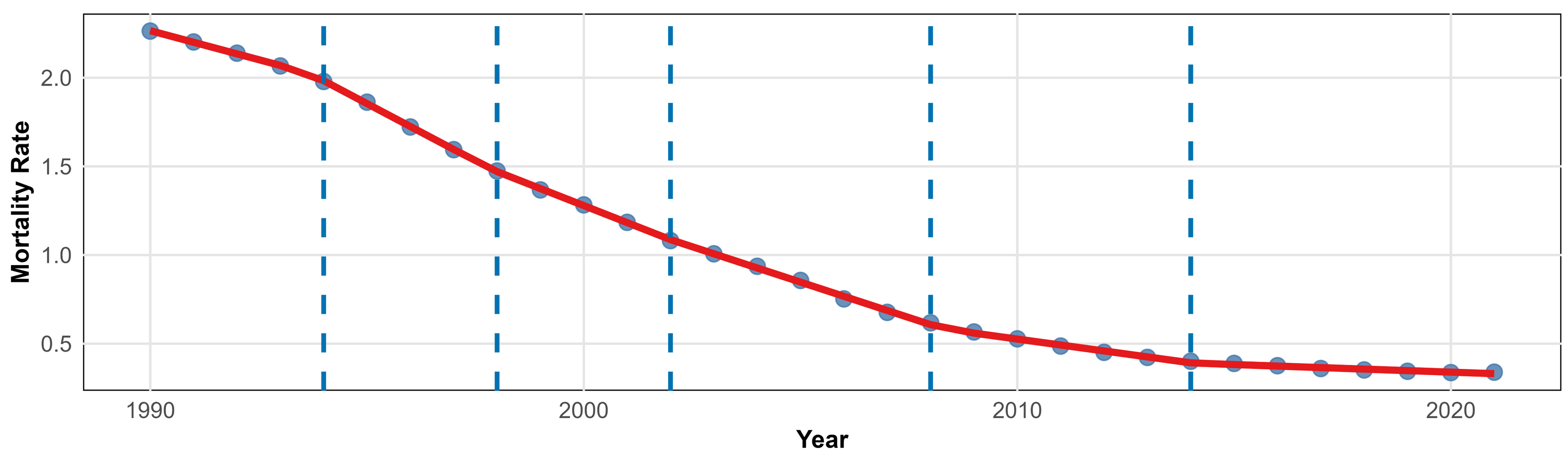

Segment	Period	CI
Segment 1	1990 - 1994	-6.33% (-6.98%, -5.67%)
Segment 2	1994 - 1998	-12.13% (-12.99%, -11.26%)
Segment 3	1998 - 2002	-9.15% (-9.92%, -8.37%)
Segment 4	2002 - 2008	-7.68% (-8.25%, -7.11%)
Segment 5	2008 - 2014	-3.30% (-3.80%, -2.78%)
Segment 6	2014 - 2021	-0.85% (-1.32%, -0.38%)
**AAPC**	**1990 - 2021**	**-5.88% (-6.13%, -5.63%)**

Joinpoint Analysis of Diet High in Sodium Impact on All Cancer Mortality in High-middle SDI

Joinpoint Years: 1994, 1997, 2005, 2006, 2015

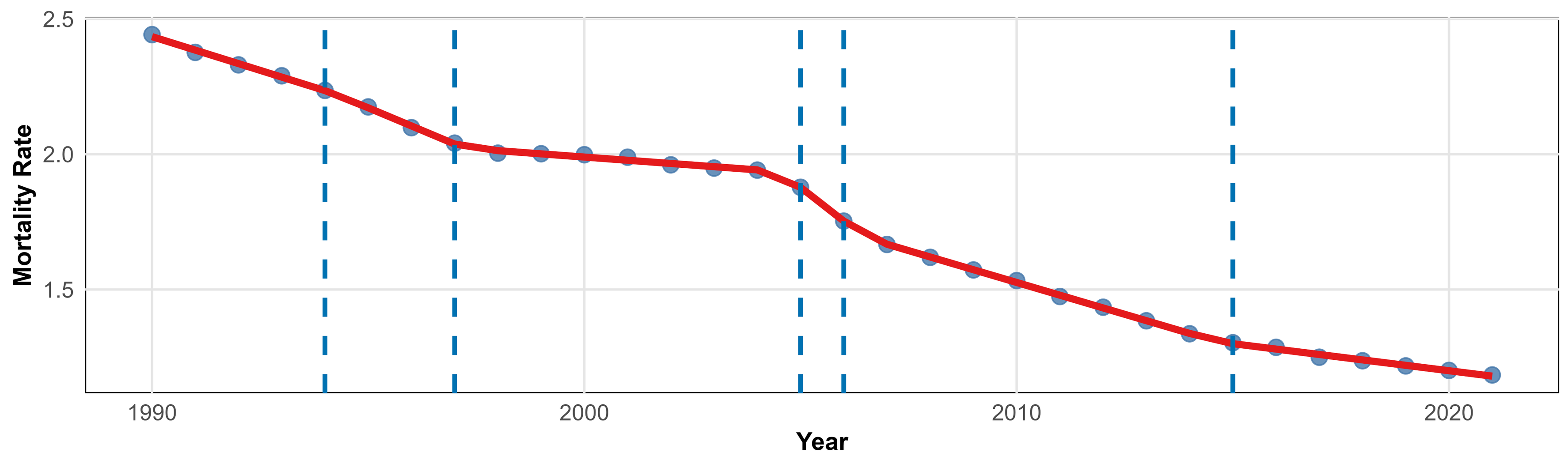

Segment	Period	CI
Segment 1	1990 - 1994	-4.87% (-5.25%, -4.48%)
Segment 2	1994 - 1997	-6.48% (-7.41%, -5.55%)
Segment 3	1997 - 2005	-1.18% (-2.10%, -0.25%)
Segment 4	2005 - 2006	-11.75% (-13.34%, -10.12%)
Segment 5	2006 - 2015	-4.60% (-6.32%, -2.85%)
Segment 6	2015 - 2021	-1.98% (-2.29%, -1.68%)
**AAPC**	**1990 - 2021**	**-3.66% (-4.23%, -3.08%)**

Joinpoint Analysis of Diet Low in Fruits Impact on All Cancer Mortality in High-middle SDI

Joinpoint Years: 1993, 2004, 2007, 2014

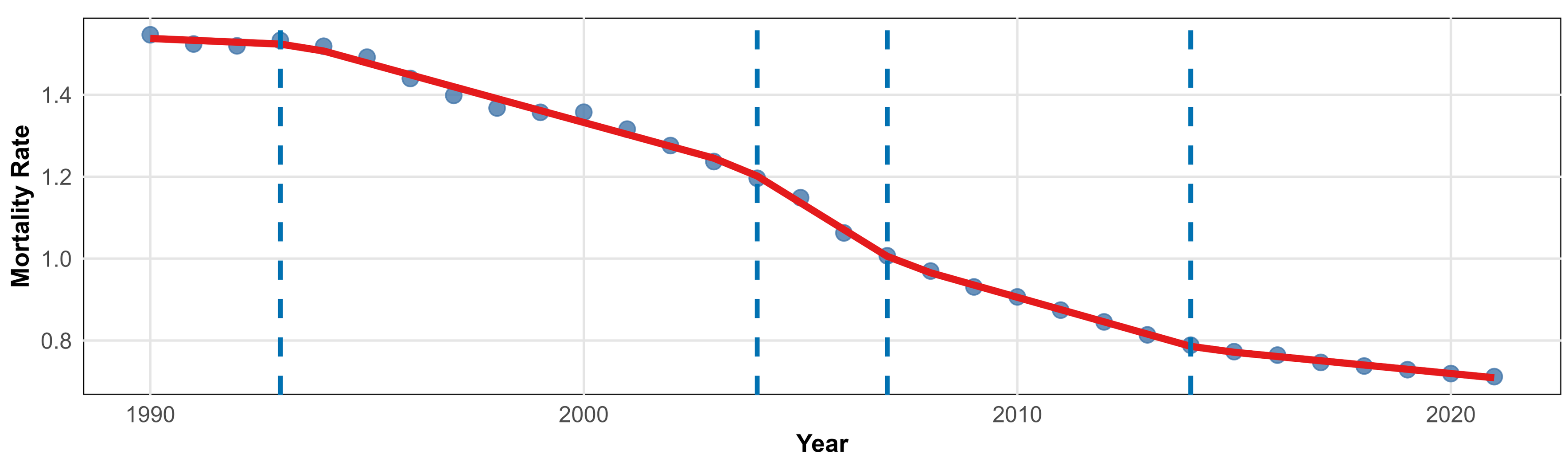

Segment	Period	CI
Segment 1	1990 - 1993	-0.46% (-1.46%, 0.56%)
Segment 2	1993 - 2004	-2.87% (-3.87%, -1.85%)
Segment 3	2004 - 2007	-6.33% (-7.30%, -5.35%)
Segment 4	2007 - 2014	-2.95% (-4.01%, -1.88%)
Segment 5	2014 - 2021	-1.03% (-1.63%, -0.43%)
**AAPC**	**1990 - 2021**	**-2.57% (-3.05%, -2.10%)**

Joinpoint Analysis of Diet High in Red Meat Impact on All Cancer Mortality in High-middle SDI

Joinpoint Years: 1994, 1997, 2002, 2014

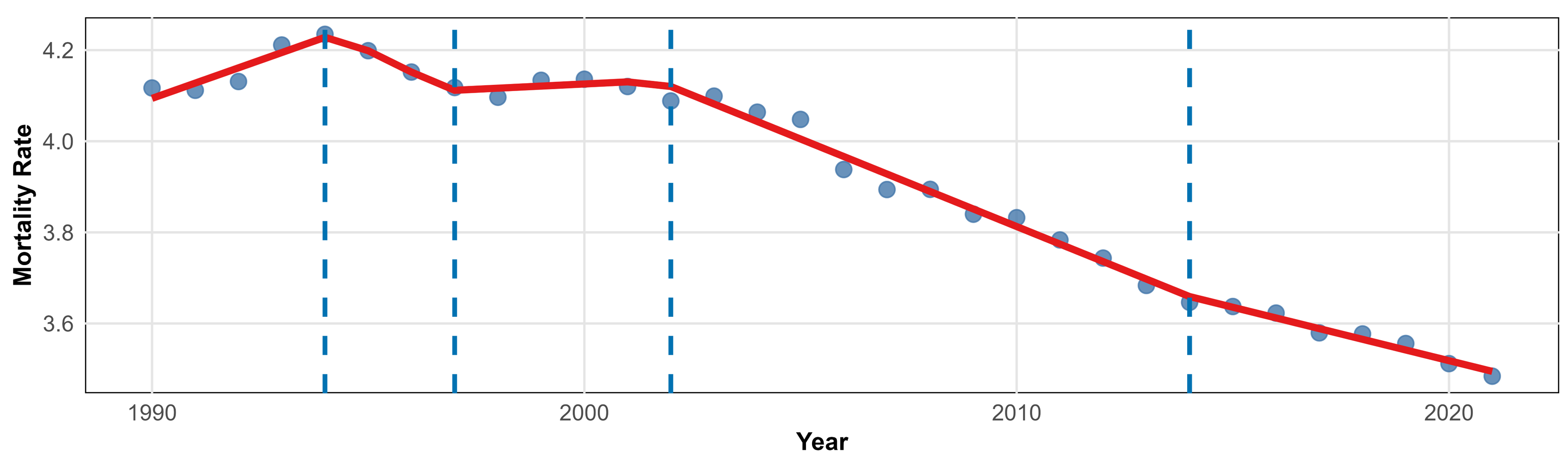

Segment	Period	CI
Segment 1	1990 - 1994	3.41% (2.04%, 4.79%)
Segment 2	1994 - 1997	-4.58% (-10.20%, 1.40%)
Segment 3	1997 - 2002	0.46% (-5.46%, 6.76%)
Segment 4	2002 - 2014	-3.77% (-5.08%, -2.44%)
Segment 5	2014 - 2021	-2.32% (-3.03%, -1.60%)
**AAPC**	**1990 - 2021**	**-1.91% (-3.16%, -0.65%)**

Joinpoint Analysis of Diet Low in Calcium Impact on All Cancer Mortality in High-middle SDI

Joinpoint Years: 1994, 1997, 2005, 2006, 2014

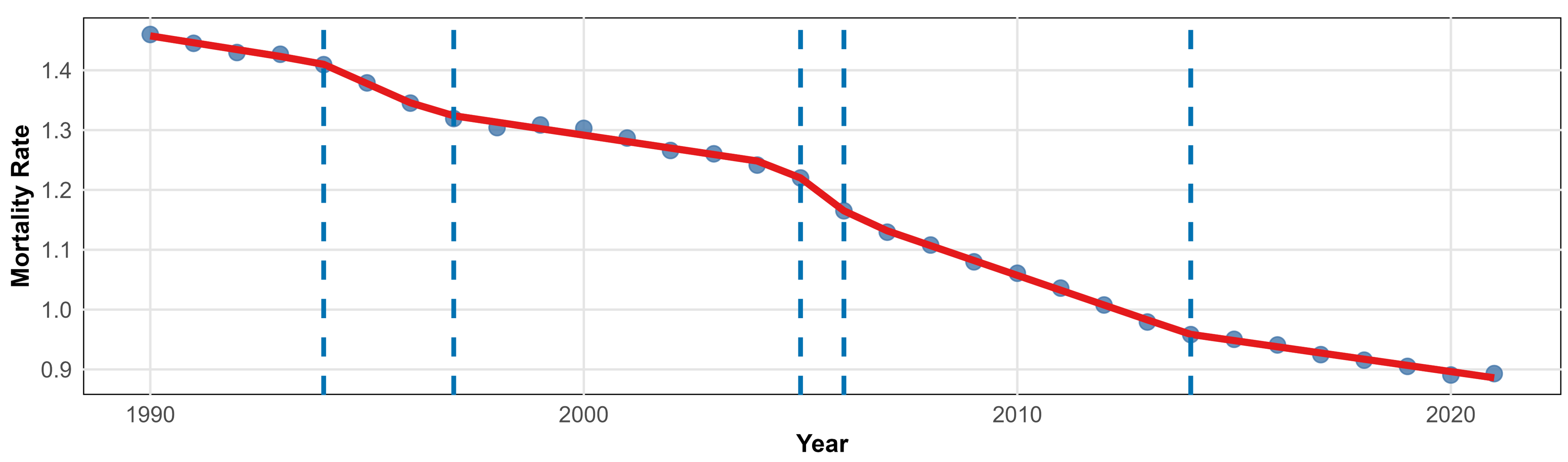

Segment	Period	CI
Segment 1	1990 - 1994	-1.14% (-1.60%, -0.67%)
Segment 2	1994 - 1997	-3.16% (-4.01%, -2.31%)
Segment 3	1997 - 2005	-1.07% (-1.82%, -0.32%)
Segment 4	2005 - 2006	-5.34% (-6.75%, -3.92%)
Segment 5	2006 - 2014	-2.45% (-3.90%, -0.98%)
Segment 6	2014 - 2021	-1.03% (-1.28%, -0.78%)
**AAPC**	**1990 - 2021**	**-1.77% (-2.21%, -1.32%)**

Joinpoint Analysis of Diet Low in Whole Grains Impact on All Cancer Mortality in High-middle SDI

Joinpoint Years: 1992, 1994, 1997, 2004, 2007

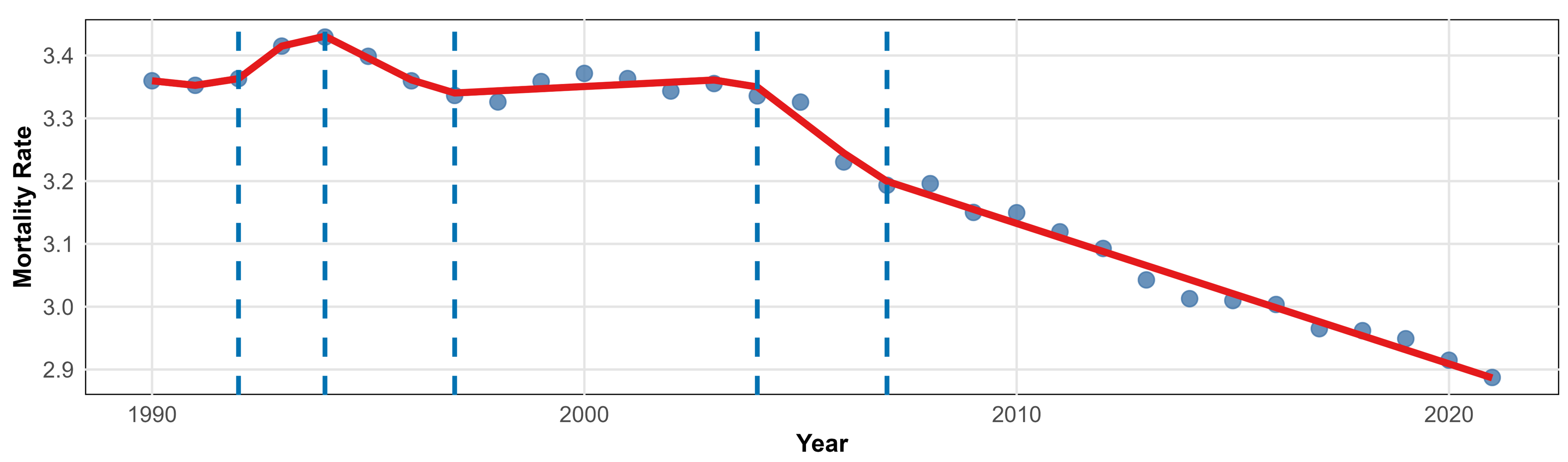

Segment	Period	CI
Segment 1	1990 - 1992	-0.72% (-5.13%, 3.89%)
Segment 2	1992 - 1994	5.31% (-1.24%, 12.30%)
Segment 3	1994 - 1997	-3.42% (-8.20%, 1.61%)
Segment 4	1997 - 2004	0.34% (-1.99%, 2.73%)
Segment 5	2004 - 2007	-5.12% (-7.33%, -2.87%)
Segment 6	2007 - 2021	-2.21% (-4.41%, 0.04%)
**AAPC**	**1990 - 2021**	**-1.45% (-2.80%, -0.09%)**

Joinpoint Analysis of Diet High in Processed Meat Impact on All Cancer Mortality in High-middle SDI

Joinpoint Years: 1994, 1998, 1999, 2003, 2013

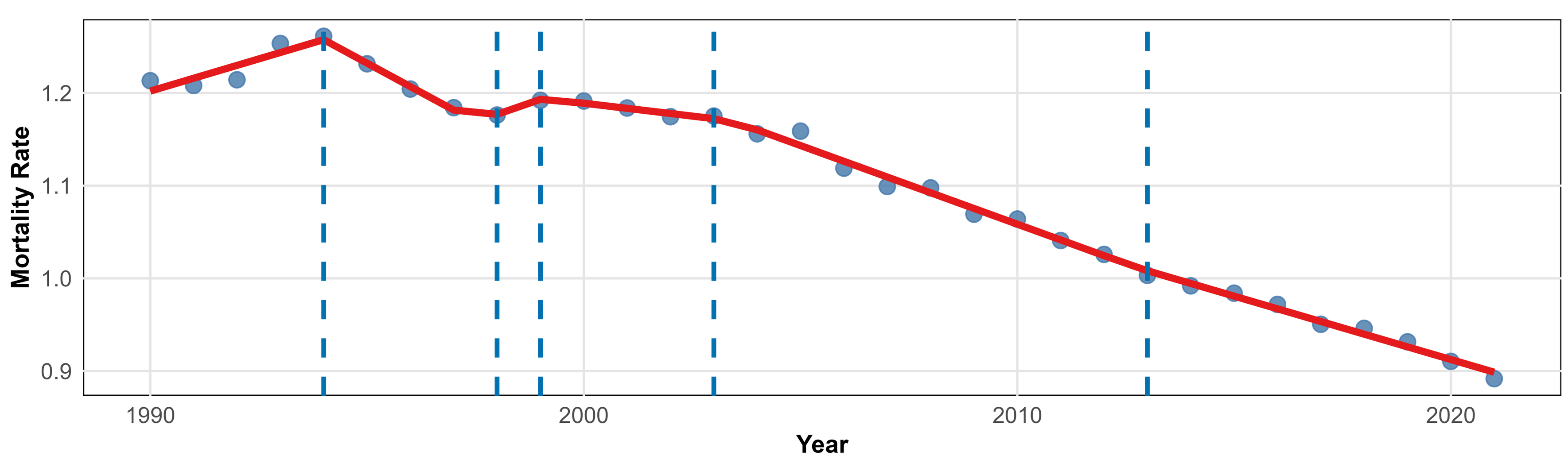

Segment	Period	CI
Segment 1	1990 - 1994	1.40% (0.69%, 2.11%)
Segment 2	1994 - 1998	-2.50% (-3.47%, -1.53%)
Segment 3	1998 - 1999	1.64% (-0.71%, 4.04%)
Segment 4	1999 - 2003	-0.55% (-2.85%, 1.79%)
Segment 5	2003 - 2013	-1.68% (-2.40%, -0.96%)
Segment 6	2013 - 2021	-1.36% (-1.64%, -1.08%)
**AAPC**	**1990 - 2021**	**-1.05% (-1.48%, -0.63%)**

Joinpoint Analysis of Diet Low in Milk Impact on All Cancer Mortality in High-middle SDI

Joinpoint Years: 1997, 2005, 2006, 2014, 2019

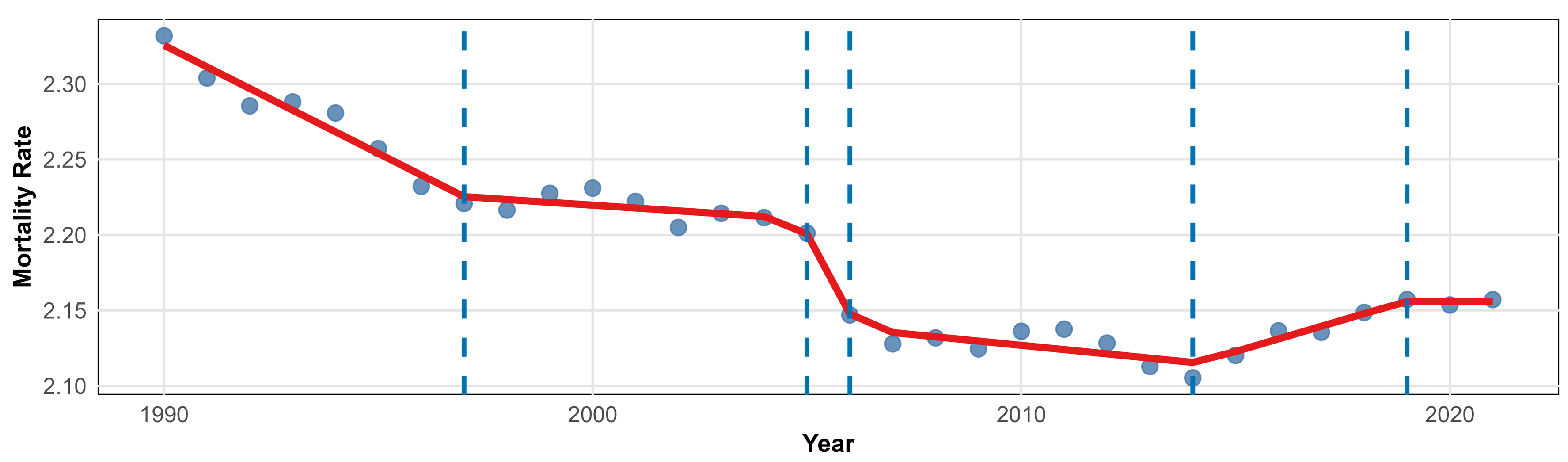

Segment	Period	CI
Segment 1	1990 - 1997	-1.42% (-1.73%, -1.11%)
Segment 2	1997 - 2005	-0.19% (-0.59%, 0.22%)
Segment 3	2005 - 2006	-5.17% (-7.39%, -2.89%)
Segment 4	2006 - 2014	-0.28% (-2.62%, 2.12%)
Segment 5	2014 - 2019	0.84% (0.05%, 1.64%)
Segment 6	2019 - 2021	0.00% (-1.38%, 1.41%)
**AAPC**	**1990 - 2021**	**-0.47% (-1.12%, 0.18%)**

Joinpoint Analysis of Diet Low in Fiber Impact on All Cancer Mortality in High-middle SDI

Joinpoint Years: 1996, 2000, 2004, 2008, 2013

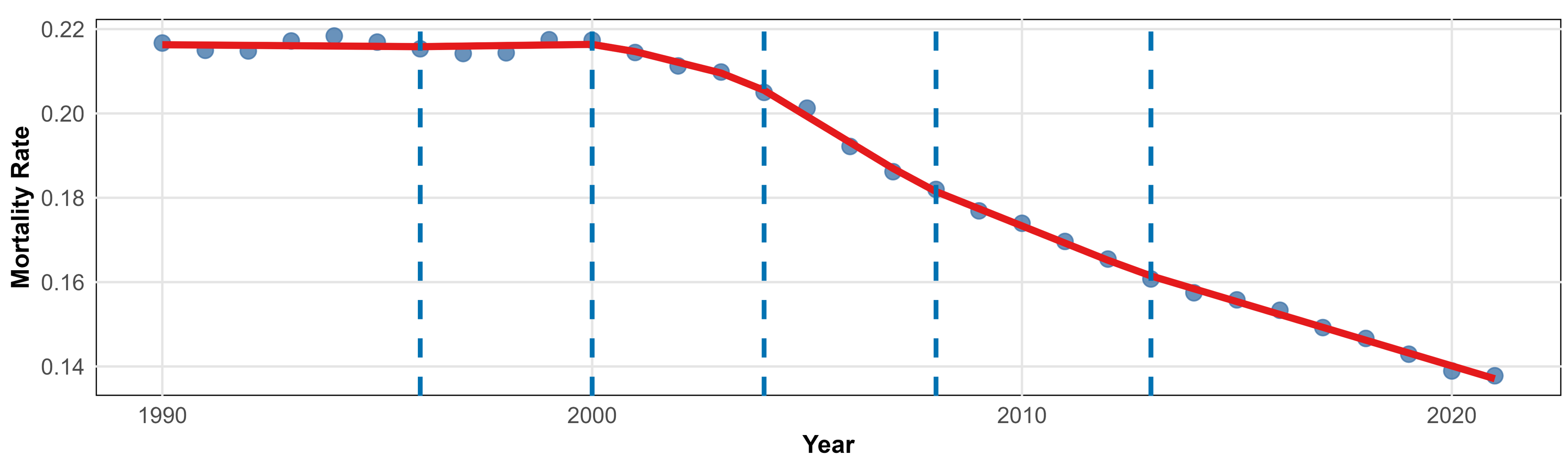

Segment	Period	CI
Segment 1	1990 - 1996	-0.01% (-0.07%, 0.05%)
Segment 2	1996 - 2000	0.01% (-0.09%, 0.11%)
Segment 3	2000 - 2004	-0.25% (-0.45%, -0.06%)
Segment 4	2004 - 2008	-0.62% (-0.82%, -0.41%)
Segment 5	2008 - 2013	-0.41% (-0.54%, -0.27%)
Segment 6	2013 - 2021	-0.30% (-0.39%, -0.22%)
**AAPC**	**1990 - 2021**	**-0.26% (-0.31%, -0.20%)**

Joinpoint Analysis of Dietary Risk Factors' Impact on All Cancer Mortality in Middle SDI Region (1990-2021)

Joinpoint Analysis of All Dietary Risks Impact on All Cancer Mortality in Middle SDI

Joinpoint Years: 1995, 1999, 2005, 2006, 2015

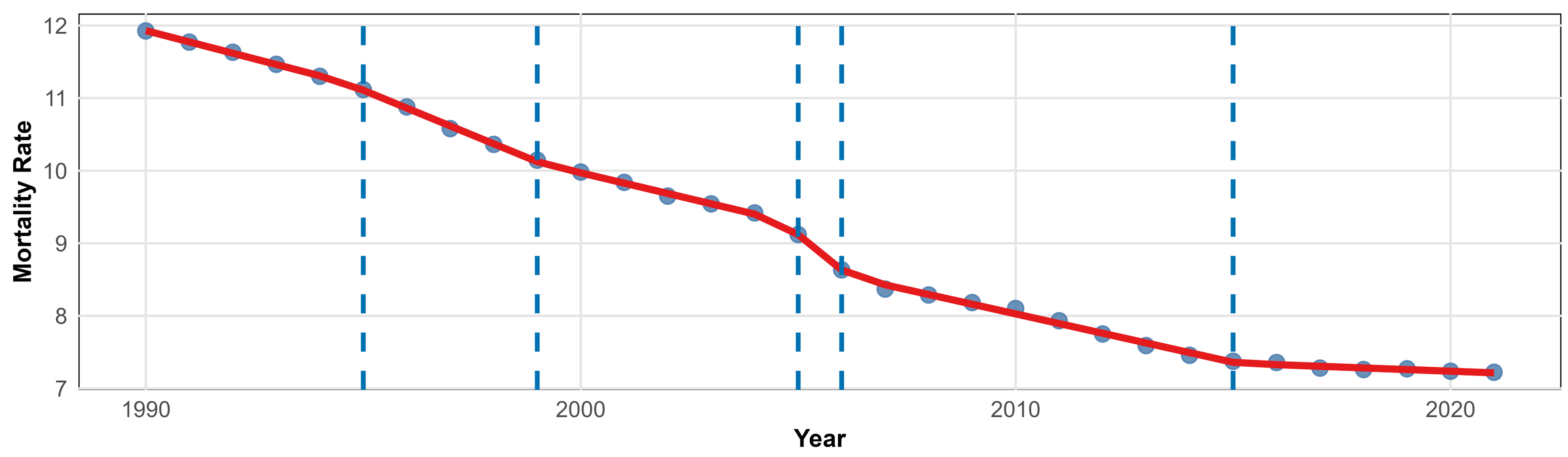

Segment	Period	CI
Segment 1	1990 - 1995	-14.41% (-16.09%, -12.71%)
Segment 2	1995 - 1999	-21.82% (-23.98%, -19.61%)
Segment 3	1999 - 2005	-13.26% (-15.65%, -10.80%)
Segment 4	2005 - 2006	-38.56% (-43.88%, -32.74%)
Segment 5	2006 - 2015	-12.49% (-19.93%, -4.37%)
Segment 6	2015 - 2021	-2.23% (-3.88%, -0.56%)
**AAPC**	**1990 - 2021**	**-13.01% (-15.34%, -10.62%)**

Joinpoint Analysis of Diet Low in Vegetables Impact on All Cancer Mortality in Middle SDI

Joinpoint Years: 1995, 1998, 2008, 2013, 2018

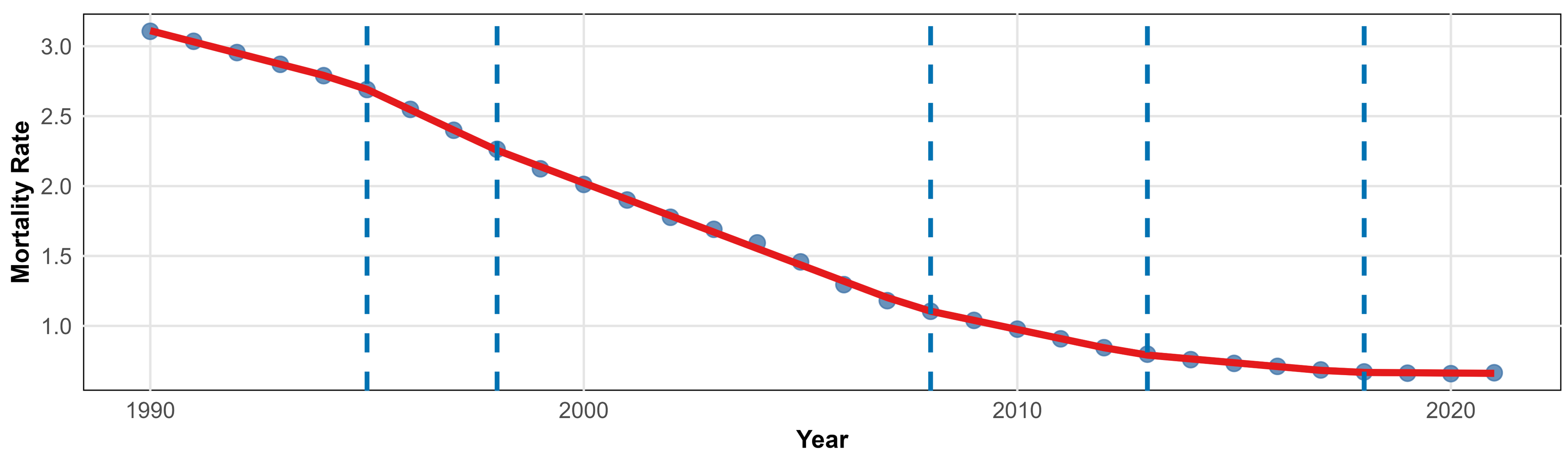

Segment	Period	CI
Segment 1	1990 - 1995	-7.68% (-8.52%, -6.84%)
Segment 2	1995 - 1998	-13.55% (-15.46%, -11.61%)
Segment 3	1998 - 2008	-11.05% (-12.86%, -9.20%)
Segment 4	2008 - 2013	-6.32% (-7.22%, -5.41%)
Segment 5	2013 - 2018	-2.70% (-3.94%, -1.44%)
Segment 6	2018 - 2021	-0.21% (-1.77%, 1.37%)
**AAPC**	**1990 - 2021**	**-7.59% (-8.30%, -6.87%)**

Joinpoint Analysis of Diet High in Sodium Impact on All Cancer Mortality in Middle SDI

Joinpoint Years: 1999, 2005, 2006, 2015

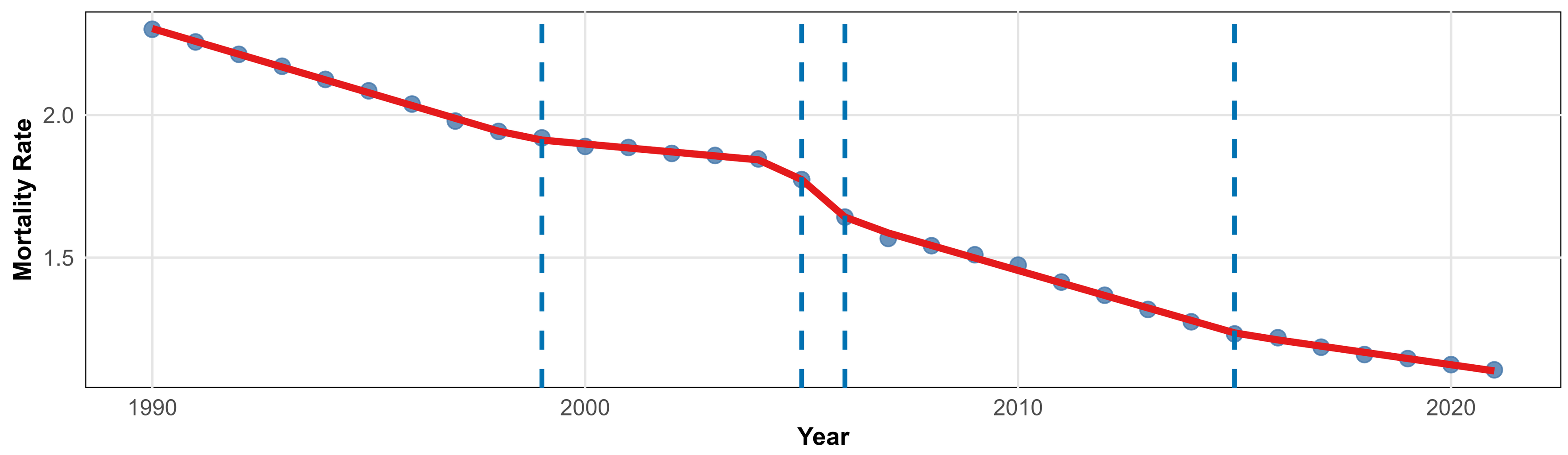

Segment	Period	CI
Segment 1	1990 - 1999	-4.40% (-4.59%, -4.21%)
Segment 2	1999 - 2005	-1.38% (-1.80%, -0.95%)
Segment 3	2005 - 2006	-12.33% (-14.28%, -10.33%)
Segment 4	2006 - 2015	-4.29% (-6.40%, -2.13%)
Segment 5	2015 - 2021	-2.15% (-2.57%, -1.73%)
**AAPC**	**1990 - 2021**	**-3.60% (-4.24%, -2.96%)**

Joinpoint Analysis of Diet Low in Calcium Impact on All Cancer Mortality in Middle SDI

Joinpoint Years: 1999, 2005, 2006, 2010, 2014

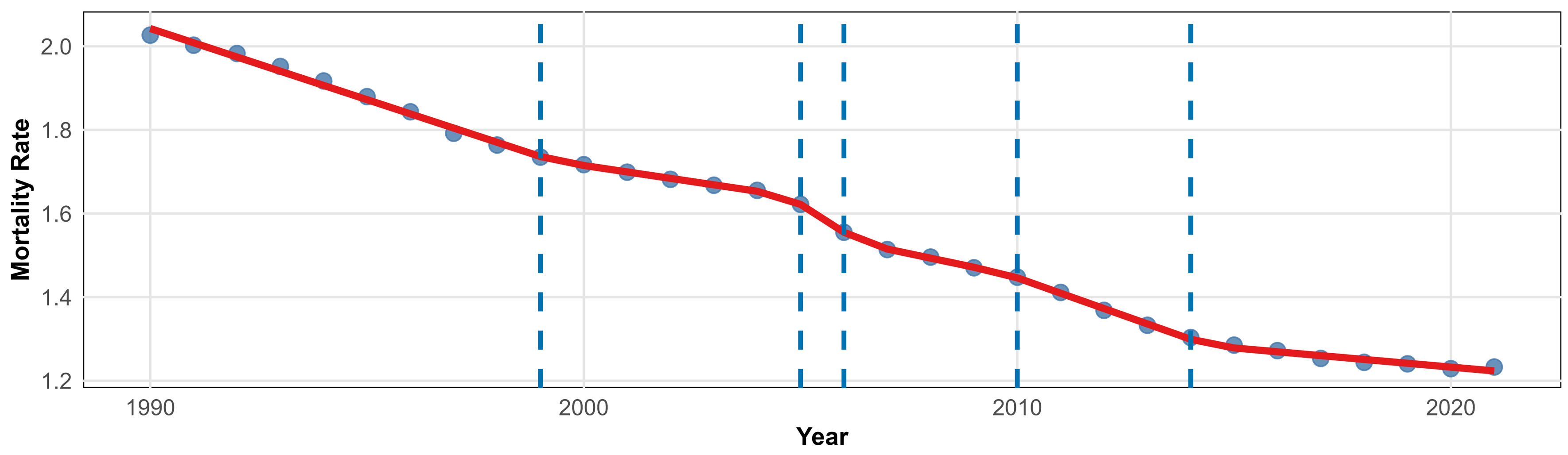

Segment	Period	CI
Segment 1	1990 - 1999	-3.34% (-3.51%, -3.18%)
Segment 2	1999 - 2005	-1.53% (-2.03%, -1.03%)
Segment 3	2005 - 2006	-6.45% (-8.48%, -4.38%)
Segment 4	2006 - 2010	-2.16% (-4.47%, 0.21%)
Segment 5	2010 - 2014	-3.61% (-4.73%, -2.47%)
Segment 6	2014 - 2021	-0.91% (-1.46%, -0.36%)
**AAPC**	**1990 - 2021**	**-2.42% (-2.80%, -2.04%)**

Joinpoint Analysis of Diet Low in Fruits Impact on All Cancer Mortality in Middle SDI

Joinpoint Years: 1995, 2005, 2006, 2015

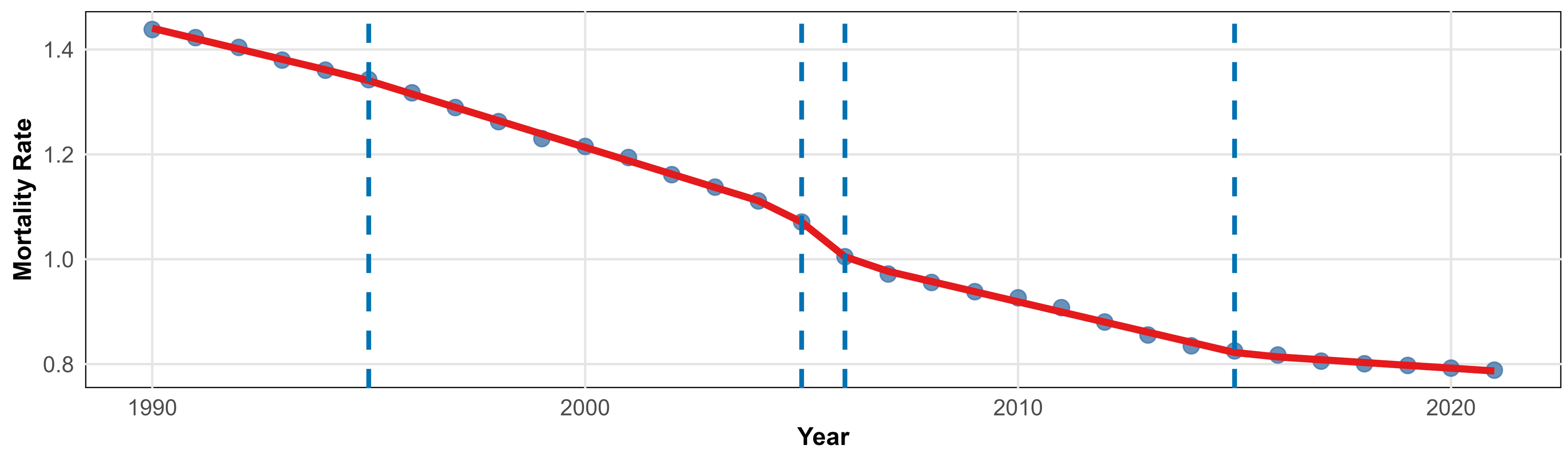

Segment	Period	CI
Segment 1	1990 - 1995	-1.96% (-2.22%, -1.70%)
Segment 2	1995 - 2005	-2.51% (-2.79%, -2.24%)
Segment 3	2005 - 2006	-6.39% (-7.51%, -5.25%)
Segment 4	2006 - 2015	-1.92% (-3.09%, -0.72%)
Segment 5	2015 - 2021	-0.53% (-0.76%, -0.30%)
**AAPC**	**1990 - 2021**	**-1.99% (-2.35%, -1.63%)**

Joinpoint Analysis of Diet High in Red Meat Impact on All Cancer Mortality in Middle SDI

Joinpoint Years: 1994, 2005, 2006, 2010, 2014

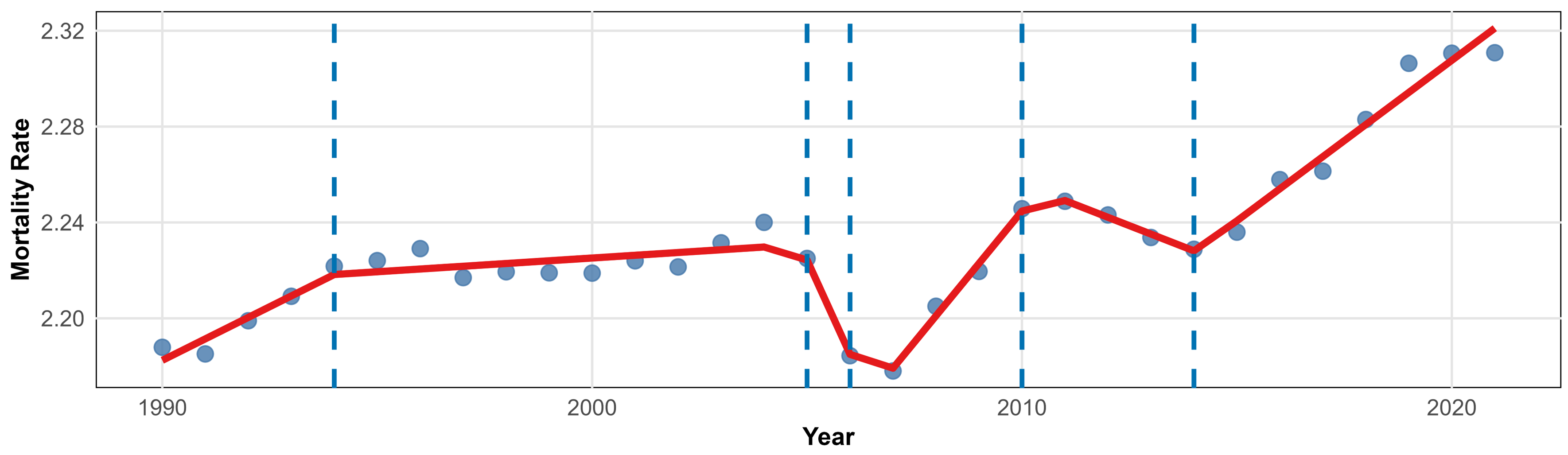

Segment	Period	CI
Segment 1	1990 - 1994	0.90% (0.50%, 1.30%)
Segment 2	1994 - 2005	0.11% (-0.30%, 0.53%)
Segment 3	2005 - 2006	-3.86% (-5.54%, -2.15%)
Segment 4	2006 - 2010	2.21% (0.34%, 4.11%)
Segment 5	2010 - 2014	-0.70% (-1.47%, 0.09%)
Segment 6	2014 - 2021	1.35% (0.74%, 1.96%)
**AAPC**	**1990 - 2021**	**0.53% (0.19%, 0.87%)**

Joinpoint Analysis of Diet Low in Fiber Impact on All Cancer Mortality in Middle SDI

Joinpoint Years: 1994, 1998, 2004, 2014, 2018

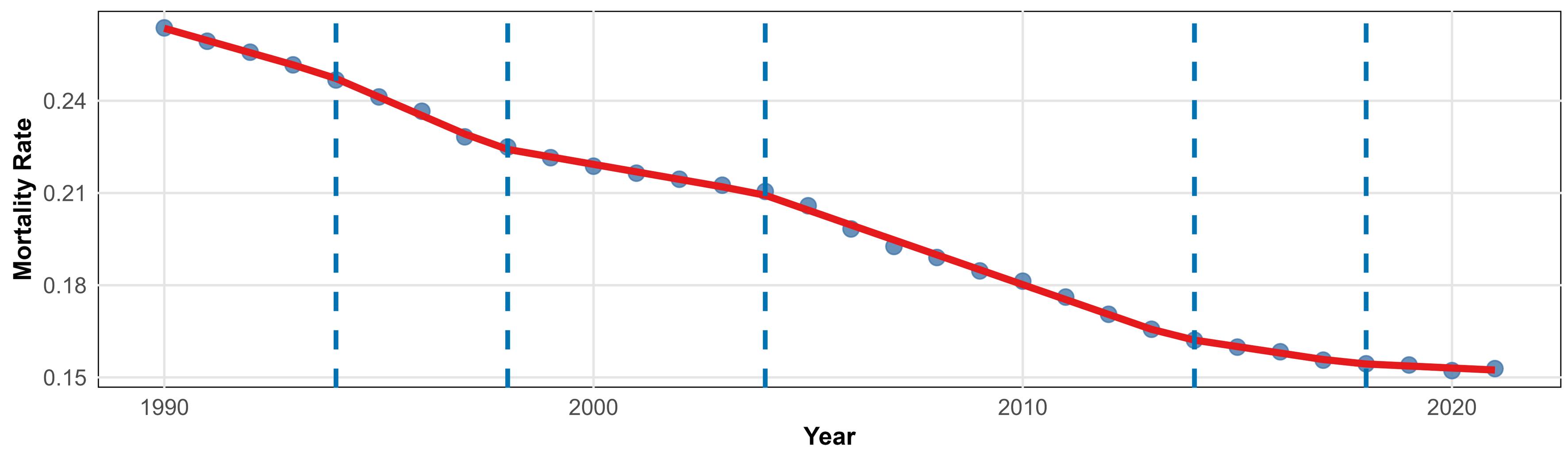

Segment	Period	CI
Segment 1	1990 - 1994	-0.40% (-0.48%, -0.31%)
Segment 2	1994 - 1998	-0.60% (-0.72%, -0.48%)
Segment 3	1998 - 2004	-0.24% (-0.34%, -0.15%)
Segment 4	2004 - 2014	-0.48% (-0.53%, -0.43%)
Segment 5	2014 - 2018	-0.21% (-0.30%, -0.12%)
Segment 6	2018 - 2021	-0.07% (-0.18%, 0.05%)
**AAPC**	**1990 - 2021**	**-0.37% (-0.40%, -0.33%)**

Joinpoint Analysis of Diet Low in Milk Impact on All Cancer Mortality in Middle SDI

Joinpoint Years: 2005, 2007, 2010, 2014

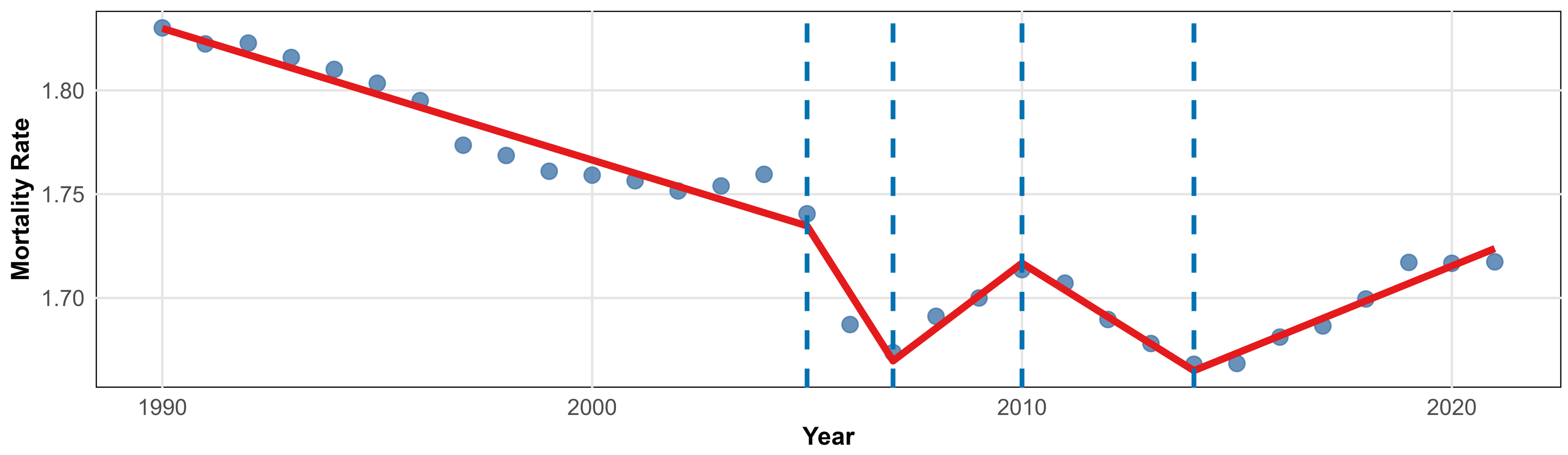

Segment	Period	CI
Segment 1	1990 - 2005	-0.63% (-0.73%, -0.54%)
Segment 2	2005 - 2007	-3.20% (-4.31%, -2.08%)
Segment 3	2007 - 2010	1.58% (-0.05%, 3.24%)
Segment 4	2010 - 2014	-1.29% (-2.87%, 0.32%)
Segment 5	2014 - 2021	0.84% (-0.33%, 2.03%)
**AAPC**	**1990 - 2021**	**-0.33% (-0.71%, 0.05%)**

Joinpoint Analysis of Diet High in Processed Meat Impact on All Cancer Mortality in Middle SDI

Joinpoint Years: 1999, 2007, 2011, 2019

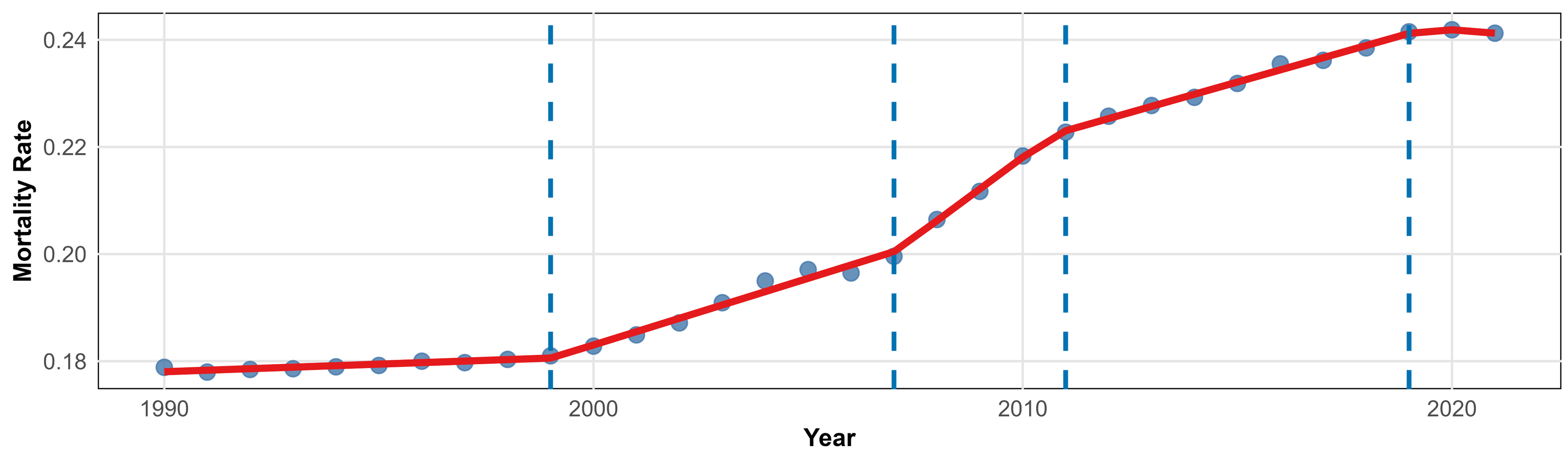

Segment	Period	CI
Segment 1	1990 - 1999	0.03% (0.01%, 0.05%)
Segment 2	1999 - 2007	0.25% (0.22%, 0.28%)
Segment 3	2007 - 2011	0.60% (0.48%, 0.71%)
Segment 4	2011 - 2019	0.23% (0.11%, 0.34%)
Segment 5	2019 - 2021	-0.06% (-0.29%, 0.17%)
**AAPC**	**1990 - 2021**	**0.20% (0.17%, 0.24%)**

Joinpoint Analysis of Diet Low in Whole Grains Impact on All Cancer Mortality in Middle SDI

Joinpoint Years: 2001, 2005, 2006, 2009, 2019

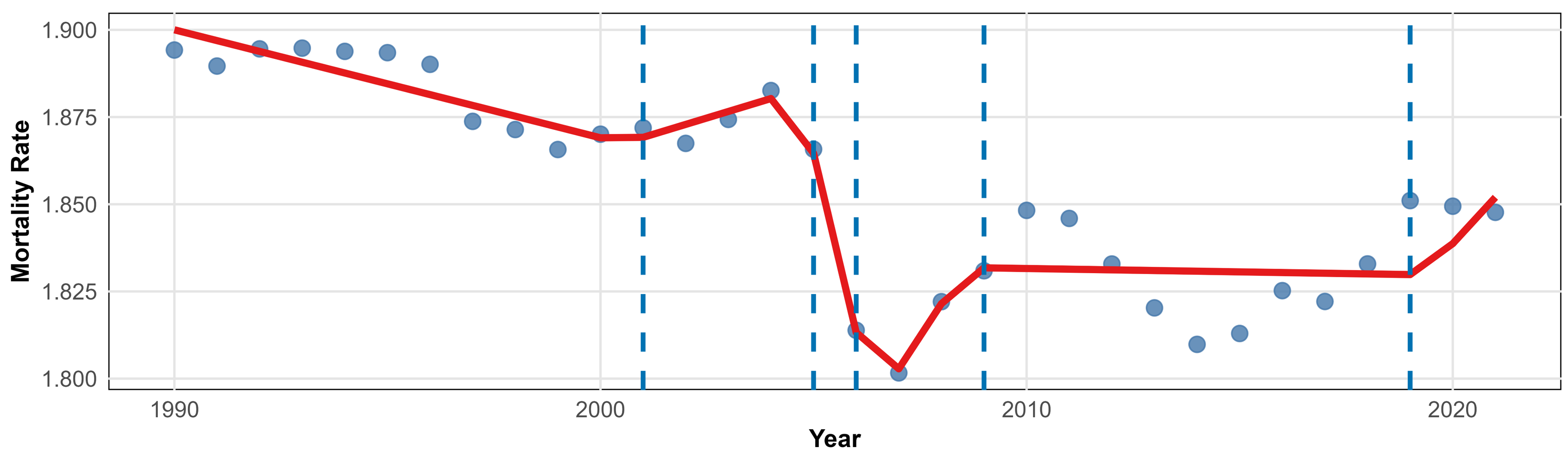

Segment	Period	CI
Segment 1	1990 - 2001	-0.31% (-0.52%, -0.10%)
Segment 2	2001 - 2005	0.37% (-0.62%, 1.38%)
Segment 3	2005 - 2006	-5.01% (-8.03%, -1.89%)
Segment 4	2006 - 2009	1.88% (-2.46%, 6.42%)
Segment 5	2009 - 2019	-0.02% (-3.06%, 3.12%)
Segment 6	2019 - 2021	1.34% (-1.74%, 4.51%)
**AAPC**	**1990 - 2021**	**0.04% (-1.07%, 1.16%)**

Joinpoint Analysis of Dietary Risk Factors' Impact on All Cancer Mortality in Low-middle SDI Region (1990-2021)

Joinpoint Analysis of Diet High in Red Meat Impact on All Cancer Mortality in Low-middle SDI

Joinpoint Years: 1997, 2001, 2003, 2012, 2018

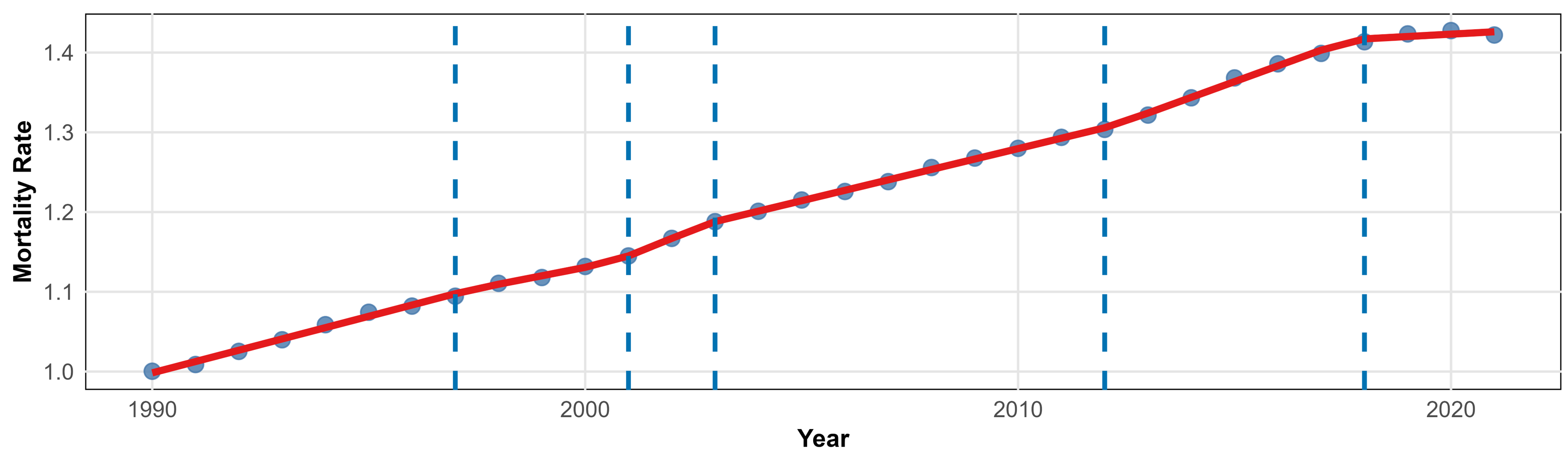

Segment	Period	CI
Segment 1	1990 - 1997	1.43% (1.33%, 1.53%)
Segment 2	1997 - 2001	1.06% (0.60%, 1.53%)
Segment 3	2001 - 2003	2.23% (1.20%, 3.26%)
Segment 4	2003 - 2012	1.32% (0.40%, 2.24%)
Segment 5	2012 - 2018	1.99% (1.78%, 2.21%)
Segment 6	2018 - 2021	0.30% (-0.05%, 0.65%)
**AAPC**	**1990 - 2021**	**1.40% (1.11%, 1.69%)**

Joinpoint Analysis of Diet High in Sodium Impact on All Cancer Mortality in Low-middle SDI

Joinpoint Years: 2006, 2007, 2019

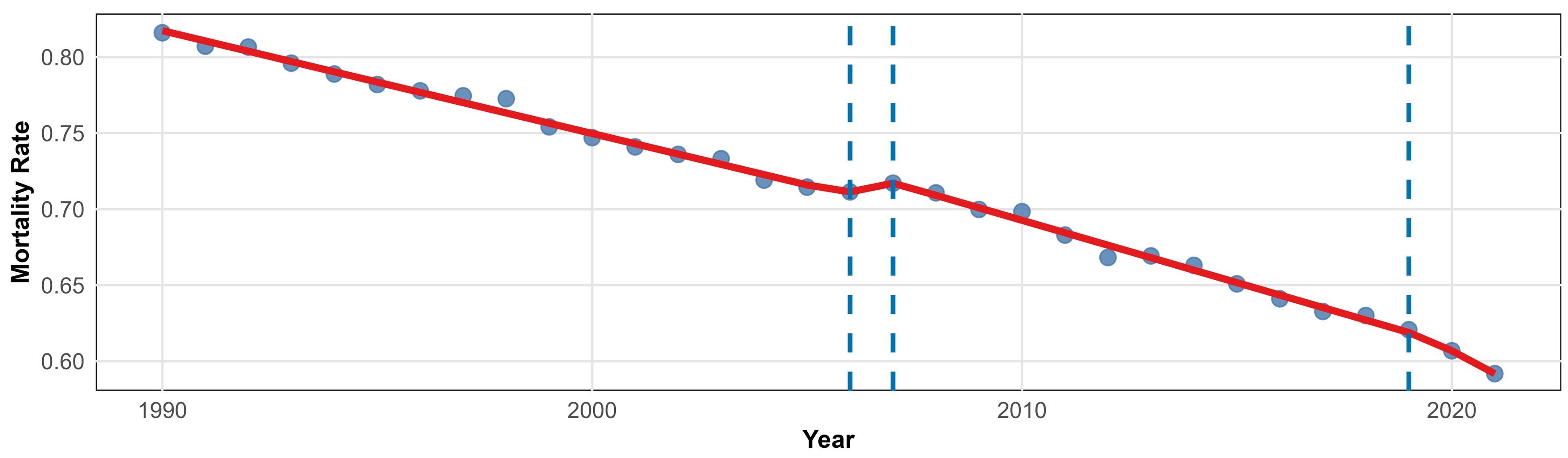

Segment	Period	CI
Segment 1	1990 - 2006	-0.67% (-0.71%, -0.64%)
Segment 2	2006 - 2007	0.57% (-0.45%, 1.60%)
Segment 3	2007 - 2019	-0.82% (-1.82%, 0.20%)
Segment 4	2019 - 2021	-1.49% (-2.49%, -0.48%)
**AAPC**	**1990 - 2021**	**-0.74% (-1.14%, -0.34%)**

Joinpoint Analysis of Diet Low in Calcium Impact on All Cancer Mortality in Low-middle SDI

Joinpoint Years: 2002, 2003, 2008, 2012, 2018

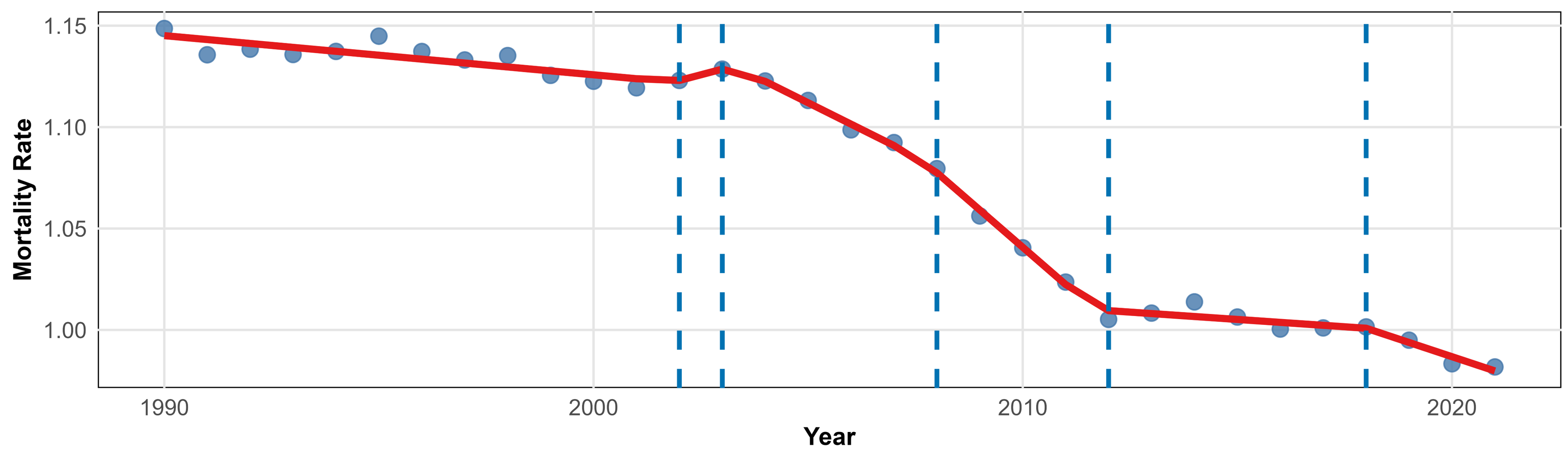

Segment	Period	CI
Segment 1	1990 - 2002	-0.19% (-0.26%, -0.12%)
Segment 2	2002 - 2003	0.56% (-0.66%, 1.80%)
Segment 3	2003 - 2008	-1.05% (-2.31%, 0.23%)
Segment 4	2008 - 2012	-1.82% (-2.35%, -1.28%)
Segment 5	2012 - 2018	-0.14% (-0.56%, 0.27%)
Segment 6	2018 - 2021	-0.70% (-1.32%, -0.07%)
**AAPC**	**1990 - 2021**	**-0.56% (-0.80%, -0.31%)**

Joinpoint Analysis of Diet Low in Vegetables Impact on All Cancer Mortality in Low-middle SDI

Joinpoint Years: 1997, 2000, 2008, 2013, 2019

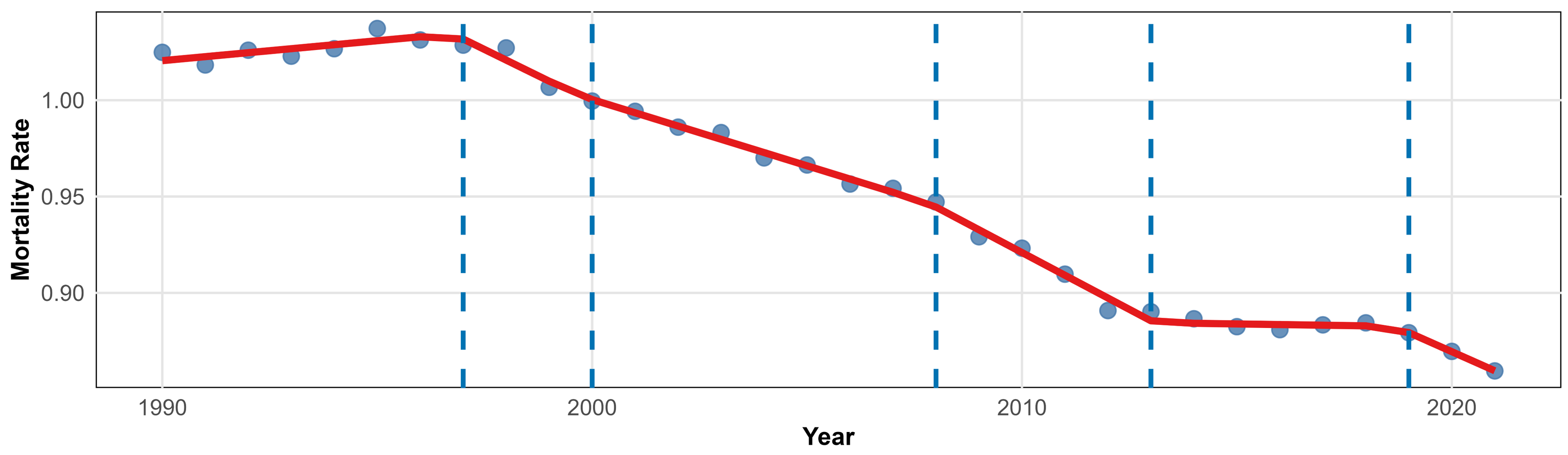

Segment	Period	CI
Segment 1	1990 - 1997	0.21% (0.06%, 0.35%)
Segment 2	1997 - 2000	-1.09% (-1.64%, -0.54%)
Segment 3	2000 - 2008	-0.69% (-1.23%, -0.14%)
Segment 4	2008 - 2013	-1.17% (-1.39%, -0.96%)
Segment 5	2013 - 2019	-0.03% (-0.33%, 0.27%)
Segment 6	2019 - 2021	-0.99% (-1.57%, -0.40%)
**AAPC**	**1990 - 2021**	**-0.49% (-0.67%, -0.32%)**

Joinpoint Analysis of Diet Low in Milk Impact on All Cancer Mortality in Low-middle SDI

Joinpoint Years: 1998, 2000, 2005, 2012, 2018

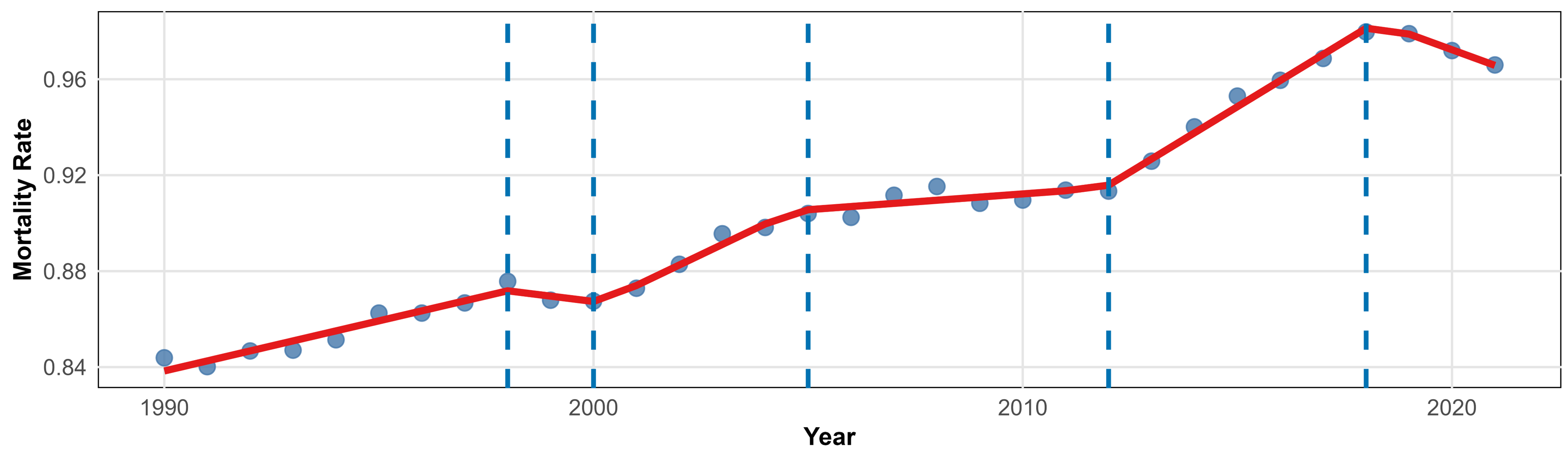

Segment	Period	CI
Segment 1	1990 - 1998	0.42% (0.31%, 0.52%)
Segment 2	1998 - 2000	-0.22% (-0.71%, 0.27%)
Segment 3	2000 - 2005	0.86% (0.29%, 1.43%)
Segment 4	2005 - 2012	0.13% (-0.20%, 0.46%)
Segment 5	2012 - 2018	1.10% (0.91%, 1.28%)
Segment 6	2018 - 2021	-0.65% (-1.14%, -0.16%)
**AAPC**	**1990 - 2021**	**0.41% (0.27%, 0.55%)**

Joinpoint Analysis of Diet Low in Whole Grains Impact on All Cancer Mortality in Low-middle SDI

Joinpoint Years: 1993, 1995, 2008, 2012, 2018

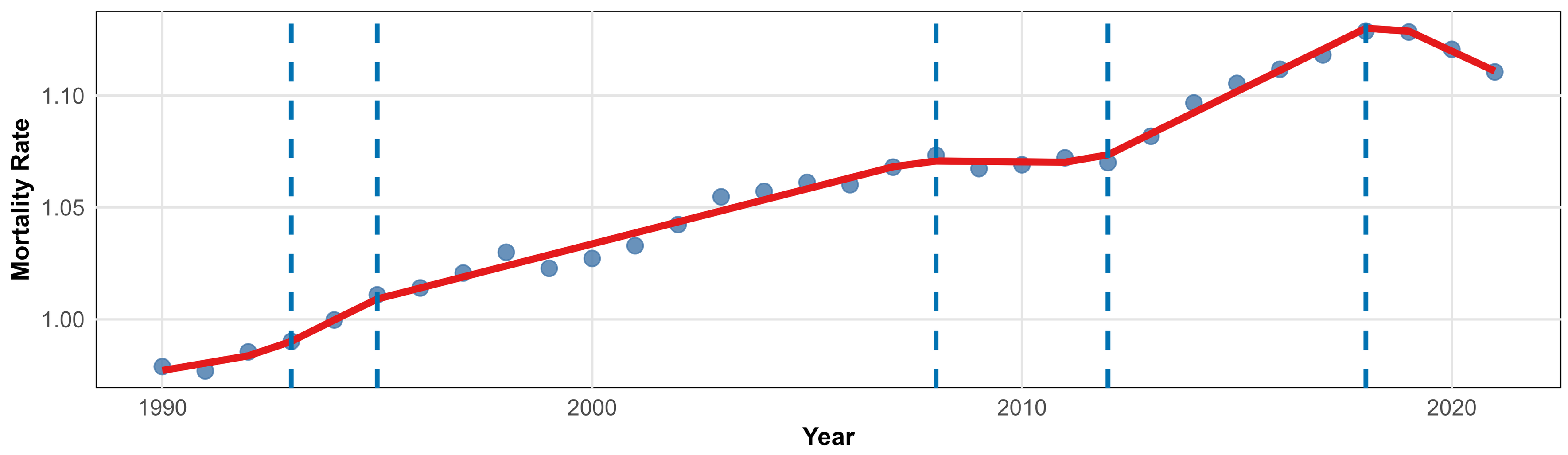

Segment	Period	CI
Segment 1	1990 - 1993	0.33% (-0.23%, 0.89%)
Segment 2	1993 - 1995	0.96% (-0.29%, 2.23%)
Segment 3	1995 - 2008	0.49% (-0.62%, 1.62%)
Segment 4	2008 - 2012	-0.02% (-0.37%, 0.34%)
Segment 5	2012 - 2018	0.95% (0.56%, 1.33%)
Segment 6	2018 - 2021	-0.89% (-1.46%, -0.31%)
**AAPC**	**1990 - 2021**	**0.40% (-0.09%, 0.89%)**

Joinpoint Analysis of Diet Low in Fruits Impact on All Cancer Mortality in Low-middle SDI

Joinpoint Years: 1996, 2006, 2015, 2019

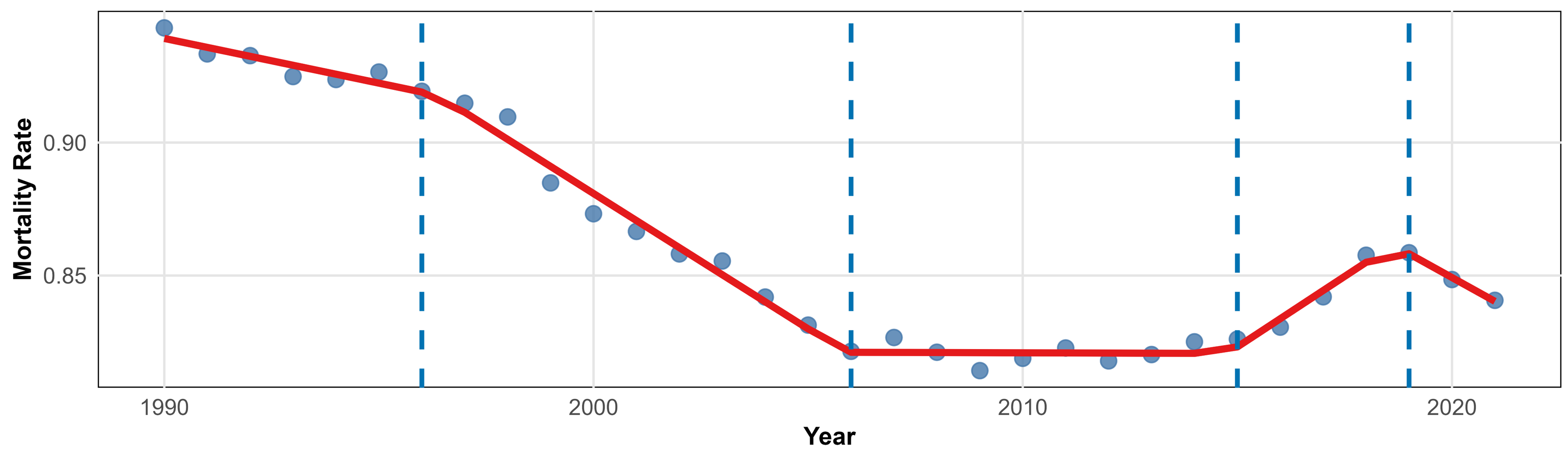

Segment	Period	CI
Segment 1	1990 - 1996	-0.34% (-0.50%, -0.17%)
Segment 2	1996 - 2006	-1.01% (-1.21%, -0.81%)
Segment 3	2006 - 2015	-0.00% (-0.16%, 0.16%)
Segment 4	2015 - 2019	1.07% (0.66%, 1.48%)
Segment 5	2019 - 2021	-0.89% (-1.61%, -0.16%)
**AAPC**	**1990 - 2021**	**-0.31% (-0.42%, -0.20%)**

Joinpoint Analysis of All Dietary Risks Impact on All Cancer Mortality in Low-middle SDI

Joinpoint Years: 1997, 1999, 2007, 2012, 2019

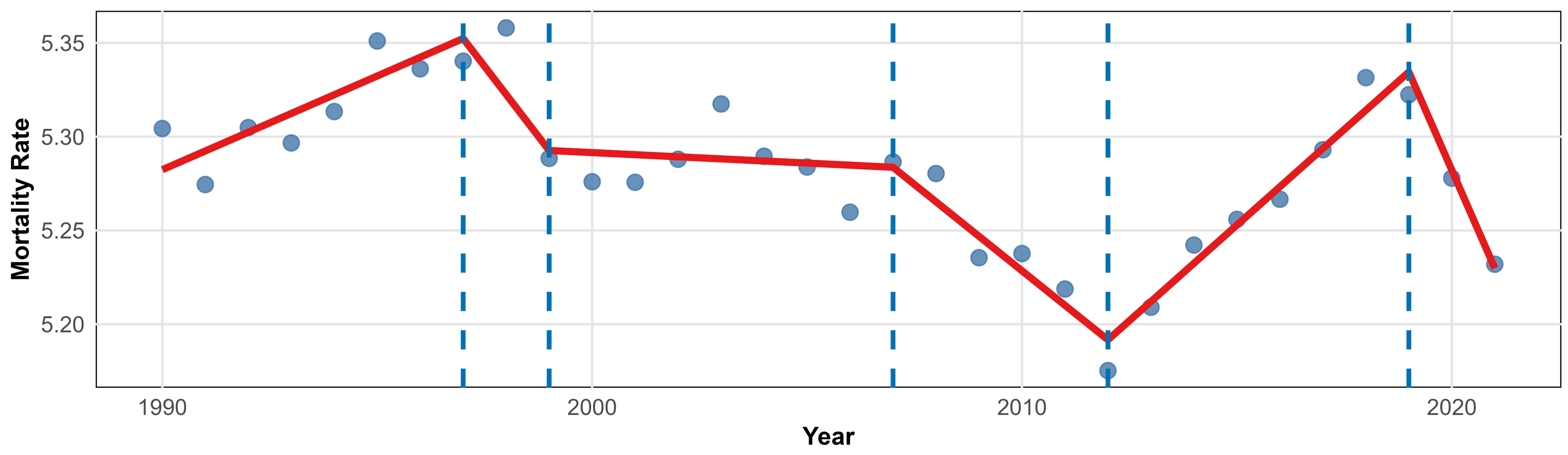

Segment	Period	CI
Segment 1	1990 - 1997	1.00% (0.34%, 1.67%)
Segment 2	1997 - 1999	-2.94% (-5.39%, -0.43%)
Segment 3	1999 - 2007	-0.11% (-2.63%, 2.47%)
Segment 4	2007 - 2012	-1.82% (-3.08%, -0.55%)
Segment 5	2012 - 2019	2.06% (0.82%, 3.32%)
Segment 6	2019 - 2021	-5.10% (-9.69%, -0.27%)
**AAPC**	**1990 - 2021**	**-0.15% (-0.98%, 0.69%)**

Joinpoint Analysis of Diet Low in Fiber Impact on All Cancer Mortality in Low-middle SDI

Joinpoint Years: 1998, 2004, 2012

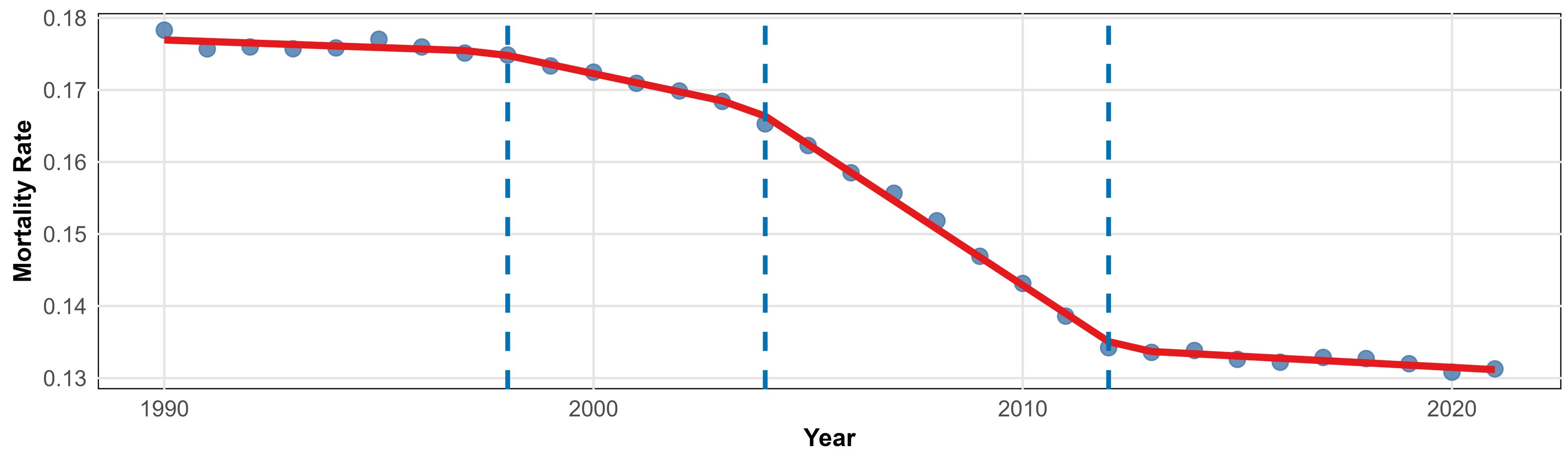

Segment	Period	CI
Segment 1	1990 - 1998	-0.02% (-0.04%, -0.00%)
Segment 2	1998 - 2004	-0.13% (-0.16%, -0.09%)
Segment 3	2004 - 2012	-0.39% (-0.43%, -0.35%)
Segment 4	2012 - 2021	-0.03% (-0.06%, -0.01%)
**AAPC**	**1990 - 2021**	**-0.14% (-0.15%, -0.12%)**

Joinpoint Analysis of Diet High in Processed Meat Impact on All Cancer Mortality in Low-middle SDI

Joinpoint Years: 1999, 2013, 2018

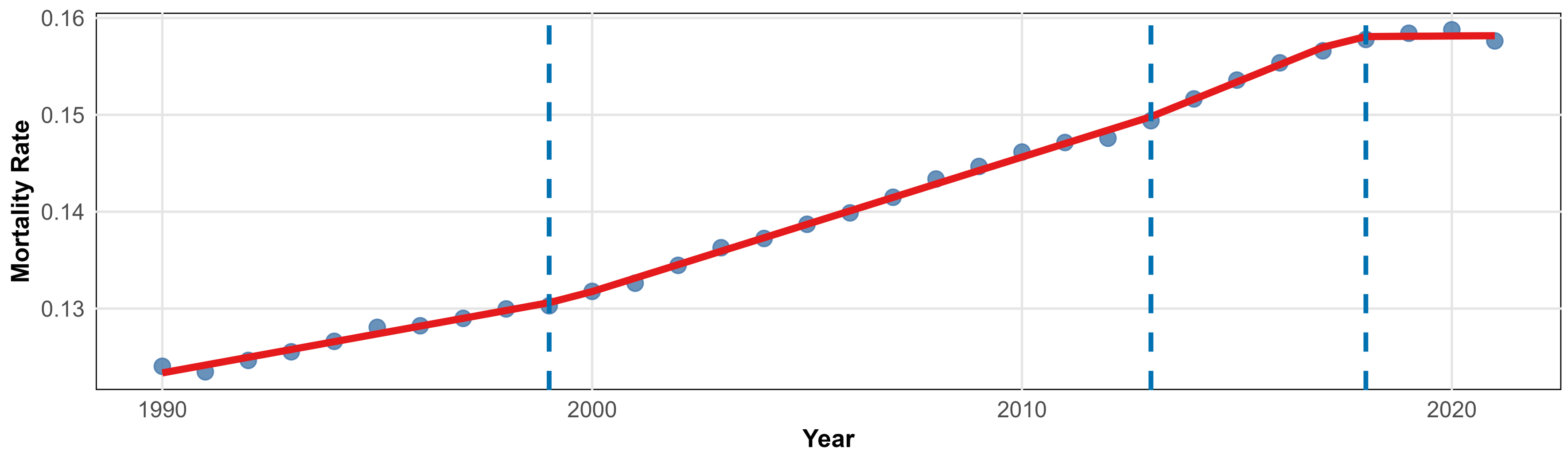

Segment	Period	CI
Segment 1	1990 - 1999	0.08% (0.07%, 0.09%)
Segment 2	1999 - 2013	0.14% (0.13%, 0.15%)
Segment 3	2013 - 2018	0.18% (0.14%, 0.22%)
Segment 4	2018 - 2021	0.00% (-0.05%, 0.06%)
**AAPC**	**1990 - 2021**	**0.12% (0.11%, 0.13%)**

Joinpoint Analysis of Dietary Risk Factors' Impact on All Cancer Mortality in Low SDI Region (1990-2021)

Joinpoint Analysis of All Dietary Risks Impact on All Cancer Mortality in Low SDI

Joinpoint Years: 1996, 2008, 2012, 2015

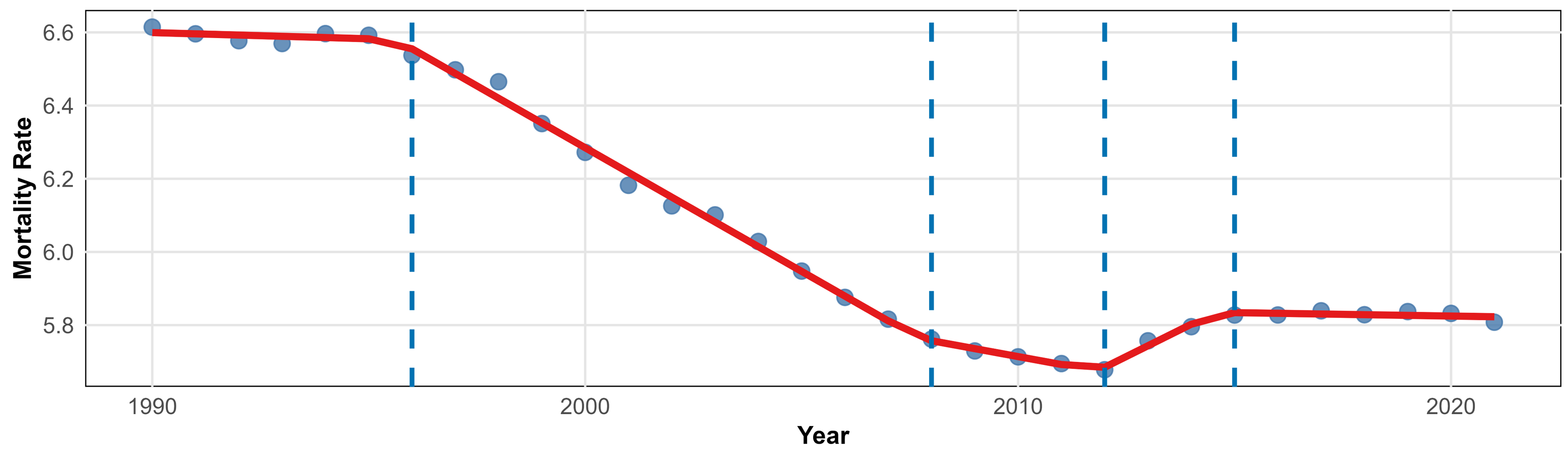

Segment	Period	CI
Segment 1	1990 - 1996	-0.34% (-1.16%, 0.50%)
Segment 2	1996 - 2008	-6.54% (-7.36%, -5.71%)
Segment 3	2008 - 2012	-2.14% (-3.68%, -0.58%)
Segment 4	2012 - 2015	6.06% (3.01%, 9.20%)
Segment 5	2015 - 2021	-0.19% (-2.70%, 2.39%)
**AAPC**	**1990 - 2021**	**-2.32% (-3.01%, -1.62%)**

Joinpoint Analysis of Diet Low in Vegetables Impact on All Cancer Mortality in Low SDI

Joinpoint Years: 1995, 1998, 2008, 2012, 2015

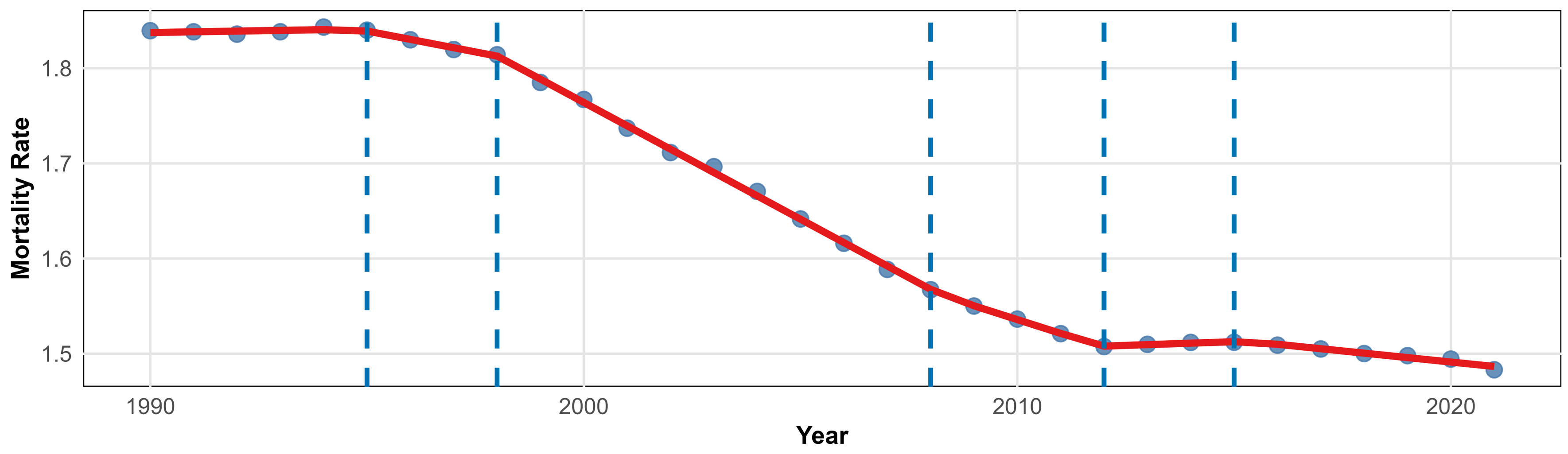

Segment	Period	CI
Segment 1	1990 - 1995	0.08% (-0.11%, 0.26%)
Segment 2	1995 - 1998	-0.87% (-1.19%, -0.56%)
Segment 3	1998 - 2008	-2.43% (-2.68%, -2.17%)
Segment 4	2008 - 2012	-1.44% (-1.85%, -1.03%)
Segment 5	2012 - 2015	0.16% (-0.33%, 0.64%)
Segment 6	2015 - 2021	-0.47% (-0.76%, -0.17%)
**AAPC**	**1990 - 2021**	**-1.12% (-1.25%, -0.99%)**

Joinpoint Analysis of Diet Low in Calcium Impact on All Cancer Mortality in Low SDI

Joinpoint Years: 1996, 2010, 2012, 2015

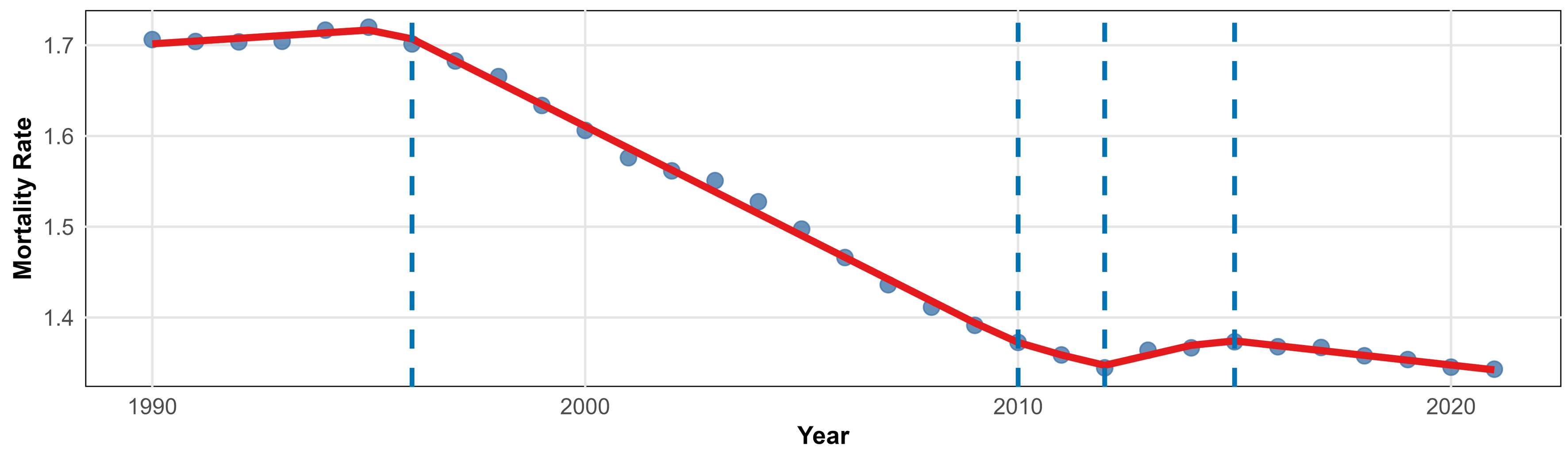

Segment	Period	CI
Segment 1	1990 - 1996	0.30% (0.02%, 0.59%)
Segment 2	1996 - 2010	-2.38% (-2.67%, -2.09%)
Segment 3	2010 - 2012	-1.37% (-3.05%, 0.33%)
Segment 4	2012 - 2015	1.11% (-0.80%, 3.06%)
Segment 5	2015 - 2021	-0.53% (-1.41%, 0.35%)
**AAPC**	**1990 - 2021**	**-1.10% (-1.41%, -0.79%)**

Joinpoint Analysis of Diet High in Sodium Impact on All Cancer Mortality in Low SDI

Joinpoint Years: 1998, 2000, 2005, 2012, 2014

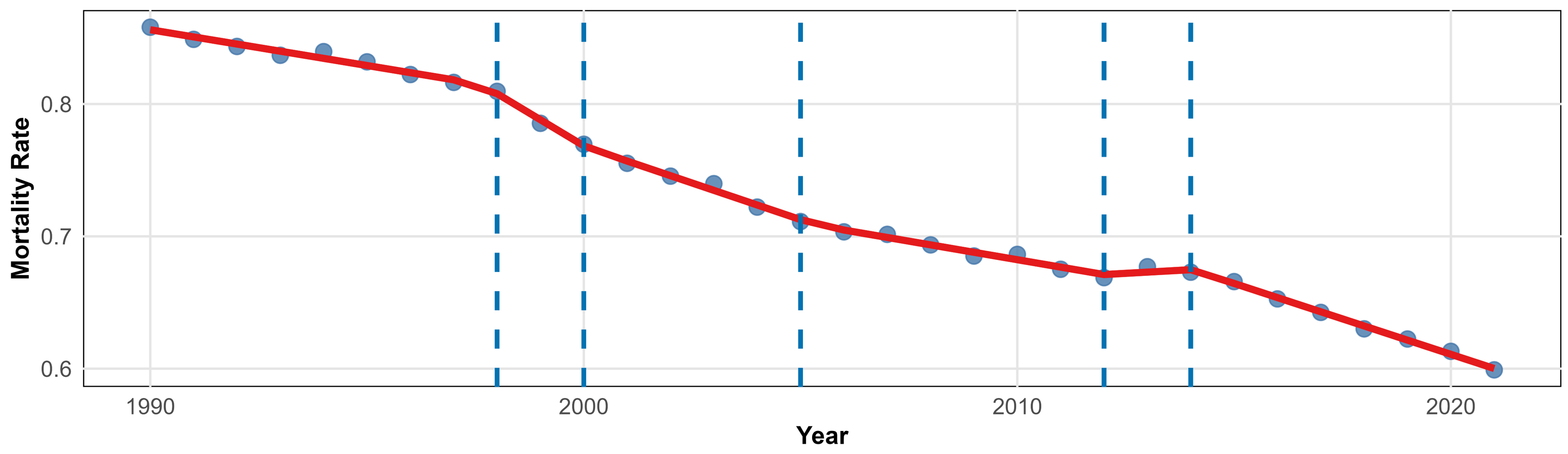

Segment	Period	CI
Segment 1	1990 - 1998	-0.54% (-0.63%, -0.45%)
Segment 2	1998 - 2000	-1.95% (-2.36%, -1.53%)
Segment 3	2000 - 2005	-1.10% (-1.55%, -0.65%)
Segment 4	2005 - 2012	-0.56% (-0.77%, -0.34%)
Segment 5	2012 - 2014	0.18% (-0.65%, 1.02%)
Segment 6	2014 - 2021	-1.07% (-1.89%, -0.24%)
**AAPC**	**1990 - 2021**	**-0.80% (-1.01%, -0.58%)**

Joinpoint Analysis of Diet High in Red Meat Impact on All Cancer Mortality in Low SDI

Joinpoint Years: 1996, 2002, 2008, 2011, 2016

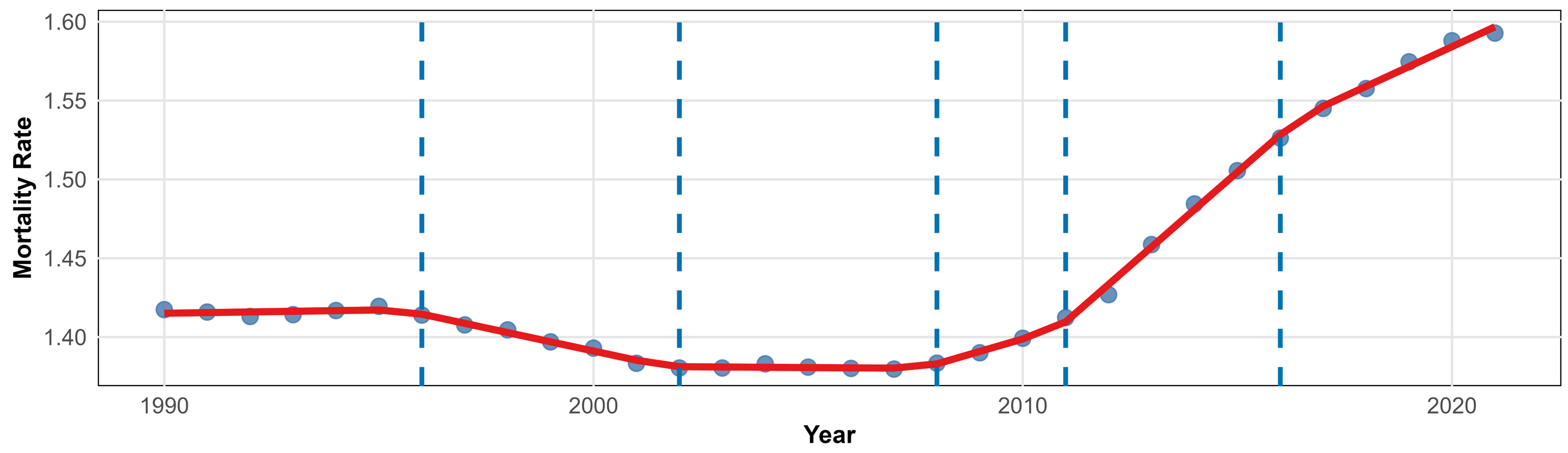

Segment	Period	CI
Segment 1	1990 - 1996	0.04% (-0.09%, 0.17%)
Segment 2	1996 - 2002	-0.58% (-0.77%, -0.40%)
Segment 3	2002 - 2008	-0.02% (-0.20%, 0.17%)
Segment 4	2008 - 2011	0.79% (0.38%, 1.21%)
Segment 5	2011 - 2016	2.40% (1.99%, 2.82%)
Segment 6	2016 - 2021	1.27% (1.05%, 1.49%)
**AAPC**	**1990 - 2021**	**0.56% (0.46%, 0.66%)**

Joinpoint Analysis of Diet Low in Milk Impact on All Cancer Mortality in Low SDI

Joinpoint Years: 1996, 2000, 2009, 2012, 2014

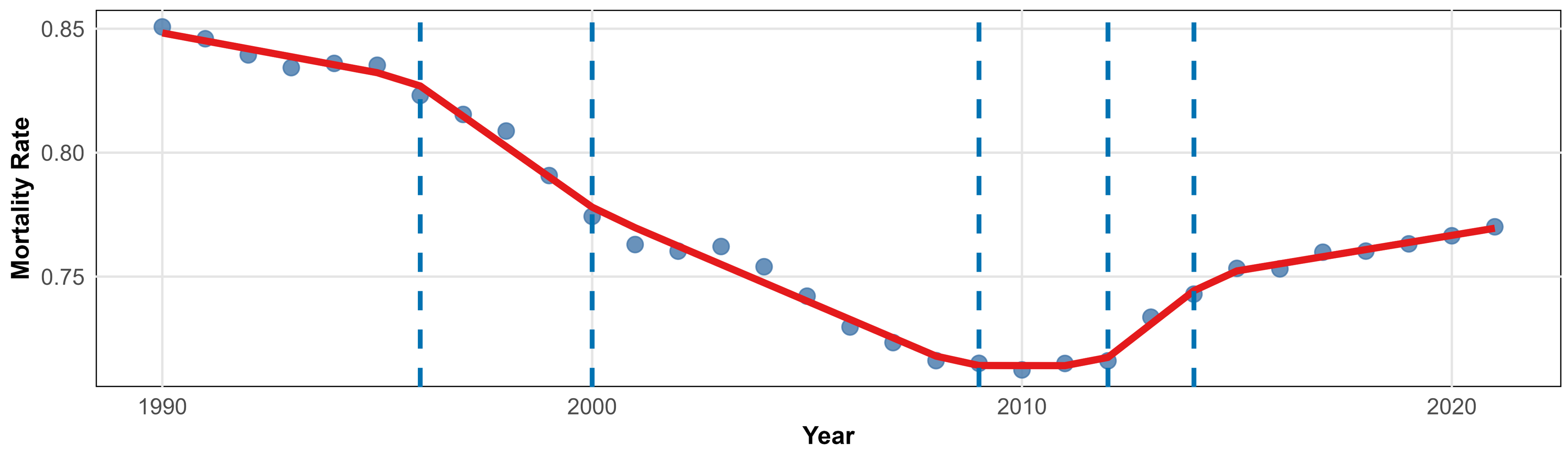

Segment	Period	CI
Segment 1	1990 - 1996	-0.32% (-0.50%, -0.14%)
Segment 2	1996 - 2000	-1.21% (-1.51%, -0.92%)
Segment 3	2000 - 2009	-0.74% (-1.00%, -0.47%)
Segment 4	2009 - 2012	-0.00% (-0.55%, 0.55%)
Segment 5	2012 - 2014	1.35% (0.59%, 2.13%)
Segment 6	2014 - 2021	0.29% (-0.27%, 0.85%)
**AAPC**	**1990 - 2021**	**-0.28% (-0.45%, -0.11%)**

Joinpoint Analysis of Diet Low in Whole Grains Impact on All Cancer Mortality in Low SDI

Joinpoint Years: 1995, 2001, 2009, 2011, 2016

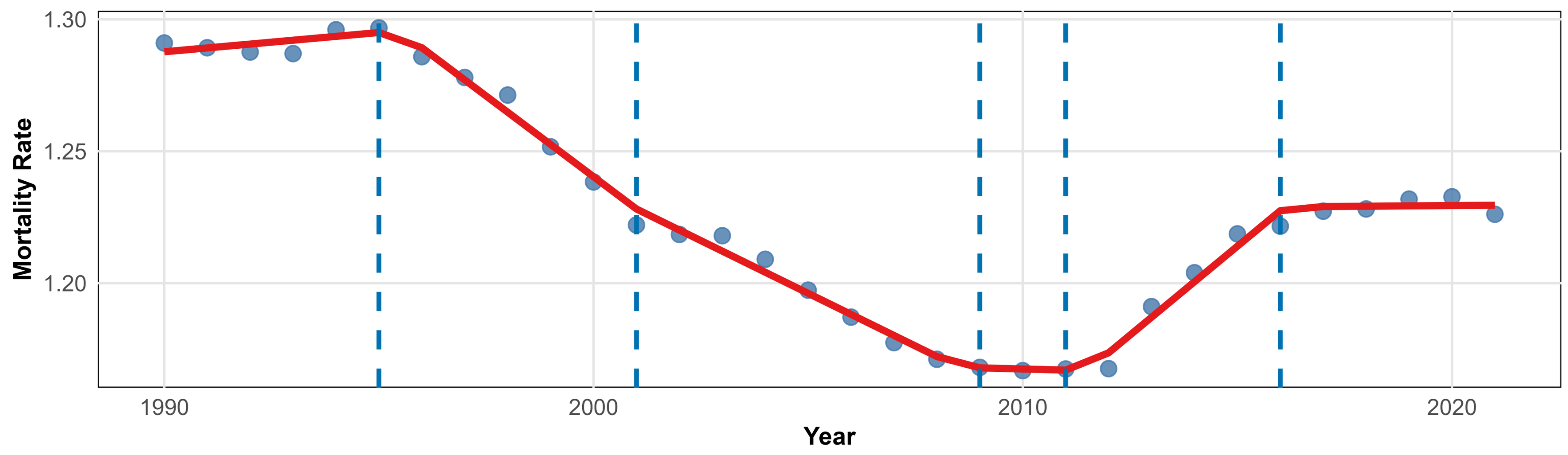

Segment	Period	CI
Segment 1	1990 - 1995	0.15% (-0.06%, 0.35%)
Segment 2	1995 - 2001	-1.21% (-1.55%, -0.88%)
Segment 3	2001 - 2009	-0.80% (-1.09%, -0.50%)
Segment 4	2009 - 2011	-0.04% (-0.66%, 0.57%)
Segment 5	2011 - 2016	1.36% (0.69%, 2.03%)
Segment 6	2016 - 2021	0.01% (-0.37%, 0.39%)
**AAPC**	**1990 - 2021**	**-0.20% (-0.37%, -0.03%)**

Joinpoint Analysis of Diet Low in Fruits Impact on All Cancer Mortality in Low SDI

Joinpoint Years: 1998, 1999, 2009, 2019

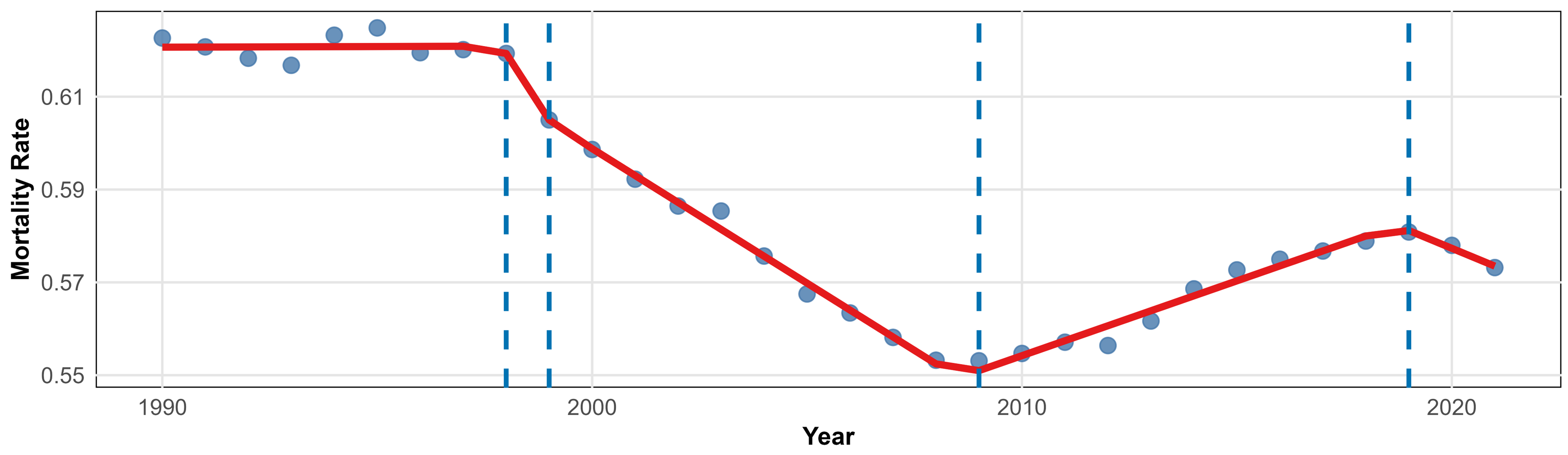

Segment	Period	CI
Segment 1	1990 - 1998	0.00% (-0.07%, 0.07%)
Segment 2	1998 - 1999	-1.42% (-2.04%, -0.80%)
Segment 3	1999 - 2009	-0.58% (-1.20%, 0.05%)
Segment 4	2009 - 2019	0.32% (0.25%, 0.40%)
Segment 5	2019 - 2021	-0.38% (-0.70%, -0.07%)
**AAPC**	**1990 - 2021**	**-0.15% (-0.36%, 0.06%)**

Joinpoint Analysis of Diet Low in Fiber Impact on All Cancer Mortality in Low SDI

Joinpoint Years: 1992, 1995, 2004, 2012, 2013

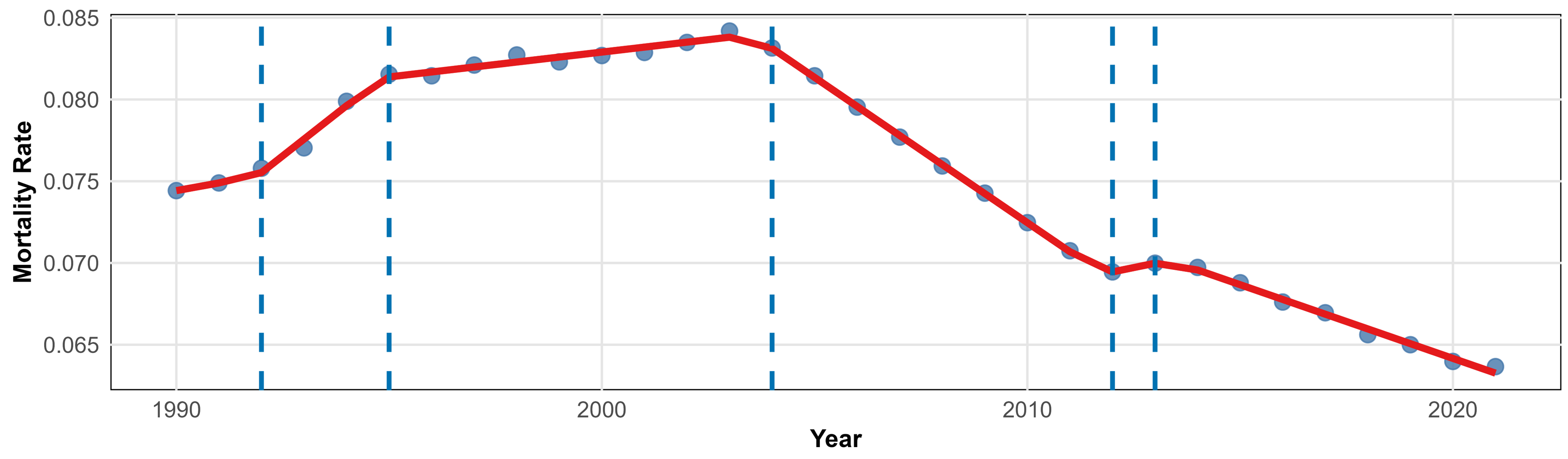

Segment	Period	CI
Segment 1	1990 - 1992	0.05% (-0.03%, 0.12%)
Segment 2	1992 - 1995	0.20% (0.12%, 0.29%)
Segment 3	1995 - 2004	0.03% (-0.01%, 0.07%)
Segment 4	2004 - 2012	-0.18% (-0.19%, -0.17%)
Segment 5	2012 - 2013	0.05% (-0.02%, 0.13%)
Segment 6	2013 - 2021	-0.09% (-0.17%, -0.01%)
**AAPC**	**1990 - 2021**	**-0.04% (-0.06%, -0.01%)**

Joinpoint Analysis of Diet High in Processed Meat Impact on All Cancer Mortality in Low SDI

Joinpoint Years: 1995, 2001, 2009, 2012, 2016

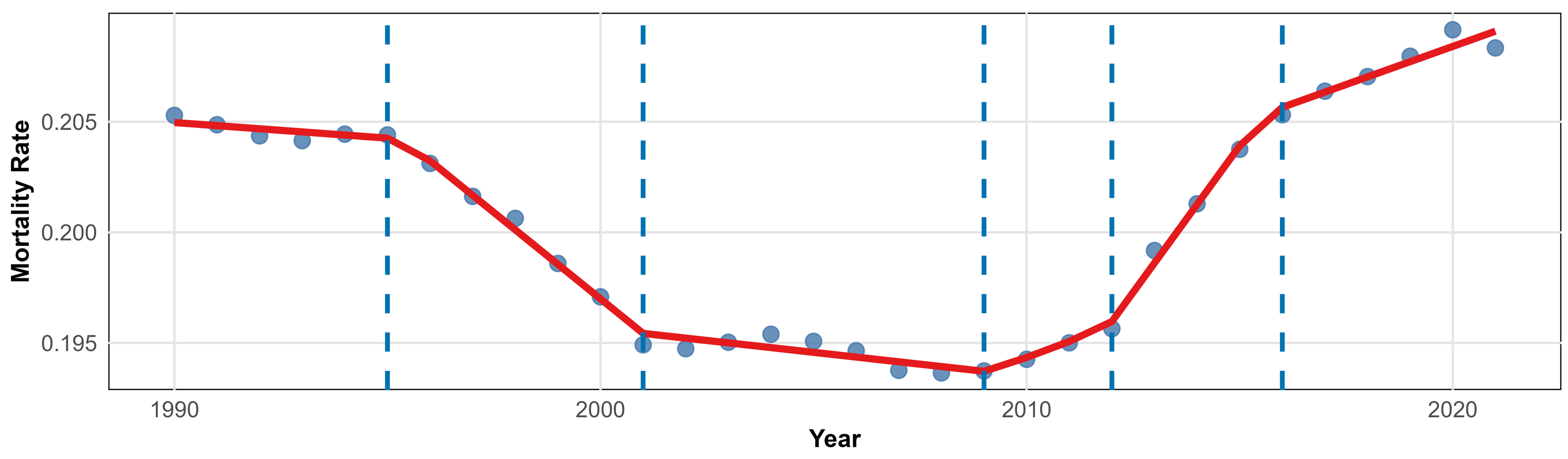

Segment	Period	CI
Segment 1	1990 - 1995	-0.01% (-0.03%, 0.01%)
Segment 2	1995 - 2001	-0.16% (-0.19%, -0.13%)
Segment 3	2001 - 2009	-0.02% (-0.05%, 0.00%)
Segment 4	2009 - 2012	0.07% (-0.05%, 0.20%)
Segment 5	2012 - 2016	0.27% (0.14%, 0.39%)
Segment 6	2016 - 2021	0.07% (0.03%, 0.11%)
**AAPC**	**1990 - 2021**	**0.01% (-0.01%, 0.04%)**

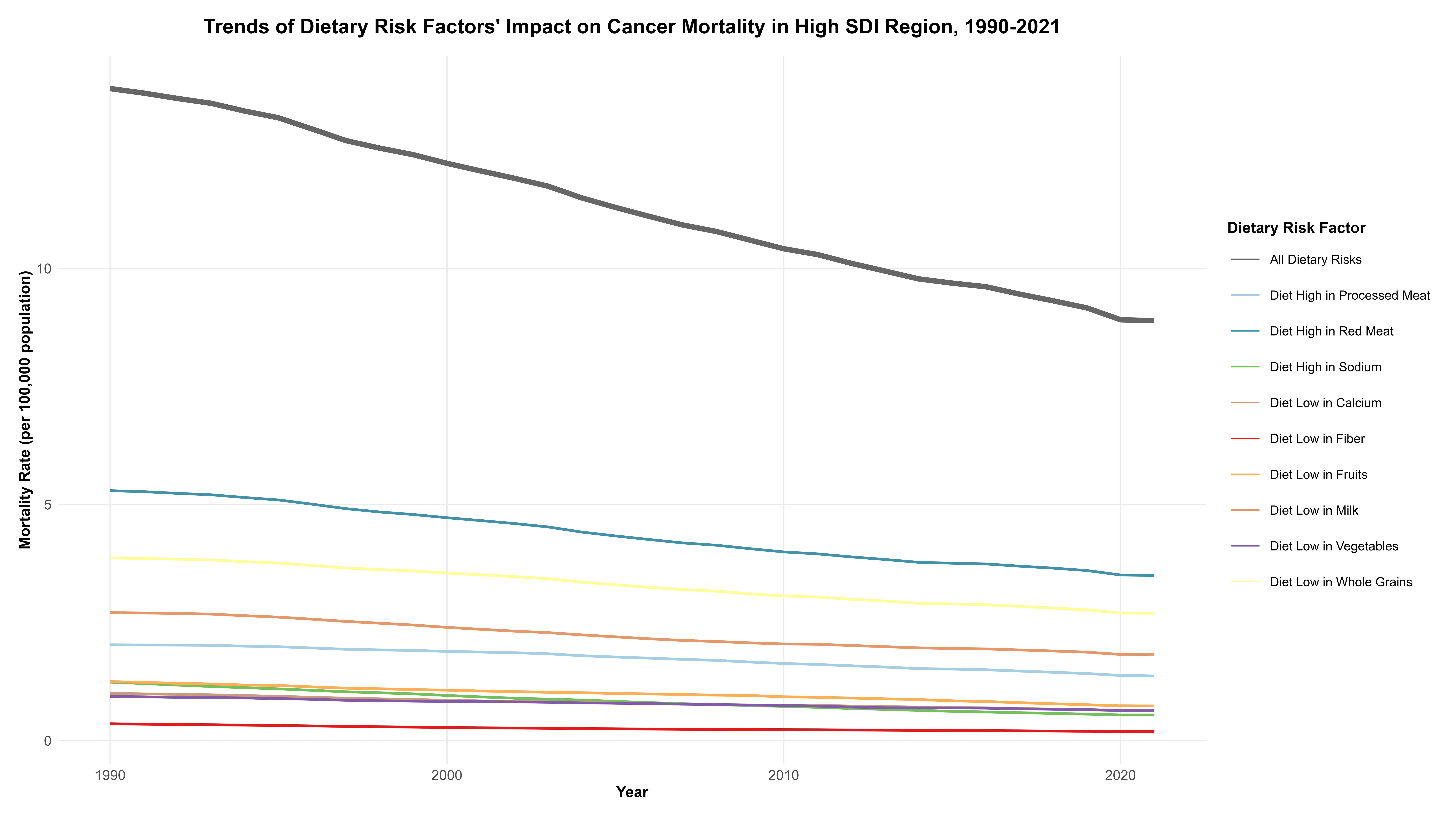

Trends of Dietary Risk Factors' Impact on Cancer Mortality in High SDI Region, 1990-2021
Mortality Rate (per 100,000 population)
Year
0
5
10
1990
2000
2010
2020
Dietary Risk Factor
All Dietary Risks
Diet High in Processed Meat
Diet High in Red Meat
Diet High in Sodium
Diet Low in Calcium
Diet Low in Fiber
Diet Low in Fruits
Diet Low in Milk
Diet Low in Vegetables
Diet Low in Whole Grains

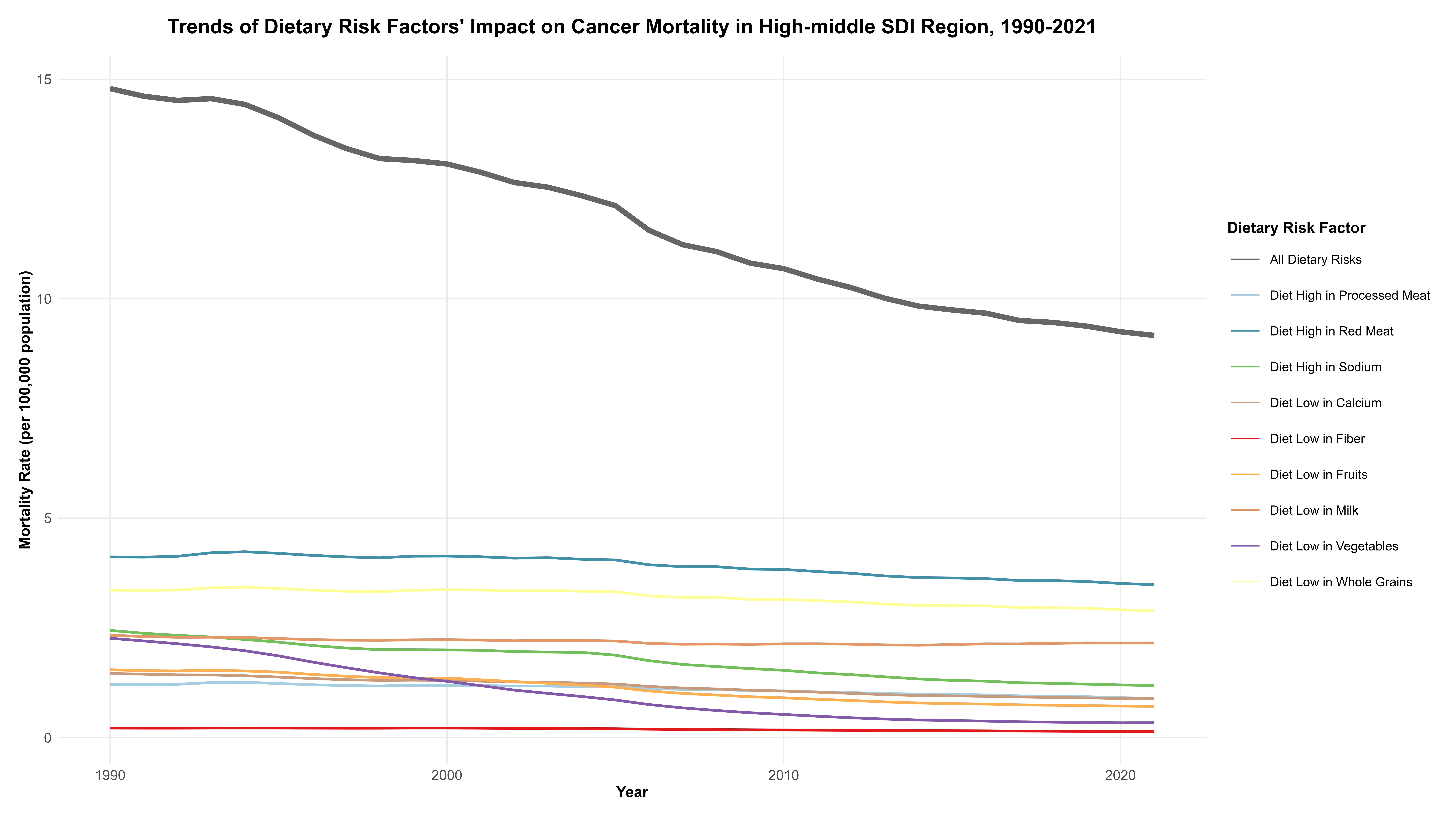
Trends of Dietary Risk Factors' Impact on Cancer Mortality in High-middle SDI Region, 1990-2021
Mortality Rate (per 100,000 population)
15
10
5
0
1990
2000
2010
2020
Year
Dietary Risk Factor
All Dietary Risks
Diet High in Processed Meat
Diet High in Red Meat
Diet High in Sodium
Diet Low in Calcium
Diet Low in Fiber
Diet Low in Fruits
Diet Low in Milk
Diet Low in Vegetables
Diet Low in Whole Grains

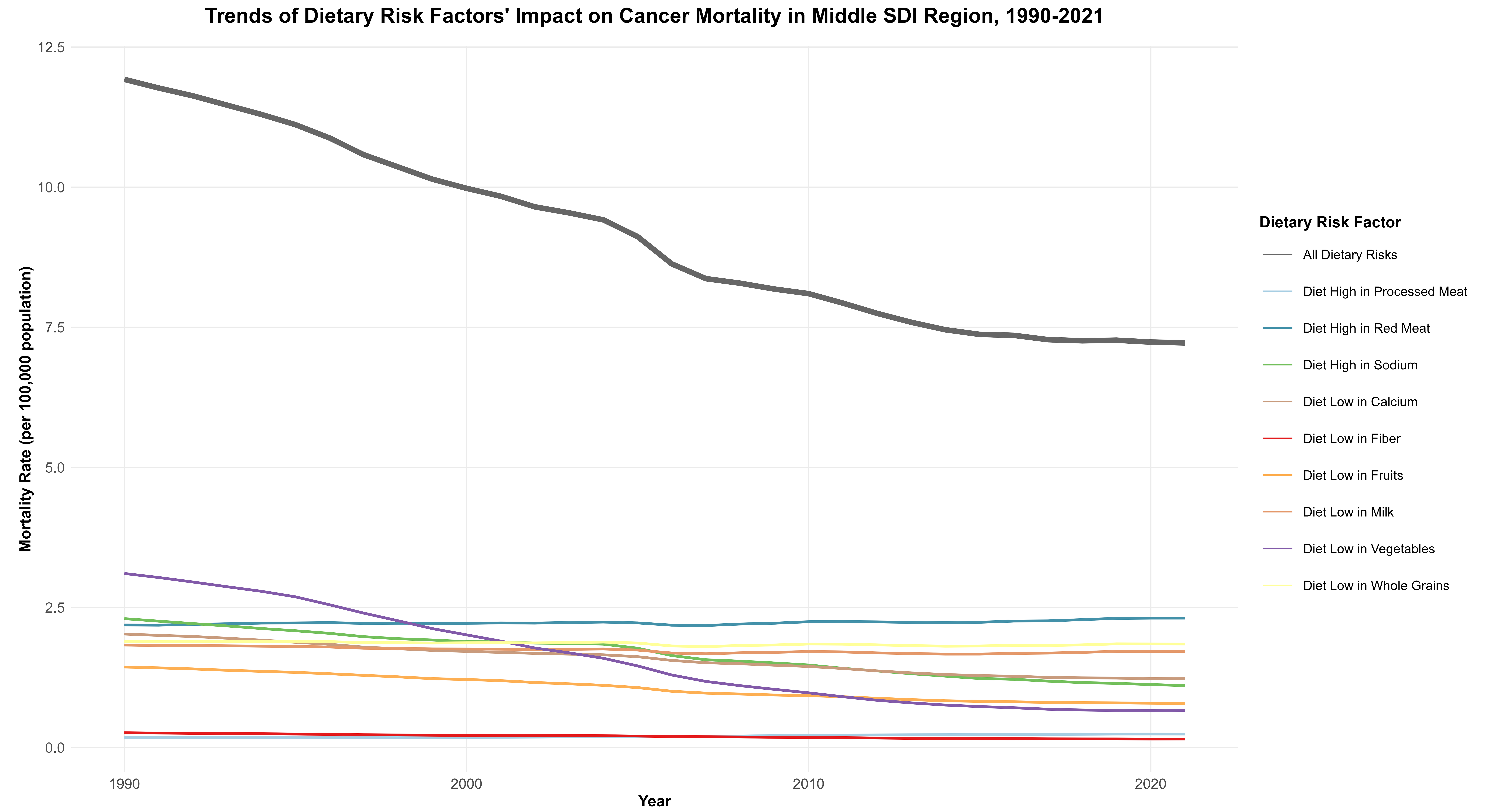
Trends of Dietary Risk Factors' Impact on Cancer Mortality in Middle SDI Region, 1990-2021
Mortality Rate (per 100,000 population)
12.5
10.0
7.5
5.0
2.5
0.0
1990
2000
2010
2020
Year
Dietary Risk Factor
All Dietary Risks
Diet High in Processed Meat
Diet High in Red Meat
Diet High in Sodium
Diet Low in Calcium
Diet Low in Fiber
Diet Low in Fruits
Diet Low in Milk
Diet Low in Vegetables
Diet Low in Whole Grains

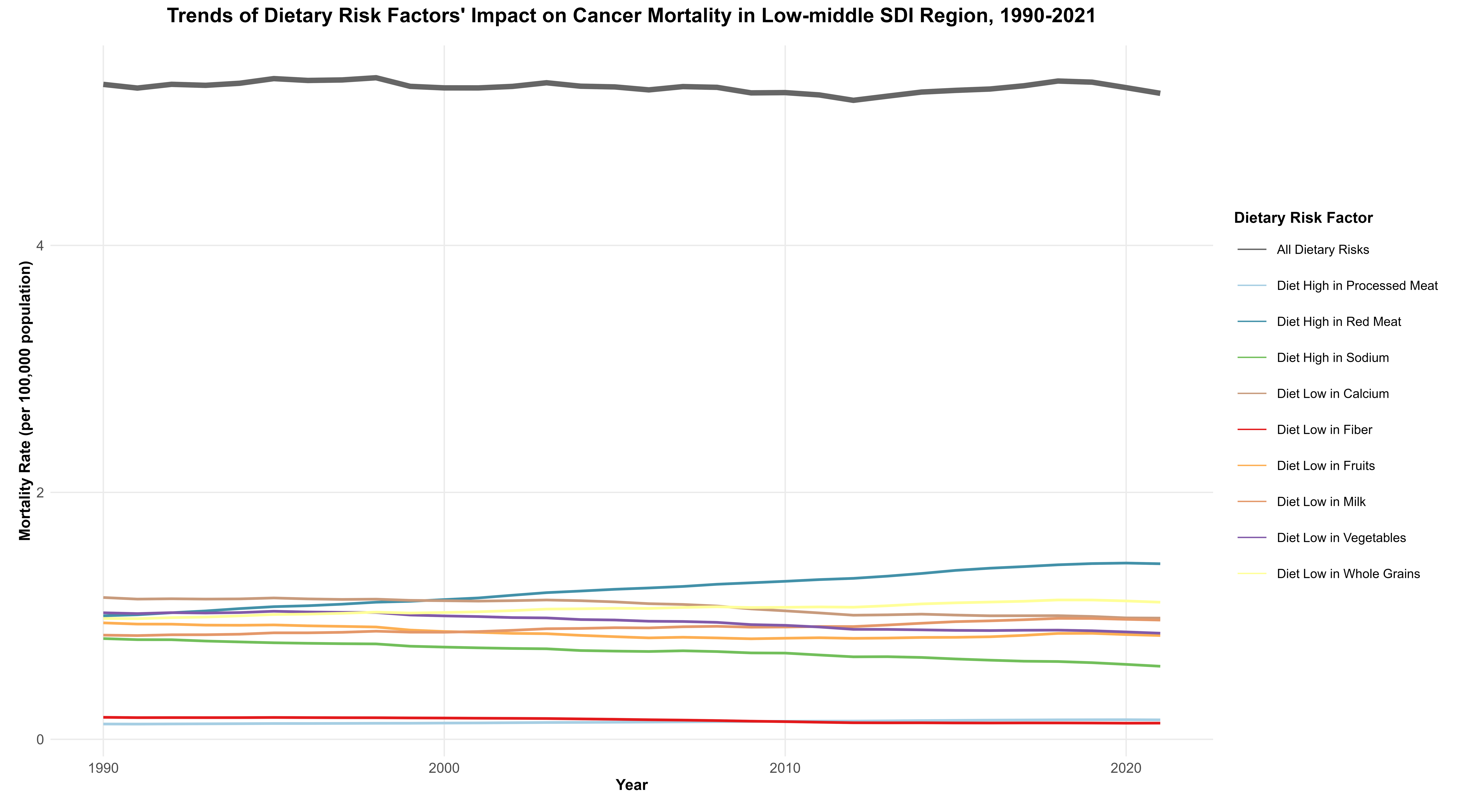

Trends of Dietary Risk Factors' Impact on Cancer Mortality in Low-middle SDI Region, 1990-2021
Mortality Rate (per 100,000 population)
0
2
4
Year
1990
2000
2010
2020
Dietary Risk Factor
All Dietary Risks
Diet High in Processed Meat
Diet High in Red Meat
Diet High in Sodium
Diet Low in Calcium
Diet Low in Fiber
Diet Low in Fruits
Diet Low in Milk
Diet Low in Vegetables
Diet Low in Whole Grains

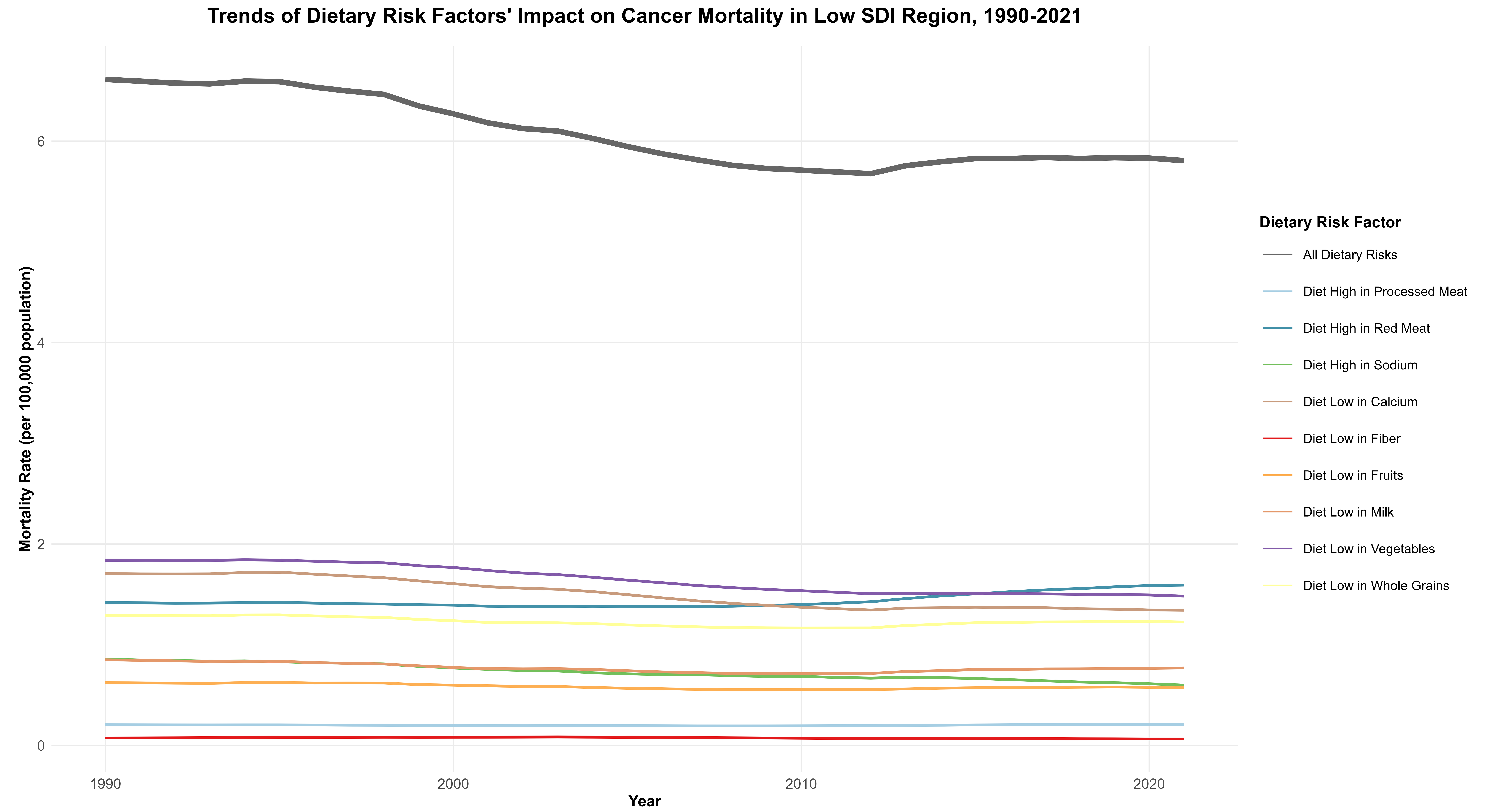
Trends of Dietary Risk Factors' Impact on Cancer Mortality in Low SDI Region, 1990-2021
Mortality Rate (per 100,000 population)
0
2
4
6
1990
2000
2010
2020
Year
Dietary Risk Factor
All Dietary Risks
Diet High in Processed Meat
Diet High in Red Meat
Diet High in Sodium
Diet Low in Calcium
Diet Low in Fiber
Diet Low in Fruits
Diet Low in Milk
Diet Low in Vegetables
Diet Low in Whole Grains
