# Supplementary material for: Dietary risk factors and cancer mortality burden from 1990 to 2021: a comparative study of China and global regions with varying sociodemographic development levels based on the Global Burden of Disease database
Source: Front Nutr. 2025 Aug 5;12:1628792. doi: 10.3389/fnut.2025.1628792 (PMC12360945; doi:10.3389/fnut.2025.1628792)
Supplement: Supplementary file 4 [file Image_3.pdf]

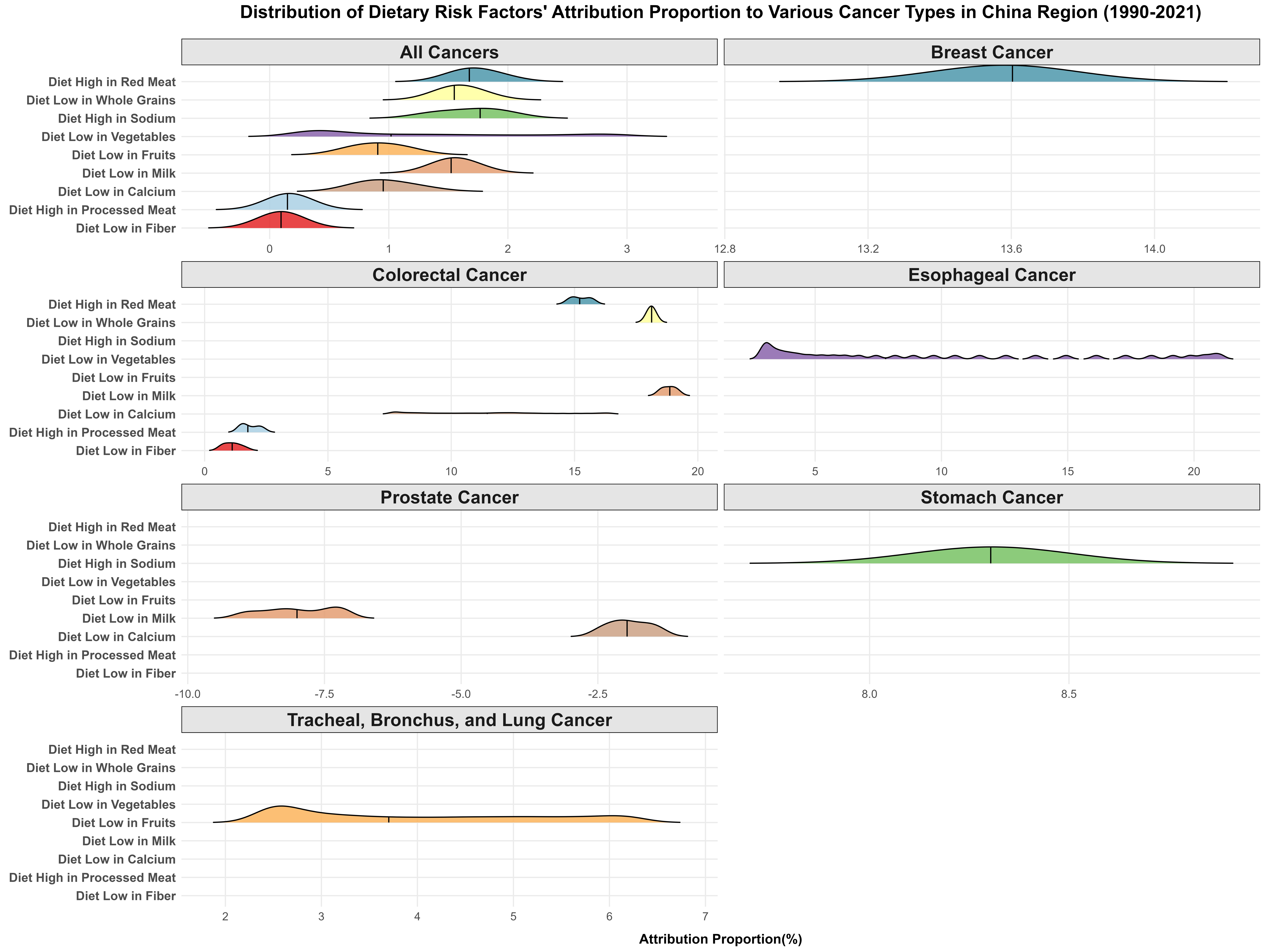
Distribution of Dietary Risk Factors' Attribution Proportion to Various Cancer Types in China Region (1990-2021)
All Cancers
Breast Cancer
Colorectal Cancer
Esophageal Cancer
Prostate Cancer
Stomach Cancer
Tracheal, Bronchus, and Lung Cancer
Diet High in Red Meat
Diet Low in Whole Grains
Diet High in Sodium
Diet Low in Vegetables
Diet Low in Fruits
Diet Low in Milk
Diet Low in Calcium
Diet High in Processed Meat
Diet Low in Fiber
Attribution Proportion(%)

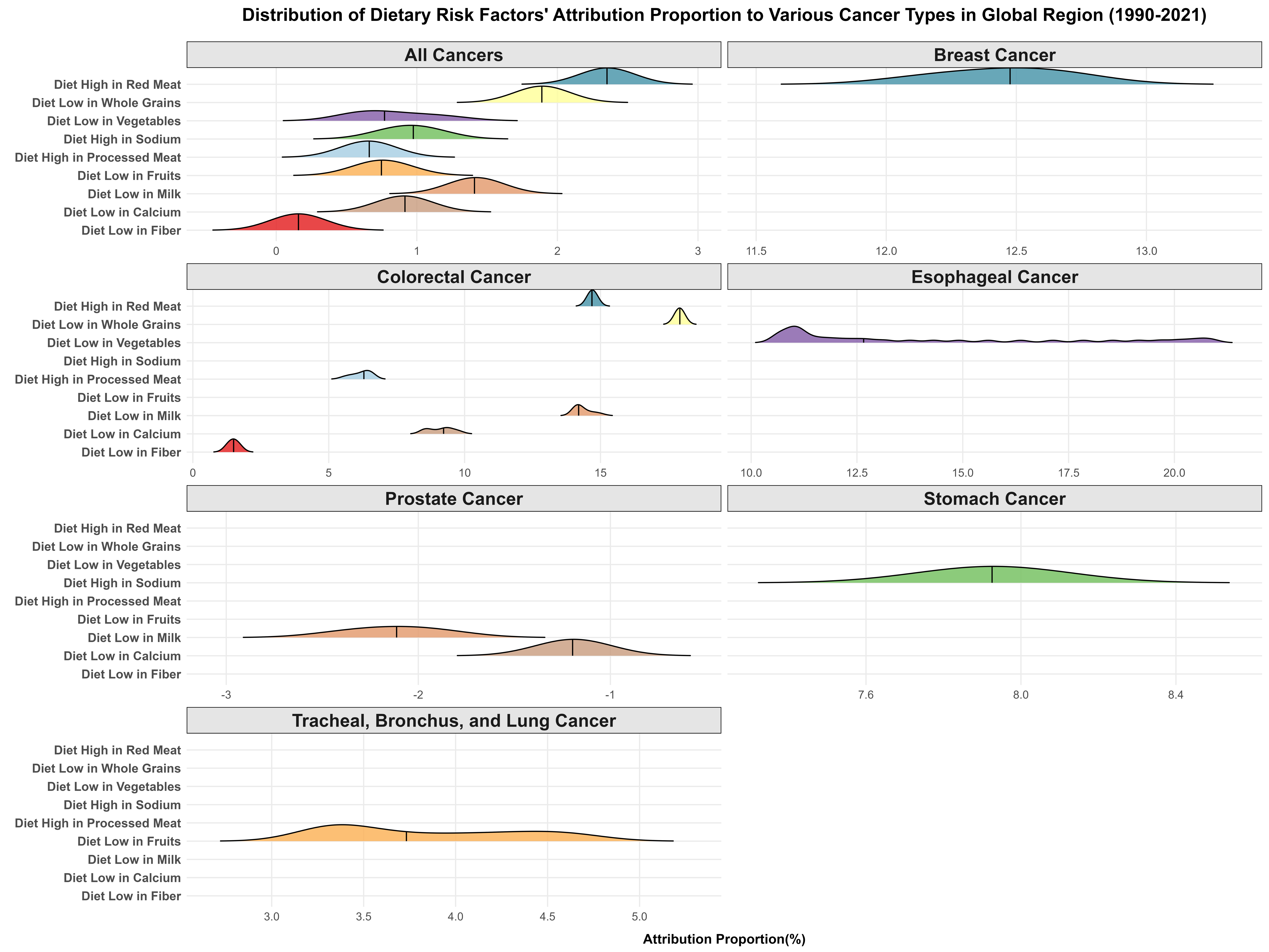
Distribution of Dietary Risk Factors' Attribution Proportion to Various Cancer Types in Global Region (1990-2021)
All Cancers
Breast Cancer
Colorectal Cancer
Esophageal Cancer
Prostate Cancer
Stomach Cancer
Tracheal, Bronchus, and Lung Cancer
Diet High in Red Meat
Diet Low in Whole Grains
Diet Low in Vegetables
Diet High in Sodium
Diet High in Processed Meat
Diet Low in Fruits
Diet Low in Milk
Diet Low in Calcium
Diet Low in Fiber
0
1
2
3
11.5
12.0
12.5
13.0
5
10
15
10.0
12.5
15.0
17.5
20.0
-3
-2
-1
7.6
8.0
8.4
3.0
3.5
4.0
4.5
5.0
Attribution Proportion(%)

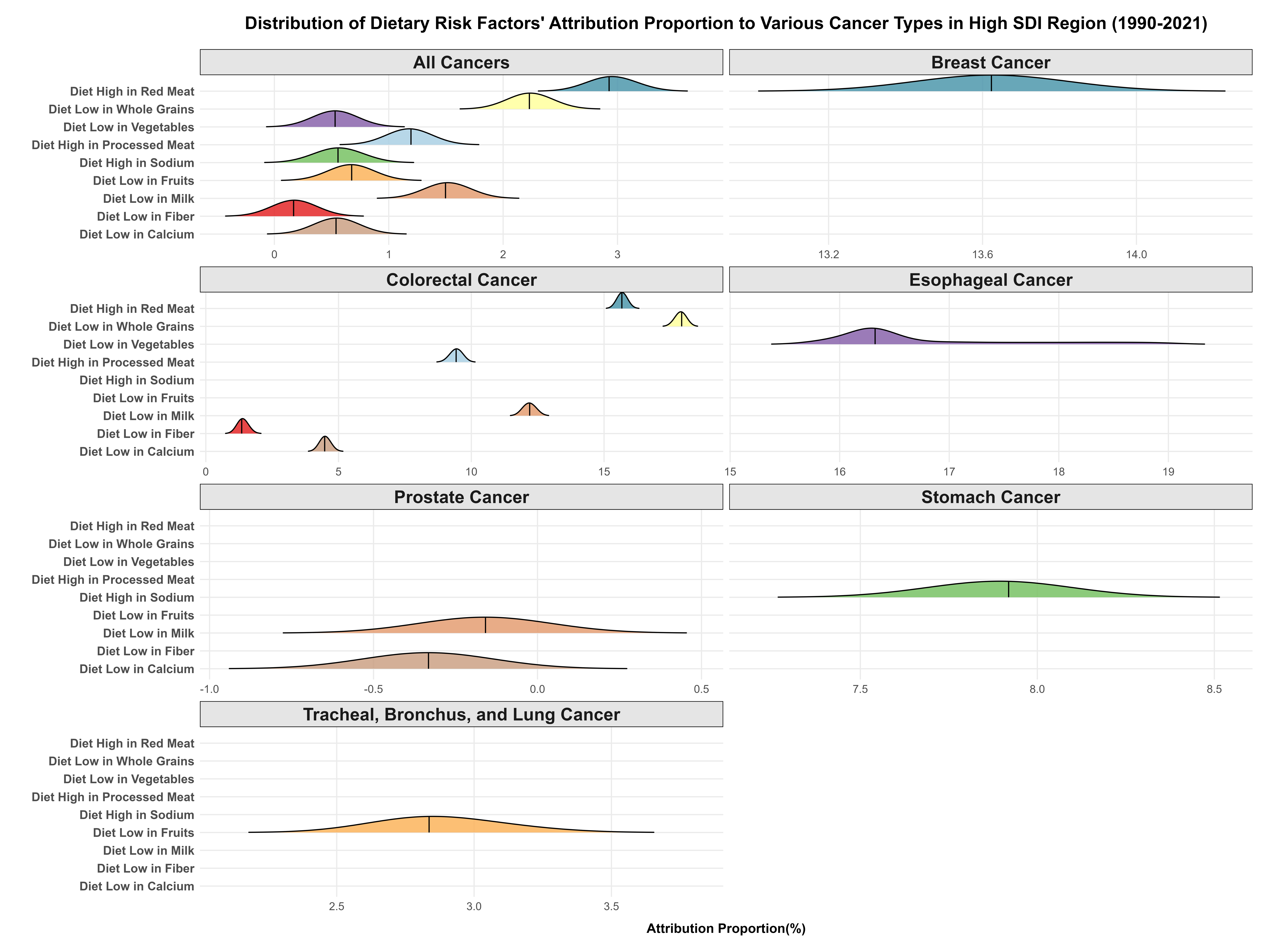

Distribution of Dietary Risk Factors' Attribution Proportion to Various Cancer Types in High SDI Region (1990-2021)
All Cancers
Breast Cancer
Colorectal Cancer
Esophageal Cancer
Prostate Cancer
Stomach Cancer
Tracheal, Bronchus, and Lung Cancer
Diet High in Red Meat
Diet Low in Whole Grains
Diet Low in Vegetables
Diet High in Processed Meat
Diet High in Sodium
Diet Low in Fruits
Diet Low in Milk
Diet Low in Fiber
Diet Low in Calcium
Attribution Proportion(%)

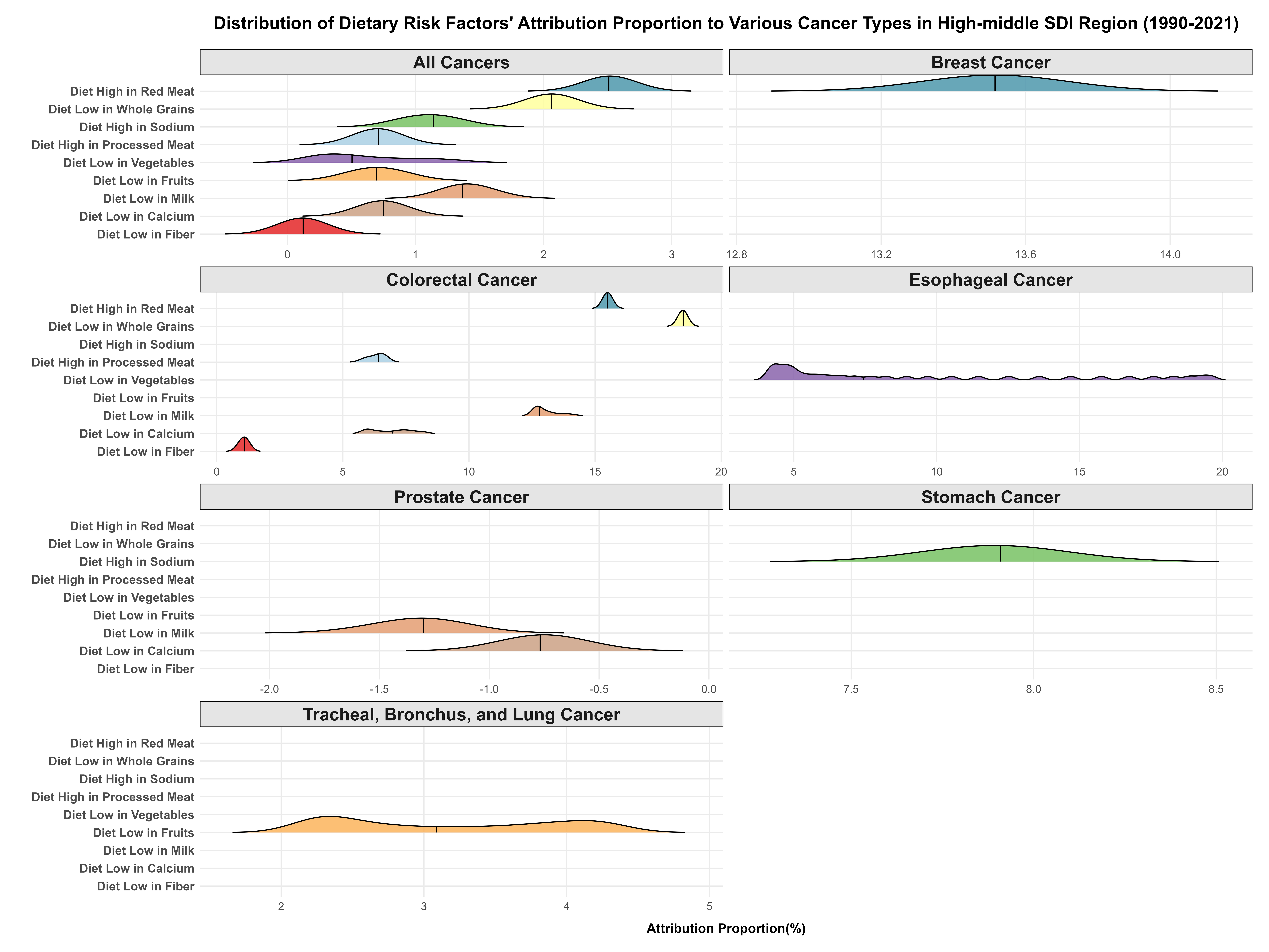

Distribution of Dietary Risk Factors' Attribution Proportion to Various Cancer Types in High-middle SDI Region (1990-2021)
All Cancers
Breast Cancer
Colorectal Cancer
Esophageal Cancer
Prostate Cancer
Stomach Cancer
Tracheal, Bronchus, and Lung Cancer
Diet High in Red Meat
Diet Low in Whole Grains
Diet High in Sodium
Diet High in Processed Meat
Diet Low in Vegetables
Diet Low in Fruits
Diet Low in Milk
Diet Low in Calcium
Diet Low in Fiber
0
1
2
3
12.8
13.2
13.6
14.0
0
5
10
15
20
5
10
15
20
-2.0
-1.5
-1.0
-0.5
0.0
7.5
8.0
8.5
2
3
4
5
Attribution Proportion(%)

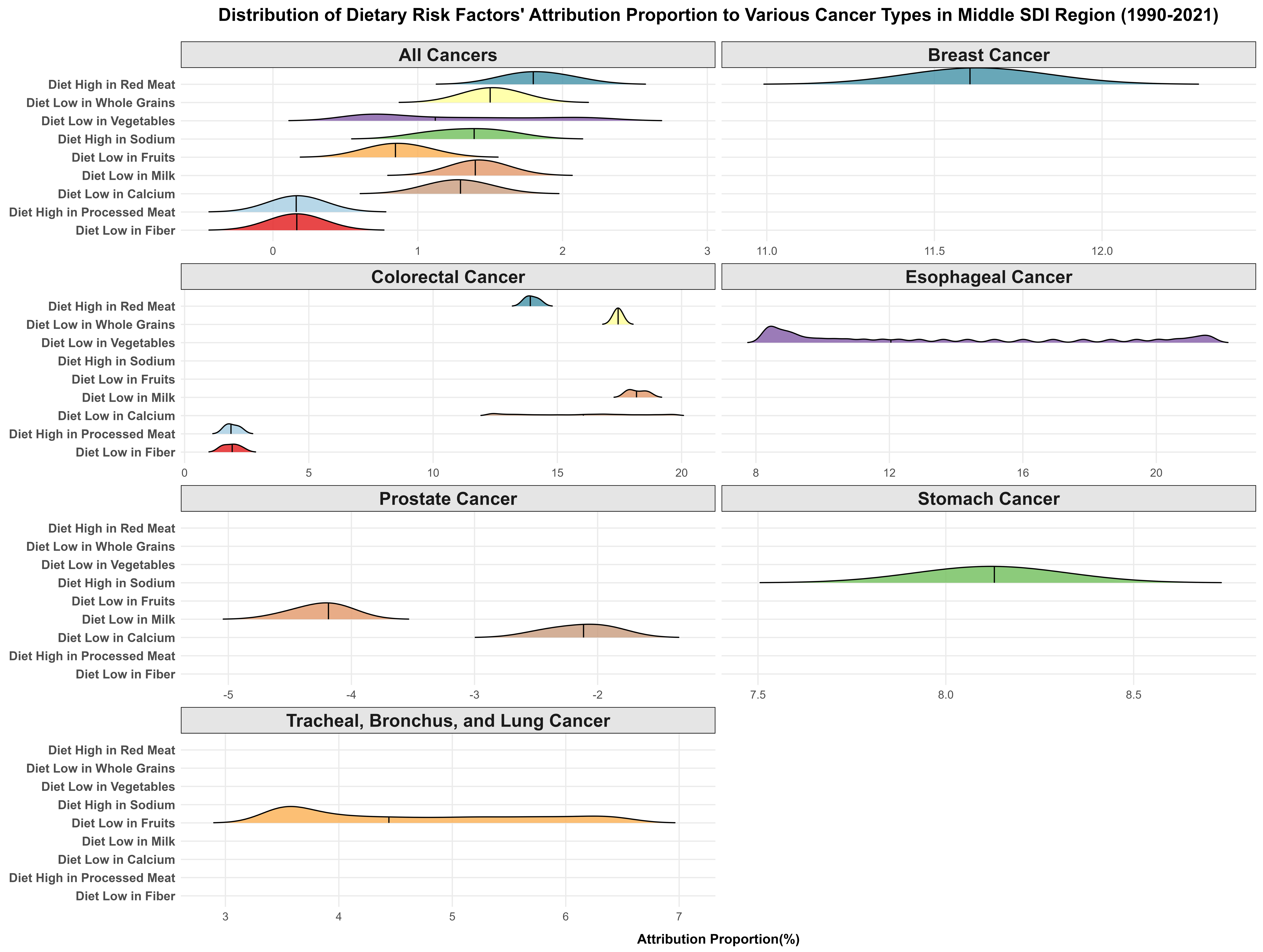
Distribution of Dietary Risk Factors' Attribution Proportion to Various Cancer Types in Middle SDI Region (1990-2021)
All Cancers
Breast Cancer
Colorectal Cancer
Esophageal Cancer
Prostate Cancer
Stomach Cancer
Tracheal, Bronchus, and Lung Cancer
Diet High in Red Meat
Diet Low in Whole Grains
Diet Low in Vegetables
Diet High in Sodium
Diet Low in Fruits
Diet Low in Milk
Diet Low in Calcium
Diet High in Processed Meat
Diet Low in Fiber
Attribution Proportion(%)

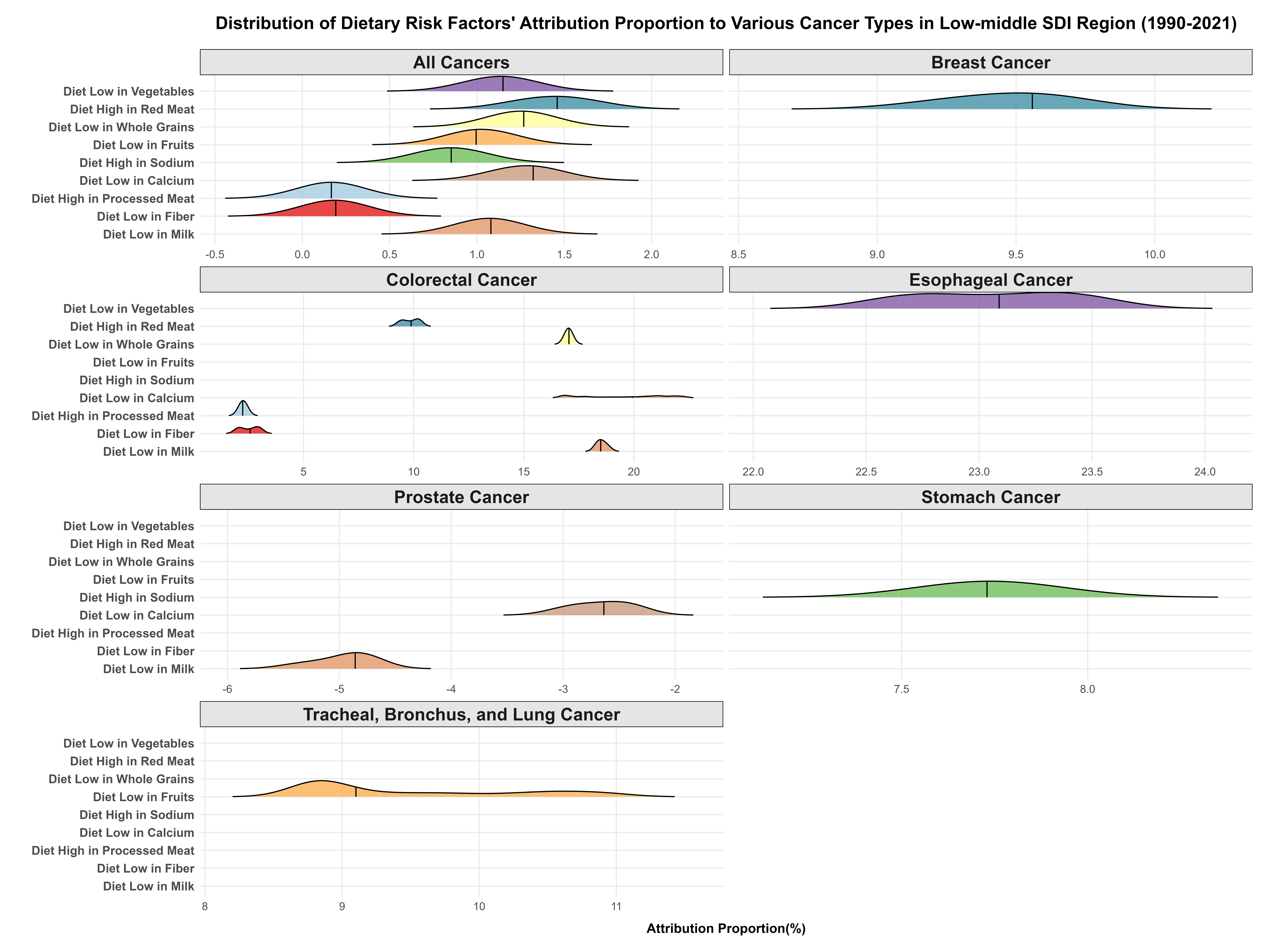

Distribution of Dietary Risk Factors' Attribution Proportion to Various Cancer Types in Low-middle SDI Region (1990-2021)
All Cancers
Breast Cancer
Colorectal Cancer
Esophageal Cancer
Prostate Cancer
Stomach Cancer
Tracheal, Bronchus, and Lung Cancer
Diet Low in Vegetables
Diet High in Red Meat
Diet Low in Whole Grains
Diet Low in Fruits
Diet High in Sodium
Diet Low in Calcium
Diet High in Processed Meat
Diet Low in Fiber
Diet Low in Milk
Attribution Proportion(%)

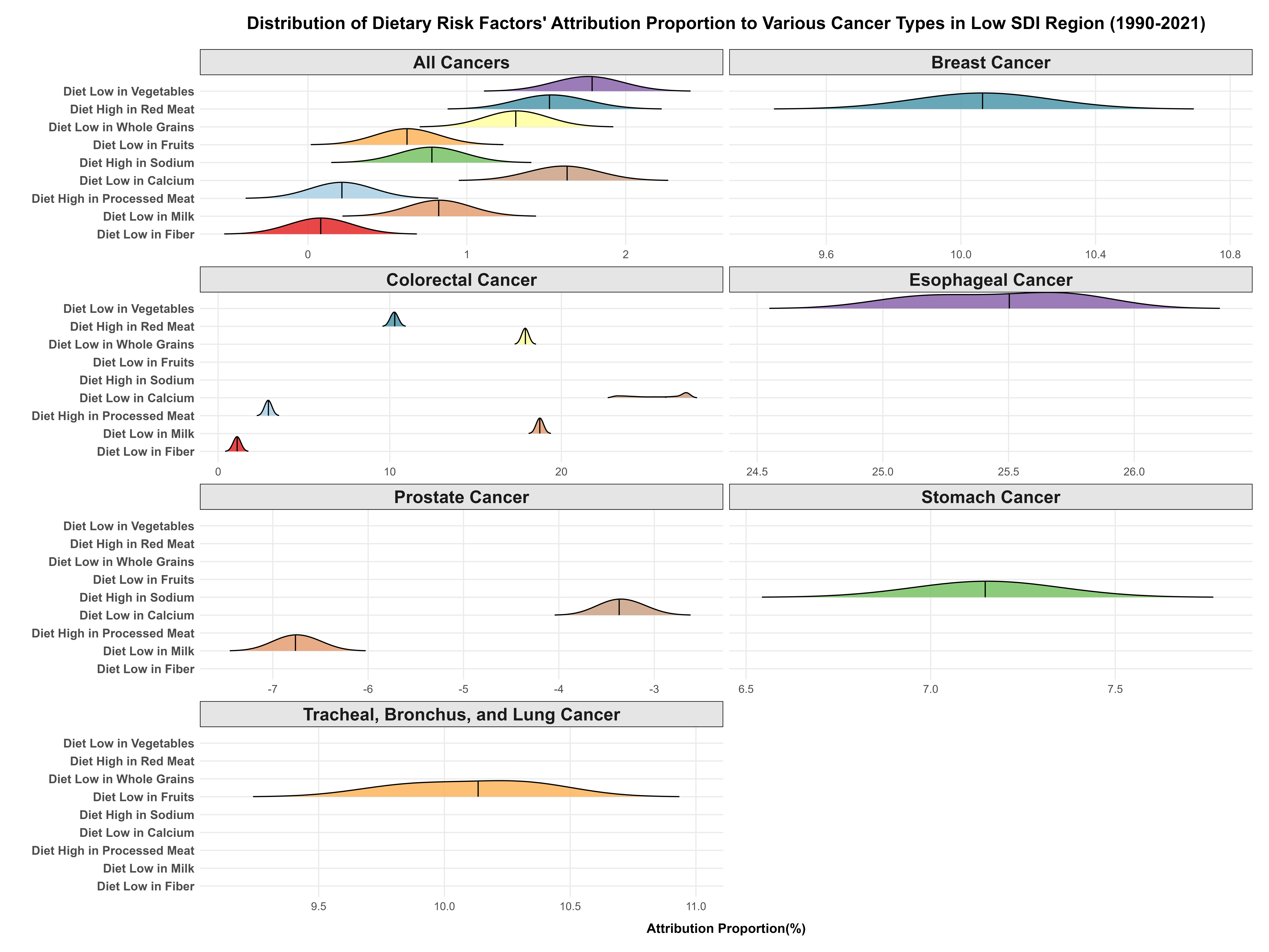

Distribution of Dietary Risk Factors' Attribution Proportion to Various Cancer Types in Low SDI Region (1990-2021)
All Cancers
Breast Cancer
Colorectal Cancer
Esophageal Cancer
Prostate Cancer
Stomach Cancer
Tracheal, Bronchus, and Lung Cancer
Diet Low in Vegetables
Diet High in Red Meat
Diet Low in Whole Grains
Diet Low in Fruits
Diet High in Sodium
Diet Low in Calcium
Diet High in Processed Meat
Diet Low in Milk
Diet Low in Fiber
0
1
2
9.6
10.0
10.4
10.8
0
10
20
24.5
25.0
25.5
26.0
-7
-6
-5
-4
-3
6.5
7.0
7.5
9.5
10.0
10.5
11.0
Attribution Proportion(%)
